# Chromosome-level genome assembly of goose provides insight into the adaptation and growth of local goose breeds

**DOI:** 10.1093/gigascience/giad003

**Published:** 2023-02-03

**Authors:** Qiqi Zhao, Zhenping Lin, Junpeng Chen, Zi Xie, Jun Wang, Keyu Feng, Wencheng Lin, Hongxin Li, Zezhong Hu, Weiguo Chen, Feng Chen, Muhammad Junaid, Huanmin Zhang, Qingmei Xie, Xinheng Zhang

**Affiliations:** Heyuan Branch, Guangdong Provincial Laboratory of Lingnan Modern Agricultural Science and Technology, College of Animal Science, South China Agricultural University, Guangzhou, Guangdong, 510642, China; Department of Science and Technology of Guangdong Province, Key Laboratory of Animal Health Aquaculture and Environmental Control, Guangzhou, Guangdong, 510642, China; Guangdong Engineering Research Center for Vector Vaccine of Animal Virus, Guangzhou, 510642, China; Guangdong Provincial Key Lab of AgroAnimal Genomics and Molecular Breeding, College of Animal Science, South China Agricultural University, Guangzhou, Guangdong, 510642, China; Shantou Baisha Research Institute of Original Species of Poultry and Stock, Shantou, Guangdong, 515000, China; Shantou Baisha Research Institute of Original Species of Poultry and Stock, Shantou, Guangdong, 515000, China; Heyuan Branch, Guangdong Provincial Laboratory of Lingnan Modern Agricultural Science and Technology, College of Animal Science, South China Agricultural University, Guangzhou, Guangdong, 510642, China; Department of Science and Technology of Guangdong Province, Key Laboratory of Animal Health Aquaculture and Environmental Control, Guangzhou, Guangdong, 510642, China; Guangdong Engineering Research Center for Vector Vaccine of Animal Virus, Guangzhou, 510642, China; College of Marine Sciences, South China Agricultural University, Guangzhou, Guangdong, 510642, China; Heyuan Branch, Guangdong Provincial Laboratory of Lingnan Modern Agricultural Science and Technology, College of Animal Science, South China Agricultural University, Guangzhou, Guangdong, 510642, China; Department of Science and Technology of Guangdong Province, Key Laboratory of Animal Health Aquaculture and Environmental Control, Guangzhou, Guangdong, 510642, China; Guangdong Engineering Research Center for Vector Vaccine of Animal Virus, Guangzhou, 510642, China; Heyuan Branch, Guangdong Provincial Laboratory of Lingnan Modern Agricultural Science and Technology, College of Animal Science, South China Agricultural University, Guangzhou, Guangdong, 510642, China; Department of Science and Technology of Guangdong Province, Key Laboratory of Animal Health Aquaculture and Environmental Control, Guangzhou, Guangdong, 510642, China; Guangdong Engineering Research Center for Vector Vaccine of Animal Virus, Guangzhou, 510642, China; Guangdong Provincial Key Lab of AgroAnimal Genomics and Molecular Breeding, College of Animal Science, South China Agricultural University, Guangzhou, Guangdong, 510642, China; Heyuan Branch, Guangdong Provincial Laboratory of Lingnan Modern Agricultural Science and Technology, College of Animal Science, South China Agricultural University, Guangzhou, Guangdong, 510642, China; Department of Science and Technology of Guangdong Province, Key Laboratory of Animal Health Aquaculture and Environmental Control, Guangzhou, Guangdong, 510642, China; Guangdong Engineering Research Center for Vector Vaccine of Animal Virus, Guangzhou, 510642, China; Guangdong Provincial Key Lab of AgroAnimal Genomics and Molecular Breeding, College of Animal Science, South China Agricultural University, Guangzhou, Guangdong, 510642, China; Heyuan Branch, Guangdong Provincial Laboratory of Lingnan Modern Agricultural Science and Technology, College of Animal Science, South China Agricultural University, Guangzhou, Guangdong, 510642, China; Heyuan Branch, Guangdong Provincial Laboratory of Lingnan Modern Agricultural Science and Technology, College of Animal Science, South China Agricultural University, Guangzhou, Guangdong, 510642, China; Department of Science and Technology of Guangdong Province, Key Laboratory of Animal Health Aquaculture and Environmental Control, Guangzhou, Guangdong, 510642, China; Guangdong Engineering Research Center for Vector Vaccine of Animal Virus, Guangzhou, 510642, China; Guangdong Provincial Key Lab of AgroAnimal Genomics and Molecular Breeding, College of Animal Science, South China Agricultural University, Guangzhou, Guangdong, 510642, China; Heyuan Branch, Guangdong Provincial Laboratory of Lingnan Modern Agricultural Science and Technology, College of Animal Science, South China Agricultural University, Guangzhou, Guangdong, 510642, China; Department of Science and Technology of Guangdong Province, Key Laboratory of Animal Health Aquaculture and Environmental Control, Guangzhou, Guangdong, 510642, China; Guangdong Provincial Key Lab of AgroAnimal Genomics and Molecular Breeding, College of Animal Science, South China Agricultural University, Guangzhou, Guangdong, 510642, China; College of Marine Sciences, South China Agricultural University, Guangzhou, Guangdong, 510642, China; Avian Disease and Oncology Laboratory, Agriculture Research Service, United States Department of Agriculture, East Lansing, MI 48823, USA; Heyuan Branch, Guangdong Provincial Laboratory of Lingnan Modern Agricultural Science and Technology, College of Animal Science, South China Agricultural University, Guangzhou, Guangdong, 510642, China; Department of Science and Technology of Guangdong Province, Key Laboratory of Animal Health Aquaculture and Environmental Control, Guangzhou, Guangdong, 510642, China; Guangdong Engineering Research Center for Vector Vaccine of Animal Virus, Guangzhou, 510642, China; Guangdong Provincial Key Lab of AgroAnimal Genomics and Molecular Breeding, College of Animal Science, South China Agricultural University, Guangzhou, Guangdong, 510642, China; Heyuan Branch, Guangdong Provincial Laboratory of Lingnan Modern Agricultural Science and Technology, College of Animal Science, South China Agricultural University, Guangzhou, Guangdong, 510642, China; Department of Science and Technology of Guangdong Province, Key Laboratory of Animal Health Aquaculture and Environmental Control, Guangzhou, Guangdong, 510642, China; Guangdong Engineering Research Center for Vector Vaccine of Animal Virus, Guangzhou, 510642, China; Guangdong Provincial Key Lab of AgroAnimal Genomics and Molecular Breeding, College of Animal Science, South China Agricultural University, Guangzhou, Guangdong, 510642, China

**Keywords:** Lion-head goose, genome assembly, comparative genome, genome-wide association study

## Abstract

**Background:**

*Anatidae* contains numerous waterfowl species with great economic value, but the genetic diversity basis remains insufficiently investigated. Here, we report a chromosome-level genome assembly of Lion-head goose (*Anser cygnoides*), a native breed in South China, through the combination of PacBio, Bionano, and Hi-C technologies.

**Findings:**

The assembly had a total genome size of 1.19 Gb, consisting of 1,859 contigs with an N50 length of 20.59 Mb, generating 40 pseudochromosomes, representing 97.27% of the assembled genome, and identifying 21,208 protein-coding genes. Comparative genomic analysis revealed that geese and ducks diverged approximately 28.42 million years ago, and geese have undergone massive gene family expansion and contraction. To identify genetic markers associated with body weight in different geese breeds, including Wuzong goose, Huangzong goose, Magang goose, and Lion-head goose, a genome-wide association study was performed, yielding an average of 1,520.6 Mb of raw data that detected 44,858 single-mucleotide polymorphisms (SNPs). Genome-wide association study showed that 6 SNPs were significantly associated with body weight and 25 were potentially associated. The significantly associated SNPs were annotated as *LDLRAD4, GPR180*, and *OR*, enriching in growth factor receptor regulation pathways.

**Conclusions:**

We present the first chromosome-level assembly of the Lion-head goose genome, which will expand the genomic resources of the *Anatidae* family, providing a basis for adaptation and evolution. Candidate genes significantly associated with different goose breeds may serve to understand the underlying mechanisms of weight differences.

## Introduction

**Figure 1:**
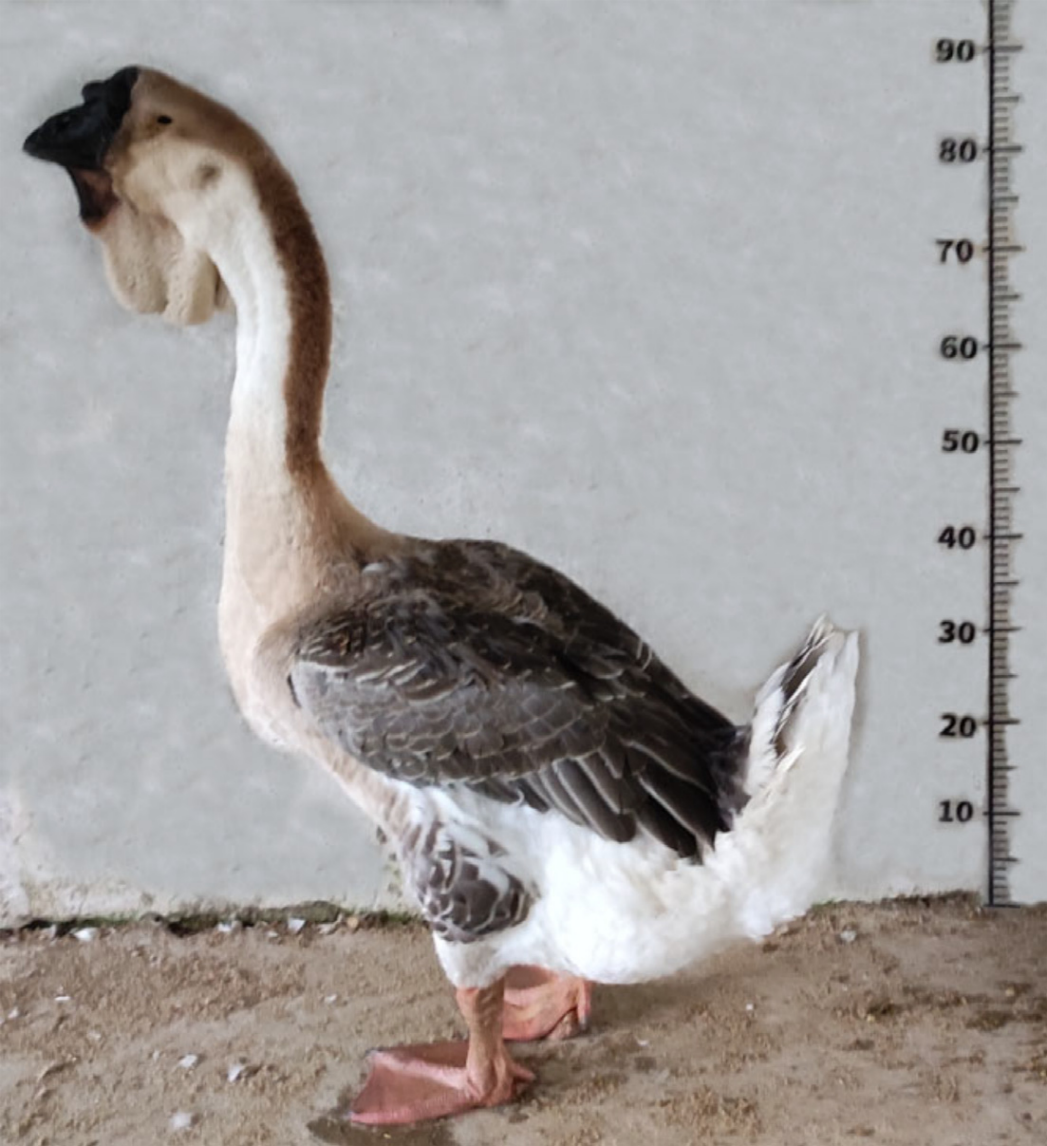
A picture of a male adult Lion-head goose.

The *Anatidae* is a family of the ancient *Aves* class with order *Anseriformes*, containing 43 genera and 174 species, including most birds of the*Anseriformes* order, such as ducks, geese, and swans, and is the most prominent family of swimming birds [1]. Physical characteristics and features vary significantly among species, making the *Anatidae* family rich in diversity and specificity. *Anatidae* adults are usually herbivores, feeding on a variety of aquatic plants, which are well suited to sustainable production practices, thereby reducing competition for human food, and some species are even used for cropping weeds and pest control [1, 2]. For a long time, duck and goose feathers have been popular in pillows, quilts, and coats [3]. Several species in the genus *Anser* are commercially important and domesticated as poultry because of their meat-producing performance and natural stuffing for warm clothing and bedding. According to archaeological evidence, geese were domesticated around 6,000 years ago near the Mediterranean Sea and later spread around the world due to human activities [4]. It is widely believed that *Anser cygnoides* (NCBI:txid8845) is the ancestor of the Chinese goose (*Anser cygnoides domesticus*) with a domestication history of more than 3,000 years [1]. After artificial domestication, the domestic goose has increased its cold tolerance and roughage resistance, but its wings have been degraded and weakened in flight, unable to travel long distances [1]. Egg-laying rate and gosling survival rate also have improved compared to wild swans, and the life span is longer [5]. Furthermore, overfeeding can cause foie gras to be at least 3-fold larger than the normal size while the goose remains healthy, making the goose a good model to study human liver steatosis [6]. Chinese domestic geese are a natural gene pool containing local breeds of diverse phenotypes, and adult domestic geese from similar regions vary greatly in weight [7]. For example, the Lion-head goose in Shantou (116°14′′–117°19′′ E, 23°02′′–23°38′′ N), Guangdong Province, can weigh more than 9 kg, while for the Wuzong goose from Qingyuan (111°55′′–113°55′′ E, 23°31′′–25°12′′ N), Guangdong Province, the average weight is only about 3 kg [8, 9]. The Lion-head goose has a large body, a deep and wide head, and large sarcomas (5 sarcomas) on the front and side of the face (Fig. 1). The adult male goose weighs 9–10 kg and the female goose 7.5–9 kg, grows rapidly, and has rich muscles. The Wuzong goose is a small goose species with a distinct band of black plumage from neck to back. The gander weighs 3–3.5 kg and the female weighs 2.5–3 kg, with a wide and short body, flat back, and thin and short feet. The Magang goose is a medium-sized goose species, with a long head, wide beak, rectangular body, gray-black bristle-like feathers on the back of the neck, gray-brown breast feathers, and white belly feathers. Adult weight is 4–5 kg for males and 3–4 kg for females. The Huangzong goose has a compact body, from the top of the head to the back of the neck, and has a brownish-yellow feather belt, shaped like a horse's mane. The chest feather is gray yellow, the belly feather is white, and the beak and sarcoma are black. Adult males weigh 3–3.5 kg and females 2.5–3 kg. However, the mechanisms for such differences have not been clarified, let alone been resolved at the genomic level. Therefore, a complete, continuous, and accurate reference genome is essential for deciphering genomic diversity and evolutionary and adaptive processes, improving production efficiency, and even developing better tools for breeding to promote the development of the goose industry.

High-quality genome assembly sequences enable us to comprehensively and scientifically decode the genetic diversity of species, explore disease mechanisms, and understand species evolution. Recently, PacBio has offered technology that can generate reads several thousand bases in size, and these long reads can span repetitive regions [10]. Although these long reads have a high error rate, they can be integrated with Illumina's short reads to improve sequencing accuracy [11]. In addition, new scaffolding techniques, such as high-throughput chromosome conformation capture (Hi-C), allow the genome to be assembled to the level of whole chromosomes [12]. PacBio single-molecule real-time (SMRT) sequencing technology has been extensively used in the study of human diseases such as tuberculosis and influenza virus [13], as well as in the study of species evolution, such as the centromere of the human Y chromosome [14]. Bionano optical mapping technology has advantages in obtaining highly repetitive sequences and detecting genomic structural variants, which is helpful for remote sequencing of sequence overlap clusters[15]. Bionano has become a powerful tool for genome assembly: a 5.1-Mbp inversion was found in the genomes of a patient with Duchenne muscular dystrophy [16].

In this study, we report the genome assembly at the chromosome level in Lion-head geese for the first time using combined data generated by 4 advanced technologies: Illumina, SMRT, Bionano, and Hi-C. In addition, we investigated the relationship between body weight and genetic variations in Lion-head goose, Wuzong goose, Huangzong goose, and Magang goose by genome-wide association analysis, trying to identify the genes involved in body weight determination from different species. These will offer valuable resources for facilitating genetic research and the improvement of the species and for studying speciation and evolution in geese.

## Methods

### Animal selection

An adult healthy purebred male Lion-head goose (*Anser cygnoides*) with classical traits was selected for whole-genome sequencing and conducting *de novo* assembly from the Shantou Baisha Research Institute of Original Species of Poultry and Stock. Blood and 8 tissues (i.e., brain, pharyngeal pouch, head sarcoma, spleen, liver, chest muscle, kidney, and heart) from another 4 healthy adult individuals were collected for RNA-sequencing (RNA-seq) analysis. All applicable institutional and national guidelines for the care and use of animals were followed. All the animal work in this study was approved by the South China Agricultural University Committee for Animal Experiments (approval ID: SYXK 2019–0136). All the research procedures and animal care activities were conducted based on the principles stated in the National and Institutional Guide for the Care and Use of Laboratory Animals.

### Genome survey library construction and sequencing

To survey the genome profile, high-quality genomic DNA was extracted from the blood of the reference individual for whole-genome sequencing using the Blood and Cell Culture DNA Midi Kit (Qiagen, Hilden, Germany) according to the manufacturer's instructions. For the quality control of purity, concentration, and integrity, we used Qubit 2.0 Fluorometry (Life Technologies, Waltham, MA, USA), NanoDrop 2000 spectrophotometer (Thermo Scientific, Waltham, MA, USA), and pulse-field gel electrophoresis (CHEF-DR II; Bio-Rad, California, USA), respectively. The following steps used for DNA extraction and quality control were similar. The short paired-end Illumina DNA library was constructed using the Illumina HiSeq X Ten system (RRID:SCR_016385) with the paired-end 350-bp sequencing strategy. After performing the sequencing and obtaining the data, the *k*-mer analysis of reads for the genome survey was calculated by the Jellyfish (RRID:SCR_005491) program with the default parameters. Additionally, the genome size, heterozygosity ratio, and repeat sequence ratio were calculated with the GenomeScope (RRID:SCR_017014) tool based on the *k*-mer frequency of 17.

### Genome sequencing and assembly strategies

A 40-kb *de novo* library for SMRT genome sequencing was constructed using the PacBio Sequel II platform (RRID:SCR_017990) (Pacific Biosciences, California, USA). All of these reads were used for contig assembly. A scalable and accurate long-read assembly tool, Canu (RRID:SCR_015880) v1.8 [17], was employed to correct and assemble the PacBio reads with the listed parameters (minThreads = 4, genome size = 1,200 m, minOverlapLength = 700, minReadLength = 1,000). The resulting contigs and corrected reads were used as inputs for HERA [18] to fill the gaps and produce longer contigs with default parameters. After that, Illumina paired-end clean data were mapped to the corrected contigs with the Burrows–Wheeler Aligner (RRID:SCR_010910) [19], and the results were filtered by Q30 with Samtools (RRID:SCR_002105) v1.8 [20]. Finally, Pilon (RRID:SCR_014731) v1.22 [21] was used to polish the assembly and enhance the base accuracy of the contigs.

Physical optical genome maps from BioNano were used to improve the assembly quality of the genome, with the ultimate goal of generating a chromosome-scale assembly. Nuclear DNA was extracted from the blood sample of the reference individual and digested with nickase Direct Labeling Enzyme Ⅰ. After labeling, repairing, and staining reactions, DNA was loaded onto the Saphyr Chip for sequencing to generate BioNano molecules. Afterward, the data were assembled with RefAligner and Assembler of BioNano Solve. The scaffold was established using BioNano Solve with HERA's contigs and a BioNano genome map. When encountering a conflict between a contig and the BioNano genome map, the contig was split by the program “hybridScaffold.pl” to correct the false connection. In brief, a pattern search of the genomic sequence was first performed to find possible cleavage labels, and the number of labels on matched and unmatched pairs in each linkage was counted and their position was recorded. Supervised processing was then performed to resolve the positions with conflicting match, and then RefAligner was called for an iterative sequence merge by pairwise alignment. Finally, the sequence map and genome map were rematched to the hybrid scaffold and checked again.

For Hi-C library, fresh blood was vacuum-infiltrated with 2% formaldehyde solution and then used for cross-link action. Later, nuclear DNA was isolated from the reference animal and digested with the restriction enzyme Mbo I. The Hi-C library with insertion sizes of 350 bp was constructed and sequenced on the Illumina HiSeq X Ten instrument. The Hi-C reads were assigned to the scaffolds by Juicer (RRID:SCR_017226) [22]. The scaffolds were further clustered, ordered, and oriented to the chromosome-level scaffolds by 3D-DNA [23]. Thus, a heatmap of Hi-C chromosomal interaction was created using the HiC-Pro software (RRID:SCR_017643) [24].

### RNA-seq and transcripts assembly

RNA-seq was conducted on blood and 8 different tissues (i.e., brain, pharyngeal pouch, head sarcoma, spleen, liver, chest muscle, kidney, and heart) from 4 healthy adult Lion-head geese. Total RNA was extracted from 4 individuals using the TRIZOL reagent and purified following the manufacturer's protocols. The concentration and quality of the isolated RNA were assessed using the Nanodrop Spectrophotometer, Qubit 2.0 Fluorometry, and the Agilent 2100 bioanalyzer (Agilent Technologies, California, USA). Library construction and sequencing were performed using the Illumina NovaSeq 6000 Sequencing System (RRID:SCR_016387). Raw RNA-seq data with 150-bp paired-end reads were trimmed for quality using Trimmomatic (RRID:SCR_011848) [25]. Thus, the Illumina sequence adapters were removed, and then low-quality reads based on Phred scores, adapter-polluted reads containing >5 adapter-polluted bases, and those containing N > 5% were trimmed, using the following parameters: LEADING:3 TRAILING:3 SLIDINGWINDOW:4:15 -threads 20 MINLEN:50. Furthermore, Trinity [26] was used to *de novo* assemble the data after quality filtering. To remove redundant sequences, CD-HIT (RRID:SCR_007105) [27] was employed to remove highly identical transcript isoforms, retaining only the longest one. After filtering, the RNA-seq reads were mapped to the assembled genome using the default parameters of STAR [28].

### Assembly evaluation

Finishing the genome assembly, quality control for the assembly's quality, accuracy, and integrity was assessed by BUSCO (RRID:SCR_015008), v 5.3.0, using aves_odb10 as the query with the following parameters: -l aves_odb10 -m genome -c 5 [29, 30].

### Genome annotation

The genome assembly was annotated by MAKER (RRID:SCR_005309), mainly including gene annotation and repeat annotation. The detailed pipeline was based on proteins from the Uniprot, the *de novo* assembly of RNA-seq data, and the total proteins of the relative species *A. cygnoides* [31]. The transposable element (TE) associated genes that were filtered out by the TEseeker database and the results were used to conduct functional annotation using InterProScan. The repeat sequencing library was identified and annotated by a combination of LTR-FINDER and RepeatModeler (RRID:SCR_015027). RepeatMasker and the query species “Chicken” were used to mask the repeats in the assembly, based on the Repbase database and the previous repeat sequence library. Tandem repeats were discovered by the Tandem Repeats Finder [32].

### Gene families and phylogenetic analysis

Interspecific syntenic blocks between the Lion-head goose and duck were explored using MCscan (RRID:SCR_017650) [33] after coding sequence alignment by BLASTN (RRID:SCR_001598). The same method was used for intraspecific collinearity analysis. To gain insight into the gene family evolution of the goose, we compared the gene families of the Lion-head goose with the genomes of the following avian species: Zhedong white goose (*Anser cygnoides*), duck (*Anas platyrhynchos*), turkey (*Meleagris gallopavo*), chicken (*Gallus gallus*), pigeon (*Columba livia*), saker (*Falco cherrug*), titmouse (*Pseudopodoces humilis*), and green lizard (*Anolis carolinensis*). Initially, alternative splicing and genes encoding fewer than 50 amino acids with a proportion of stop codons greater than 20% were filtered; meanwhile, the longest transcript of genes with multiple isoforms was retained to represent the gene. Similarity relationships among the protein sequences of species were aligned by the BLASTP (RRID:SCR_001010) algorithm and clustered using OrthoMCL methodology with an expansion coefficient of 1.5 to obtain single- and multiple-copy gene families and specific gene families of Lion-head goose. The sequences of the single-copy gene families were employed to perform multiple alignments by MUSCLE (RRID:SCR_011812). Then, RAxML (RRID:SCR_006086) [34] was used to construct a phylogenetic tree of 9 species, with the green lizard (*Anolis carolinensis*) being designated an out-group. Taking the divergence time of the pigeon and turkey (92.9 million years ago [Mya]) as the calibration, the r8s (RRID:SCR_021161) [35] software was used to estimate the divergence time of the species and construct ultrametric trees. After filtering out gene families with gene counts of more than 100 in some individual species, CAFÉ (RRID:SCR_005983) [36] was employed to detect gene families that had undergone expansion or contraction per million years independently along each branch of the phylogenetic tree. Subsequently, a gene ontology (GO) enrichment analysis of gene families was performed using the clusterProfiler package in R [37].

### Experimental sample processing and variant detection for genome-wide association study

Blood samples of 514 geese (including Lion-head goose, Wuzong goose, Huangzong goose, and Magang goose) were collected and stored in 2-mL tubes containing ACD anticoagulant for DNA extraction, and the weight of the geese was recorded. DNA was extracted from blood samples using the HiPure Blood DNA Mini Kit (Magenbio, Guangzhou, China). The samples that passed the quality testing were subjected to library construction using the Easy DNA Library Prep Kit (MGI, Shenzhen, China) and paired-end 100 sequencing using BGIseq 500 (RRID:SCR_017979). Raw data were filtered for adapters and low-quality reads using SOAPnuke software, low-quality threshold parameters were set to 20, and the filtered sequences were compared with the constructed goose reference genome using Burrows–Wheeler Aligner software with the following parameters: mem, -M. Then, variant detection was performed using Samtools, GATK4 software with the following parameters: HaplotypeCaller –ERC GVCF. Single-nucleotide polymorphism (SNP) variants were filtered based on a minimum allele frequency threshold of 0.05, a Hardy–Weinberg equilibrium test significance threshold of 10^−7^, and a maximum missing rate threshold of 0.7. Principal component analysis (PCA) was performed and plotted with R. To understand relationships among groups of the samples, the phylogenetic trees were constructed using SNP data with Phylip software.

### Genome-wide association study

Based on the SNP set obtained above, the genetic variation was analyzed with individual corresponding body weight information using the 2 separate and independent models to assess the significance of SNP effects in Plink (RRID:SCR_001757) v1.90b6.21 [38]. In the first model, top 20 principal components from the PCA analysis were used as covariates, and a linear analysis was performed on sample variances with the following parameters: –linear –allow-extra-chr –allow-no-sex –covar. In the second model, an asymptotic Wald test analysis was carried out with the following parameters: –assoc –allow-extra-chr –allow-no-sex. Finally, SNPs with Bonferroni-corrected *P* values less than 0.05 were taken as significant loci in the SNPs obtained from the 2 models above, and these loci were annotated. The annotated genes were subjected to GO enrichment analysis using the genomic genes of Lion-head goose as background.

### Selective-sweep analysis

To analyze regions affected by long-term selection and associated with domestication of geese, we calculated the Fixation indices (F_ST_) for 4 goose species using vcftools software with sliding windows length of 20 kb that had a 10-kb overlap between adjacent windows. The top 5% of regions were designated as candidate selective regions, and the genes in these regions were considered candidate genes.

## Results

### Genome sequencing and assembly

**Figure 2:**
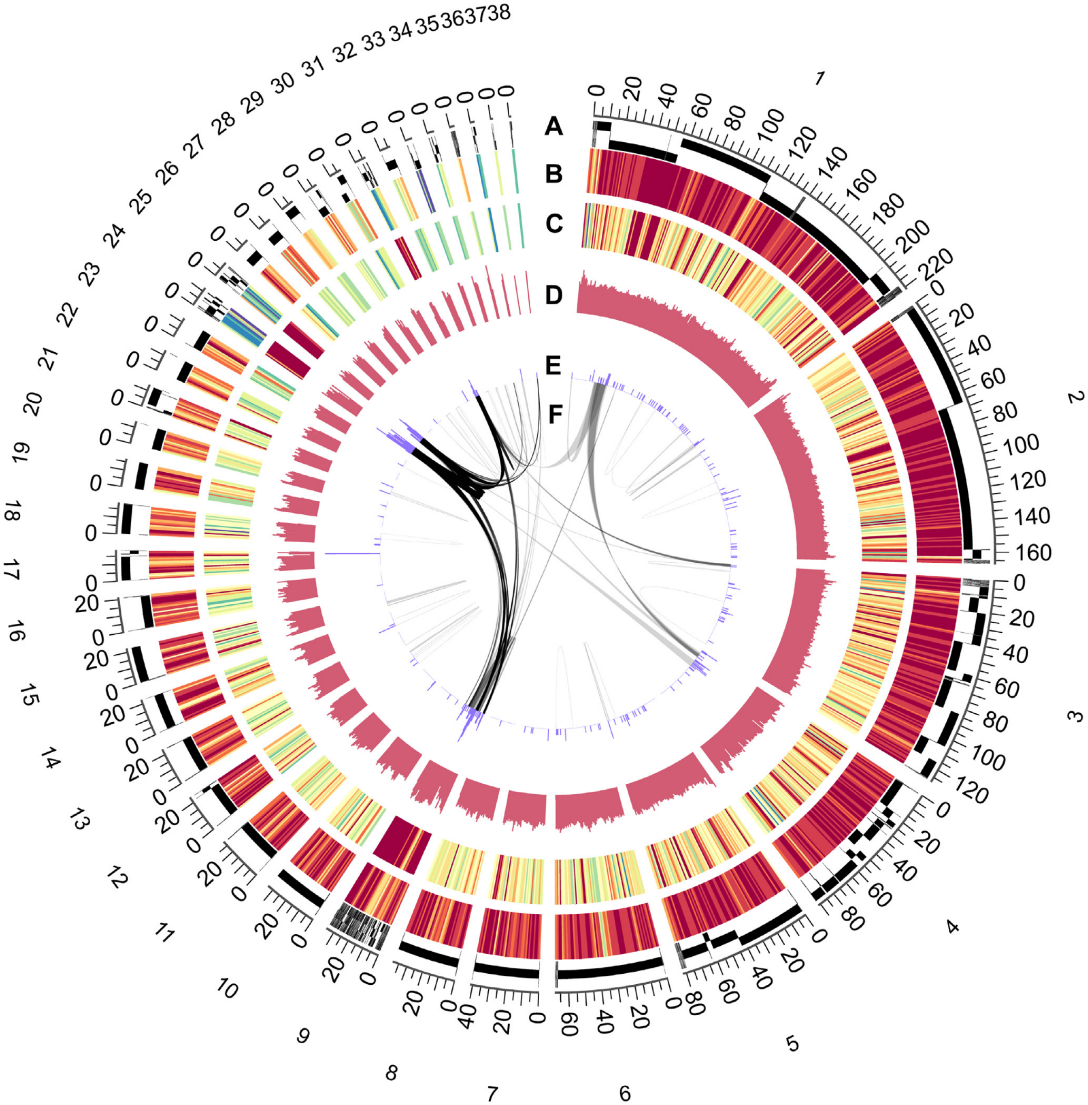
Distribution of genomic features. Concentric circle diagram presents the distribution of genomic features of Lion-head goose using nonoverlapping sliding windows with sizes of 1 Mb (from outmost to innermost). (A) The assembled pseudo-chromosome and the corresponding position. (B) Gene density calculated on the basis of the number of genes. (C) Average expression level of overall 36 samples. Eight tissues (i.e., brain, pharyngeal pouch, head sarcoma, spleen, liver, chest muscle, kidney, and heart) and blood were collected from 4 healthy adult animals. (D) GC content. (E) Density of TE. (F) Gene synteny and collinearity analysis.

The Lion-head goose is a famous local variety in China and one of the most giant goose breeds worldwide, with a unique appearance and social benefits. Here, we attempt to construct a highly continuous chromosome-scale genome of an adult purebred male Lion-head goose with a high degree of homozygosity to minimize heterozygous alleles. The following sequencing and genome assemble strategies were applied: Illumina sequencing, PacBio SMRT sequencing, BioNano optical mapping, and Hi-C approach (Supplementary Table S1). We assemble these data step by step and generate a progressively improved assembled genome (Supplementary Fig. S1). A total of 185.37 Gb of high-quality PacBio long reads were generated, representing a ∼168× depth of the estimated 1.05-Gb genome with heterozygosity of 0.335% based on the *k*-mer analysis of the Illumina sequences (Supplementary Fig. S1, Supplementary Table S2). Combing the *de novo* assembly of the Illumina and PacBio sequences resulted in a draft genome of 1.20 Gb, yielding 1,859 contigs with a length of 13.7 Mb for contig N50 and 57.6 Mb for the longest (Table 1). Furthermore, with the help of BioNano optical mapping, the scaffold N50 value was increased to 37 Mb. To obtain a chromosome-scale assembly, a set of ∼230 Gb Hi-C data was used to orient, order, phase, and anchor the contigs. Approximately 97.27% of the reads assembled were anchored to 40 high-confidence pseudo-chromosomes (39 autosomes and Z chromosome) using the high-density genetic map (Supplementary Fig. S1, Fig. 2). After polishing, we finally assembled the ultimate genome into 1.19 Gb with the final contig N50 of 20.59 Mb and scaffold N50 of 25.8 Mb, with a GC content of 42.39% (Supplementary Tables S2 and S3). The structure and quality of the assembled genome were determined by mapping a Hi-C chromosomal contact map.

**Table 1:** Summary of repeat classification

Type	Length	Percent
Long interspersed nuclear element	76,437,757	5.98
Simple sequence repeats	23,026,311	1.80
Low complexity	4,663,288	0.36
Tandem repeats	52,426,380	4.10
Total	156,553,736	12.25

The completeness of the Lion-head goose genome assembly was assessed using the BUSCO gene set. The result showed that almost 99.02% of the reads were correctly mapped to the genome. We then evaluated the assembled genome with 95.21% single-copy and 1.70% duplicated orthologs from the BUSCO dataset, confirming that 8,081 genes (96.92%) were intact in this genome. These results indicate the high reliability and integrity of the assembled genome (Supplementary Fig. S2 and Table S4).

### Genome annotation

To support the genome annotation, we conducted RNA-seq analysis using RNA samples of blood and 8 tissues (brain, pharyngeal pouch, head sarcoma, spleen, liver, chest muscle, kidney, and heart) from 4 healthy adult individuals. The aggregate of 760 Gb raw reads was accumulated by the paired-end sequencing of the 36 constructed libraries. After filtering the adapter and low-quality sequences, 723 Gb qualified Illumina reads remained and were *de novo* assembled into unique transcripts (unigenes). Overall, a total of 216,229 unigenes were assembled, and at the level N50, 5,082 nucleotides were obtained. A total of 21,208 protein-coding gene annotations were predicted in Lion-head goose by combining *de novo* prediction, homologous protein prediction, and transcription alignment. After filtering TE-related genes, a total of 21,010 protein-coding gene annotations were finally obtained by the TE seeker database (Fig. 2). Furthermore, a total of 8.15% repeat sequence and 4.10% tandem repeats of the genome were detected (Table 1). Comparative statistics of genome quality metrics with the assembled goose genome (including Zhedong white goose, Sichuan white goose, and Tianfu goose) are shown in Table 2.

**Table 2:** Comparison of the present study with previous quality metrics of goose genome assembly

Genomic features	Lion-head goose	Zhedong white goose	Sichuan white goose	Tianfu goose
Estimate of genome size (bp)	1,278,045,811	1,208,661,181	1,198,802,839	1,277,099,016
Total length of contigs (bp)	1,268,074,106	1,086,838,604	1,100,859,441	1,113,842,245
Total length of scaffolds (bp)	1,277,289,474	1,122,178,121	1,130,663,797	1,113,913,845
Number of contigs	1,318	60,979	53,336	2,771
Number of scaffolds	1,266	1,050	1,837	2,055
Contig N50 (bp)	21,589,146	27,602	35,032	1,849,874
Scaffold N50 (bp)	27,064,542	5,202,740	5,103,766	33,116,532
Longest contig (bp)	91,420,268	201,281	399,111	10,766,871
Longest scaffold (bp)	98,160,899	24,051,356	20,207,557	70,896,740
GC content	42.39%	38.00%	41.68%	42.15%
No. of predicted protein-coding genes	21,010	16,150	16,288	17,568
Percentage of repeat sequences	12.25%	6.33%	6.90%	8.67%

### Phylogenetic analysis

**Figure 3:**
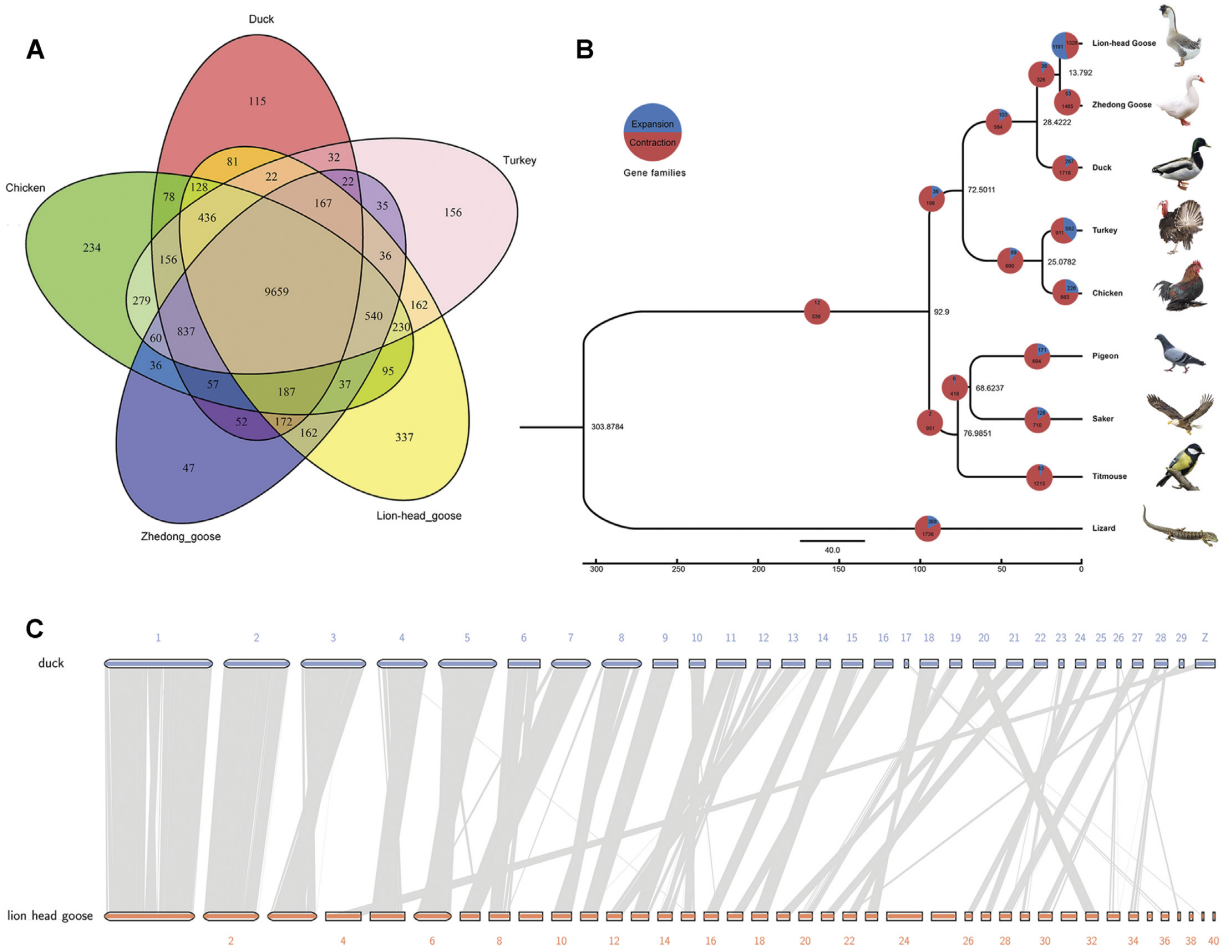
Phylogenetic relationship and comparative genomics analyses. (A) Venn diagram showing the orthologous gene families shared among the genomes of Lion-head goose, Zhedong white goose, chicken, duck, and turkey. (B) Phylogenetic tree with the divergence times and history of orthologous gene families. Numbers on the nodes represent divergence times. The numbers of gene families that expanded (green) or contracted (red) in each lineage after speciation are shown in the circles of the corresponding branch. (C) Gene comparison of homologous chromosomes between Lion-head goose and duck. Gray lines indicate collinearity between the genomes.

To investigate the genomic evolution of poultry, we compared the sequences of 8 bird species (Lion-head goose, Zhedong white goose, duck, turkey, chicken, pigeon, saker, and titmouse) and green lizard, clustering the genes into 15,162 gene families (Fig. 3A, Supplementary Table S5). Among these, 6,422 single-copy gene families were identified and used to construct a phylogenetic tree (Fig. 3B). This revealed that the geese and ducks were clustered into a subclade that probably evolved from a common ancestor approximately 28.42 Mya. As expected, the Lion-head goose displayed a close relationship with the Zhedong white goose. The divergence time between the Lion-head goose and Zhedong white goose was estimated to be 13.79 Mya, and that between chicken and turkey was nearly 25.07 Mya. The above results confirmed the reliability of the tree.

**Figure 4:**
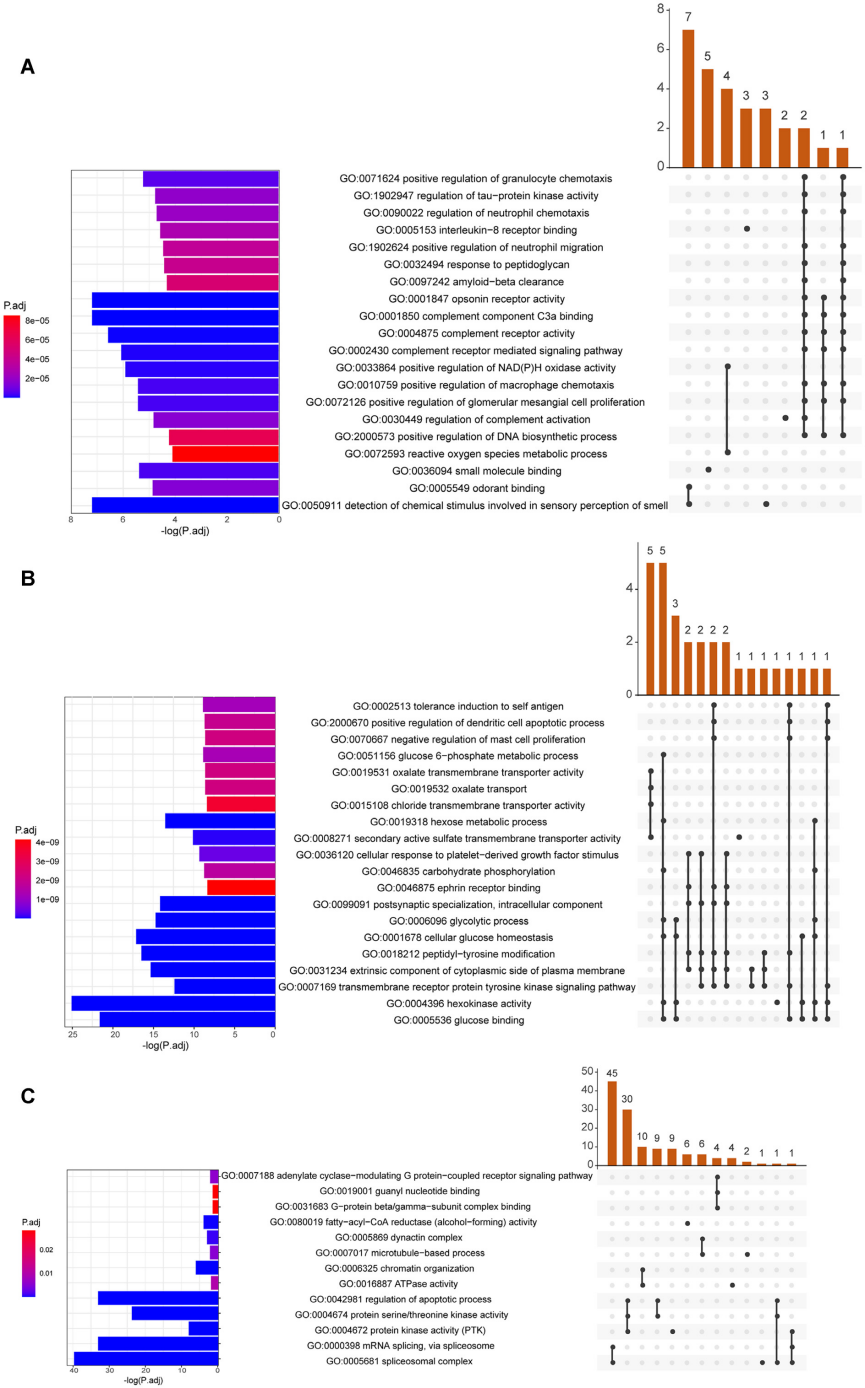
GO enrichment analysis of gene families. (A) Expanded and (B) contracted gene families from *Anatidae* varieties (duck, Zhedong white goose, Lion-head goose). (C) Unique gene families from the Lion-head goose. The bar graph on the left represents the *P*-adjust gradient of GO terms, and the color corresponds to the number on the x-axis (i.e., −log (P.adj)). The bluer the color is, the smaller the *P*-adjust is, and the more significant it is. The redder the color is, the larger the *P*-adjust is, and the less significant it is. The upper right bar chart exhibits that several genes act together on the terms below. The lower right chart displays the intersection of the genes of each term; the dots connected by lines represent the intersection of multiple terms; the black dots represent “yes,” and the gray dots represent “no.”

Of all the gene families in the Lion-head goose, 4,233 gene families were significantly expanded and 324 were contracted. Compared with Zhedong white goose, the Lion-head goose had more gene families, and there are also more events of gene family expansion and contraction. Moreover, we mixed the gene family sets of several *Anatidae* varieties (duck, Zhedong white goose, Lion-head goose), and performed expansion and contraction analysis and corresponding GO enrichment analysis. In this task, the GO analysis of expanded gene families suggested olfactory perception, such as detection of chemical stimulus involved in sensory perception of smell (GO:0050911, *P* = 6.97 × 10^−8^), and odorant binding (GO:0005549, *P* = 1.47 × 10^−5^), both of which may be related to the adaptation of the species to find food in water (Fig. 4A, Supplementary Table S6). Meanwhile, contracted gene families were concentrated in the areas of glucose synthesis and metabolism, such as hexokinase activity (GO:0004396, *P* = 7.64 × 10^−26^), glucose binding (GO:0005536, *P* = 2.30 × 10^−22^), cellular glucose homeostasis (GO:0001678, *P* = 6.84 × 10^−18^), glycolytic process (GO:0 006 096, *p* = 1.75×10^−15^), hexose metabolic process (GO:0 019 318, *p* = 2.66×10^−14^), carbohydrate phosphorylation (GO:0 046 835, *p* = 1.68×10^−9^), and glucose 6-phosphate metabolic process (GO:0051156, *P* = 1.27 × 10^−9^), which may be closely related to characteristics of glycogen storage and utilization during migration (Fig. 4B, Supplementary Table S7). Besides, 220 unique gene families (other species lack these gene families) of the Lion-head goose were identified and functionally annotated in GO categories, such as protein kinase activity (GO:0004672, *P* = 6.85 × 10^−9^), the regulation of apoptotic process (GO:0042981, *P* = 5.78 × 10^−34^), the adenylate cyclase–modulating G protein–coupled receptor signaling pathway (GO:0 007 188, *p* = 5.92×10^−3^), and fatty-acyl-CoA reductase (alcohol-forming) activity (GO:0080019, *P* = 8.94 × 10^−5^, Fig. 4C, Supplementary Table S8). Interestingly, we annotated a reproduction-related protein in the species-specific gene family, *Sterile* (Pfam ID: PF03015), acting on fatty-acyl-CoA reductase (alcohol-forming) activity, which may be related to the low reproductive rate caused by congenital infertility in geese.

Collinearity analysis allows one to judge molecular evolutionary events between species and explain the structural differences between the 2 genomes. We identified synteny blocks among avian genomes and found high collinearity between our assembly and the duck genome (genome size = 1.19 Gb). Here, multiple chromosomes (Chr 1–5, 10, 12, 15, 17–20, 23, 26, 27, 29, 30, 32, 34, 36, 37, 39) of Lion-head goose were almost one-to-one collinear with those of the duck, but some chromosomal rearrangements occurred (Fig. 3C, Supplementary Fig. S3). For example, on some chromosomes like Chr 1, 2, 3, and 4 of the duck genome, genes break and rearrange on the Lion-head goose genome, resulting in sequential inversion. In addition, some scaffolds, such as Chr 9, 24, 25, 31, 35, 38, and 40, were not correlated with any chromosome of the duck genome, which may be due to the different sources of genes on the chromosome. These results indicate that chromosome inversion and interchromosomal recombination may have occurred specifically in Lion-head goose during the evolutionary process, but this requires further investigation and verification. Moreover, Chr 4 of Lion-head goose was found to correspond to the sex chromosome Z of duck, except for the inversions of small patches of segments; therefore, we inferred that Chr 4 was the sex chromosome of the Lion-head goose. This information will be fundamental for comparative genomic studies in *Anatidae* animals.

### Cluster analysis of different goose species population

**Figure 5:**
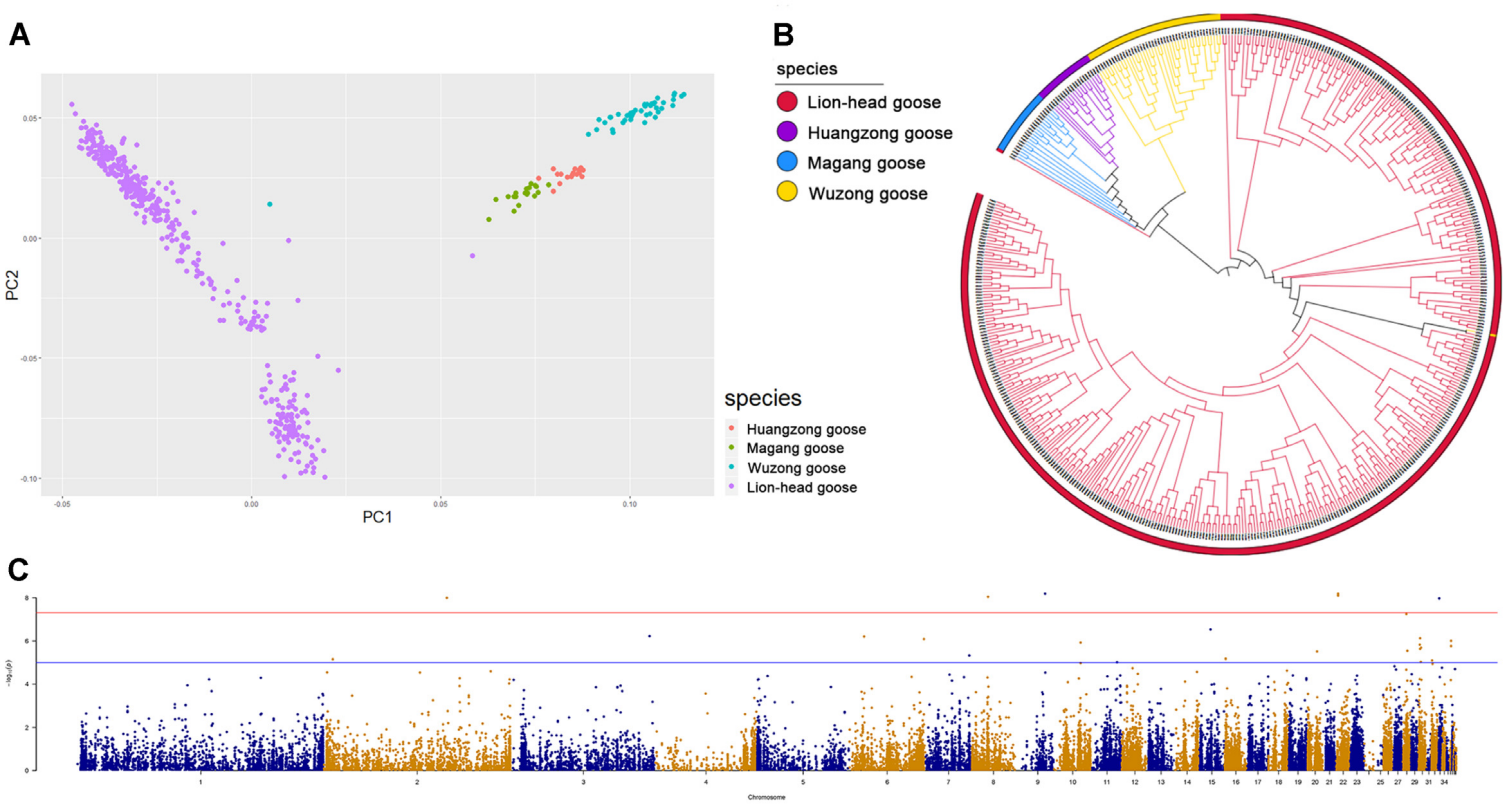
Comparison of different goose species and genome-wide association analysis of body weight. (A) Principal component analysis of sample structures using first 2 principal components. (B) The phylogenetic trees of several goose species. (C) Manhattan plot of genome-wide association analysis for body weight. The x-axis indicates chromosomes, and the y-axis indicates the *P* values of the SNP markers. The red solid line indicates the threshold *P* value for genome-wide significance. The blue solid line indicates the threshold *P* value for the significance of potential association.

Blood samples were collected from 514 geese (including Lion-head goose, Wuzong goose, Huangzong goose, and Magang goose), and their weight was recorded, with the Lion-head goose using the minimum weight, the Wuzong goose using the maximum weight, and the Huangzong goose and Magang goose using the average weight. That is, the Lion-head goose weighed at least 9 kg, the Wuzong goose weighed at most 2.5 kg, the Huangzong goose weighed about 3–4 kg, and the Magang goose weighed 4.8–5.5 kg (Table 3). Blood from each sample was used for paired-end 100 resequencing. The average raw data was 1,520.60 Mb, the average sequencing depth was 12.05×, the average coverage was 7.56%, the average matching rate was 91.31%, and 44,858 SNP loci were retained for subsequent analysis after screening SNPs with minimum allele frequency <5%, Hardy–Weinberg equilibrium test significance threshold of 10^−7^, and maximum deletion rate threshold of 0.7. We reconstructed the goose population structure using SNP data, revealing 4 distinct subpopulations. The PCA results demonstrated that the Lion-head Goose population was clearly distinguishable from the Magang goose, Wuzong goose, and Huangzong goose, and there was a clear differentiation within the species (Fig. 5A). The clustering of Magang goose and Huangzong goose was closer together, probably related to their closer geographical location and the existence of some genetic exchange. The phylogenetic tree results were consistent with the PCA results. The clustering of Magang goose and Huangzong goose was closer to each other, and they clustered into one branch with Wuzong goose (Fig. 5B).

**Table 3:** Descriptive statistical of body weight traits

Species	Number	Maximum (kg)	Minimum (kg)	Mean ± SEM
Lion-head goose	416	15.70	9.00	13.55 ± 1.97
Magang goose	20	5.50	4.80	5.32 ± 0.36
Huangzong goose	20	4.30	2.70	3.40 ± 0.83
Wuzong goose	44	2.50	1.80	2.24 ± 0.25

### Candidate genomic regions for body weight based on combined analyses of GWAS and selective-sweep

The Lion-head goose, Huangzong goose, Magang goose, and Wuzong goose are all local species in Guangdong, but they differ greatly in body weight. In this study, we sought to reveal genomic changes associated with body weight in the 4 goose species and screen genomic regions and genes. Selective sweep analysis was performed based on the F_ST_ index, considering the top 5% window as candidate regions. In total, 979 selective regions containing 818 genes were detected.

**Figure 6:**
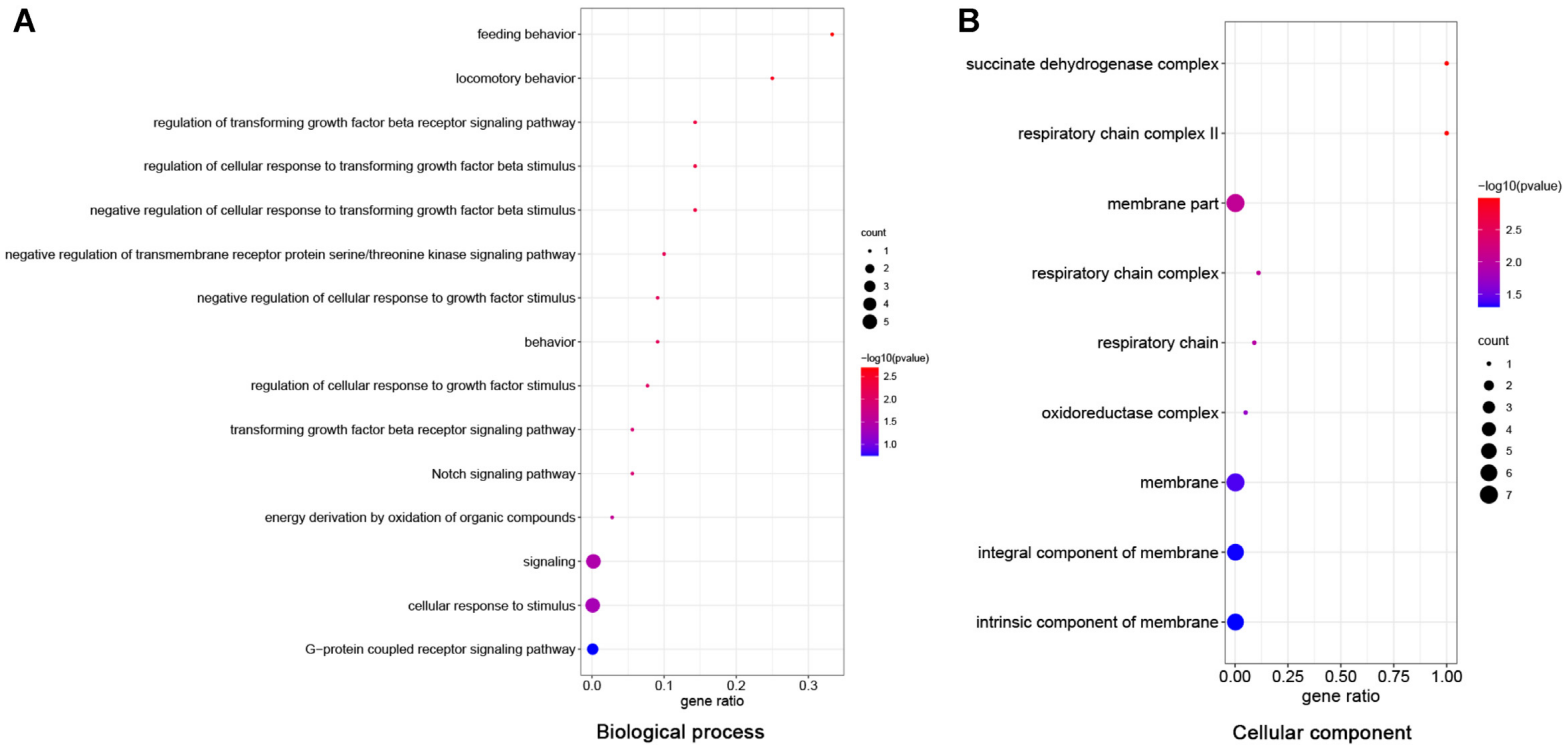
GO analysis of body weight-related genes: (A) biological processes level and (B) cellular component level.

We then combined the genome-wide association study (GWAS) results with the detected selective features to screen for candidate genomic regions responsible for the differences in goose weight. From the Manhattan plot (Fig. 5C), a total of 10 significant signals were found to be associated with body weight trait in geese at the genome-wide level, including 1 significant SNP detected on Chr 2, 8, 9, and 33, respectively (−log (*P*) > 7.30), and 6 significant SNPs annotated by 2 genes on Chr 22, with the closest Manhattan plot SNP peak on Chr 9 for the gene *OR* (olfactory receptor). Six significant SNPs on Chr 22 were located between 1,992,485 and 1,992,520 bp, a region that spans only a physical distance of 35 bp but contains 6 SNP loci, making it necessary to analyze these SNPs in this small region in detail to determine whether multiple QTL (Quantitative trait locus) are involved. The most significant SNP in this region could explain about 8.19% of the phenotypic variation. Apart from significant SNPs, potentially significant QTLs were detected on many chromosomes (including Chr 2, 3, 6, 7, 10, 11, 15, 16, 20, 28, 30, 32, 36), with a total of 25 implied significant SNPs (4.90 < −log (*P*) <7.30). On Chr 30, the suggestively significant SNPs were located between 1,258,517 and 2,422,666 bp, spanning approximately 1.16 Mb, with the most significant SNPs in this region explaining approximately 6.12% of the phenotypic variation (Table 4). In the present study, we identified genes in the region near the significant SNPs, annotating a total of 21 genes. These genes may be important in mediating growth and development, and we inference that the *LDLRAD4* gene may play a key role in developmental plasticity in geese, while the *GPR180* gene may regulate the locomotor behavior of geese to make them stronger (Fig. 6). GWAS peaks overlapped with genomic regions with selective features on some chromosomes (Supplementary Data). This suggests that the region carrying QTL are not only associated with body weight in GWAS but also under selection during domestication.

**Table 4:** Genome-wide association analysis of body weight in geese

Chr	Allele	Physical position	Regression coefficient	*P* value	Genes
2	A	108,496,954	−0.1886	1.01E-08	*LDLRAD4*
2	G	7,706,165	0.2612	6.98E-06	*LDLRAD4*
3	T	123,032,780	−0.3979	6.03E-07	*EGF, KBTBD*
6	A	13,264,157	−0.24	6.28E-07	*TSPAN*
6	T	66,027,192	0.2127	8.14E-07	*IGFN1*
7	T	39,117,443	−0.3131	4.66E-06	―
8	T	14,712,470	0.1865	8.97E-09	*PPEF1*
9	T	26,883,582	−2.7E+12	0	*OR*
10	C	23,997,415	−0.3032	1.19E-06	―
10	C	23,997,399	−0.2542	1.05E-05	―
10	T	23,997,401	−0.2542	1.05E-05	―
11	A	22,838,749	0.1548	9.55E-06	―
15	T	10,257,386	0.2527	2.96E-07	*GPR180, GPCPD1*
16	A	1,477,673	−0.1892	6.53E-06	―
16	G	1,477,679	−0.1891	6.78E-06	―
20	A	8,531,879	0.151	3.05E-06	―
22	A	1,992,485	−0.3972	6.51E-09	*GALNT, AUTS2*
22	A	1,992,518	−0.3973	7.69E-09	*GALNT, AUTS2*
22	G	1,992,501	−0.3974	7.94E-09	*GALNT, AUTS2*
22	C	1,992,505	−0.3974	7.94E-09	*GALNT, AUTS2*
22	C	1,992,507	−0.3974	7.94E-09	*GALNT, AUTS2*
22	G	1,992,515	−0.3974	7.94E-09	*GALNT, AUTS2*
28	C	3,587,271	0.2936	5.81E-08	*PPP1R15B, FGD2*
28	G	4,472,051	−0.2359	2.82E-06	*PPP1R15B, FGD2*
30	C	1,652,158	−0.3469	7.53E-07	*SH2*
30	T	1,258,517	0.2205	1.48E-06	*SH2*
30	G	2,422,665	0.1894	2.04E-06	*SH2*
30	T	2,422,666	0.1894	2.04E-06	*SH2*
30	A	1,652,207	−0.3289	2.3E-06	*SH2*
30	T	2,269,897	0.211	9.22E-06	*SH2*
32	G	655,318	0.2599	7.95E-06	―
33	A	975,487	0.2567	1.07E-08	*SDHA*
36	A	1,523,127	−0.3274	9.86E-07	*SPRY*
36	G	1,523,132	−0.3216	1.7E-06	*SPRY*
36	C	1,523,105	−0.3291	1.72E-06	*SPRY*

## Discussion

Despite the importance of the genus *Anser*, an economically important animal, the relative scarcity of genomic resources has largely hindered progress in studying genome evolution and molecular breeding in the major animals. High-quality chromosome-level genomes can provide key resources for studying. This study describes a chromosome-scale assembly of Lion-head goose obtained by a combination of data from the Illumina, SMRT, BioNano, and Hi-C platforms. The genome assembly is 1.19 Gb in length, and more than 97.27% of the assembled genome is anchored on 40 pseudo-chromosomes. The BUSCO assessment revealed 99.02% complete genes in the assembled genome, making it a better-continuity and higher-quality genome assembly than the recently published Tianfu goose genome with a contig N50 of 1.85 Mb and scaffold N50 of 33.12 Mb [39]. Compared with the cultivated breed Tianfu goose, Lion-head goose, a traditional native breed, should occupy a more prominent position in the germplasm resources, and its evolving message can provide a reference for other local breeds that is worthy of in-depth study.

Comparative genomics is the analysis of the structural characteristics of multiple individual genomes of a species or genomes of multiple species to find out the similarities and differences of gene sequences of species with the help of bioinformatics and then to study the gene family analysis, analyze the differentiation and evolution of species, and provide a basis for elucidating species evolution. In this study, the evolutionary events of the Lion-head goose were analyzed by comparing the genome sequences with those of other birds. The results showed that the Lion-head goose and Zhedong white goose were most closely related, diverging at about 13.8 Mya, while the geese and ducks diverged at 28.4 Mya. The results were similar to those of Zhedong white goose, Sichuan white goose, and Tianfu goose, indicating the accuracy of the assembly result of this study. Comparative genomic analysis revealed the genetic basis of interesting characters, which helped elucidate important biological implications and obtain solutions for genomic evolution between Lion-head geese and other species of *Anatidae* family, facilitating future genetic breeding programs. To our knowledge, this is the first chromosomal-level reference genome of Lion-head goose, providing important genomic data for the study of the family *Anatidae*.

The genomic information of the species population was obtained by whole-genome resequencing, and a large amount of variation information was obtained by comparison with the reference genome. Based on the correlation between differences in variation information and phenotypic differences of individuals, the adaptation of species to the environment, scanning of variant loci associated with important traits at the genome level, and localization of genetic mutations were discussed. Lion-head goose, Magang goose, Huangzong goose, and Wuzong goose are the main breeds of geese in Guangdong Province. Although they all belong to Guangdong Province, the body weight of adult geese varies greatly, and the molecular mechanism causing the huge difference is still unclear. In this study, 4 goose species were resequenced and examined for variation. PCA and phylogenetic tree analysis revealed significant differences among several goose species, indicating the feasibility of this study. Subsequently, GWAS was used to identify the candidate functional SNPs that might cause the weight difference of the 4 goose species, and genes such as *LDLRAD4, GPR180*, and *OR* were analyzed, annotated, and attributed to play an important role in mediating growth and development. Recently, there have been several studies related to agricultural traits that have achieved success in animal GWAS projects—for example, GWAS for improving reproductive performance and egg quality in geese and *TMEM161A* gene for embryo development [40]. Genome-wide association analysis of the early lactation milk fat content in 3,513 Fleckvieh bulls and 2,327 Holstein bulls detected 6 associated QTL regions, 2 of which were located near the gene *DGAT1* [41]. GWAS was conducted on 225 ducks with different-sized black spots, and the results showed that *EDNRB2* was the gene responsible for the variation in duck body surface spot size [42]. In this study, *LDLRAD4* (low-density lipoprotein receptor class A domain containing 4), *OR* (olfactory receptor), and *GPR180* (G protein–coupled receptor 180) were mainly found to function in body weight traits. Knockdown of *LDLRAD4* enhances transforming growth factor β (TGF-β)−induced cell migration, which in turn regulates cell growth, differentiation, motility, apoptosis, and matrix protein production [43]. The olfactory receptor (*OR2AT4*) has been shown to stimulate the proliferation of keratin-forming cells in peripheral human tissues [44]. *GPR180*, a component of the TGF-β signaling pathway, also has metabolic relevance in the body and may play an essential role in regulating adipose tissue and systemic energy metabolism [45]. Here we found some correlation between these genes and the TGF-β signaling; presumably, this pathway also acts on body weight. Identifying molecular genetic markers and the main effect QTL associated with critical agricultural traits is of great interest to breeders. Nevertheless, the candidate genes identified in this study were only detected by sequencing data and not experimentally validated. The functions of these candidate SNPs and gene markers need to be further verified by experimental results or other techniques. Thus, the findings in our GWAS study represent a valuable resource for geese and provide a new opportunity and basis for geneticists and breeders to work together to explore the genetics behind various agricultural traits.

## Conclusions

In summary, we have obtained a high-quality chromosome-scale draft assembly of a purebred Lion-head goose, which provides a genetic basis for understanding the acquisition of related traits and facilitates advances in goose genomics and genetic improvement. Moreover, the candidate genes and their variants identified in this study will help clarify our understanding of goose selective breeding and the development of new breeds. The obtained genome sequence of Lion-head goose is a vital addition to the genome of genus *Anser* and is valuable for further understanding goose molecular breeding strategies. This genomic resource is also of high value for evolutionary studies of closely related species.

## Supplementary Material

giad003_GIGA-D-22-00016_Original_Submission

giad003_GIGA-D-22-00016_Revision_1

giad003_GIGA-D-22-00016_Revision_2

giad003_GIGA-D-22-00016_Revision_3

giad003_Response_to_Reviewer_Comments_Original_Submission

giad003_Response_to_Reviewer_Comments_Revision_1

giad003_Response_to_Reviewer_Comments_Revision_2

giad003_Reviewer_1_Report_Original_Submission Guangliang Gao bang Luo -- 4/1/2022 Reviewed

giad003_Reviewer_1_Report_Revision_1 Guangliang Gao -- 7/18/2022 Reviewed

giad003_Reviewer_2_Report_Original_Submission Shuo Feng -- 4/7/2022 Reviewed

giad003_Reviewer_3_Report_Original_Submission Filippo Biscarini -- 4/7/2022 Reviewed

giad003_Reviewer_3_Report_Revision_1 Filippo Biscarini -- 8/8/2022 Reviewed

giad003_Reviewer_3_Report_Revision_2 Filippo Biscarini -- 9/16/2022 Reviewed

giad003_Supplemental_File

## Data Availability

The final genome assembly data supporting the results of this article are available in the NCBI BioProject repository (accession number: PRJNA736831). The RNA assembly data are available in the NCBI BioProject repository (accession number: PRJNA807796). The raw resequencing genome data supporting the GWAS study are available in the NCBI BioProject repository (accession numbers: PRJNA552198, PRJNA552383, and PRJNA552384). All supporting data are available in the *GigaScience* GigaDB database [46].

## Additional Files

**Supplementary Fig. S1**. Sequencing process and presentation.

**Supplementary Fig. S2**. BUSCO assessment of the assembly genome of Lion-head goose.

**Supplementary Fig. S3**. Gene synteny between the Lion-head goose and duck genomes.

**Supplementary Table S1**. Statistics of sequenced clean data.

**Supplementary Table S2**. Statistics of genome survey.

**Supplementary Table S3**. Statistics of genome assembly quality.

**Supplementary Table S4**. Summary of BUSCO genome evaluation.

**Supplementary Table S5**. Summary of gene families from several species.

**Supplementary Table S6**. GO annotation of expanded gene families from *Anatidae* varieties (duck, Zhedong white goose, Lion-head goose; top 20).

**Supplementary Table S7**. GO annotation of contraction gene families from *Anatidae* varieties (duck, Zhedong white goose, Lion-head goose; top 20).

**Supplementary Table S8**. GO annotation of unique gene families from the Lion-head goose.

**Supplementary Data**. Significant information of selective-sweep analysis.

## Abbreviations

BLAST: Basic Local Alignment Search Tool; BUSCO: Benchmarking Universal Single-Copy Orthologs; Chr: chromosome; GATK4: Genome Analysis Toolkit 4; Gb: gigabase pairs; GO: gene ontology; GPR180: G protein–coupled receptor 180; GWAS: genome-wide association study; HERA: Highly Efficient Repeat Assembly; Hi-C: high-throughput chromosome conformation capture; Kb: kilobase pairs; kg: kilogram; LDLRAD4: low-density lipoprotein receptor class A domain containing 4; LTR: long terminal repeat; Mb: megabase pairs; Mya: million years ago; NCBI: National Center for Biotechnology Information; OR: olfactory receptor; OR2AT4: olfactory receptor family 2 subfamily AT member 4; PacBio: Pacific Biosciences; PCA: principal component analysis; QTL: quantitative trait locus; RAxML: Randomized Axelerated Maximum Likelihood; RNA-seq: RNA sequencing; SMRT: single molecule real time; SNP: single-nucleotide polymorphism; STAR: Spliced Transcripts Alignment to a Reference; TE: transposable element; TGF: transforming growth factor; TMEM161A: transmembrane protein 161A.
