# Supplementary material for: Chromosome-level genome assembly of goose provides insight into the adaptation and growth of local goose breeds
Source: Gigascience. 2023 Feb 3;12:giad003. doi: 10.1093/gigascience/giad003 (PMC9896136; doi:10.1093/gigascience/giad003)
Supplement: giad003_GIGA-D-22-00016_Original_Submission [file giad003_giga-d-22-00016_original_submission.pdf]

GigaScience

Chromosome-level genome assembly of goose provides insight into the adaptation and growth of local goose breeds

--Manuscript Draft--

**Manuscript Number:**	GIGA-D-22-00016
**Full Title:**	Chromosome-level genome assembly of goose provides insight into the adaptation and growth of local goose breeds
**Article Type:**	Research
**Funding Information:**	Key Research and Development Program of Guangdong Province (2020B020222001) — Not applicable<br>Construction of Modern Agricultural Science and Technology Innovation Alliance in Guangdong Province (2021KJ128, 2020KJ128) — Not applicable<br>National Modern Agricultural Industry Science and Technology Innovation Center in Guangzhou (2018kczx01) — Not applicable<br>Guangdong Provincial Promotion Project on Preservation and Utilization of Local Breed of Livestock and Poultry (4000-F18260) — Not applicable<br>Guangdong Basic and Applied Basic Research Foundation (2019A1515012006) — Not applicable
**Abstract:**	Backgrouond: Anatidae contains numerous waterfowl species with great economic value, but the genetic diversity basis remains insufficiently investigated. Here, we report a chromosome-level genome assembly of Lion-head goose (Anser cygnoides), a native breed in South China, through the combination of PacBio, Bionano and Hi-C technologies. Findings: The assembly had a total genome size of 1.19 Gb, consisting of 1,859 contigs with an N50 length of 20.59 Mb, generating 40 pseudochromosomes, representing 97.27% of the assembled genome, and identifying 21,208 protein-coding genes. Comparative genomic analysis revealed that geese and ducks diverged approximately 28.42 million years ago, and geese have undergone massive gene family expansion and contraction. To identify genetic markers associated with body weight in different geese breeds including Wuzong goose, Huangzong goose, Magang goose and Lion-head goose, a genome-wide association study was performed, yielding an average of 1,520.6 Mb of raw data with detecting 44,858 SNPs. GWAS showed that six SNPs were significantly associated with body weight and 25 were potentially associated. The significantly associated SNPs were annotated as LDLRAD4 , GPR180 , OR , enriching in growth factor receptors regulation pathways. Conclusions: We present the first chromosome-level assembly of the Lion-head goose genome, which will expand the genomic resources of the Anatidae family, providing a basis for adaptation and evolution. Candidate genes significantly associated with different goose breeds may serve to understand the underlying mechanisms of weight differences.
**Corresponding Author:**	Xinheng Zhang<br>South China Agricultural University<br>Guangzhou, Guangdong CHINA
**Corresponding Author Secondary Information:**	
**Corresponding Author's Institution:**	South China Agricultural University
**Corresponding Author's Secondary Institution:**	
**First Author:**	Qiqi Zhao
**First Author Secondary Information:**	

Powered by Editorial Manager® and ProduXion Manager® from Aries Systems Corporation

Order of Authors:	Qiqi Zhao
	Junpeng Chen
	Zi Xie
	Jun Wang
	Keyu Feng
	Wencheng Lin
	Hongxin Li
	Zezhong Hu
	Weiguo Chen
	Feng Chen
	Muhammad Junaid
	Huanmin Zhang
	Zhenping Lin
	Qingmei Xie
	Xinheng Zhang
Order of Authors Secondary Information:	
Additional Information:	
**Question**	**Response**
Are you submitting this manuscript to a special series or article collection?	No
**Experimental design and statistics**<br><br>Full details of the experimental design and statistical methods used should be given in the Methods section, as detailed in our Minimum Standards Reporting Checklist. Information essential to interpreting the data presented should be made available in the figure legends.<br><br>Have you included all the information requested in your manuscript?	Yes
**Resources**<br><br>A description of all resources used, including antibodies, cell lines, animals and software tools, with enough information to allow them to be uniquely identified, should be included in the Methods section. Authors are strongly encouraged to cite Research Resource	Yes

Powered by Editorial Manager® and ProduXion Manager® from Aries Systems Corporation

Identifiers (RRIDs) for antibodies, model organisms and tools, where possible.<br><br>Have you included the information requested as detailed in our Minimum Standards Reporting Checklist?	
**Availability of data and materials**<br><br>All datasets and code on which the conclusions of the paper rely must be either included in your submission or deposited in publicly available repositories (where available and ethically appropriate), referencing such data using a unique identifier in the references and in the "Availability of Data and Materials" section of your manuscript.<br><br>Have you have met the above requirement as detailed in our Minimum Standards Reporting Checklist?	Yes

Powered by Editorial Manager® and ProduXion Manager® from Aries Systems Corporation

Manuscript

Click here to access/download;Manuscript;renamed_4ae47.docx

Chromosome-level genome assembly of goose provides insight into the adaptation and growth of local goose breeds

Qiqi Zhao[1,3,5], Junpeng Chen[2], Zi Xie[1,3,5], Jun Wang[4], Keyu Feng[1,3,5], Wencheng Lin[1,3,5], Hongxin Li[1,3,5], Zezhong Hu[1], Weiguo Chen[1,3,5], Feng Chen[1,3], Muhammad Junaid[4], Huanmin Zhang[6], Zhenping Lin[2*], Qingmei Xie[1,3,5*], Xinheng Zhang[1,3,5*]

[1]Heyuan Branch, Guangdong Provincial Laboratory of Lingnan Modern Agricultural Science and technology & Guangdong Provincial Key Lab of Agro-Animal Genomics and Molecular Breeding, College of Animal Science, South China Agricultural University, Guangzhou, Guangdong 510642, China; [2]Shantou Baisha Research Institute of Original Species of Poultry and Stock, Shantou, Guangdong 515000, China; [3]Department of Science and Technology of Guangdong Province, Key Laboratory of Animal Health Aquaculture and Environmental Control, Guangzhou, Guangdong 510642, China; [4]College of Marine Sciences, South China Agricultural University, Guangzhou, Guangdong, 510642, China; [5]Guangdong Engineering Research Center for Vector Vaccine of Animal Virus, Guangzhou, 510642, China and [6]Avian Disease and Oncology Laboratory, Agriculture Research Service, United States Department of Agriculture, East Lansing, MI, 48823, USA

* Correspondence address:

Zhenping Lin, Shantou Baisha Research Institute of Original Species of Poultry and Stock, Shantou, China. E-mail: Linzp02@163.com; Qingmei Xie and Xinheng Zhang, College of Animal Science, South China Agricultural University, Guangzhou, China. E-mails: qmx@scau.edu.cn (Q.X.); xhzhang@scau.edu.cn (X.Z.)

running title: Goose chromosome-level Genome Assembly

Abstract

Backgrouond: *Anatidae* contains numerous waterfowl species with great economic value, but the genetic diversity basis remains insufficiently investigated. Here, we report a chromosome-level genome assembly of Lion-head goose (*Anser cygnoides*), a native breed in South China, through the combination of PacBio, Bionano and Hi-C technologies. **Findings:** The assembly had a total genome size of 1.19 Gb, consisting of 1,859 contigs with an N50 length of 20.59 Mb, generating 40 pseudochromosomes, representing 97.27% of the assembled genome, and identifying 21,208 protein-coding genes. Comparative genomic analysis revealed that geese and ducks diverged approximately 28.42 million years ago, and geese have undergone massive gene family expansion and contraction. To identify genetic markers associated with body weight in different geese breeds including Wuzong goose, Huangzong goose, Magang goose and Lion-head goose, a genome-wide association study was performed, yielding an average of 1,520.6 Mb of raw data with detecting 44,858 SNPs. GWAS showed that six SNPs were significantly associated with body weight and 25 were potentially associated. The significantly associated SNPs were annotated as *LDLRAD4*, *GPR180*, *OR*, enriching in growth factor receptors regulation pathways. **Conclusions:** We present the first chromosome-level assembly of the Lion-head goose genome, which will expand the genomic resources of the *Anatidae* family, providing a basis for adaptation and evolution. Candidate genes significantly associated with different goose breeds may serve to understand the underlying mechanisms of weight differences.

Keywords: Lion-head goose, Genome assembly, Comparative genome, Genome-wide association study

Introduction

The *Anatidae* is a family of the ancient *Aves* class with order *Anseriformes*, containing 43 genuses and 174 species, including the majority of birds, such as ducks, geese, swans, and is the most prominent family of wandering birds [1]. Physical characteristics and features vary significantly among species, making the *Anatidae* family rich in diversity and specificity. *Anatidae* adults are usually herbivores, feeding on a variety of aquatic plants, which are well suited to sustainable production practices thereby reducing competition for human food; and some species are even used for crop weeds and pests control [1, 2]. For a long time, duck and goose feathers have been popular in pillows, quilts and coats [3]. Several species in the genus *Anser* are commercially important and domesticated as poultry because of their unique warmth retention properties and meat-producing performance. According to archaeological evidence, geese were domesticated around 6,000 years ago near the Mediterranean Sea, and later spread around the world due to human activities [4]. It is widely believed that *Anser cygnoides* is the ancestor of the Chinese goose (*Anser cygnoides domesticus*) with a domestication history of more than 3,000 years [1]. After artificial domestication, the domestic goose has increased its cold tolerance and roughage-resistance, but its wings are degraded and weakened in flight, unable to travel long distances [1]. Egg-laying rate and goslings survival rate are also improved compared to wild swans, and the lifespan is longer [5]. Furthermore, overfeeding can cause foie gras to be at least three-fold larger than the normal size while the goose remains healthy, making the goose a good model to study human liver steatosis [6]. Chinese domestic geese, a natural gene pool, contain a variety of native breeds with diverse phenotypes [7]. One interesting phenomenon we found here is that the weight of adult domestic geese varies greatly in the same region, for example, the Lion-head goose in Shantou (116°14'-117°19' E, 23°02'-23°38' N), Guangdong Province, can weigh more than 9 kg, while the Wuzong goose in Qingyuan (111°55'-113°55' E, 23°31'-25°12' N), Guangdong Province, the average weight is only about 3 kg [8, 9]. However, the mechanisms for such differences have not been clarified, let alone being resolved at the genomic level. Therefore, a complete, continuous and accurate reference genome is essential, for deciphering genomic diversity, evolutionary and adaptive processes, and the industry's

development.

High-quality genome assembly sequences enable us to comprehensively and scientifically decode the genetic diversity of species, explore disease mechanisms, and understand species evolution. Recently, Pacbio has offered technology that can generate reads several thousand bases in size, and these long reads can span repetitive regions [10]. Although these long reads have a high error rate, they can be integrated with Illumina's short reads technology to improve accuracy [11]. In addition, new scaffold techniques, such as high-throughput chromosome conformation capture (Hi-C), allow the genome to be assembled to the level of whole chromosomes [12]. Pacbio single molecule real-time (SMRT) sequencing technology has been extensively used in the study of human diseases such as tuberculosis and influenza virus [13], as well as in the study of species evolution, such as the centromere of the human Y chromosome [14].

In this study, we report the genome assembly at the chromosome level in Lion-head geese for the first time using combined data generated by four advanced technologies, Illumina, SMRT, Bionano, and Hi-C. In addition, we investigated the genetic basis of body weight correlation in Lion-head goose, Wuzong goose, Huangzong goose and Magang goose by genome-wide association analysis, trying to identify the genes involved in body weight determination from different species. These will offer valuable resources for facilitating genetic research and the improvement of the species and for studying speciation and evolution in geese.

Methods

Animal selection

An adult healthy purebred male Lion-head goose (*Anser cygnoides*) with classical traits was selected for whole-genome sequencing and conducting *de novo* assembly from Shantou Baisha Research Institute of Original Species of Poultry and Stock. Blood and eight tissues (i.e., brain, pharyngeal pouch, head sarcoma, spleen, liver, chest muscle, kidney, and heart) from another four healthy adult accessions were collected for RNA-seq analysis. All applicable institutional and national guidelines for the care and use of animals were followed. All the animal work in this study was approved by the South China

Agricultural University Committee for Animal Experiments (approval ID: SYXK 2019-0136). All the research procedures and animal care activities were conducted based on the principles stated in the National and Institutional Guide for the Care and Use of Laboratory Animals.

Genome survey library construction and sequencing

To survey the genome profile, high-quality genomic DNA was extracted from the blood of the reference individual for whole-genome sequencing using the Qiagen Blood and Cell Culture DNA Midi Kit according to the manufacturer's instructions. For the quality control of purity, concentration, and integrity, we used Qubit 2.0 Fluorometry (Life Technologies, USA), NanoDrop 2000 spectrophotometer (Thermo Scientific), and pulse-field gel electrophoresis (Bio-rad CHEF-DR II), respectively. The following steps used for DNA extraction and quality control were similar. The short paired-end Illumina DNA library was constructed using the Illumina HiSeq system (with the paired-end 350 bp sequencing strategy). After performing the sequencing and obtaining the data, the k-mer analysis of reads for the genome survey was calculated by the Jellyfish program with the default parameters. Additionally, the genome size, heterozygosity ratio, and repeat sequence ratio were calculated with the GenomeScope tool based on the k-mer frequency of 17.

Genome sequencing and assembly strategies

A 40 kb *de novo* library for SMRT genome sequencing was constructed using the PacBio Sequel Ⅲ platform (Pacific Biosciences, USA). All of these reads were used for contigs assembly. A scalable and accurate long-read assembly tool, Canu (v1.8) [15], was employed to correct and assemble the PacBio reads with the listed parameters (minThreads = 4, genome size = 1200m, minOverlapLength = 700, minReadLength = 1000). The resulting contigs and corrected reads were used as inputs for HERA [16] to fill the gaps and produce longer contigs with default parameters. After that, Illumina paired-end clean data were mapped to the corrected contigs with the Burrows-Wheeler Aligner (BWA) [17], and the results were filtered by Q30 with Samtools (v1.8) [18]. At last, Pilon (v1.22) [19] was used to polish the assembly and enhance the base accuracy of the contigs.

Physical optical genome maps from BioNano were used to improve the assembly quality of the genome, with the ultimate goal of generating a chromosome-scale assembly. Nuclear DNA was

extracted from the blood sample of the reference individual and digested with nickase Direct Labeling Enzyme Ⅰ. After labeling, repairing and staining reactions, DNA was loaded onto the Saphyr Chip for sequencing to generate BioNano molecules. Afterward, the data were assembled with RefAligner and Assembler of BioNano Solve. The scaffold was established using BioNano Solve with HERA's contigs and a BioNano genome map. When encountering a conflict between a contig and the genome map, the contig was split to correct the false connection.

For Hi-C library, fresh blood was vacuum-infiltrated with 2% formaldehyde solution and then used for cross-link action. Later nuclear DNA was isolated from the reference animal and digested with the restriction enzyme Mbo I. The Hi-C library with insertion sizes of 350 bp was constructed and sequenced on the Illumina HiSeq X Ten instrument. The Hi-C reads were assigned to the scaffolds by Juicer [20]. The scaffolds were further clustered, ordered, and oriented to the chromosome-level scaffolds by 3D-DNA [21]. Thus, a heatmap of Hi-C chromosomal interaction was created using the HiC-pro software [22].

RNA-Seq and transcripts assembly

RNA-seq was conducted on blood and eight different tissues (i.e., brain, pharyngeal pouch, head sarcoma, spleen, liver, chest muscle, kidney, and heart) from four healthy adult accessions. Total RNA was extracted from four individuals using the TRIZOL reagent and purified following the manufacturer's protocols. The concentration and quality of the isolated RNA were assessed using the Nanodrop Spectrophotometer, Qubit 2.0 Fluorometry, and the Agilent 2100 bioanalyzer (Agilent Technologies, USA). Libraries construction and sequencing were performed using the Illumina NovaSeq 6000 platform. Raw RNA-seq data with 150 bp paired-end reads were trimmed for quality using Trimmomatic [23]. Thus, the Illumina sequence adaptors were removed, then low-quality and polluted reads were trimmed. Furthermore, Trinity [24] was arranged to *de novo* assemble the data after quality filtering. To remove redundant sequences, CD-HIT [25] was employed to remove highly identical transcript isoforms, retaining only the longest one. After filtering, the RNA-seq reads were mapped to the assembled genome using the default parameters of STAR [26].

Assembly evaluation

Finishing the genome assembly, quality control for the assembly's quality, accuracy, and integrity was predicted by Benchmarking Universal Single-Copy Orthologs (BUSCO, v 3.0), using aves_odb10 as the query [27].

Genome annotation

The genome assembly was annotated by MAKER, mainly including gene annotation and repeat annotation. The detailed pipeline was based on proteins from the Uniprot, the *de novo* assembly of RNA-seq data, and the total proteins of the relative species *Anser cygnoides* [28]. The transposable elements (TE) associated genes that were filtered out by the TEseeker database, and the results were used to conduct functional annotation using InterProScan. The repeat sequencing library was identified and annotated by a combination of LTR-FINDER and RepeatModeler. RepeatMasker and the query species "Chicken" were used to mask the repeats in the assembly, based on the Repbase database and the previous repeat sequence library. Tandem repeats were discovered by the Tandem Repeats Finder [29].

Gene families and phylogenetic analysis

Interspecific syntenic blocks between the Lion-head goose and duck were explored using MCscan [30] after coding sequence alignment by BLASTn. The same method was used for intraspecific collinearity analysis. To gain insight into the gene family evolution of the goose, we compared the gene families of Lion-head goose with the genomes of the following avian species: Zhedong white goose, duck, turkey, chicken, pigeon, saker, titmouse, and green lizard. Initially, alternative splicing and genes encoding less than 50 amino acids with a proportion of stop codon greater than 20% were filtered; meanwhile, the longest transcript of genes with multiple isoforms was retained to represent the gene. Similarity relationships among the protein sequences of species were aligned by BLASTP algorithm and clustered using OrthoMCL methodology with an expansion coefficient of 1.5 to obtain single- and multiple-copy gene families, and specific gene families of Lion-head goose. The sequences of the single-copy gene families were employed to perform multiple alignments by MUSCLE. Then RAxML [31] was used to construct a phylogenetic tree of nine species, with the lizard being designated an outgroup. Taking the divergence time of the pigeon and turkey (92.9Mya) as the calibration, the r8s [32] software was served to estimate the divergence time of the species and construct ultrametric trees. After filtering out gene

families with gene counts of more than 100 in some individual species, CAFÉ [33] was employed to detect gene families that had undergone expansion or contraction per million years independently along each branch of the phylogenetic tree. Subsequently, a gene ontology (GO) enrichment analysis of gene families was performed using the clusterProfiler package in R [34].

Experimental sample processing and genotyping

Blood samples of 514 geese were collected and stored in 2 mL tubes containing ACD anticoagulant for DNA extraction, and the weight of the geese was recorded. It was considered as a continuous trait rather than a categorical trait in the different goose control analyses of this study. This is due to the fact that continuous data are better for a small number of samples, only 514 individuals were analyzed in this study; continuous data can avoid some bias and are more sensitive and powerful to obtain more dependable results. DNA was extracted from blood samples using the HiPure Blood DNA Mini Kit (Magenbio, Guangzhou, China). The samples that passed the quality testing were subjected to library construction using Easy DNA Library Prep Kit (MGI, Shenzhen, China) and paired-end 100 sequencing using MGIseq 500. Raw data were filtered for adaptors and low quality reads using SOAPnuke software, and the filtered sequences were compared with the constructed goose reference genome using BWA software. Then variant detection as well as genotyping was performed using Samtools, GATK4 software. Variants were filtered based on a minimum allele frequency threshold of 0.05, a Hardy Weinberg equilibrium test significance threshold of 10e-7, and a maximum deletion rate threshold of 0.7. Principal component analysis (PCA) was performed and plotted with R. To understand the kinship among the samples, and phylogenetic trees were constructed.

Genome-wide association study

The sample variation was analyzed with the corresponding weight information using the asymptotic Wald test (assoc) in Plink. Combining the top 20 principal component values in the PCA analysis as covariates, the sample variances with the corresponding weight information were subjected to the linear analysis in Plink, that is, regression analysis with the inclusion of covariates. The variances with Bonferroni corrected p-values less than 0.05 in the results of the assoc and linear analyses were annotated. The corresponding genes of significantly related SNPs were used to identify the GO pathway.

Statistical analysis

R was used for statistical analyses. $p < 0.05$ was considered significant.

Results

Genome sequencing and assembly

The Lion-head goose is a famous local variety in China and one of the most giant goose breeds worldwide, with a unique appearance and social benefits. Here, we attempt to construct a highly continuous chromosome-scale genome of an adult purebred male Lion-head goose with a high degree of homozygosity to minimize heterozygous alleles. The following sequencing strategies were applied: Illumina sequencing, Pacbio SMRT sequencing, BioNano optical mapping, and Hi-C **(Supplementary Table S1)**. Assemble these data step by step and produce progressively improved assemblies **(Fig. 1A)**. A total of 185.37 Gb of high-quality Pacbio long reads were generated, representing a ~168× depth of the estimated 1.05 Gb genome with heterozygosity of 0.335% based on the k-mer analysis of the Illumina sequences **(Fig. 1B, Supplementary Table S2)**. Combing the *de novo* assembly of the Illumina and Pacbio sequences resulted in a draft genome of 1.20 Gb, yielding 1,859 contigs with a length of 13.7 Mb for contig N50 and 57.6 Mb for the longest **(Table 1)**. Furthermore, with the help of BioNano optical mapping, the scaffold N50 value was increased to 37 Mb. To obtain a chromosome-scale assembly, a set of ~230 Gb Hi-C data was used to orient, order, phase, and anchor the contigs. Approximately 97.27% of the reads assembled were anchored to 40 high-confidence pseudo-chromosomes (39 autosomes and Z chromosome) using the high-density genetic map **(Fig. 1C, Fig. 2)**. After polishing, we finally assembled the ultimate genome into 1.19 Gb with the final contig N50 of 20.59 Mb and scaffold N50 of 25.8 Mb, with a GC content of 42.39% **(Table 1, Supplementary Table S2)**. The structure and quality of the assembled genome were determined by mapping a Hi-C chromosomal contact map.

The completeness of the Lion-head goose genome assembly was assessed using the BUSCO gene set. The result showed that almost 99.02% of the reads were correctly mapped to the genome. We then evaluated the assembled genome with 98.24% single-copy and 1.76% duplicated orthologs from the

BUSCO dataset, confirming that 8,081 genes (96.92%) were intact in this genome. These results indicate the high reliability and integrity of the assembled genome **(Table 2, Supplementary Figure S1)**.

Genome annotation

To support the genome annotation, we conducted RNA-Seq analysis using RNA samples of blood and eight tissues (brain, pharyngeal pouch, head sarcoma, spleen, liver, chest muscle, kidney, and heart) from four healthy adult animals. The aggregate of 760 Gb raw reads was accumulated by the paired-end sequencing of the 36 constructed libraries. After filtering the adaptor and low-quality sequences, 723 Gb qualified Illumina reads remained, *de novo* assembled into unique transcripts (unigenes). Overall, a total of 216,229 unigenes were assembled and at the level N50, 5,082 nucleotides were obtained. Total 21,208 protein-coding gene annotations were predicted in Lion-head goose by combining *de novo* prediction, homologous protein prediction, and transcription alignment. After filtering TE-related genes, a total of 21,010 protein-coding gene annotations were finally obtained by the TE seeker database **(Fig. 2)**. Furthermore, a total of 8.15% repeat sequence and 4.10% tandem repeats of the genome were detected **(Table 3 and Supplementary Table S3)**.

Phylogenetic analysis

To investigate the genomic evolution of poultry, we compared the sequences of eight bird species (Lion-head goose, Zhedong white goose, duck, turkey, chicken, pigeon, saker, and titmouse) and green lizard, clustering the genes into 15,162 gene families **(Fig. 3A, Table 4)**. Among these, 6,422 single-copy gene families were identified and used to construct a phylogenetic tree **(Fig. 3B)**. This revealed that the geese and ducks were clustered into a subclade that probably evolved from a common ancestor approximately 28.42 million years ago (Mya). As expected, the Lion-head goose displayed a close relationship with the Zhedong white goose. The divergence time between the Lion-head goose and Zhedong white goose was estimated to be 13.79 Mya, and that between chicken and turkey was nearly 25.07 Mya. The above results confirmed the reliability of the tree.

Of all the gene families in the Lion-head goose, 4,233 gene families were significantly expanded and 324 were contracted. Compared with Zhedong white goose, the Lion-head goose had more gene families

and there are also more events of gene family expansion and contraction. Moreover, we mixed the gene family sets of several *Anatidae* varieties (duck, Zhedong white goose, Lion-head goose), and performed expansion and contraction analysis and corresponding GO enrichment analysis. In this task, the GO analysis of expanded gene families suggested the olfactory perception, such as detection of chemical stimulus involved in sensory perception of smell (GO:0050911, p = 6.97×10^{-8}), and odorant-binding (GO:0005549, p = 1.47×10^{-5}), both of which may be related to the adaptation of the species to find food in water **(Fig. 4A, Supplementary Table S4)**. Meanwhile, contracted gene families were concentrated in the areas of glucose synthesis and metabolism, such as hexokinase activity (GO:0004396, p = 7.64×10^{-26}), glucose binding (GO:0005536, p = 2.30×10^{-22}), cellular glucose homeostasis (GO:0001678, p = 6.84×10^{-18}), glycolytic process (GO:0006096, p = 1.75×10^{-15}), hexose metabolic process (GO:0019318, p = 2.66×10^{-14}), carbohydrate phosphorylation (GO:0046835, p = 1.68×10^{-9}), and glucose 6-phosphate metabolic process (GO:0051156, p = 1.27×10^{-9}), which may be closely related to characteristics of glycogen storage and utilization during migration **(Fig. 4B, Supplementary Table S5)**. Besides, 220 unique gene families (other species lack these gene families) of the Lion-head goose were identified and functionally annotated in GO categories, such as protein kinase activity (GO:0004672, p = 6.85×10^{-9}), the regulation of apoptotic process (GO:0042981, p = 5.78×10^{-34}), the adenylate cyclase-modulating G protein-coupled receptor signaling pathway (GO:0007188, p = 5.92×10^{-3}), and fatty-acyl-CoA reductase (alcohol-forming) activity (GO:0080019, p = 8.94×10^{-5}, **Fig. 4C, Supplementary Table S6**). Interestingly, we annotated a reproduction-related protein in the species-specific gene family, Sterile (Pfam ID: PF03015), acting on fatty-acyl-CoA reductase (alcohol-forming) activity, which may be related to the low reproductive rate caused by congenital infertility in geese.

Collinearity analysis allows one to judge molecular evolutionary events between species and explain the structural differences between the two genomes. We identified synteny blocks among avian genomes and found high collinearity between our assembly and the duck genome (genome size =1.19 Gb). Here, multiple chromosomes (Chr 1-5, 10, 12, 15, 17-20, 23, 26, 27, 29, 30, 32, 34, 36, 37, 39) of Lion-head goose were almost one-to-one collinear with those of the duck, but some chromosomal rearrangements occurred **(Fig. 3C, Supplementary Figure S2)**. For example, on some chromosomes like Chr 1, 2, 3,

and 4 of the duck genome, genes break and rearrange on the Lion-head goose genome, resulting in sequential inversion. In addition, some scaffolds such as Chr 9, 24, 25, 31, 35, 38 and 40, were not correlated with any chromosome of the duck genome due to the presence of a large number of tandem repeats. These results indicate that chromosome inversion and interchromosomal recombination may have occurred specifically in Lion-head goose during the evolutionary process, but this requires further investigation and verification. Moreover, Chr 4 of Lion-head goose was found to correspond to the sex chromosome Z of duck, except for the inversions of small patches of segments; therefore, we inferred that Chr 4 was the sex chromosome of the Lion-head goose. This information will be fundamental for comparative genomic studies in *Anatidae* animals.

Cluster analysis of different goose species

Blood samples were collected from 514 geese (including Lion-head goose, Wuzong goose, Huangzong goose and Magang goose), and their weight was recorded, with the Lion-head goose using the minimum weight, the Wuzong goose using the maximum weight, and the Huangzong goose and Magang goose using the average weight. That is, the Lion-head goose weighed at least 9 kg, the Wuzong goose weighed at most 2.5 kg, the Huangzong goose weighed about 3-4 kg, and the Magang goose weighed 4.8-5.5 kg. The average raw data was 1,520.60 Mb, the average sequencing depth was 12.05×, the average coverage was 7.56%, the average matching rate was 91.31%, and 44,858 SNP loci were retained for subsequent analysis after screening SNPs with minimum allele frequency <5%, Hardy-Weinberg equilibrium test significance threshold of 10e-7, and maximum deletion rate threshold of 0.7. We reconstructed the goose population structure using SNP data, revealing four distinct subpopulations. The PCA results demonstrated that the Lion-head Goose population was clearly distinguishable from the Magang Goose, Wuzong Goose and Huangzong Goose, and there was a clear differentiation within the species (**Fig. 5A**). The clustering of Magang Goose and Huangzong Goose was closer together, probably related to their closer geographical location and the existence of some genetic exchange. The phylogenetic tree results were consistent with the PCA results. The clustering of Magang Goose and Huangzong Goose was closer to each other, and they clustered into one branch with Wuzong Goose (**Fig. 5B**).

Variation identification from four different kinds of goose

From the Manhattan plot (**Fig. 5C**), a total of 10 significant signals were found to be associated with body weight trait in geese at the genome-wide level, including one significant SNP detected on Chr 2, 8, 9, and 33 respectively (-log10(Pvalue > 7.30), and six significant SNPs annotated by two genes on Chr 22, with the closest Manhattan plot SNP peak on Chr 9 for the gene *OR* (Olfactory receptor). Six significant SNPs on Chr 22 are located between 1,992,485 and 1,992,520 bp, a region that spans only a physical distance of 35 bp but contains six SNP loci, making it necessary to analyze these SNPs in this small region in detail to determine whether multiple QTL are involved. The most significant SNP in this region could explain about 8.19% of the phenotypic variation. Apart from significant SNPs, potentially significant QTLs were detected on many chromosomes (including Chr 2, 3, 6, 7, 10, 11, 15, 16, 20, 28, 30, 32, 36), with a total of 25 implied significant SNPs (4.90< -log(Pvalue) <7.30). On Chr 30, the suggestively significant SNPs were located between 1,258,517 and 2,422,666 bp, spanning approximately 1.16 Mb, with the most significant SNPs in this region explaining approximately 6.12% of the phenotypic variation (**Table 5**). In the present study, we identified genes in the region near the significant SNPs, annotating a total of 21 genes. These genes may be important in mediating growth and development, and we hypothesize that the *LDLRAD4* gene may play a key role in developmental plasticity in geese, while the *GPR180* gene may regulate the locomotor behavior of geese to make them stronger (**Fig. 6**).

Discussion

Despite the importance of the genus *Anser*, an economically important animal, the relative scarcity of genomic resources has largely hindered progress in studying genome evolution and molecular breeding in the major animals. High-quality chromosome-level genomes can provide key resources for studying. This study describes a chromosome-scale assembly of Lion-head goose obtained by a combination of data from the Illumina, SMRT, BioNano, and Hi-C platforms. The genome assembly is 1.19 Gb in length, and more than 97.27% of the assembled genome is anchored on 40 chromosomes. The BUSCO assessment revealed 99.02% complete genes in the assembled genome, making it a better-continuity and higher-quality genome assembly than the recently published Tianfu goose genome [35]. Compared with

the cultivated breed Tianfu goose, Lion-head goose, a traditional native breed, should occupy a more prominent position in the germplasm resources, and its evolving message can provide a reference for other local breeds which is worthy of in-depth study. Comparative genomic analysis revealed the genetic basis of interesting characters, which helped elucidate important biological implications and obtain solutions for genomic evolution between Lion-head geese and other species of *Anatidae* family, facilitating future genetic breeding programs. This is the first chromosomal level reference genome of Lion-head goose, providing important genomic data for the study of the family *Anatidae*.

We have identified genes associated with body weight traits in four different goose species, through GWAS analysis. Recently, there have been several studies related to agricultural traits that have achieved success in animal GWAS projects, for example, GWAS for improving reproductive performance and egg quality in geese and *TMEM161A* gene for embryo development [36]. In this study, *LDLRAD4* (low-density lipoprotein receptor class A domain containing 4), *OR* (Olfactory receptor), and *GPR180* (G protein-coupled receptor 180) were mainly found to function in body weight traits. Knockdown of *LDLRAD4* enhances transforming growth factor (TGF)-β-induced cell migration, which in turn regulates cell growth, differentiation, motility, apoptosis and matrix protein production [37]. The olfactory receptor (*OR2AT4*) has been shown to stimulate the proliferation of keratin-forming cells in peripheral human tissues [38]. *GPR180*, a component of the TGF-β signaling pathway, also has metabolic relevance in the body and may play an essential role in regulating adipose tissue and systemic energy metabolism [39]. Here we found some correlation between these genes and the TGF-β signaling, presumably this pathway also acts on body weight. Identifying of molecular genetic markers and the main effect QTL associated with critical agricultural traits is of great interest to breeders. Nevertheless, the candidate genes identified in this study were only detected by sequencing data and not experimentally validated. The functions of these candidate SNPs and gene markers need to be further verified by experimental results or other techniques. Thus, the findings in our GWAS study represent a valuable resource for geese and provide a new opportunity and basis for geneticists and breeders to work together to explore the genetics behind various agricultural traits.

Conclusions

In summary, we have obtained a high-quality chromosome-scale draft assembly of a purebred Lion-head goose, which provides a genetic basis for understanding the acquisition of related traits and facilitates advances in goose genomics and genetic improvement. Moreover, the candidate genes and their variants identified in this study will help clarify our understanding of goose selective breeding and the development of new breeds. The obtained genome sequence of Lion-head goose is a vital addition to the genome of genus *Anser* and is valuable for further understanding goose molecular breeding strategies. This genomic resource is also of high value for evolutionary studies of closely related species.

Data Availability

The final genome assembly data supporting the results of this article is available in the NCBI BioProject repository, [Accession number: PRJNA736831]. The raw re-sequencing genome data supporting of the GWAS study is available in the NCBI BioProject repository [Accession number: PRJNA552198, PRJNA552383, and PRJNA552384].

Additional Files

Supplementary Figure S1. BUSCO assessment of the assembly genome of Lion-head goose.

Supplementary Figure S2. Gene synteny between the Lion-head goose and duck genomes.

Supplementary Table S1. Statistics of sequenced clean data.

Supplementary Table S2. Statistics of genome survey.

Supplementary Table S3. Summary of repetitive sequence identification.

Supplementary Table S4. GO annotation of expanded gene families from Anatidae varieties (Duck, Zhedong white goose, Lion-head goose; Top 20).

Supplementary Table S5. GO annotation of contraction gene families from Anatidae varieties (Duck, Zhedong white goose, Lion-head goose; Top 20).

Supplementary Table S6. GO annotation of unique gene families from the Lion-head goose.

Abbreviations

BLAST: Basic Local Alignment Search Tool; BWA: Burrows-Wheeler Aligner; BUSCO:

Benchmarking Universal Single-Copy Orthologs; Chr: chromosome; GATK4: Genome Analysis Toolkit 4; Gb: gigabase pairs; GO: gene ontology; GPR180: G protein-coupled receptor 180; GWAS: genome-wide association study; HERA: Highly Efficient Repeat Assembly; Hi-C: high-throughput chromosome conformation capture; Kb: kilobase pairs; kg: kilogram; LDLRAD4: low-density lipoprotein receptor class A domain containing 4; LTR: long terminal repeat; Mb: megabase pairs; Mya: million years ago; NCBI: National Center for Biotechnology Information; OR: Olfactory receptor; OR2AT4: olfactory receptor family 2 subfamily AT member 4; PacBio: Pacific Biosciences; PCA: Principal component analysis; QTL: quantitative trait locus; RAxML: Randomized Axelerated Maximum Likelihood; RNA-seq: RNA sequencing; SMRT: single molecule real-time; SNP: single-nucleotide polymorphism; STAR: Spliced Transcripts Alignment to a Reference; TE: transposable element; TGF: transforming growth factor; TMEM161A: Transmembrane protein 161A.

Competing Interests

The authors declare that they have no conflict of interest.

Funding

This work was supported by the Key Research and Development Program of Guangdong Province (2020B020222001), the Construction of Modern Agricultural Science and Technology Innovation Alliance in Guangdong Province (2021KJ128, 2020KJ128), the National Modern Agricultural Industry Science and Technology Innovation Center in Guangzhou (2018kczx01), the Guangdong Provincial Promotion Project on Preservation and Utilization of Local Breed of Livestock and Poultry (4000-F18260), the Guangdong Basic and Applied Basic Research Foundation (2019A1515012006). The authors would like to thank the BGI in Shenzhen for their work on genome sequencing. We also thank the staff of Minglead Gene for providing the technical and computing support during the research.

Author’s Contributions

Q.X., Z.L., and X.Z. conceived and designed the research. X.Z., J.C., and Q.Z. coordinated the project. J.C. and Z.L. provided animal samples. Q.Z. and Z. X. collected and prepared the samples. Q.Z. performed sequencing, assembly and bioinformatics analysis. W.L., and F.C. led work identifying genes,

and H.L., W.C. aided with many aspects of gene identification and did the GO analyses. Q.Z., X.Z. wrote and revised the manuscript and the supplementary information. J.W., M.J., Z.H., H.Z., Z.L., and Q.X. participated in discussions and provided valuable advice. All authors read and approved the manuscript.

References

1. Hoyo JD, Elliott A, Sargatal J, et al. Handbook of the birds of the world. Barcelona: Lynx Edicions; 1992.
2. Madsen J, Marcussen LK, Knudsen N, et al. Does intensive goose grazing affect breeding waders? Ecol Evol 2019;**9**(24):14512-14522. doi:10.1002/ece3.5923.
3. Wang Y, Li SM, Huang J, et al. Mutations of TYR and MITF Genes are Associated with Plumage Colour Phenotypes in Geese. Asian-Australas J Anim Sci 2014;**27**(6):778-83. doi:10.5713/ajas.2013.13350.
4. Gao G, Zhao X, Li Q, et al. Genome and metagenome analyses reveal adaptive evolution of the host and interaction with the gut microbiota in the goose. Sci Rep 2016;**6**:32961. doi:10.1038/srep32961.
5. Yao Y, Yang YZ, Gu TT, et al. Comparison of the broody behavior characteristics of different breeds of geese. Poult Sci 2019;**98**(11):5226-5233. doi:10.3382/ps/pez366.
6. Lu L, Chen Y, Wang Z, et al. The goose genome sequence leads to insights into the evolution of waterfowl and susceptibility to fatty liver. Genome Biol 2015;**16**:89. doi:10.1186/s13059-015-0652-y.
7. Li HF, Zhu WQ, Chen KW, et al. Two maternal origins of Chinese domestic goose. Poult Sci 2011;**90**(12):2705-10. doi:10.3382/ps.2011-01425.
8. Tang J, Shen X, Ouyang H, et al. Transcriptome analysis of pituitary gland revealed candidate genes and gene networks regulating the growth and development in goose. Anim Biotechnol 2020:1-11. doi:10.1080/10495398.2020.1801457.
9. Zhang X, Wang J, Li X, et al. Transcriptomic investigation of embryonic pectoral muscle reveals increased myogenic processes in Shitou geese compared to Wuzong geese. Br Poult Sci 2021;**62**(5):650-657. doi:10.1080/00071668.2021.1912292.
10. Ardui S, Ameur A, Vermeesch JR, et al. Single molecule real-time (SMRT) sequencing comes of age: applications and utilities for medical diagnostics. Nucleic Acids Res 2018;**46**(5):2159-2168. doi:10.1093/nar/gky066.
11. Yoshinaga Y, Daum C, He G, et al. Genome Sequencing. Methods Mol Biol 2018;**1775**:37-52.

doi:10.1007/978-1-4939-7804-5_4.
12. Kong S, Zhang Y. Deciphering Hi-C: from 3D genome to function. Cell Biol Toxicol 2019;**35**(1):15-32. doi:10.1007/s10565-018-09456-2.
13. Nakano K, Shiroma A, Shimoji M, et al. Advantages of genome sequencing by long-read sequencer using SMRT technology in medical area. Hum Cell 2017;**30**(3):149-161. doi:10.1007/s13577-017-0168-8.
14. Jain M, Olsen HE, Turner DJ, et al. Linear assembly of a human centromere on the Y chromosome. Nat Biotechnol 2018;**36**(4):321-323. doi:10.1038/nbt.4109.
15. Koren S, Walenz BP, Berlin K, et al. Canu: scalable and accurate long-read assembly via adaptive k-mer weighting and repeat separation. Genome Res 2017;**27**(5):722-736. doi:10.1101/gr.215087.116.
16. Du H, Liang C. Assembly of chromosome-scale contigs by efficiently resolving repetitive sequences with long reads. Nat Commun 2019;**10**(1):5360. doi:10.1038/s41467-019-13355-3.
17. Li H, Durbin R. Fast and accurate short read alignment with Burrows-Wheeler transform. Bioinformatics 2009;**25**(14):1754-60. doi:10.1093/bioinformatics/btp324.
18. Li H, Handsaker B, Wysoker A, et al. The Sequence Alignment/Map format and SAMtools. Bioinformatics 2009;**25**(16):2078-9. doi:10.1093/bioinformatics/btp352.
19. Walker BJ, Abeel T, Shea T, et al. Pilon: an integrated tool for comprehensive microbial variant detection and genome assembly improvement. Plos One 2014;**9**(11):e112963. doi:10.1371/journal.pone.0112963.
20. Durand NC, Shamim MS, Machol I, et al. Juicer Provides a One-Click System for Analyzing Loop-Resolution Hi-C Experiments. Cell Syst 2016;**3**(1):95-8. doi:10.1016/j.cels.2016.07.002.
21. Dudchenko O, Batra SS, Omer AD, et al. De novo assembly of the Aedes aegypti genome using Hi-C yields chromosome-length scaffolds. Science 2017;**356**(6333):92-95. doi:10.1126/science.aal3327.
22. Servant N, Varoquaux N, Lajoie BR, et al. HiC-Pro: an optimized and flexible pipeline for Hi-C data processing. Genome Biol 2015;**16**(1). doi:10.1186/s13059-015-0831-x.
23. Bolger AM, Lohse M, Usadel B. Trimmomatic: a flexible trimmer for Illumina sequence data. Bioinformatics 2014;**30**(15):2114-20. doi:10.1093/bioinformatics/btu170.
24. Grabherr MG, Haas BJ, Yassour M, et al. Full-length transcriptome assembly from RNA-Seq data without a reference genome. Nat Biotechnol 2011;**29**(7):644-52. doi:10.1038/nbt.1883.
25. Huang Y, Niu B, Gao Y, et al. CD-HIT Suite: a web server for clustering and comparing biological sequences. Bioinformatics 2010;**26**(5):680-2. doi:10.1093/bioinformatics/btq003.

26. Dobin A, Davis CA, Schlesinger F, et al. STAR: ultrafast universal RNA-seq aligner. Bioinformatics 2013;**29**(1):15-21. doi:10.1093/bioinformatics/bts635.

27. Seppey M, Manni M, Zdobnov EM. BUSCO: Assessing Genome Assembly and Annotation Completeness. Methods Mol Biol 2019;**1962**:227-245. doi:10.1007/978-1-4939-9173-0_14.

28. Lu L, Chen Y, Wang Z, et al. The goose genome sequence leads to insights into the evolution of waterfowl and susceptibility to fatty liver. Genome Biol 2015;**16**:89. doi:10.1186/s13059-015-0652-y.

29. Benson G. Tandem repeats finder: a program to analyze DNA sequences. Nucleic Acids Res 1999;**27**(2):573-80. doi:10.1093/nar/27.2.573.

30. Wang Y, Tang H, Debarry JD, et al. MCScanX: a toolkit for detection and evolutionary analysis of gene synteny and collinearity. Nucleic Acids Res 2012;**40**(7):e49. doi:10.1093/nar/gkr1293.

31. Stamatakis A. RAxML version 8: a tool for phylogenetic analysis and post-analysis of large phylogenies. Bioinformatics 2014;**30**(9):1312-3. doi:10.1093/bioinformatics/btu033.

32. Sanderson MJ. r8s: inferring absolute rates of molecular evolution and divergence times in the absence of a molecular clock. Bioinformatics 2003;**19**(2):301-2. doi:10.1093/bioinformatics/19.2.301.

33. Han MV, Thomas GW, Lugo-Martinez J, et al. Estimating gene gain and loss rates in the presence of error in genome assembly and annotation using CAFE 3. Mol Biol Evol 2013;**30**(8):1987-97. doi:10.1093/molbev/mst100.

34. Yu G, Wang LG, Han Y, et al. clusterProfiler: an R package for comparing biological themes among gene clusters. Omics 2012;**16**(5):284-7. doi:10.1089/omi.2011.0118.

35. Li Y, Gao G, Lin Y, et al. Pacific Biosciences assembly with Hi-C mapping generates an improved, chromosome-level goose genome. Gigascience 2020;**9**(10). doi:10.1093/gigascience/giaa114.

36. Gao G, Gao D, Zhao X, et al. Genome-Wide Association Study-Based Identification of SNPs and Haplotypes Associated With Goose Reproductive Performance and Egg Quality. Front Genet 2021;**12**:602583. doi:10.3389/fgene.2021.602583.

37. Nakano N, Maeyama K, Sakata N, et al. C18 ORF1, a novel negative regulator of transforming growth factor-beta signaling. J Biol Chem 2014;**289**(18):12680-92. doi:10.1074/jbc.M114.558981.

38. Cheret J, Bertolini M, Ponce L, et al. Olfactory receptor OR2AT4 regulates human hair growth. Nat Commun 2018;**9**(1):3624. doi:10.1038/s41467-018-05973-0.

39. Balazova L, Balaz M, Horvath C, et al. GPR180 is a component of TGFbeta signalling that promotes

thermogenic adipocyte function and mediates the metabolic effects of the adipocyte-secreted factor CTHRC1. Nat Commun 2021;**12**(1):7144. doi:10.1038/s41467-021-27442-x.

Figure legends

Figure 1. Sequencing process and presentation. (A) The pipeline for generating chromosome scale scaffolds. Four sets of sequencing data (PacBio, BioNano optical mapping, Hi-C, and Illumina paired-end reads) were produced to generate the Lion-head goose reference genome. A tiered assembled technique using optical mapping data, followed by Hi-C assembly, was used to produce a high-quality assembled genome. **(B)** K-mer (17-mer) analysis for estimating the genome size of Lion-head goose. **(C)** Heatmap of Hi-C chromosomal interaction density. Hi-C interactions among 40 pseudo-chromosomes ordered by length. Dark red indicates strong interactions and yellow indicates weak interactions.

Figure 2. Distribution of genomic features. Concentric circle diagram presents the distribution of genomic features of Lion-head goose using nonoverlapping sliding windows with sizes of 1 Mb (from outmost to innermost). **(A)** the assembled pseudo-chromosome and the corresponding position; **(B)** gene density calculated on the basis of the number of genes; **(C)** average expression level of overall 36 samples. eight tissues (i.e., brain, pharyngeal pouch, head sarcoma, spleen, liver, chest muscle, kidney and heart) and blood collected from four healthy adult animals; **(D)** GC content; **(E)** density of TE; **(F)** gene synteny and collinearity analysis.

Figure 3. Phylogenetic relationship and comparative genomics analyses. (A) Venn diagram showing the orthologous gene families shared among the genomes of Lion-head goose, Zhedong white goose, chicken, duck, and turkey. **(B)** Phylogenetic tree with the divergence times and history of orthologous gene families. Numbers on the nodes represent divergence times. The numbers of gene families that expanded (green) or contracted (red) in each lineage after speciation are shown on the circles of the corresponding branch. **(C)** Gene comparison of homologous chromosomes between Lion-head goose and duck. Gray lines indicate collinearity between the genomes.

Figure 4. GO enrichment analysis of gene families. (A) Expanded and **(B)** contracted gene families from Anatidae varieties (duck, Zhedong white goose, Lion-head goose). **(C)** Unique gene families from the Lion-head goose. The bar graph on the left represents the P-adjust gradient of GO terms, and the color corresponds to the number on the x-axis (i.e. -log (P.adj)). The bluer the color is, the smaller the P-adjust is, and the more significant it is. The redder the color is, the larger the P-adjust is, and the less significant it is. The upper right bar chart exhibits that several genes act together on the terms below. The lower right chart displays the intersection of the genes of each term; the dots connected by lines represent the intersection of multiple terms; the black dots represent "yes", and the gray dots represent "no".

Figure 5. Comparison of different goose species and genome-wide association analysis of body weight. (A) Principal component analysis of sample structures using first two principal components. **(B)** The phylogenetic trees of several goose species. **(C)** Manhattan plot of genome-wide association analysis for body weight. The X-axis indicates chromosomes, and Y-axis indicates the P values of the SNP markers. The red solid line indicates the threshold P value for genome-wide significance. The blue solid line indicates the threshold P value for the significance of potential association.

Figure 6. GO analysis of body weight-related genes:(A) Biological processes level, (B) Cellular component level.

Table 1: Statistics of genome assembly quality.

Method	Type	N50	Number	Max length
PacBio	Contig	13,732,492	1,859	57,632,554
BioNano	Scaffold	37,123,516	110	98,698,500
Hi-C	Contig	21,589,146	1,318	91,420,268
	Scaffold	27,064,542	1,266	98,160,899
Assembly	Contig	21,589,146	1,318	91,420,268
	Scaffold	27,064,542	1,266	98,160,899

Table 2: Summary of BUSCOs genome evaluation.

Item	Number	Percent (%)
Complete BUSCOs (C)	8081	96.9
Complete and single-copy BUSCOs (S)	7939	95.2
Complete and duplicated BUSCOs (D)	142	1.7
Fragmented BUSCOs (F)	93	1.1
Missing BUSCOs (M)	164	2.0
Total BUSCO groups searched	8338	100

Table 3: Summary of repeat classification.

Type	Length	Percent
Long interspersed nuclear element	76,437,757	5.98
Simple sequence repeats	23,026,311	1.80
Low complexity	4,663,288	0.36
Tandem repeats	52,426,380	4.10
Total	156,553,736	12.25

Table 4: Summary of gene families from several species.

Animals	Expansion	Contraction	Unique	Total
Lion-head goose	1,191	1,328	220	12,451
Zhedong white goose	53	1,465	2	12,106
Chicken	228	663	94	13,049
Duck	267	1,718	80	12,201
Turkey	582	911	46	12,829
Pigeon	171	694	21	12,454
Saker	128	710	7	12,427
Titmouse	83	1,215	15	12,407
Lizard	368	1,736	282	13,034

Table 5: Genome-wide association analysis of body weight in geese.

Chr	Allele	Physical position	Regression coefficient	P value	Genes
2	A	108496954	-0.1886	1.01E-08	LDLRAD4
2	G	7706165	0.2612	6.98E-06	LDLRAD4
3	T	123032780	-0.3979	6.03E-07	EGF, KBTBD

6	A	13264157	-0.24	6.28E-07	TSPAN
6	T	66027192	0.2127	8.14E-07	IGFN1
7	T	39117443	-0.3131	4.66E-06	—
8	T	14712470	0.1865	8.97E-09	PPEF1
9	T	26883582	-2.7E+12	0	OR
10	C	23997415	-0.3032	1.19E-06	—
10	C	23997399	-0.2542	1.05E-05	—
10	T	23997401	-0.2542	1.05E-05	—
11	A	22838749	0.1548	9.55E-06	—
15	T	10257386	0.2527	2.96E-07	GPR180, GPCPD1
16	A	1477673	-0.1892	6.53E-06	—
16	G	1477679	-0.1891	6.78E-06	—
20	A	8531879	0.151	3.05E-06	—
22	A	1992485	-0.3972	6.51E-09	GALNT, AUTS2
22	A	1992518	-0.3973	7.69E-09	GALNT, AUTS2
22	G	1992501	-0.3974	7.94E-09	GALNT, AUTS2
22	C	1992505	-0.3974	7.94E-09	GALNT, AUTS2
22	C	1992507	-0.3974	7.94E-09	GALNT, AUTS2
22	G	1992515	-0.3974	7.94E-09	GALNT, AUTS2
28	C	3587271	0.2936	5.81E-08	PPP1R15B, FGD2
28	G	4472051	-0.2359	2.82E-06	PPP1R15B, FGD2
30	C	1652158	-0.3469	7.53E-07	SH2
30	T	1258517	0.2205	1.48E-06	SH2
30	G	2422665	0.1894	2.04E-06	SH2
30	T	2422666	0.1894	2.04E-06	SH2
30	A	1652207	-0.3289	2.3E-06	SH2
30	T	2269897	0.211	9.22E-06	SH2
32	G	655318	0.2599	7.95E-06	—
33	A	975487	0.2567	1.07E-08	SDHA
36	A	1523127	-0.3274	9.86E-07	SPRY
36	G	1523132	-0.3216	1.7E-06	SPRY
36	C	1523105	-0.3291	1.72E-06	SPRY

Figure 1

Click here to access/download;Figure;1.tif

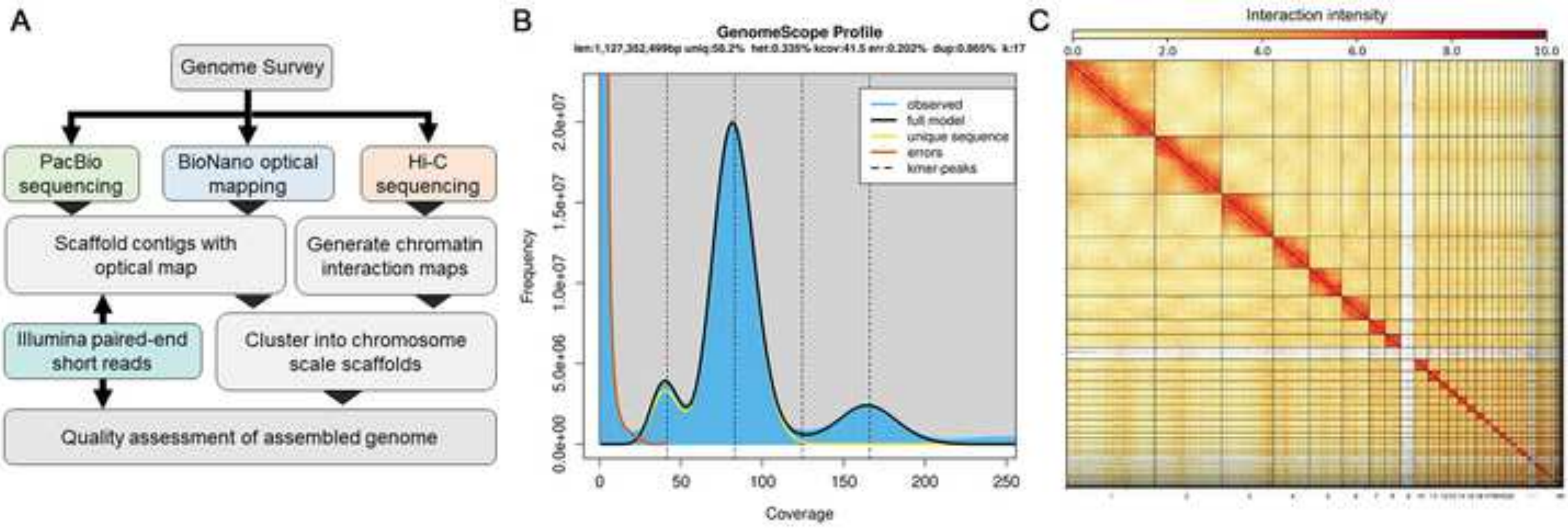

Figure 2

Click here to access/download;Figure;2.tif

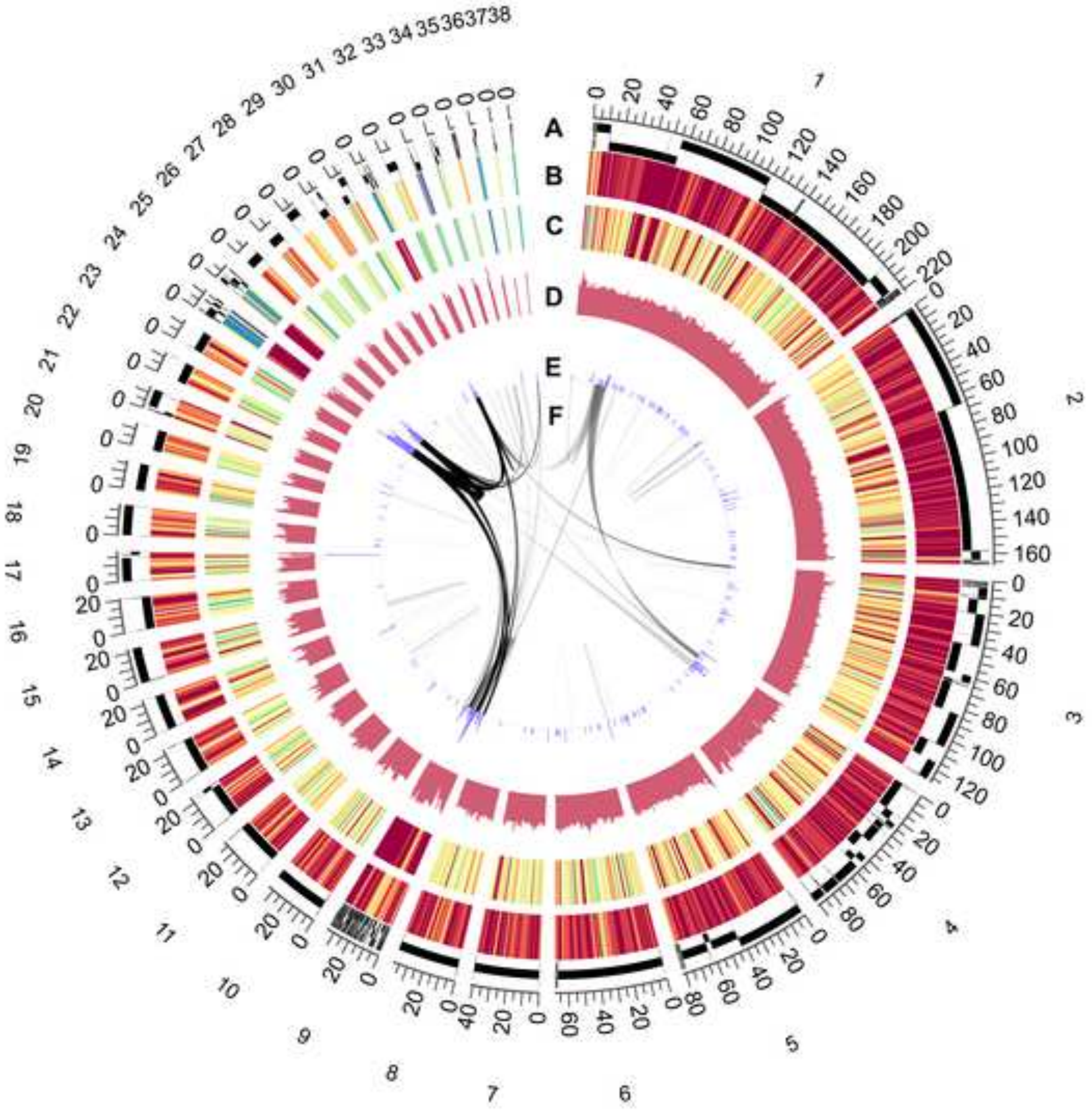

Figure 3

Click here to access/download;Figure;3.tif

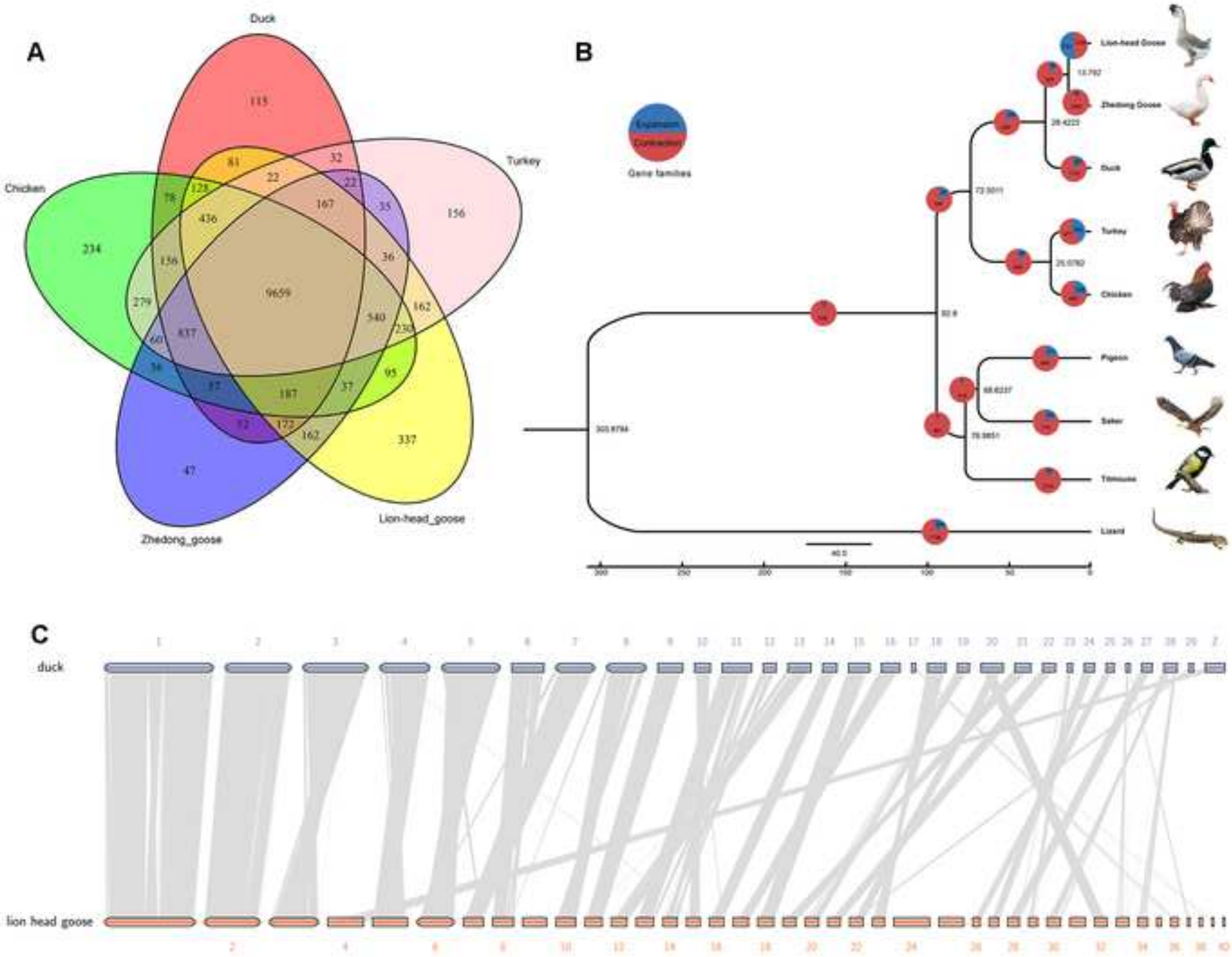

Figure 4 Click here to access/download;Figure;444.tif

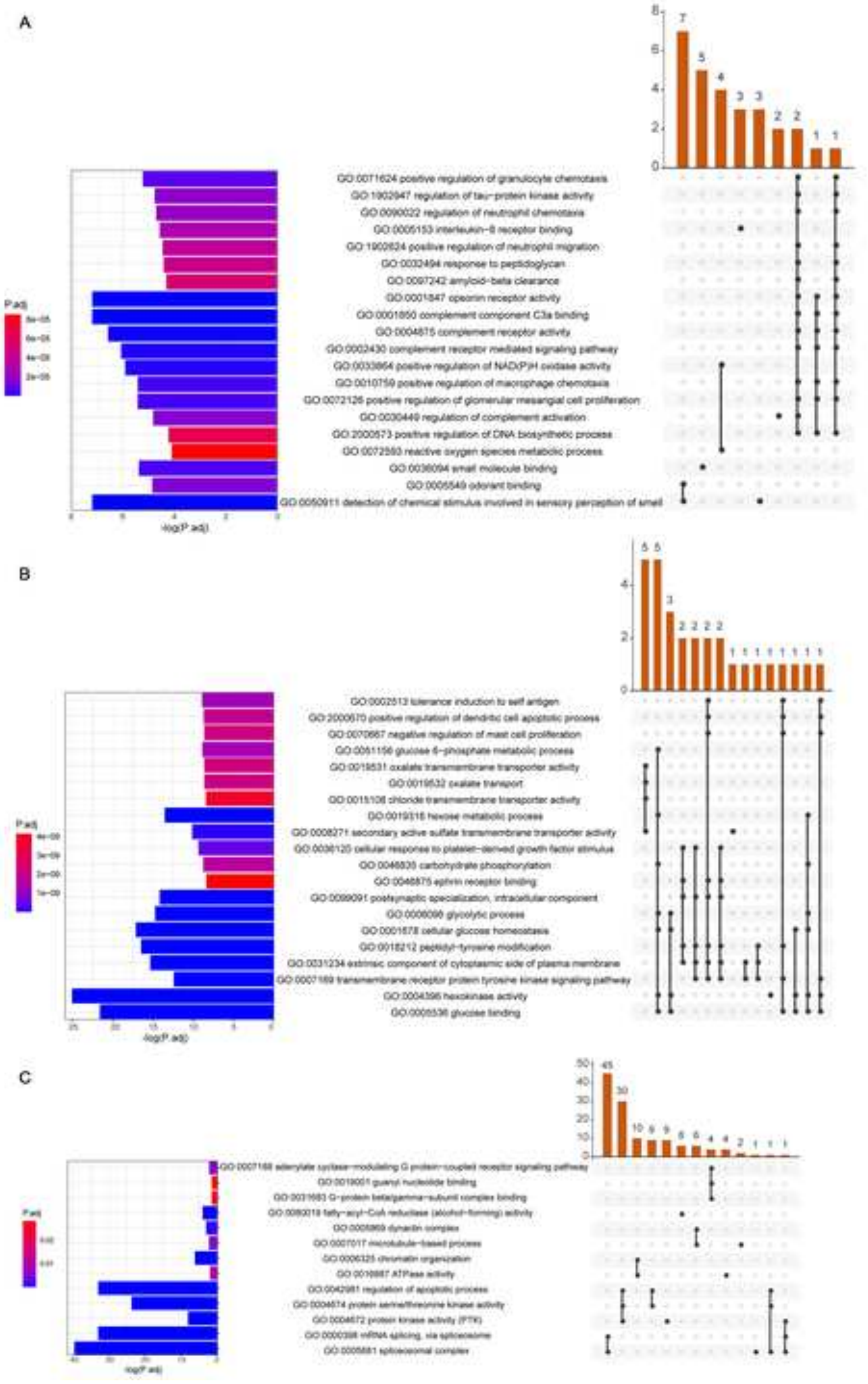

Figure 5

Click here to access/download;Figure;5.tif

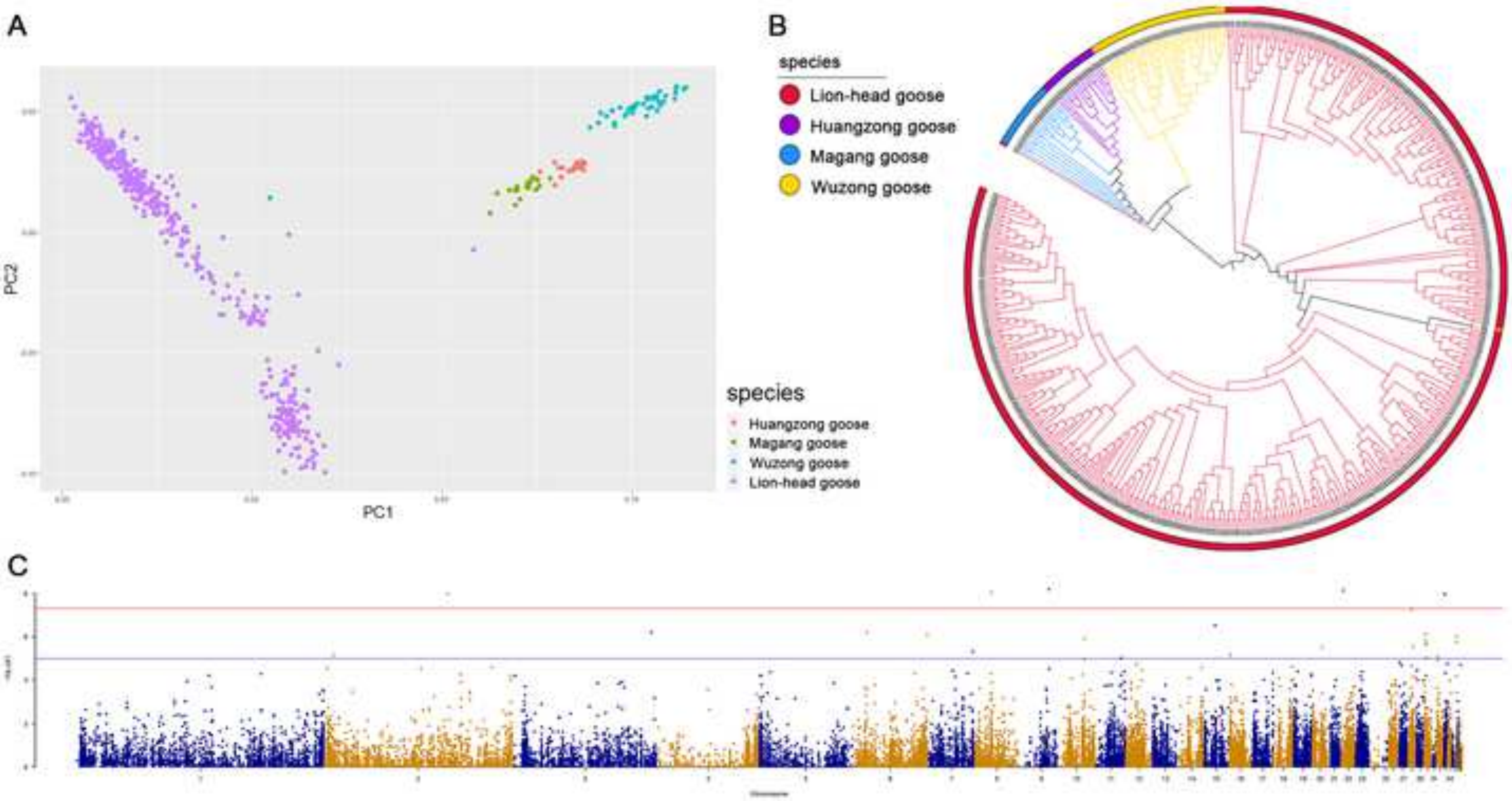

Figure 6

Click here to access/download;Figure;6.tif

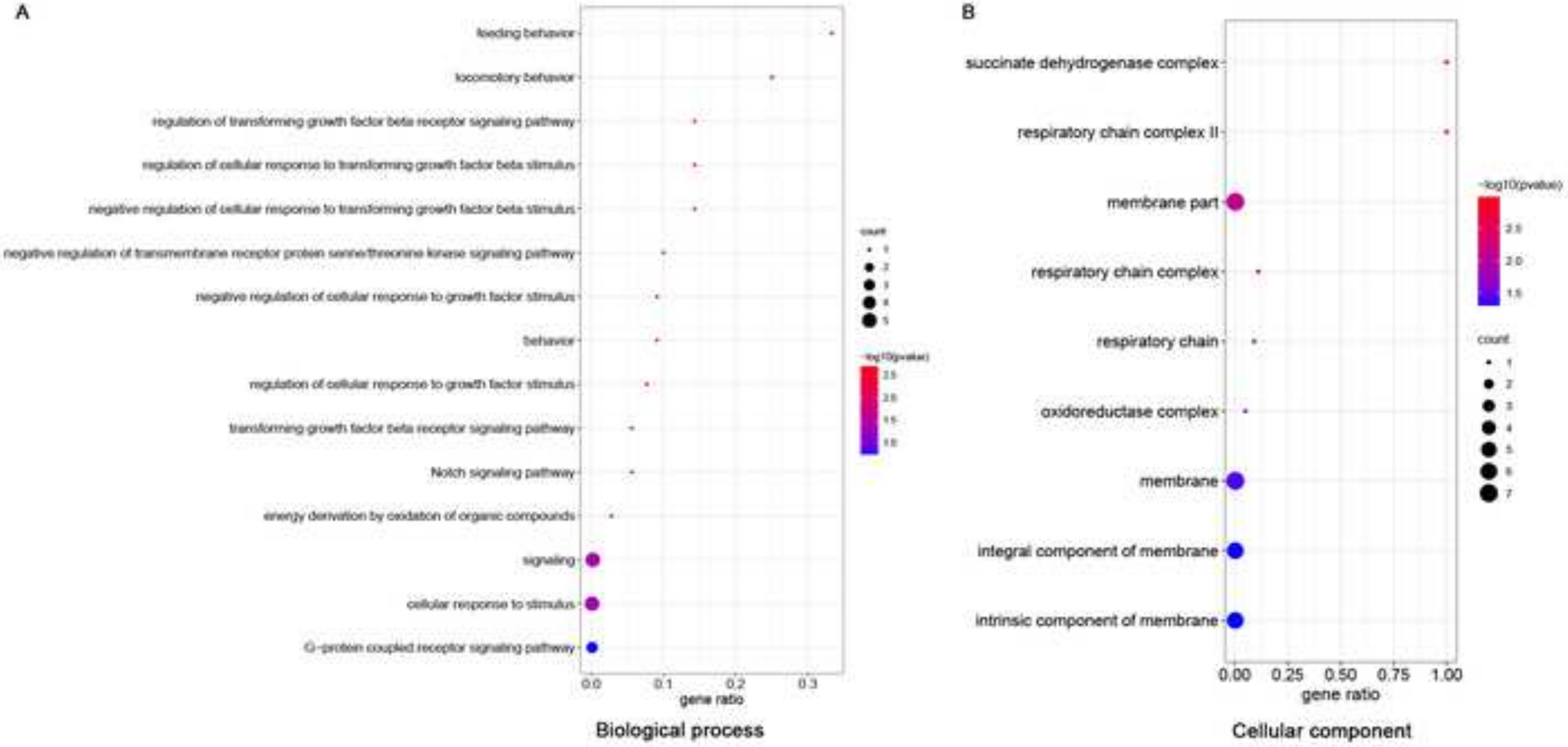

Click here to access/download
Supplementary Material
Supplemental_Information.docx

Personal Cover Click here to access/download;Personal Cover;renamed_d625b.docx

GigaScience

Dear Editor,

I hereby submit a research article entitled “Chromosome-level genome assembly of goose provides insight into the adaptation and growth of local goose breeds” for publication in multidisciplinary journal *GigaScience* on behalf of my co-authors. We have read and have abided by the statement of ethical standards for manuscripts submitted to *GigaScience*.

In this study, we assembled a high-quality chromosome-level 1.19 Gb genome of the lion-head goose. The genome assembly has contig and scaffold N50 of 20.59 Mb and 25.8 Mb, respectively. The comparative genomic results show that the genomes of lion-head goose and other goose species were similar in size and had a common origin. And in the population study, a genome-wide association study (GWAS) was performed on 514 geese including Wuzong goose, Huangzong goose, Magang goose and lion-head goose, yielding an average of 1520.6 Mb of raw data with 12.05× sequencing depth, identifying 44,858 SNPs. GWAS showed that six SNPs were significantly associated with body weight and 25 were potentially associated. Among the significantly associated SNP markers were annotated as *LDLRAD4*, *GPR189*, *OR*, etc., which enrich in the regulation of growth factor receptors signaling pathways. We imply that these results can play a significant role in promoting the goose industry and laying a foundation for future research.

We believe that these novel findings will approach a broad audience, including geneticists, breeders, and the general public. We are convinced that the results and impact of this work are consistent with the aims and style of *GigaScience*.

The authors declare that they have no conflict of interest. This manuscript describes original work and is not under consideration by any other journal. All authors approved the manuscript and this submission. All data has been uploaded to the NCBI BioProject database. Thank you for receiving our manuscript and considering it for review. We appreciate your time and look forward to your response.

With kind personal regards,

Professor Xinheng Zhang

College of Animal Science, South China Agricultural University

483 Wushan Road, Tianhe District, Guangzhou, 510642, China

E-mail: xhzhang@scau.edu.cn
