# Supplementary material for: Chromosome-level genome assembly of goose provides insight into the adaptation and growth of local goose breeds
Source: Gigascience. 2023 Feb 3;12:giad003. doi: 10.1093/gigascience/giad003 (PMC9896136; doi:10.1093/gigascience/giad003)
Supplement: giad003_GIGA-D-22-00016_Revision_1 [file giad003_giga-d-22-00016_revision_1.pdf]

GigaScience

Chromosome-level genome assembly of goose provides insight into the adaptation and growth of local goose breeds

--Manuscript Draft--

**Manuscript Number:**	GIGA-D-22-00016R1
**Full Title:**	Chromosome-level genome assembly of goose provides insight into the adaptation and growth of local goose breeds
**Article Type:**	Research
**Funding Information:**	Key Research and Development Program of Guangdong Province (2020B020222001) — Not applicable<br>Construction of Modern Agricultural Science and Technology Innovation Alliance in Guangdong Province (2021KJ128, 2020KJ128) — Not applicable<br>National Modern Agricultural Industry Science and Technology Innovation Center in Guangzhou (2018kczx01) — Not applicable<br>Guangdong Provincial Promotion Project on Preservation and Utilization of Local Breed of Livestock and Poultry (4000-F18260) — Not applicable<br>Guangdong Basic and Applied Basic Research Foundation (2019A1515012006) — Not applicable
**Abstract:**	Backgrouond: Anatidae contains numerous waterfowl species with great economic value, but the genetic diversity basis remains insufficiently investigated. Here, we report a chromosome-level genome assembly of Lion-head goose (Anser cygnoides), a native breed in South China, through the combination of PacBio, Bionano and Hi-C technologies. Findings: The assembly had a total genome size of 1.19 Gb, consisting of 1,859 contigs with an N50 length of 20.59 Mb, generating 40 pseudochromosomes, representing 97.27% of the assembled genome, and identifying 21,208 protein-coding genes. Comparative genomic analysis revealed that geese and ducks diverged approximately 28.42 million years ago, and geese have undergone massive gene family expansion and contraction. To identify genetic markers associated with body weight in different geese breeds including Wuzong goose, Huangzong goose, Magang goose and Lion-head goose, a genome-wide association study was performed, yielding an average of 1,520.6 Mb of raw data with detecting 44,858 SNPs. GWAS showed that six SNPs were significantly associated with body weight and 25 were potentially associated. The significantly associated SNPs were annotated as LDLRAD4 , GPR180 , OR , enriching in growth factor receptors regulation pathways. Conclusions: We present the first chromosome-level assembly of the Lion-head goose genome, which will expand the genomic resources of the Anatidae family, providing a basis for adaptation and evolution. Candidate genes significantly associated with different goose breeds may serve to understand the underlying mechanisms of weight differences.
**Corresponding Author:**	Xinheng Zhang<br>South China Agricultural University<br>Guangzhou, Guangdong CHINA
**Corresponding Author Secondary Information:**	
**Corresponding Author's Institution:**	South China Agricultural University
**Corresponding Author's Secondary Institution:**	
**First Author:**	Qiqi Zhao
**First Author Secondary Information:**	

Powered by Editorial Manager® and ProduXion Manager® from Aries Systems Corporation

**Order of Authors:**	Qiqi Zhao
	Junpeng Chen
	Zi Xie
	Jun Wang
	Keyu Feng
	Wencheng Lin
	Hongxin Li
	Zezhong Hu
	Weiguo Chen
	Feng Chen
	Muhammad Junaid
	Huanmin Zhang
	Zhenping Lin
	Qingmei Xie
	Xinheng Zhang
**Order of Authors Secondary Information:**	
**Response to Reviewers:**	Dear editor,<br><br>Thank you very much for your letter dated 07 Apr 2022, and the reviewer's comments concerning our manuscript "Chromosome-level genome assembly of goose provides insight into the adaptation and growth of local goose breeds" (ID: GIGA-D-22-00016).<br><br>These comments are of great value and very helpful for revising and improving our paper, as well as the importance guiding significant to our research. According to your opinion and request, we have made some revisions to the original manuscript. The responses to the questions are shown below, the black bold font part is the questions raised by the reviewers, and the dark blue font part is our reply.<br><br>We have resubmitted the revised version in both PDF and MS word format, on the system for your review. The revised parts are marked in yellow in the MS word file of the revised manuscript for your review.<br><br>Should you have any questions, please contact us without hesitate.<br><br>Best wishes,<br>Xinheng Zhang<br><br><br>Questions and Responses:<br><br>Reviewer #1: Annotation and assembly of the Lion-headed goose genome were performed using a combination of four technologies, including Illumina, SMRT, Bionano, and Hi-C. Based on the chromosome-level genome sequence, a genome-wide association study (GWAS) was performed on 514 geese including Wuzong goose, Huangzong goose, Magang goose, and Lion-head goose, yielding an average of 1.52 Gb raw data, identifying 44,858 SNPs The GWAS results showed that six SNPs were significantly associated with body weight and 25 were potentially associated.<br>The authors should explore the spatial organization of chromatin and gene expression in the goose blood tissue (inter-pseudo-chromosomal interaction patterns, compartments, topologically associating domains, and promoter-enhancer interactions), to check if the goose genome shows similar basic principles to other animal genomes in terms of its inter-chromosomal interaction pattern, compartments, topologically associating domains, and promoter-enhancer interactions. Providing basic characterization of the three-dimensional organization of the goose genome, and

supports the conclusion the goose genome assembly is chromosomal-level. For the four goose population, the authors should perform selective sweep analysis (Fst、XP-CLR、π or Tajima's D), and combine with the GWAS results to illustrate the topic of the this article.
Response: Thank you very much for your questions and suggestions. The purpose of this article is to construct a complete genome map of the Lion-head Goose and to analyze the evolutionary relationships with other avian species, and to determine the genomic level variations in the selection of Lion-head, Magang, Huangzong and Wuzong geese. In response to your questions, we made the following replies.
1. The question of spatial organization of chromatin and gene expression in the goose blood you raise is a good point, and we have reviewed the relevant literature and are concerned that this issue has been investigated in the chromatin in goose liver tissue of Tianfu goose [1], chicken embryonic fibroblasts (CEF) and adult erythrocytes [2], suggesting the additive effects of enhancers on the transcriptional levels of target genes, and the important role of topologically associated domains (TADs) as genomic regulatory units, respectively. The aim in this article is to construct the Lion-head goose genome and perform evolutionary and population analysis. The analysis of the samples collected in this study did not perform blood whole transcriptome analysis, so we could not perform the analysis of spatial organization of chromatin and gene expression. But your comments are constructive and we will focus on your suggestions for further study in the next article.
2. The diploid cotton genome researchers used Hi-C technology to anchored and oriented 1,573 Mb of assembly to 13 pseudochromosomes [3]. The chromosome-level genome assembly of scimitar-horned oryx, generated 29 chromosomes using 10X Chromium sequencing and Hi-C technology [4]. The genome of autopolyploid sugarcane Saccharum spontaneum L. was assembled using a Hi-C-based physical map to obtain 32 pseudochromosomes [5]. The Musa Balbisiana Genome constructed a high-throughput chromosomal conformational capture (Hi-C) library with 430 Mb (87.27%) of assembly and 94.0% of genes placed on 11 chromosome groups [6]. The above examples fully demonstrate that the construction of chromosomal level genome by Hi-C is feasible and credible, and it is a widely used assembly method. Most researchers use Hi-C technology to illustrate genomic chromosome number. In this study, the Hi-C method we used to construct chromosomal level genomes is also an assisted assembly method based on Hi-C sequencing results, showing the three-dimensional structural characteristics of goose genomes.
3. For the four goose populations, we performed selective sweep analysis and combine with the GWAS results. Related description was added in L227-232, L341-365.

Reference:
[1]Li Y, Gao G, Lin Y, Hu S, Luo Y,Wang G, et al. Pacific Biosciences assembly with Hi-C mapping generates an improved, chromosome-level goose genome. Gigascience 2020;9:
[2]Fishman V, Battulin N, Nuriddinov M, Maslova A, Zlotina A,Strunov A, et al. 3D organization of chicken genome demonstrates evolutionary conservation of topologically associated domains and highlights unique architecture of erythrocytes' chromatin. Nucleic Acids Res 2019;47:648-65
[3] Du X, Huang G, He S, Yang Z, Sun G,Ma X, et al. Resequencing of 243 diploid cotton accessions based on an updated A genome identifies the genetic basis of key agronomic traits. Nat Genet 2018;50:796-802
[4] Humble E, Dobrynin P, Senn H, Chuven J, Scott A F,Mohr D W, et al. Chromosomal-level genome assembly of the scimitar-horned oryx: Insights into diversity and demography of a species extinct in the wild. Mol Ecol Resour 2020;20:1668-81
[5] Zhang J, Zhang X, Tang H, Zhang Q, Hua X,Ma X, et al. Publisher Correction: Allele-defined genome of the autopolyploid sugarcane Saccharum spontaneum L. Nat Genet 2018;50:1754
[6] Wang Z, Miao H, Liu J, Xu B, Yao X,Xu C, et al. Musa balbisiana genome reveals subgenome evolution and functional divergence. Nat Plants 2019;5:810-21

Line 27, "and identifying 21,208 protein-coding genes". Previous studies have shown that there are 16,150, 16288 and 17568 genes in Zhedong White goose, Sichuan white goose and Tianfu goose genome, respectively, please illustrate reason why the gene number were different the results from previous studies.

	Response: Thank you for your comments. This is because of the differences of each species itself. By the previous studies, we can see that the number of genes is different among Zhedong White Goose, Sichuan White Goose and Tianfu Goose. The genome size of the Lion-head Goose in our present study is even larger than the former three, so it is normal to have a higher number of genes. During the process, we have performed TE filtering on the gene annotation.<br><br>Line 27, "…generating 40 pseudochromosomes", the assignment of 40 chromosomes to Hi-C scaffolds is very tentative and needs to be validated, the 40 pseudo-chromosomes do not equate to the 40 physical chromosomes. Moreover, the result is conflict with the 39 pseudochromosomes in Tianfu goose genome, how did the authors confirm the number of chromosomes?<br>Response: Thank you for your comments. According to the analysis results of Hi-C sequencing of spatial genome, there are indeed 40 chromosomes. Others are described in the second part of the first question: The diploid cotton genome researchers used Hi-C technology to anchored and oriented 1,573 Mb of assembly to 13 pseudochromosomes. The chromosome-level genome assembly of scimitar-horned oryx, generated 29 chromosomes using 10X Chromium sequencing and Hi-C technology. The genome of autopolyploid sugarcane Saccharum spontaneum L. was assembled using a Hi-C-based physical map to obtain 32 pseudochromosomes. The Musa Balbisiana Genome constructed a high-throughput chromosomal conformational capture (Hi-C) library with 430 Mb (87.27%) of assembly and 94.0% of genes placed on 11 chromosome groups. The above examples fully demonstrate that the construction of chromosomal level genome by Hi-C is feasible and credible, and it is a widely used assembly method. Most researchers use Hi-C technology to illustrate genomic chromosome number. In this study, the Hi-C method we used to construct chromosomal level genomes is also an assisted assembly method based on Hi-C sequencing results, showing the three-dimensional structural characteristics of goose genomes.<br><br>Line 33, "…an average of 1,520.6 Mb of raw data with detecting 44,858 SNPs". Based on whole-genome resequencing data, researchers have identified 9,279,339 SNPs in the goose genome using an average depth of 12.44× whole genome resequencing data. Referring to SNP number, it is uncertain whether the results in this study (44,858 SNPs generated from 1,5Gb data) is correct. Therefore, the authors should apply the BWA-GATK pipeline to Tianfu meat goose and Lion-head goose for GWAS analysis to determine whether the results are correct.<br>Response: Thank you for your comments. The sequencing data of the Lion-head goose population is from enzyme-based RAD-Seq, and the average sequencing depth is 12.44×. Due to the depth of sequencing coverage, it is normal to detect differences in the number of SNPs. In addition, the genome assembly of Tianfu goose has been completed by Chongqing Academy of Animal Husbandry, and the assembly data can be found on NCBI, but no re-sequencing data of Tianfu goose could be found on NCBI. Therefore, there is currently no way to conduct genome-wide association analysis between Tianfu meat goose and Lion-head goose. Furthermore, the Tianfu goose is a cultivated breed, while the Lion-head goose is a native breed, and a comparative analysis of the two does not seem to yield a relatively accurate result.<br><br>Line 61 to 65, It is recommended to rewrite or replace the descriptions for the goose breeds with methods sections.<br>Response: Thank you for your comments. We refined the phenotypic descriptions of the four goose species.<br><br>Methods section<br>Line 88, Provide a detailed description of the picture(s) for the Lion-head goose to display the "classical trails". Please supply the pictures for the four goose breed (Wuzong goose, Huangzong goose, Magang goose and Lion-head goose) to help the more clear the understanding of design.<br>Response: Thank you for your comments. Due to the limitation of conditions, it is difficult for us to take photos of four goose breed in the same background. Therefore, we use text to describe the characteristics of four goose species in the INTRODUCTION, and show pictures of individual Lion-head goose. The description is as follows: The Lion-head goose has a large body, a deep and wide head, and large sarcomas (five sarcomas) on the front and side of the face. The adult male goose

weighs 9-10 kg and the female goose 7.5-9 kg, grows rapidly and has rich muscles. Wuzong goose is a small goose species with a distinct band of black plumage from neck to back. The gander weighs 3-3.5kg and the female weighs 2.5-3kg, with wide and short body, flat back, and thin and short feet. Magang goose is a medium-sized goose species, with a long head, wide beak, rectangular body, a gray-black bristle-like feathers on the back of the neck, gray brown breast feathers and white belly feathers. Adult weight is 4-5 kg for males and 3-4 kg for females. Huangzong goose has a compact body, from the top of the head to the back of the neck has a brownish yellow feather belt, shaped like a horse's mane. The chest feather is gray yellow, the belly feather is white, the beak and sarcoma are black. Adult males weigh 3-3.5 kg, females 2.5-3 kg. Displayed in L64-74.

Line 91 to 92, "from another four healthy adult accessions were collected for RNA-seq analysis", please rewrite the sentence since it is unclear.
Response: Thank you for your comments. We have modified it as “another four healthy adult individuals”.

Supply the detail information for GWAS analysis, including the software, models What parameters were used to run GATK, plink, BWA? did the authors performed GWAS analysis using plink software, rather than GEMMA, TASSEL or other software ?
Response: Thank you for your suggestions. We have supplemented the relevant software parameters and correlation analysis model, showing in L212-232.

line 200 "the results of the assoc and linear analyses were…", supply the detail of GWAS analysis, including the software, analysis model. please provide more detailed information about the models and assumptions.
Response: Thank you for your suggestions. We have supplemented the relevant data.

What the top 20 PCs? Did the PCs paly an important role in GWAS analysis?
Response: Thank you for your comments. The top 20 PCs are the top 20 principal components after PCA analysis based on genetic variant information, and top 20 PCs were used as covariables for GWAS to reduce the interference of population structure on results.

Detailed information is not given in several parts of this paper, especially the methodology. How many individuals from the four-goose population? The GWAS analysis were performed in one goose population or the four-goose population? How did the authors do the GWAS analysis and annotation the SNPs? please supply detail analysis steps and analysis models, software. For GWAS analysis model, were there any family or environmental effects? how did you test the significance of the random variables? Many sentences are not clear all over the entire manuscript and need to be re-writen. For instance, line 201, "The corresponding genes of significantly related SNPs were used to identify the GO pathway", define the corresponding genes, and how did the GO pathway analysis?
Response: Thank you for your suggestion, we have polished the points you mentioned, please see the manuscript marked yellow.

Line 203, please rewrite the statistical analysis section to provide more detail. For example, authors should define "potential associated" in this section.
Response: Thank you for your comments. Based on the opinions of other reviewers, we deleted this section and integrated relevant information into other analysis section.

Line 283 to 284, "…correlated with any chromosome of the duck genome due to the presence of a large number of tandem repeats". Provide the detail data or the figure(s) to support your claim.
Response: Thank you for your suggestions. We are very sorry that we have described it wrong here. The collinearity analyses use blocks of several genes as basic units to determine whether regions of two genomes are homologous. Therefore, we change to “…were not correlated with any chromosome of the duck genome maybe due to the heterogenous of genes on the chromosome”.

Results section
Compare with the quality metrics of this study with the previous four goose genome, including contig N50, scaffold N50, gene number, Repetitive regions proportion of

	genome, etc.<br>Response: Thank you for your comments, we have added Table 2 to compare the assembled genomes of the four goose species (i.e., Zhedong white goose, Sichuan white goose, Tianfu goose and Lion-head goose).<br><br>For gene annotation, the authors did not perform the none coding RNA in the goose genome, please supply the analysis.<br>Response: Thank you for your comments. In this study, mRNA was used for transcriptome sequencing. Sequencing of the none coding RNA was not performed, so annotation analysis of none coding RNA could not be performed. Your suggestions are very constructive, and we will continue to conduct in-depth studies from the whole transcriptome in the future.<br><br>The author(s) should perform the positive selection genes analysis with the avian chromosome genomes, such as chicken, duck, zebra finch, etc.<br>Response: Thank you for your comments. We have conducted gene family expansion and contraction analysis of some avian species that already reflect the selection of functions in various avian species during evolution. We have done the correlation analysis, and the relevant description is in the Line 285-303 of the article. Described as follows: Moreover, we mixed the gene family sets of several Anatidae varieties (duck, Zhedong white goose, Lion-head goose), and performed expansion and contraction analysis and corresponding GO enrichment analysis. In this task, the GO analysis of expanded gene families suggested the olfactory perception, such as detection of chemical stimulus involved in sensory perception of smell (GO:0050911, p = 6.97×10-8), and odorant-binding (GO:0005549, p = 1.47×10-5), both of which may be related to the adaptation of the species to find food in water. Meanwhile, contracted gene families were concentrated in the areas of glucose synthesis and metabolism, such as hexokinase activity (GO:0004396, p = 7.64×10-26), glucose binding (GO:0005536, p = 2.30×10-22), cellular glucose homeostasis (GO:0001678, p = 6.84×10-18), glycolytic process (GO:0006096, p = 1.75×10-15), hexose metabolic process (GO:0019318, p = 2.66×10-14), carbohydrate phosphorylation (GO:0046835, p = 1.68×10-9), and glucose 6-phosphate metabolic process (GO:0051156, p = 1.27×10-9), which may be closely related to characteristics of glycogen storage and utilization during migration. Besides, 220 unique gene families (other species lack these gene families) of the Lion-head goose were identified and functionally annotated in GO categories, such as protein kinase activity (GO:0004672, p = 6.85×10-9), the regulation of apoptotic process (GO:0042981, p = 5.78×10-34), the adenylate cyclase-modulating G protein-coupled receptor signaling pathway (GO:0007188, p = 5.92×10-3), and fatty-acyl-CoA reductase (alcohol-forming) activity (GO:0080019, p = 8.94×10-5).<br><br>Please supply the detail information of the 40 pseudo-chromosomes for the goose genome assembly.<br>Response: Thank you for your comments. In fact, we have already uploaded the raw data to the GigaSciences as requested, and provided the circos plot with the corresponding data including the GC content of 40 chromosomes, gene abundance and other information. Meanwhile, we have uploaded the data to the submission system, please check.<br><br>Please show the summary of the economic traits used in this study, including the mean, stand error, numbers of individuals, breed, male or female.<br>Response: Thank you for your comments. We have added tables of descriptive statistics for different goose weights, as shown in Table 3.<br><br>line 233-234, "The aggregate of 760 Gb raw reads was accumulated by the paired-end sequencing of the 36 constructed libraries", Why did the authors conduct 760 Gb RNAseq? It is obvious too much larger than previous goose genome annotation, did they perform more analysis?<br>Response: Thank you for your suggestions. We performed RNA-seq to assist genome assembly and increase the credibility of genome annotations. The 760 Gb of data is composed of 4 individuals, 8 tissues and blood for a total of 36 samples combined, and we have uploaded the transcriptome data to NCBI.<br><br>Line 286 to 287, "Chr 4 of Lion-head goose was found to correspond to the sex chromosome Z of duck, except for the inversions of small patches of segments;

therefore, we inferred that Chr 4 was the sex chromosome of the Lion-head goose", To better understand the unique biological characteristics and breeding of geese, it is essential to distinguish the sex chromosomes from the autosomes. For updating the sequence of Z and W chromosomes, it is recommended to filter the sequence of autosomes using experimental methods. How did the authors filter autosomal sequences in the Chr4? Moreover, the W chromosome sequence should be identified similarly to the Z chromosome. Authors should identify the Z and W chromosome sequence from public databases based on the Z and W chromosome sequence from the chromosome-level avian genome.
Response: Thank you for your suggestions. The aim of this study was to construct a complete and accurate genome map of Lion-head goose, and to analyze evolutionary relationship with avian species, and to determine the changes of genome level of different goose species during the selection and breeding process. Refinement analysis and confirmation of sex chromosomes are not the focus, but your suggestion is interesting, and we are curious about it, and we will focus on it in the future. Additionally, this study was used for genome assembly in male Lion-head goose, which have been described at Methods - Animal selection, and males are without W chromosomes.

Line 292-294, "and their weight was recorded, with the Lion-head goose using the minimum weight, the Wuzong goose using the maximum weight, and the Huangzong goose and Magang goose using the average weight." Why did the authors select the body weight trait? The artificial selection would lead to the inaccurate GWAS results.
Response: Thank you for your comments. In this study, we did not make artificial selection. We only selected four domestic geese from Guangdong province of China for the association analysis of body weight and SNP. The difference in body weight was due to the goose species themselves, e.g., Lion-head geese weighing more than 9 kg, the Wuzong geese 1.8-2.5kg, Huangzong geese 2.7-4.3kg, and Magang geese 4.8-5.5kg. These weight ranges were obtained from the samples collected, without any artificial selection involved.

From figure 5A, there are significant population stratification in Lion goose population (obvious clustering 2 clusters), how did the authors sure to provide accurate GWAS results? Did the author detect the SNPs associated with body weight in the goose population to test the accurate of GWAS results? The discussion tends to be mere story telling.
Response: Thank you for your suggestions. The stratification of the Lion-head geese mainly due to gender differences. Using PCA analysis to obtain the top 20 principal components as covariables for GWAS, which can reduce the interference of group structure on the results and eliminate the influence of group stratification on the statistical results of GWAS as much as possible. Based on your suggestions, we have revised the discussion to make it more specific and in-depth.

Tables and Figures
In table 1, the "Hi-C" results is repeat with the "Assembly", please modify it.
Response: Thank you for your suggestions. We have modified.

The table 2-4, Figure 1-2, are not very informative and I suggest moving these to the supplementary information.
Response: Thank you for your suggestions, we have adjusted all the figures and tables.

Reviewer#2: In this manuscript titled "Chromosome-level genome assembly of goose provides insight into the adaptation and growth of local goose breeds", Zhao et al. Based on PacBio, Bionano and Hi-C technologies, they report a chromosome-level genome assembly of Lion-head goose (Anser cygnoides).The assembly had a total genome size of 1.19 Gb, consisting of 1,859 contigs with an N50 length of 20.59 Mb, generating 40 pseudochromosomes, representing 97.27% of the assembled genome, and identifying 21,208 protein-coding genes. To identify genetic markers associated with body weight in different geese breeds including Wuzong goose, Huangzong goose, Magang goose and Lion-head goose, a genome-wide association study was performed, yielding an average of 1,520.6 Mb of raw data with detecting 44,858 SNPs. GWAS showed that six SNPs were significantly associated with body weight and 25 were potentially associated. The writing of the paper is mostly clear, except a few

things I would like to have them clarified (or re-written):
(1)Line 209, "sequencing strategies" should be replaced with "sequencing and genome assemble strategies".
Response: Thank you for your suggestions. We have revised it according to your suggestion.

(2)Line 210, "Hi-C" should be replaced with "Hi-C approach".
Response: Thank you for your suggestions. We have modified it according to your suggestion.

(3)Line 233, "four healthy adult animals" should be replaced with "four healthy adult individuals".
Response: Thank you for your comments. We have modified it according to your suggestion.

(4)Line 243-244, "eight bird species (Lion-head goose, Zhedong white goose, duck, turkey, chicken, pigeon, saker, and titmouse) and green lizard". Please give the very exact Latin name and the article published after the sequencing of the genome of the eight bird species.
Response: Thank you for your comments. We have added the very exact Latin name.

(5) Line 273, gene name (Sterile) should be italic.
Response: Thank you very much for your comments and suggestions. We have revised the above problems and marked them in yellow in the text.

(6) Line 296, "The average raw data was 1,520.60 Mb". What kind of data is this raw data?
Response: Thank you for your comments. We may not have elaborated well in the original manuscript; this kind of data is about the resequencing data of 514 goose blood samples. And we have revised it as follows: Blood from each sample was used for paired-end 100 resequencing. And the average raw data was 1,520.60 Mb. Marked yellow on L327.

(7) Lines 290-306, The logic of paragraph Cluster analysis of different goose species was confused. What is the purpose of this analysis?
Response: Thank you for your comments. This part of the study was conducted by whole-genome resequencing of blood samples from four goose populations to identify genetic variants and demonstrate the great phenotypic differences among the four goose populations by PCA and phylogenetic trees, followed by genome-wide association analysis of SNPs with body weight of different goose species to mine the available functional SNPs.

(8) Line 299, please use uniform scientific notation, 10e-7 should be ×10-7.
Response: Thank you for your comments. We have refined this.

(9) The PCA results in Figure 5A need a statistic test.
Response: Thank you for your comments. The main purpose of PCA analysis is to visualize between- and within-group differences in subgroups, and top PC20 of PCA is also used for statistical analysis of GWAS to reduce the interference of group structure on the results.

(10) Lines 258-271, 310, 317, Pvalue or P? The form in the whole manuscript should remain uniform.
Response: Thank you for your comments. We have performed a full-text check of the Pvalue and unified them as “p”.

Reviewer #3: Zhao et al.
Title: "Chromosome-level genome assembly of goose provides insight into the adaptation and growth of local goose breeds"
- please place figures and tables within the text, not at the end: this makes it difficult to read and review the article (From editors: you can disagree this comment)
- English: needs to be improved
Response: Thank you for your comments. The English of this manuscript has been improved according to your suggestion.

Powered by Editorial Manager® and ProduXion Manager® from Aries Systems Corporation

- Figures: low quality, low resolution --> hard to read. Additionally, Figure legends/captions are separate from Figures --> difficult reading
Response: Thank you for your comments. We have uploaded the high-definition images and put them together with the legends.

Introduction

L44: what do you mean with "the majority of birds"?
Response: Thank you for your comments. It means most birds of Anseriformes, and we have polished it.

L51: warmth retention of the birds?
Response: Thank you for your comments. We changed it to “the warmth properties of feather products”.

L63: " ... while IN the Wuzong goose ... the average weight is ..."
Response: Thank you for your comments. We have modified it.

L67: how is an accurate reference genome essential to decipher the industry's development? Which industry?
Response: Thank you for your comments. We revised it to “improving production efficiency and even promoting the development of goose industry.”

L73: maybe it's new scaffolding techniques
Response: Thank you for your comments. We have modified it.

L80-81: here you mention two sequencing technologies (SMRT, Illumina NGS), one scaffolding method (Hi-C) and one unspecified technology/methods by Bionano (which was not mentioned earlier). Please rephrase and be more specific and clearer
Response: Thank you for your comments. Thank you for your comments. We have described the Bionano optical mapping technology as described below: Bionano optical mapping technology has advantages in obtaining highly repetitive sequences and detecting genomic structural variants, which is helpful for remote sequencing of sequence overlap clusters. Bionano has become a powerful tool for genome assembly, a 5.1 Mbp inversion was found in the genomes of a patient with Duchenne muscular dystrophy. Displayed in L88-91.

L81: correlation of body weight with what?
Response: Thank you for your comments. Correlation of body weight with genetic variations.

Methods

L119-181: how were Bionano maps used to improve the quality of your genome assembly?
Response: Thank you for your comments. This sentence is a general introduction to the whole text, and we describe the relevant methods and parameters after the sentence: “Afterward, the data were assembled with RefAligner and Assembler of BioNano Solve. The scaffold was established using BioNano Solve with HERA’s contigs and a BioNano genome map. When encountering a conflict between a contig and the genome map, the contig was split to correct the false connection”.

L136: adult accessions?
Response: Thank you for your comments. We have changed “adult accessions” to “adult Lion-head goose”.

L142: how were low-quality reads defined? Based on average Phred scores?
Response: Thank you for your comments. Adaptors and low-quality reads of raw data were removed using Trimmomatic and the sequence will be trimmed according to the base quality value (i.e. Phred scores) with the following parameters: LEADING:3 TRAILING:3 SLIDINGWINDOW:4:15 -threads 20 MINLEN:50, and other default.

L143: what do you mean by polluted reads?

Response: Thank you for your comments. Polluted reads are erroneous sequences such as those containing more than 5% of N base due to systematic errors.

L143: what do you mean with "Trinity was arranged"?
Response: Thank you for your comments. We have changed “Trinity was arranged” to “Trinity was used”.

L148-150: poor English, please rephrase. Additionally, more details are needed on the quality metrics used to evaluate the assembled genome
Response: Thank you for your comments. The reference library used for BUSCO evaluation is aves_odb10, and the rest of the parameters are BUSCO default, so no detailed parameters are listed.

L164-165: could you add the scientific names (genus species) of the mentioned avian species? (green lizard is not an avian species)
Response: Thank you for your comments. We have made changes and additions, and the changes are as follows "we compared the gene families of Lion-head goose with the genomes of the following avian species: Zhedong white goose (Anser cygnoides), duck (Anas platyrhynchos), turkey (Meleagris gallopavo), chicken (Gallus gallus), pigeon (Columba livia), saker (Falco cherrug), titmouse (Pseudopodoces humilis), and green lizard (Anolis carolinensis).”

L173: from where did you get the divergence time between turkeys and pigeons? (~100 million years? Really?) And why did you choose this specific divergence value for calibration?
Response: Thank you for your comments. Divergence time data were obtained from the website http://www.timetree.org/. Pigeon and turkey divergence times were chosen as controls because these two species are genetically distant in avian species and have lower correction errors.

L173: the r8s software was served to estimate: bad English
Response: Thank you for your comments. We have changed “served” to “used”.

L179: "Experimental sample processing and genotyping" this is a bit unclear: you already took biological samples, maybe you need to highlight that this is genotyping (your title should be more about genotyping and phenotyping for GWAS, since you spend the first few lines of the paragraph to describe the phenotypes)
Response: Thank you for your comments. We have changed “Experimental sample processing and genotyping” to “Experimental sample processing and variant detection for Genome-wide association study”.

L181-185: body weight is naturally a continuous trait, it would be rather arbitrary to split it into categories: therefore I don't understand this whole bit on categorical vs continuous body weight
Response: Thank you for your comments. This may be due to our choice of species, each species has a wide range of body weight, but basically within a relatively fixed range. The detailed values are as follows: 9-14 kg for Lion-head goose, 2.7-4.3 kg for Huangzong goose, 1.8-2.5 kg for Wuzong goose, and 4.8-5.5 kg for Magang goose. Although there seems to be some stratification, we still consider their weight as a continuous variable.

L186-190: what you describe is RAD-sequencing/GBS/resequencing, not "genotyping". By genotyping usually an array-based approach is meant
Response: Thank you very much for your professional opinion. We have improved it by removing the description of “genotyping” and modifying it as follows: “Then variant detection as well as genotyping was performed using Samtools, GATK4 software”.

L188: how did you define low quality reads here? (Phred scores?) No filters on average reads coverage per site?
Response: Thank you for your comments. Low quality threshold parameters set to 5.

L191: it is not clear which variants were called? SNP? MNP? Indels? All? etc.
Response: Thank you for your comments. It means SNP variants, and we have modified in the manuscript.

	L191: why did you set the MAF threshold at 5%? You have 514 samples, with a filter at MAF 1% you'd still have more than 10 copies of the minor allele in the worst case scenario<br>Response: Thank you for your comments. Because MAF threshold at 5% is a conventional threshold, this is to ensure that this SNP analysis remains consistent with previous and subsequent analyses, and does not introduce additional systematic errors.<br><br>L192: maximum deletion threshold? Is this max missing rate?<br>Response: Thank you for your suggestion, we have modified it.<br><br>L192-193: what was the objective of PCA? PCA on which data? (I guess the genotype data? Which?)<br>Response: Thank you for your comments. In this study, PCA was performed based on SNP variation information to extract the main principal components, and the top principal components were used as dependent variables for genome-wide association analysis.<br><br>L193-194: "To understand the kinship among the samples, and phylogenetic trees were constructed." This sentence seems wrong/incomplete<br>Response: Thank you for your comments. We made the following changes: To understand the kinship among the samples, and the phylogenetic trees were constructed using SNP data with Phylip software.<br><br>L196: maybe you mean genetic variation?<br>Response: Thank you for your suggestion, we have modified it.<br><br>L197: did you use the --linear option in Plink?<br>Response: Thank you for your suggestion, GWAS analysis was done for both the assoc model and the linear model in Plink.<br><br>L197-199: this sentence is poorly written, please rephrase<br>Response: Thank you for your comments. We modified it to “The top 20 PCs in the PCA analysis were used as covariates, and regression analysis was performed on sample variances with corresponding weight information by Plink.”<br><br>L199: I guess its the variants, not the variances (if it is SNPs, please say SNPs)<br>Response: Thank you for your comments. We modified it to “SNPs”.<br><br>L196-200: I think it would be better if you wrote the GWAS model explicitly (the model equation)<br>Response: Thanks to your suggestion, we have added the model to line 212-232.<br><br>L200: why did you choose Bonferroni correction over other methods to control for spurious results (e.g. FDR, Bayesian odds, permutation test, q-values etc.)<br>Response: Thank you for your comments. Because the Bonferroni correction is more rigorous, it is more effective in removing false positive results.<br><br>L202: this part is useless, as it is: which statistical analysis? Why did you choose the 0.05 threshold for significance? (you just said above that you used Bonferroni corrected p-values for GWAS)<br>Response: Thank you for your suggestion, we have removed this section.<br><br>Results<br>-------<br>L211: "Assemble these data step by step and produce progressively improved assemblies (Fig. 1A)." This sentence seems incomplete or wrong<br>Response: Thank you for your comments. We modified it to “We assemble these data step by step and produce generate progressively improved assemblies assembled genome”.
**Additional Information:**	
**Question**	**Response**

Powered by Editorial Manager® and ProduXion Manager® from Aries Systems Corporation

Are you submitting this manuscript to a special series or article collection?	No
**Experimental design and statistics**<br><br>Full details of the experimental design and statistical methods used should be given in the Methods section, as detailed in our Minimum Standards Reporting Checklist. Information essential to interpreting the data presented should be made available in the figure legends.<br><br>Have you included all the information requested in your manuscript?	Yes
**Resources**<br><br>A description of all resources used, including antibodies, cell lines, animals and software tools, with enough information to allow them to be uniquely identified, should be included in the Methods section. Authors are strongly encouraged to cite Research Resource Identifiers (RRIDs) for antibodies, model organisms and tools, where possible.<br><br>Have you included the information requested as detailed in our Minimum Standards Reporting Checklist?	Yes
**Availability of data and materials**<br><br>All datasets and code on which the conclusions of the paper rely must be either included in your submission or deposited in publicly available repositories (where available and ethically appropriate), referencing such data using a unique identifier in the references and in the "Availability of Data and Materials" section of your manuscript.<br><br>Have you have met the above requirement as detailed in our Minimum	Yes

Standards Reporting Checklist?	

Powered by Editorial Manager® and ProduXion Manager® from Aries Systems Corporation

Manuscript

Click here to access/download;Manuscript;renamed_5e4b1.docx

Chromosome-level genome assembly of goose provides insight into the adaptation and growth of local goose breeds

Qiqi Zhao[1,3,5], Junpeng Chen[2], Zi Xie[1,3,5], Jun Wang[4], Keyu Feng[1,3,5], Wencheng Lin[1,3,5], Hongxin Li[1,3,5], Zezhong Hu[1], Weiguo Chen[1,3,5], Feng Chen[1,3], Muhammad Junaid[4], Huanmin Zhang[6], Zhenping Lin[2*], Qingmei Xie[1,3,5*], Xinheng Zhang[1,3,5*]

[1]Heyuan Branch, Guangdong Provincial Laboratory of Lingnan Modern Agricultural Science and technology & Guangdong Provincial Key Lab of Agro-Animal Genomics and Molecular Breeding, College of Animal Science, South China Agricultural University, Guangzhou, Guangdong 510642, China; [2]Shantou Baisha Research Institute of Original Species of Poultry and Stock, Shantou, Guangdong 515000, China; [3]Department of Science and Technology of Guangdong Province, Key Laboratory of Animal Health Aquaculture and Environmental Control, Guangzhou, Guangdong 510642, China; [4]College of Marine Sciences, South China Agricultural University, Guangzhou, Guangdong, 510642, China; [5]Guangdong Engineering Research Center for Vector Vaccine of Animal Virus, Guangzhou, 510642, China and [6]Avian Disease and Oncology Laboratory, Agriculture Research Service, United States Department of Agriculture, East Lansing, MI, 48823, USA

* Correspondence address:

Zhenping Lin, Shantou Baisha Research Institute of Original Species of Poultry and Stock, Shantou, China. E-mail: Linzp02@163.com; Qingmei Xie and Xinheng Zhang, College of Animal Science, South China Agricultural University, Guangzhou, China. E-mails: qmx@scau.edu.cn (Q.X.); xhzhang@scau.edu.cn (X.Z.)

running title: Goose chromosome-level Genome Assembly

Abstract

Backgrouond: *Anatidae* contains numerous waterfowl species with great economic value, but the genetic diversity basis remains insufficiently investigated. Here, we report a chromosome-level genome assembly of Lion-head goose (*Anser cygnoides*), a native breed in South China, through the combination of PacBio, Bionano and Hi-C technologies. **Findings:** The assembly had a total genome size of 1.19 Gb, consisting of 1,859 contigs with an N50 length of 20.59 Mb, generating 40 pseudochromosomes, representing 97.27% of the assembled genome, and identifying 21,208 protein-coding genes. Comparative genomic analysis revealed that geese and ducks diverged approximately 28.42 million years ago, and geese have undergone massive gene family expansion and contraction. To identify genetic markers associated with body weight in different geese breeds including Wuzong goose, Huangzong goose, Magang goose and Lion-head goose, a genome-wide association study was performed, yielding an average of 1,520.6 Mb of raw data with detecting 44,858 SNPs. GWAS showed that six SNPs were significantly associated with body weight and 25 were potentially associated. The significantly associated SNPs were annotated as *LDLRAD4*, *GPR180*, *OR*, enriching in growth factor receptors regulation pathways. **Conclusions:** We present the first chromosome-level assembly of the Lion-head goose genome, which will expand the genomic resources of the *Anatidae* family, providing a basis for adaptation and evolution. Candidate genes significantly associated with different goose breeds may serve to understand the underlying mechanisms of weight differences.

Keywords: Lion-head goose, Genome assembly, Comparative genome, Genome-wide association study

Introduction

The *Anatidae* is a family of the ancient *Aves* class with order *Anseriformes*, containing 43 genuses and 174 species, including most birds of *Anseriformes*, such as ducks, geese, swans, and is the most prominent family of swimming birds [1]. Physical characteristics and features vary significantly among species, making the *Anatidae* family rich in diversity and specificity. *Anatidae* adults are usually herbivores, feeding on a variety of aquatic plants, which are well suited to sustainable production practices thereby reducing competition for human food; and some species are even used for crop weeds and pests control [1, 2]. For a long time, duck and goose feathers have been popular in pillows, quilts and coats [3]. Several species in the genus *Anser* are commercially important and domesticated as poultry because of their meat-producing performance and the warmth properties of feather products. According to archaeological evidence, geese were domesticated around 6,000 years ago near the Mediterranean Sea, and later spread around the world due to human activities [4]. It is widely believed that *Anser cygnoides* is the ancestor of the Chinese goose (*Anser cygnoides domesticus*) with a domestication history of more than 3,000 years [1]. After artificial domestication, the domestic goose has increased its cold tolerance and roughage-resistance, but its wings are degraded and weakened in flight, unable to travel long distances [1]. Egg-laying rate and goslings survival rate are also improved compared to wild swans, and the lifespan is longer [5]. Furthermore, overfeeding can cause foie gras to be at least three-fold larger than the normal size while the goose remains healthy, making the goose a good model to study human liver steatosis [6]. Chinese domestic geese is a natural gene pool containing local breeds of diverse phenotypes, and adult domestic geese from similar region vary greatly in weight [7]. For example, the Lion-head goose in Shantou (116°14'-117°19' E, 23°02'-23°38' N), Guangdong Province, can weigh more than 9 kg, while in the Wuzong goose from Qingyuan (111°55'-113°55' E, 23°31'-25°12' N), Guangdong Province, the average weight is only about 3 kg [8, 9]. The Lion-head goose has a large body, a deep and wide head, and large sarcomas (five sarcomas) on the front and side of the face **(Fig. 1)**. The adult male goose weighs 9-10 kg and the female goose 7.5-9 kg, grows rapidly and has rich muscles. Wuzong goose is a small goose species with a distinct band of black plumage from

neck to back. The gander weighs 3-3.5kg and the female weighs 2.5-3kg, with wide and short body, flat back, and thin and short feet. Magang goose is a medium-sized goose species, with a long head, wide beak, rectangular body, a gray-black bristle-like feathers on the back of the neck, gray brown breast feathers and white belly feathers. Adult weight is 4-5 kg for males and 3-4 kg for females. Huangzong goose has a compact body, from the top of the head to the back of the neck has a brownish yellow feather belt, shaped like a horse's mane. The chest feather is gray yellow, the belly feather is white, the beak and sarcoma is black. Adult males weigh 3-3.5 kg, females 2.5-3 kg. However, the mechanisms for such differences have not been clarified, let alone being resolved at the genomic level. Therefore, a complete, continuous and accurate reference genome is essential, for deciphering genomic diversity, evolutionary and adaptive processes, improving production efficiency and even promoting the development of goose industry.

High-quality genome assembly sequences enable us to comprehensively and scientifically decode the genetic diversity of species, explore disease mechanisms, and understand species evolution. Recently, Pacbio has offered technology that can generate reads several thousand bases in size, and these long reads can span repetitive regions [10]. Although these long reads have a high error rate, they can be integrated with Illumina's short reads to improve sequencing accuracy [11]. In addition, new scaffolding techniques, such as high-throughput chromosome conformation capture (Hi-C), allow the genome to be assembled to the level of whole chromosomes [12]. Pacbio single molecule real-time (SMRT) sequencing technology has been extensively used in the study of human diseases such as tuberculosis and influenza virus [13], as well as in the study of species evolution, such as the centromere of the human Y chromosome [14]. Bionano optical mapping technology has advantages in obtaining highly repetitive sequences and detecting genomic structural variants, which is helpful for remote sequencing of sequence overlap clusters[15]. Bionano has become a powerful tool for genome assembly, a 5.1 Mbp inversion was found in the genomes of a patient with Duchenne muscular dystrophy[16].

In this study, we report the genome assembly at the chromosome level in Lion-head geese for the first time using combined data generated by four advanced technologies, Illumina, SMRT, Bionano, and Hi-C. In addition, we investigated the relationship between body weight and genetic variations in Lion-

head goose, Wuzong goose, Huangzong goose and Magang goose by genome-wide association analysis, trying to identify the genes involved in body weight determination from different species. These will offer valuable resources for facilitating genetic research and the improvement of the species and for studying speciation and evolution in geese.

Methods

Animal selection

An adult healthy purebred male Lion-head goose (*Anser cygnoides*) with classical traits was selected for whole-genome sequencing and conducting *de novo* assembly from Shantou Baisha Research Institute of Original Species of Poultry and Stock. Blood and eight tissues (i.e., brain, pharyngeal pouch, head sarcoma, spleen, liver, chest muscle, kidney, and heart) from another four healthy adult individuals were collected for RNA-seq analysis. All applicable institutional and national guidelines for the care and use of animals were followed. All the animal work in this study was approved by the South China Agricultural University Committee for Animal Experiments (approval ID: SYXK 2019-0136). All the research procedures and animal care activities were conducted based on the principles stated in the National and Institutional Guide for the Care and Use of Laboratory Animals.

Genome survey library construction and sequencing

To survey the genome profile, high-quality genomic DNA was extracted from the blood of the reference individual for whole-genome sequencing using the Qiagen Blood and Cell Culture DNA Midi Kit according to the manufacturer's instructions. For the quality control of purity, concentration, and integrity, we used Qubit 2.0 Fluorometry (Life Technologies, USA), NanoDrop 2000 spectrophotometer (Thermo Scientific), and pulse-field gel electrophoresis (Bio-rad CHEF-DR II), respectively. The following steps used for DNA extraction and quality control were similar. The short paired-end Illumina DNA library was constructed using the Illumina HiSeq system (with the paired-end 350 bp sequencing strategy). After performing the sequencing and obtaining the data, the k-mer analysis of reads for the genome survey was calculated by the Jellyfish program with the default parameters. Additionally, the genome size, heterozygosity ratio, and repeat sequence ratio were calculated with the GenomeScope

tool based on the k-mer frequency of 17.

Genome sequencing and assembly strategies

A 40 kb *de novo* library for SMRT genome sequencing was constructed using the PacBio Sequel Ⅲ platform (Pacific Biosciences, USA). All of these reads were used for contigs assembly. A scalable and accurate long-read assembly tool, Canu (v1.8) [17], was employed to correct and assemble the PacBio reads with the listed parameters (minThreads = 4, genome size = 1200m, minOverlapLength = 700, minReadLength = 1000). The resulting contigs and corrected reads were used as inputs for HERA [18] to fill the gaps and produce longer contigs with default parameters. After that, Illumina paired-end clean data were mapped to the corrected contigs with the Burrows-Wheeler Aligner (BWA) [19], and the results were filtered by Q30 with Samtools (v1.8) [20]. At last, Pilon (v1.22) [21] was used to polish the assembly and enhance the base accuracy of the contigs.

Physical optical genome maps from BioNano were used to improve the assembly quality of the genome, with the ultimate goal of generating a chromosome-scale assembly. Nuclear DNA was extracted from the blood sample of the reference individual and digested with nickase Direct Labeling Enzyme Ⅰ. After labeling, repairing and staining reactions, DNA was loaded onto the Saphyr Chip for sequencing to generate BioNano molecules. Afterward, the data were assembled with RefAligner and Assembler of BioNano Solve. The scaffold was established using BioNano Solve with HERA's contigs and a BioNano genome map. When encountering a conflict between a contig and the genome map, the contig was split to correct the false connection.

For Hi-C library, fresh blood was vacuum-infiltrated with 2% formaldehyde solution and then used for cross-link action. Later nuclear DNA was isolated from the reference animal and digested with the restriction enzyme Mbo I. The Hi-C library with insertion sizes of 350 bp was constructed and sequenced on the Illumina HiSeq X Ten instrument. The Hi-C reads were assigned to the scaffolds by Juicer [22]. The scaffolds were further clustered, ordered, and oriented to the chromosome-level scaffolds by 3D-DNA [23]. Thus, a heatmap of Hi-C chromosomal interaction was created using the HiC-pro software [24].

RNA-Seq and transcripts assembly

RNA-seq was conducted on blood and eight different tissues (i.e., brain, pharyngeal pouch, head sarcoma, spleen, liver, chest muscle, kidney, and heart) from four healthy adult Lion-head goose. Total RNA was extracted from four individuals using the TRIZOL reagent and purified following the manufacturer's protocols. The concentration and quality of the isolated RNA were assessed using the Nanodrop Spectrophotometer, Qubit 2.0 Fluorometry, and the Agilent 2100 bioanalyzer (Agilent Technologies, USA). Libraries construction and sequencing were performed using the Illumina NovaSeq 6000 platform. Raw RNA-seq data with 150 bp paired-end reads were trimmed for quality using Trimmomatic [25]. Thus, the Illumina sequence adaptors were removed, then low-quality reads based on Phred scores and polluted reads containing N > 5% were trimmed, using the following parameters: LEADING:3 TRAILING:3 SLIDINGWINDOW:4:15 -threads 20 MINLEN:50. Furthermore, Trinity [26] was used to *de novo* assemble the data after quality filtering. To remove redundant sequences, CD-HIT [27] was employed to remove highly identical transcript isoforms, retaining only the longest one. After filtering, the RNA-seq reads were mapped to the assembled genome using the default parameters of STAR [28].

Assembly evaluation

Finishing the genome assembly, quality control for the assembly's quality, accuracy, and integrity was predicted by Benchmarking Universal Single-Copy Orthologs (BUSCO, v 3.0), using aves_odb10 as the query [29].

Genome annotation

The genome assembly was annotated by MAKER, mainly including gene annotation and repeat annotation. The detailed pipeline was based on proteins from the Uniprot, the *de novo* assembly of RNA-seq data, and the total proteins of the relative species *Anser cygnoides* [30]. The transposable elements (TE) associated genes that were filtered out by the TEseeker database, and the results were used to conduct functional annotation using InterProScan. The repeat sequencing library was identified and annotated by a combination of LTR-FINDER and RepeatModeler. RepeatMasker and the query species "Chicken" were used to mask the repeats in the assembly, based on the Repbase database and the previous repeat sequence library. Tandem repeats were discovered by the Tandem Repeats Finder [31].

Gene families and phylogenetic analysis

Interspecific syntenic blocks between the Lion-head goose and duck were explored using MCscan [32] after coding sequence alignment by BLASTn. The same method was used for intraspecific collinearity analysis. To gain insight into the gene family evolution of the goose, we compared the gene families of Lion-head goose with the genomes of the following avian species: Zhedong white goose (*Anser cygnoides*), duck (*Anas platyrhynchos*), turkey (*Meleagris gallopavo*), chicken (*Gallus gallus*), pigeon (*Columba livia*), saker (*Falco cherrug*), titmouse (*Pseudopodoces humilis*), and green lizard (*Anolis carolinensis*). Initially, alternative splicing and genes encoding less than 50 amino acids with a proportion of stop codon greater than 20% were filtered; meanwhile, the longest transcript of genes with multiple isoforms was retained to represent the gene. Similarity relationships among the protein sequences of species were aligned by BLASTP algorithm and clustered using OrthoMCL methodology with an expansion coefficient of 1.5 to obtain single- and multiple-copy gene families, and specific gene families of Lion-head goose. The sequences of the single-copy gene families were employed to perform multiple alignments by MUSCLE. Then RAxML [33] was used to construct a phylogenetic tree of nine species, with the green lizard (*Anolis carolinensis*) being designated an outgroup. Taking the divergence time of the pigeon and turkey (92.9Mya, http://www.timetree.org/) as the calibration, the r8s [34] software was used to estimate the divergence time of the species and construct ultrametric trees. After filtering out gene families with gene counts of more than 100 in some individual species, CAFÉ [35] was employed to detect gene families that had undergone expansion or contraction per million years independently along each branch of the phylogenetic tree. Subsequently, a gene ontology (GO) enrichment analysis of gene families was performed using the clusterProfiler package in R [36].

Experimental sample processing and variant detection for Genome-wide association study

Blood samples of 514 geese (including Lion-head goose, Wuzong goose, Huangzong goose and Magang goose) were collected and stored in 2 mL tubes containing ACD anticoagulant for DNA extraction, and the weight of the geese was recorded. DNA was extracted from blood samples using the HiPure Blood DNA Mini Kit (Magenbio, Guangzhou, China). The samples that passed the quality testing were

subjected to library construction using Easy DNA Library Prep Kit (MGI, Shenzhen, China) and paired-end 100 sequencing using MGIseq 500. Raw data were filtered for adaptors and low quality reads using SOAPnuke software, low quality threshold parameters set to 5, and the filtered sequences were compared with the constructed goose reference genome using BWA software with parameters: mem, -M. Then variant detection was performed using Samtools, GATK4 software with parameters: HaplotypeCaller –ERC GVCF. SNP variants were filtered based on a minimum allele frequency threshold of 0.05, a Hardy Weinberg equilibrium test significance threshold of 10^{-7}, and a max missing rate threshold of 0.7. Principal component analysis (PCA) was performed and plotted with R. To understand the kinship among the samples, the phylogenetic trees were constructed using SNP data with Phylip software.

Genome-wide association study

The genetic variation was analyzed with individualcorresponding weight information using the asymptotic Wald test (assoc) in Plink. The top 20 PCs in PCA analysis were used as covariates, and linear analysis was performed on sample variances with corresponding weight information by Plink.And the common parameters in two types of model analysis is --allow-extra-chr --allow-no-sex -out, where the assoc parameter is -assoc and the linear parameter is --linear --covar plink.eigenvec. The statistical analysis model for genome-wide association analysis was as follows:

$$P = \mu + Z\alpha + SNP + e$$

where P is the phenotypic variable; μ is the intercept; Z is the random multigene effect relationship matrix; α is the random multigene effect; SNP is the SNP effect; e is the residual, distributed as e~(0, I σ_e), and I is the unit matrix.

Genome-wide 5% significance threshold was determined using the Bonferrorni method. To reduce false negative, the threshold was expanded by 20-fold as a second threshold and the SNP in this region was defined as potentially associated. The SNPs with Bonferroni corrected p-values less than 0.05 in the results of the assoc and linear analyses were annotated. The corresponding genes annotated with significantly related SNPs were used to identify the GO pathway.

Selective-sweep analysis

To analyze regions affected by long-term selection and are associated with domestication of geese, we calculated the Fixation indices (F_{ST}) for four goose species using vcftools software with sliding windows length of 20 kb that had a 10-kb overlap between adjacent windows. The top 5% of regions were designated as candidate selective regions and the genes in these regions were considered as candidate genes.

Results

Genome sequencing and assembly

The Lion-head goose is a famous local variety in China and one of the most giant goose breeds worldwide, with a unique appearance and social benefits. Here, we attempt to construct a highly continuous chromosome-scale genome of an adult purebred male Lion-head goose with a high degree of homozygosity to minimize heterozygous alleles. The following sequencing and genome assemble strategies were applied: Illumina sequencing, Pacbio SMRT sequencing, BioNano optical mapping, and Hi-C approach **(Supplementary Table S1)**. We assemble these data step by step and generate progressively improved assembled genome **(Supplementary Figure S1)**. A total of 185.37 Gb of high-quality Pacbio long reads were generated, representing a ~168× depth of the estimated 1.05 Gb genome with heterozygosity of 0.335% based on the k-mer analysis of the Illumina sequences **(Supplementary Figure S1, Supplementary Table S2)**. Combing the *de novo* assembly of the Illumina and Pacbio sequences resulted in a draft genome of 1.20 Gb, yielding 1,859 contigs with a length of 13.7 Mb for contig N50 and 57.6 Mb for the longest **(Table 1)**. Furthermore, with the help of BioNano optical mapping, the scaffold N50 value was increased to 37 Mb. To obtain a chromosome-scale assembly, a set of ~230 Gb Hi-C data was used to orient, order, phase, and anchor the contigs. Approximately 97.27% of the reads assembled were anchored to 40 high-confidence pseudo-chromosomes (39 autosomes and Z chromosome) using the high-density genetic map **(Supplementary Figure S1, Fig. 2)**. After polishing, we finally assembled the ultimate genome into 1.19 Gb with the final contig N50 of 20.59 Mb and scaffold N50 of 25.8 Mb, with a GC content of 42.39% **(Supplementary Table S2 and S3)**. The structure and quality of the assembled genome were determined by mapping a Hi-C chromosomal

contact map.

The completeness of the Lion-head goose genome assembly was assessed using the BUSCO gene set. The result showed that almost 99.02% of the reads were correctly mapped to the genome. We then evaluated the assembled genome with 98.24% single-copy and 1.76% duplicated orthologs from the BUSCO dataset, confirming that 8,081 genes (96.92%) were intact in this genome. These results indicate the high reliability and integrity of the assembled genome **(Supplementary Figure S2 and Table S4)**.

Genome annotation

To support the genome annotation, we conducted RNA-Seq analysis using RNA samples of blood and eight tissues (brain, pharyngeal pouch, head sarcoma, spleen, liver, chest muscle, kidney, and heart) from four healthy adult individuals. The aggregate of 760 Gb raw reads was accumulated by the paired-end sequencing of the 36 constructed libraries. After filtering the adaptor and low-quality sequences, 723 Gb qualified Illumina reads remained, *de novo* assembled into unique transcripts (unigenes). Overall, a total of 216,229 unigenes were assembled and at the level N50, 5,082 nucleotides were obtained. Total 21,208 protein-coding gene annotations were predicted in Lion-head goose by combining *de novo* prediction, homologous protein prediction, and transcription alignment. After filtering TE-related genes, a total of 21,010 protein-coding gene annotations were finally obtained by the TE seeker database **(Fig. 2)**. Furthermore, a total of 8.15% repeat sequence and 4.10% tandem repeats of the genome were detected **(Table 1)**. Comparative statistics of genome quality metrics with the assembled goose genome (including Zhedong white goose, Sichuan white goose and Tianfu goose) are shown in **Table 2**.

Phylogenetic analysis

To investigate the genomic evolution of poultry, we compared the sequences of eight bird species (Lion-head goose, Zhedong white goose, duck, turkey, chicken, pigeon, saker, and titmouse) and green lizard, clustering the genes into 15,162 gene families **(Fig. 3A, Supplementary Table S5)**. Among these, 6,422 single-copy gene families were identified and used to construct a phylogenetic tree **(Fig. 3B)**. This revealed that the geese and ducks were clustered into a subclade that probably evolved from a common ancestor approximately 28.42 million years ago (Mya). As expected, the Lion-head goose displayed a

close relationship with the Zhedong white goose. The divergence time between the Lion-head goose and Zhedong white goose was estimated to be 13.79 Mya, and that between chicken and turkey was nearly 25.07 Mya. The above results confirmed the reliability of the tree.

Of all the gene families in the Lion-head goose, 4,233 gene families were significantly expanded and 324 were contracted. Compared with Zhedong white goose, the Lion-head goose had more gene families and there are also more events of gene family expansion and contraction. Moreover, we mixed the gene family sets of several *Anatidae* varieties (duck, Zhedong white goose, Lion-head goose), and performed expansion and contraction analysis and corresponding GO enrichment analysis. In this task, the GO analysis of expanded gene families suggested the olfactory perception, such as detection of chemical stimulus involved in sensory perception of smell (GO:0050911, $p = 6.97\times10^{-8}$), and odorant-binding (GO:0005549, $p = 1.47\times10^{-5}$), both of which may be related to the adaptation of the species to find food in water **(Fig. 4A, Supplementary Table S6)**. Meanwhile, contracted gene families were concentrated in the areas of glucose synthesis and metabolism, such as hexokinase activity (GO:0004396, $p = 7.64\times10^{-26}$), glucose binding (GO:0005536, $p = 2.30\times10^{-22}$), cellular glucose homeostasis (GO:0001678, $p = 6.84\times10^{-18}$), glycolytic process (GO:0006096, $p = 1.75\times10^{-15}$), hexose metabolic process (GO:0019318, $p = 2.66\times10^{-14}$), carbohydrate phosphorylation (GO:0046835, $p = 1.68\times10^{-9}$), and glucose 6-phosphate metabolic process (GO:0051156, $p = 1.27\times10^{-9}$), which may be closely related to characteristics of glycogen storage and utilization during migration **(Fig. 4B, Supplementary Table S7)**. Besides, 220 unique gene families (other species lack these gene families) of the Lion-head goose were identified and functionally annotated in GO categories, such as protein kinase activity (GO:0004672, $p = 6.85\times10^{-9}$), the regulation of apoptotic process (GO:0042981, $p = 5.78\times10^{-34}$), the adenylate cyclase-modulating G protein-coupled receptor signaling pathway (GO:0007188, $p = 5.92\times10^{-3}$), and fatty-acyl-CoA reductase (alcohol-forming) activity (GO:0080019, $p = 8.94\times10^{-5}$, **Fig. 4C, Supplementary Table S8**). Interestingly, we annotated a reproduction-related protein in the species-specific gene family, *Sterile* (Pfam ID: PF03015), acting on fatty-acyl-CoA reductase (alcohol-forming) activity, which may be related to the low reproductive rate caused by congenital infertility in geese.

Collinearity analysis allows one to judge molecular evolutionary events between species and explain

the structural differences between the two genomes. We identified synteny blocks among avian genomes and found high collinearity between our assembly and the duck genome (genome size =1.19 Gb). Here, multiple chromosomes (Chr 1-5, 10, 12, 15, 17-20, 23, 26, 27, 29, 30, 32, 34, 36, 37, 39) of Lion-head goose were almost one-to-one collinear with those of the duck, but some chromosomal rearrangements occurred **(Fig. 3C, Supplementary Figure S3)**. For example, on some chromosomes like Chr 1, 2, 3, and 4 of the duck genome, genes break and rearrange on the Lion-head goose genome, resulting in sequential inversion. In addition, some scaffolds such as Chr 9, 24, 25, 31, 35, 38 and 40, were not correlated with any chromosome of the duck genome maybe due to the different sources of genes on the chromosome. These results indicate that chromosome inversion and interchromosomal recombination may have occurred specifically in Lion-head goose during the evolutionary process, but this requires further investigation and verification. Moreover, Chr 4 of Lion-head goose was found to correspond to the sex chromosome Z of duck, except for the inversions of small patches of segments; therefore, we inferred that Chr 4 was the sex chromosome of the Lion-head goose. This information will be fundamental for comparative genomic studies in *Anatidae* animals.

Cluster analysis of different goose species population

Blood samples were collected from 514 geese (including Lion-head goose, Wuzong goose, Huangzong goose and Magang goose), and their weight was recorded, with the Lion-head goose using the minimum weight, the Wuzong goose using the maximum weight, and the Huangzong goose and Magang goose using the average weight. That is, the Lion-head goose weighed at least 9 kg, the Wuzong goose weighed at most 2.5 kg, the Huangzong goose weighed about 3-4 kg, and the Magang goose weighed 4.8-5.5 kg (**Table 6**). Blood from each sample was used for paired-end 100 resequencing. And the average raw data was 1,520.60 Mb, the average sequencing depth was 12.05×, the average coverage was 7.56%, the average matching rate was 91.31%, and 44,858 SNP loci were retained for subsequent analysis after screening SNPs with minimum allele frequency <5%, Hardy-Weinberg equilibrium test significance threshold of 10^{-7}, and maximum deletion rate threshold of 0.7. We reconstructed the goose population structure using SNP data, revealing four distinct subpopulations. The PCA results demonstrated that the Lion-head Goose population was clearly distinguishable from the Magang Goose, Wuzong Goose and

Huangzong Goose, and there was a clear differentiation within the species (**Fig. 5A**). The clustering of Magang Goose and Huangzong Goose was closer together, probably related to their closer geographical location and the existence of some genetic exchange. The phylogenetic tree results were consistent with the PCA results. The clustering of Magang Goose and Huangzong Goose was closer to each other, and they clustered into one branch with Wuzong Goose (**Fig. 5B**).

Candidate genomic regions for body weight based on combined analyses of GWAS and selective-sweep

The Lion-head Goose, Huangzong Goose, Magang Goose, and Wuzong Goose are all local species in Guangdong, but they differ greatly in body weight. In this study, we sought to reveal genomic changes associated with body weight in the four goose species and screen genomic regions and genes. Selective sweep analysis was performed based on the F_{ST} index, considering the top 5% window as candidate regions. And 979 selective regions containing 818 genes were detected.

We then combined the GWAS results with the detected selective features to screen for candidate genomic regions responsible for the differences in goose weight. From the Manhattan plot (**Fig. 5C**), a total of 10 significant signals were found to be associated with body weight trait in geese at the genome-wide level, including one significant SNP detected on Chr 2, 8, 9, and 33 respectively (-log (*p*) > 7.30), and six significant SNPs annotated by two genes on Chr 22, with the closest Manhattan plot SNP peak on Chr 9 for the gene *OR* (Olfactory receptor). Six significant SNPs on Chr 22 are located between 1,992,485 and 1,992,520 bp, a region that spans only a physical distance of 35 bp but contains six SNP loci, making it necessary to analyze these SNPs in this small region in detail to determine whether multiple QTL are involved. The most significant SNP in this region could explain about 8.19% of the phenotypic variation. Apart from significant SNPs, potentially significant QTLs were detected on many chromosomes (including Chr 2, 3, 6, 7, 10, 11, 15, 16, 20, 28, 30, 32, 36), with a total of 25 implied significant SNPs (4.90< -log (*p*) <7.30). On Chr 30, the suggestively significant SNPs were located between 1,258,517 and 2,422,666 bp, spanning approximately 1.16 Mb, with the most significant SNPs in this region explaining approximately 6.12% of the phenotypic variation (**Table 4**). In the present study, we identified genes in the region near the significant SNPs, annotating a total of 21 genes. These genes

may be important in mediating growth and development, and we inference that the *LDLRAD4* gene may play a key role in developmental plasticity in geese, while the *GPR180* gene may regulate the locomotor behavior of geese to make them stronger (**Fig. 6**). GWAS peaks overlapped with genomic regions with selective features on some chromosomes (**Supplementary Data**). This suggests that the region carrying QTL are not only associated with body weight in GWAS, but are also under selection during domestication.

Discussion

Despite the importance of the genus *Anser*, an economically important animal, the relative scarcity of genomic resources has largely hindered progress in studying genome evolution and molecular breeding in the major animals. High-quality chromosome-level genomes can provide key resources for studying. This study describes a chromosome-scale assembly of Lion-head goose obtained by a combination of data from the Illumina, SMRT, BioNano, and Hi-C platforms. The genome assembly is 1.19 Gb in length, and more than 97.27% of the assembled genome is anchored on 40 pseudo-chromosomes. The BUSCO assessment revealed 99.02% complete genes in the assembled genome, making it a better-continuity and higher-quality genome assembly than the recently published Tianfu goose genome with a contig N50 of 1.85 Mb and scaffold N50 of 33.12 Mb [37]. Compared with the cultivated breed Tianfu goose, Lion-head goose, a traditional native breed, should occupy a more prominent position in the germplasm resources, and its evolving message can provide a reference for other local breeds which is worthy of in-depth study.

Comparative genomics is the analysis of the structural characteristics of multiple individual genomes of a species or genomes of multiple species to find out the similarities and differences of gene sequences of species with the help of bioinformatics, and then to study the gene family analysis, analyze the differentiation and evolution of species, to provide a basis for elucidating species evolution. In this study, the evolutionary events of the Lion-head goose were analyzed by comparing the genome sequences with those of other birds. The results showed that the Lion-head goose and Zhedong White goose were most closely related, diverging at about 13.8 Mya, while the geese and ducks diverged at 28.4 Mya. The

results were similar to those of Zhedong White goose, Sichuan White goose and Tianfu goose, indicating the accuracy of the assembly result of this study. Comparative genomic analysis revealed the genetic basis of interesting characters, which helped elucidate important biological implications and obtain solutions for genomic evolution between Lion-head geese and other species of *Anatidae* family, facilitating future genetic breeding programs. This is the first chromosomal level reference genome of Lion-head goose, providing important genomic data for the study of the family *Anatidae*.

The genomic information of the species population was obtained by whole-genome resequencing, and a large amount of variation information was obtained by comparison with the reference genome. Based on the correlation between differences in variation information and phenotypic differences of individuals, the adaptation of species to the environment, scanning of variant loci associated with important traits at the genome level, and localization of genetic mutations were discussed. Lion head goose, Magang goose, Huangzong goose and Wuzong goose are the main breeds of geese in Guangdong Province. Although they all belong to Guangdong Province, the body weight of adult geese varies greatly, and the molecular mechanism causing the huge difference is still unclear. In this study, four goose species were resequenced and examined for variation. Principal component analysis and phylogenetic tree analysis revealed significant differences among several goose species, indicating the feasibility of this study. Subsequently, GWAS was used to identify the candidate functional SNPs that might cause the weight difference of the four goose species, and the genes such as LDLRAD4, GPR180, and OR were analyzed and annotated, attributed to play an important role in mediating growth and development. Recently, there have been several studies related to agricultural traits that have achieved success in animal GWAS projects, for example, GWAS for improving reproductive performance and egg quality in geese and *TMEM161A* gene for embryo development [38]. Genome-wide association analysis of the early-lactation milk fat content in 3,513 Fleckvieh bulls and 2327 Holstein bulls detected 6 associated QTL regions, two of which were located near the gene DGAT1 [39]. GWAS was conducted on 225 ducks with different-sized black spots, and the results showed that EDNRB2 was the gene responsible for the variation in duck body surface spot size [40]. In this study, *LDLRAD4* (low-

density lipoprotein receptor class A domain containing 4), *OR* (Olfactory receptor), and *GPR180* (G protein-coupled receptor 180) were mainly found to function in body weight traits. Knockdown of *LDLRAD4* enhances transforming growth factor (TGF)-β-induced cell migration, which in turn regulates cell growth, differentiation, motility, apoptosis and matrix protein production [41]. The olfactory receptor (*OR2AT4*) has been shown to stimulate the proliferation of keratin-forming cells in peripheral human tissues [42]. *GPR180*, a component of the TGF-β signaling pathway, also has metabolic relevance in the body and may play an essential role in regulating adipose tissue and systemic energy metabolism [43]. Here we found some correlation between these genes and the TGF-β signaling, presumably this pathway also acts on body weight. Identifying of molecular genetic markers and the main effect QTL associated with critical agricultural traits is of great interest to breeders. Nevertheless, the candidate genes identified in this study were only detected by sequencing data and not experimentally validated. The functions of these candidate SNPs and gene markers need to be further verified by experimental results or other techniques. Thus, the findings in our GWAS study represent a valuable resource for geese and provide a new opportunity and basis for geneticists and breeders to work together to explore the genetics behind various agricultural traits.

Conclusions

In summary, we have obtained a high-quality chromosome-scale draft assembly of a purebred Lion-head goose, which provides a genetic basis for understanding the acquisition of related traits and facilitates advances in goose genomics and genetic improvement. Moreover, the candidate genes and their variants identified in this study will help clarify our understanding of goose selective breeding and the development of new breeds. The obtained genome sequence of Lion-head goose is a vital addition to the genome of genus *Anser* and is valuable for further understanding goose molecular breeding strategies. This genomic resource is also of high value for evolutionary studies of closely related species.

Data Availability

The final genome assembly data supporting the results of this article is available in the NCBI BioProject repository, [Accession number: PRJNA736831]. The RNA assembly data is available in the NCBI

BioProject repository, [Accession number: PRJNA807796]. The raw re-sequencing genome data supporting of the GWAS study is available in the NCBI BioProject repository [Accession number: PRJNA552198, PRJNA552383, and PRJNA552384].

Additional Files

Supplementary Figure S1. Sequencing process and presentation.

Supplementary Figure S2. BUSCO assessment of the assembly genome of Lion-head goose.

Supplementary Figure S3. Gene synteny between the Lion-head goose and duck genomes.

Supplementary Table S1. Statistics of sequenced clean data.

Supplementary Table S2. Statistics of genome survey.

Supplementary Table S3. Statistics of genome assembly quality.

Supplementary Table S4. Summary of BUSCOs genome evaluation.

Supplementary Table S5: Summary of gene families from several species.

Supplementary Table S6. GO annotation of expanded gene families from Anatidae varieties (Duck, Zhedong white goose, Lion-head goose; Top 20).

Supplementary Table S7. GO annotation of contraction gene families from Anatidae varieties (Duck, Zhedong white goose, Lion-head goose; Top 20).

Supplementary Table S8. GO annotation of unique gene families from the Lion-head goose.

Supplementary Data. Significant information of selective-sweep analysis.

Abbreviations

BLAST: Basic Local Alignment Search Tool; BWA: Burrows-Wheeler Aligner; BUSCO: Benchmarking Universal Single-Copy Orthologs; Chr: chromosome; GATK4: Genome Analysis Toolkit 4; Gb: gigabase pairs; GO: gene ontology; GPR180: G protein-coupled receptor 180; GWAS: genome-wide association study; HERA: Highly Efficient Repeat Assembly; Hi-C: high-throughput chromosome conformation capture; Kb: kilobase pairs; kg: kilogram; LDLRAD4: low-density lipoprotein receptor class A domain containing 4; LTR: long terminal repeat; Mb: megabase pairs; Mya: million years ago; NCBI: National Center for Biotechnology Information; OR: Olfactory receptor; OR2AT4: olfactory

receptor family 2 subfamily AT member 4; PacBio: Pacific Biosciences; PCA: Principal component analysis; QTL: quantitative trait locus; RAxML: Randomized Axelerated Maximum Likelihood; RNA-seq: RNA sequencing; SMRT: single molecule real-time; SNP: single-nucleotide polymorphism; STAR: Spliced Transcripts Alignment to a Reference; TE: transposable element; TGF: transforming growth factor; TMEM161A: Transmembrane protein 161A.

Competing Interests

The authors declare that they have no conflict of interest.

Funding

This work was supported by the Key Research and Development Program of Guangdong Province (2020B020222001), the Construction of Modern Agricultural Science and Technology Innovation Alliance in Guangdong Province (2021KJ128, 2020KJ128), the National Modern Agricultural Industry Science and Technology Innovation Center in Guangzhou (2018kczx01), the Guangdong Provincial Promotion Project on Preservation and Utilization of Local Breed of Livestock and Poultry (4000-F18260), the Guangdong Basic and Applied Basic Research Foundation (2019A1515012006). The authors would like to thank the BGI in Shenzhen for their work on genome sequencing. We also thank the staff of Minglead Gene for providing the technical and computing support during the research.

Author’s Contributions

Q.X., Z.L., and X.Z. conceived and designed the research. X.Z., J.C., and Q.Z. coordinated the project. J.C. and Z.L. provided animal samples. Q.Z. and Z. X. collected and prepared the samples. Q.Z. performed sequencing, assembly and bioinformatics analysis. W.L., and F.C. led work identifying genes, and H.L., W.C. aided with many aspects of gene identification and did the GO analyses. Q.Z., X.Z. wrote and revised the manuscript and the supplementary information. J.W., M.J., Z.H., H.Z., Z.L., and Q.X. participated in discussions and provided valuable advice. All authors read and approved the manuscript.

References

1. Hoyo JD, Elliott A, Sargatal J, et al. Handbook of the birds of the world. Barcelona: Lynx Edicions; 1992.
2. Madsen J, Marcussen LK, Knudsen N, et al. Does intensive goose grazing affect breeding waders? Ecol Evol 2019;**9**(24):14512-14522. doi:10.1002/ece3.5923.
3. Wang Y, Li SM, Huang J, et al. Mutations of TYR and MITF Genes are Associated with Plumage Colour Phenotypes in Geese. Asian-Australas J Anim Sci 2014;**27**(6):778-83. doi:10.5713/ajas.2013.13350.
4. Gao G, Zhao X, Li Q, et al. Genome and metagenome analyses reveal adaptive evolution of the host and interaction with the gut microbiota in the goose. Sci Rep 2016;**6**:32961. doi:10.1038/srep32961.
5. Yao Y, Yang YZ, Gu TT, et al. Comparison of the broody behavior characteristics of different breeds of geese. Poult Sci 2019;**98**(11):5226-5233. doi:10.3382/ps/pez366.

6. Lu L, Chen Y, Wang Z, et al. The goose genome sequence leads to insights into the evolution of waterfowl and susceptibility to fatty liver. Genome Biol 2015;**16**:89. doi:10.1186/s13059-015-0652-y.
7. Li HF, Zhu WQ, Chen KW, et al. Two maternal origins of Chinese domestic goose. Poult Sci 2011;**90**(12):2705-10. doi:10.3382/ps.2011-01425.
8. Tang J, Shen X, Ouyang H, et al. Transcriptome analysis of pituitary gland revealed candidate genes and gene networks regulating the growth and development in goose. Anim Biotechnol 2020:1-11. doi:10.1080/10495398.2020.1801457.
9. Zhang X, Wang J, Li X, et al. Transcriptomic investigation of embryonic pectoral muscle reveals increased myogenic processes in Shitou geese compared to Wuzong geese. Br Poult Sci 2021;**62**(5):650-657. doi:10.1080/00071668.2021.1912292.
10. Ardui S, Ameur A, Vermeesch JR, et al. Single molecule real-time (SMRT) sequencing comes of age: applications and utilities for medical diagnostics. Nucleic Acids Res 2018;**46**(5):2159-2168. doi:10.1093/nar/gky066.
11. Yoshinaga Y, Daum C, He G, et al. Genome Sequencing. Methods Mol Biol 2018;**1775**:37-52. doi:10.1007/978-1-4939-7804-5_4.
12. Kong S, Zhang Y. Deciphering Hi-C: from 3D genome to function. Cell Biol Toxicol 2019;**35**(1):15-32. doi:10.1007/s10565-018-09456-2.
13. Nakano K, Shiroma A, Shimoji M, et al. Advantages of genome sequencing by long-read sequencer using SMRT technology in medical area. Hum Cell 2017;**30**(3):149-161. doi:10.1007/s13577-017-0168-8.
14. Jain M, Olsen HE, Turner DJ, et al. Linear assembly of a human centromere on the Y chromosome. Nat Biotechnol 2018;**36**(4):321-323. doi:10.1038/nbt.4109.
15. Sun L, Gao T, Wang F, et al. Chromosome-level genome assembly of a cyprinid fish Onychostoma macrolepis by integration of nanopore sequencing, Bionano and Hi-C technology. Mol Ecol Resour 2020;**20**(5):1361-1371. doi:10.1111/1755-0998.13190.
16. Bocklandt S, Hastie A, Cao H. Bionano Genome Mapping: High-Throughput, Ultra-Long Molecule Genome Analysis System for Precision Genome Assembly and Haploid-Resolved Structural Variation Discovery. Adv Exp Med Biol 2019;**1129**:97-118. doi:10.1007/978-981-13-6037-4_7.
17. Koren S, Walenz BP, Berlin K, et al. Canu: scalable and accurate long-read assembly via adaptive k-mer weighting and repeat separation. Genome Res 2017;**27**(5):722-736. doi:10.1101/gr.215087.116.
18. Du H, Liang C. Assembly of chromosome-scale contigs by efficiently resolving repetitive sequences with long reads. Nat Commun 2019;**10**(1):5360. doi:10.1038/s41467-019-13355-3.
19. Li H, Durbin R. Fast and accurate short read alignment with Burrows-Wheeler transform. Bioinformatics 2009;**25**(14):1754-60. doi:10.1093/bioinformatics/btp324.
20. Li H, Handsaker B, Wysoker A, et al. The Sequence Alignment/Map format and SAMtools. Bioinformatics 2009;**25**(16):2078-9. doi:10.1093/bioinformatics/btp352.
21. Walker BJ, Abeel T, Shea T, et al. Pilon: an integrated tool for comprehensive microbial variant detection and genome assembly improvement. Plos One 2014;**9**(11):e112963. doi:10.1371/journal.pone.0112963.
22. Durand NC, Shamim MS, Machol I, et al. Juicer Provides a One-Click System for Analyzing Loop-Resolution Hi-C Experiments. Cell Syst 2016;**3**(1):95-8. doi:10.1016/j.cels.2016.07.002.
23. Dudchenko O, Batra SS, Omer AD, et al. De novo assembly of the Aedes aegypti genome using Hi-C yields chromosome-length scaffolds. Science 2017;**356**(6333):92-95. doi:10.1126/science.aal3327.
24. Servant N, Varoquaux N, Lajoie BR, et al. HiC-Pro: an optimized and flexible pipeline for Hi-C data processing. Genome Biol 2015;**16**(1). doi:10.1186/s13059-015-0831-x.
25. Bolger AM, Lohse M, Usadel B. Trimmomatic: a flexible trimmer for Illumina sequence data. Bioinformatics 2014;**30**(15):2114-20. doi:10.1093/bioinformatics/btu170.
26. Grabherr MG, Haas BJ, Yassour M, et al. Full-length transcriptome assembly from RNA-Seq data without a reference genome. Nat Biotechnol 2011;**29**(7):644-52. doi:10.1038/nbt.1883.
27. Huang Y, Niu B, Gao Y, et al. CD-HIT Suite: a web server for clustering and comparing biological sequences. Bioinformatics 2010;**26**(5):680-2. doi:10.1093/bioinformatics/btq003.
28. Dobin A, Davis CA, Schlesinger F, et al. STAR: ultrafast universal RNA-seq aligner. Bioinformatics 2013;**29**(1):15-21. doi:10.1093/bioinformatics/bts635.
29. Seppey M, Manni M, Zdobnov EM. BUSCO: Assessing Genome Assembly and Annotation Completeness. Methods Mol Biol 2019;**1962**:227-245. doi:10.1007/978-1-4939-9173-0_14.
30. Lu L, Chen Y, Wang Z, et al. The goose genome sequence leads to insights into the evolution of waterfowl and susceptibility to fatty liver. Genome Biol 2015;**16**:89. doi:10.1186/s13059-015-0652-y.
31. Benson G. Tandem repeats finder: a program to analyze DNA sequences. Nucleic Acids Res 1999;**27**(2):573-80. doi:10.1093/nar/27.2.573.
32. Wang Y, Tang H, Debarry JD, et al. MCScanX: a toolkit for detection and evolutionary analysis of gene

synteny and collinearity. Nucleic Acids Res 2012;**40**(7):e49. doi:10.1093/nar/gkr1293.

33. Stamatakis A. RAxML version 8: a tool for phylogenetic analysis and post-analysis of large phylogenies. Bioinformatics 2014;**30**(9):1312-3. doi:10.1093/bioinformatics/btu033.
34. Sanderson MJ. r8s: inferring absolute rates of molecular evolution and divergence times in the absence of a molecular clock. Bioinformatics 2003;**19**(2):301-2. doi:10.1093/bioinformatics/19.2.301.
35. Han MV, Thomas GW, Lugo-Martinez J, et al. Estimating gene gain and loss rates in the presence of error in genome assembly and annotation using CAFE 3. Mol Biol Evol 2013;**30**(8):1987-97. doi:10.1093/molbev/mst100.
36. Yu G, Wang LG, Han Y, et al. clusterProfiler: an R package for comparing biological themes among gene clusters. Omics 2012;**16**(5):284-7. doi:10.1089/omi.2011.0118.
37. Li Y, Gao G, Lin Y, et al. Pacific Biosciences assembly with Hi-C mapping generates an improved, chromosome-level goose genome. Gigascience 2020;**9**(10). doi:10.1093/gigascience/giaa114.
38. Gao G, Gao D, Zhao X, et al. Genome-Wide Association Study-Based Identification of SNPs and Haplotypes Associated With Goose Reproductive Performance and Egg Quality. Front Genet 2021;**12**:602583. doi:10.3389/fgene.2021.602583.
39. Daetwyler HD, Capitan A, Pausch H, et al. Whole-genome sequencing of 234 bulls facilitates mapping of monogenic and complex traits in cattle. Nat Genet 2014;**46**(8):858-65. doi:10.1038/ng.3034.
40. Xi Y, Xu Q, Huang Q, et al. Genome-wide association analysis reveals that EDNRB2 causes a dose-dependent loss of pigmentation in ducks. Bmc Genomics 2021;**22**(1):381. doi:10.1186/s12864-021-07719-7.
41. Nakano N, Maeyama K, Sakata N, et al. C18 ORF1, a novel negative regulator of transforming growth factor-beta signaling. J Biol Chem 2014;**289**(18):12680-92. doi:10.1074/jbc.M114.558981.
42. Cheret J, Bertolini M, Ponce L, et al. Olfactory receptor OR2AT4 regulates human hair growth. Nat Commun 2018;**9**(1):3624. doi:10.1038/s41467-018-05973-0.
43. Balazova L, Balaz M, Horvath C, et al. GPR180 is a component of TGFbeta signalling that promotes thermogenic adipocyte function and mediates the metabolic effects of the adipocyte-secreted factor CTHRC1. Nat Commun 2021;**12**(1):7144. doi:10.1038/s41467-021-27442-x.

Figure legends

Figure 1. A picture of a male adult Lion-head goose.

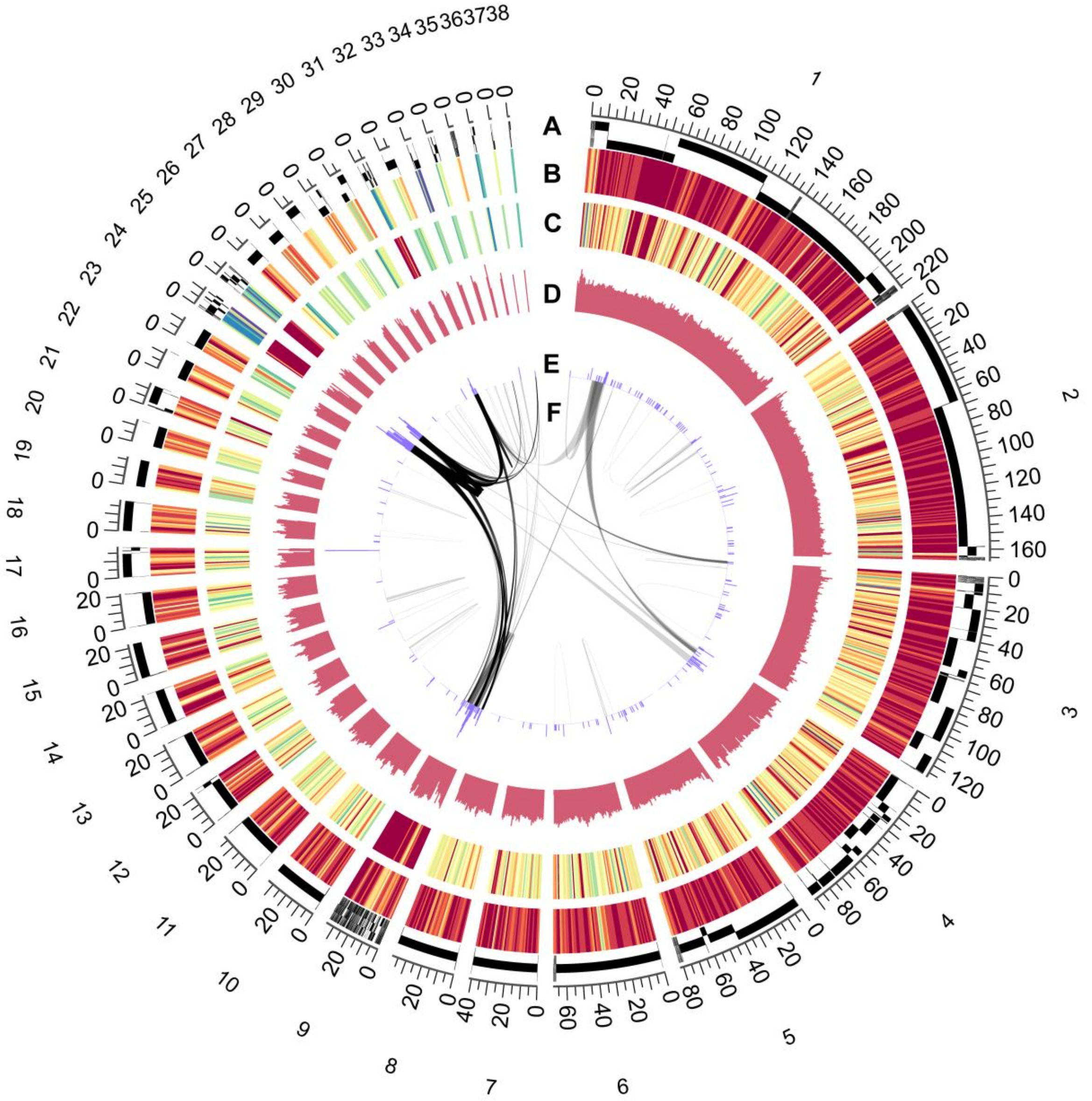

Figure 2. Distribution of genomic features. Concentric circle diagram presents the distribution of genomic features of Lion-head goose using nonoverlapping sliding windows with sizes of 1 Mb (from outmost to innermost). **(A)** the assembled pseudo-chromosome and the corresponding position; **(B)** gene density calculated on the basis of the number of genes; **(C)** average expression level of overall 36 samples. eight tissues (i.e., brain, pharyngeal pouch, head sarcoma, spleen, liver, chest muscle, kidney and heart) and blood collected from four healthy adult animals; **(D)** GC content; **(E)** density of TE; **(F)** gene synteny and collinearity analysis.

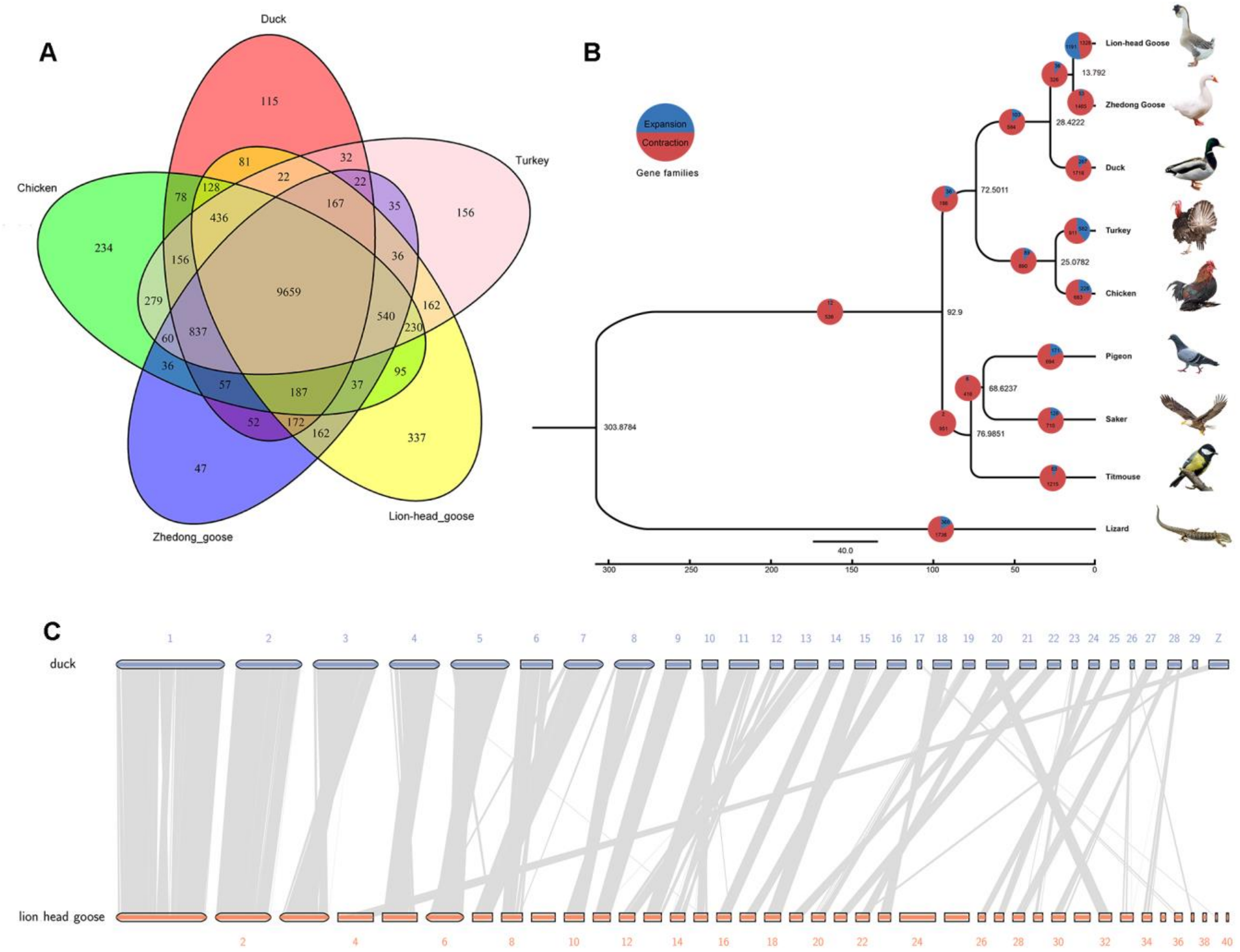

Figure 3. Phylogenetic relationship and comparative genomics analyses. (A) Venn diagram showing the orthologous gene families shared among the genomes of Lion-head goose, Zhedong white goose, chicken, duck, and turkey. **(B)** Phylogenetic tree with the divergence times and history of orthologous gene families. Numbers on the nodes represent divergence times. The numbers of gene families that expanded (green) or contracted (red) in each lineage after speciation are shown on the circles of the corresponding branch. **(C)** Gene comparison of homologous chromosomes between Lion-head goose and duck. Gray lines indicate collinearity between the genomes.

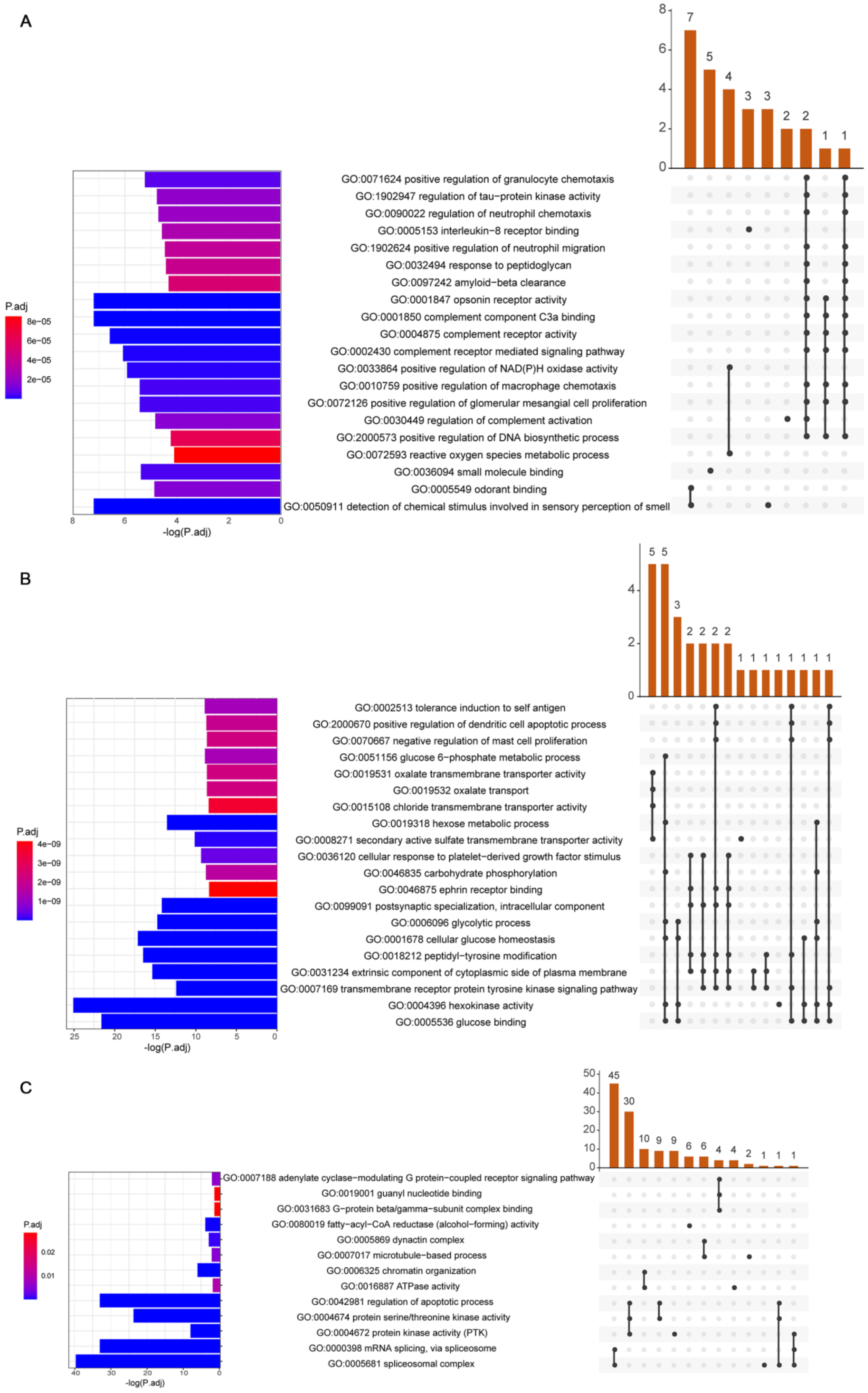
A
P.adj
-log(P.adj)
GO:0071624 positive regulation of granulocyte chemotaxis
GO:1902947 regulation of tau−protein kinase activity
GO:0090022 regulation of neutrophil chemotaxis
GO:0005153 interleukin−8 receptor binding
GO:1902624 positive regulation of neutrophil migration
GO:0032494 response to peptidoglycan
GO:0097242 amyloid−beta clearance
GO:0001847 opsonin receptor activity
GO:0001850 complement component C3a binding
GO:0004875 complement receptor activity
GO:0002430 complement receptor mediated signaling pathway
GO:0033864 positive regulation of NAD(P)H oxidase activity
GO:0010759 positive regulation of macrophage chemotaxis
GO:0072126 positive regulation of glomerular mesangial cell proliferation
GO:0030449 regulation of complement activation
GO:2000573 positive regulation of DNA biosynthetic process
GO:0072593 reactive oxygen species metabolic process
GO:0036094 small molecule binding
GO:0005549 odorant binding
GO:0050911 detection of chemical stimulus involved in sensory perception of smell
B
P.adj
-log(P.adj)
GO:0002513 tolerance induction to self antigen
GO:2000670 positive regulation of dendritic cell apoptotic process
GO:0070667 negative regulation of mast cell proliferation
GO:0051156 glucose 6−phosphate metabolic process
GO:0019531 oxalate transmembrane transporter activity
GO:0019532 oxalate transport
GO:0015108 chloride transmembrane transporter activity
GO:0019318 hexose metabolic process
GO:0008271 secondary active sulfate transmembrane transporter activity
GO:0036120 cellular response to platelet−derived growth factor stimulus
GO:0046835 carbohydrate phosphorylation
GO:0046875 ephrin receptor binding
GO:0099091 postsynaptic specialization, intracellular component
GO:0006096 glycolytic process
GO:0001678 cellular glucose homeostasis
GO:0018212 peptidyl−tyrosine modification
GO:0031234 extrinsic component of cytoplasmic side of plasma membrane
GO:0007169 transmembrane receptor protein tyrosine kinase signaling pathway
GO:0004396 hexokinase activity
GO:0005536 glucose binding
C
P.adj
-log(P.adj)
GO:0007188 adenylate cyclase−modulating G protein−coupled receptor signaling pathway
GO:0019001 guanyl nucleotide binding
GO:0031683 G−protein beta/gamma−subunit complex binding
GO:0080019 fatty−acyl−CoA reductase (alcohol−forming) activity
GO:0005869 dynactin complex
GO:0007017 microtubule−based process
GO:0006325 chromatin organization
GO:0016887 ATPase activity
GO:0042981 regulation of apoptotic process
GO:0004674 protein serine/threonine kinase activity
GO:0004672 protein kinase activity (PTK)
GO:0000398 mRNA splicing, via spliceosome
GO:0005681 spliceosomal complex

Figure 4. GO enrichment analysis of gene families. (A) Expanded and **(B)** contracted gene families from Anatidae varieties (duck, Zhedong white goose, Lion-head goose). **(C)** Unique gene families from the Lion-head goose. The bar graph on the left represents the P-adjust gradient of GO terms, and the color corresponds to the number on the x-axis (i.e. -log (P.adj)). The bluer the color is, the smaller the P-adjust is, and the more significant it is. The redder the color is, the larger the P-adjust is, and the less significant it is. The upper right bar chart exhibits that several genes act together on the terms below. The lower right chart displays the intersection of the genes of each term; the dots connected by lines represent the intersection of multiple terms; the black dots represent “yes”, and the gray dots represent “no”.

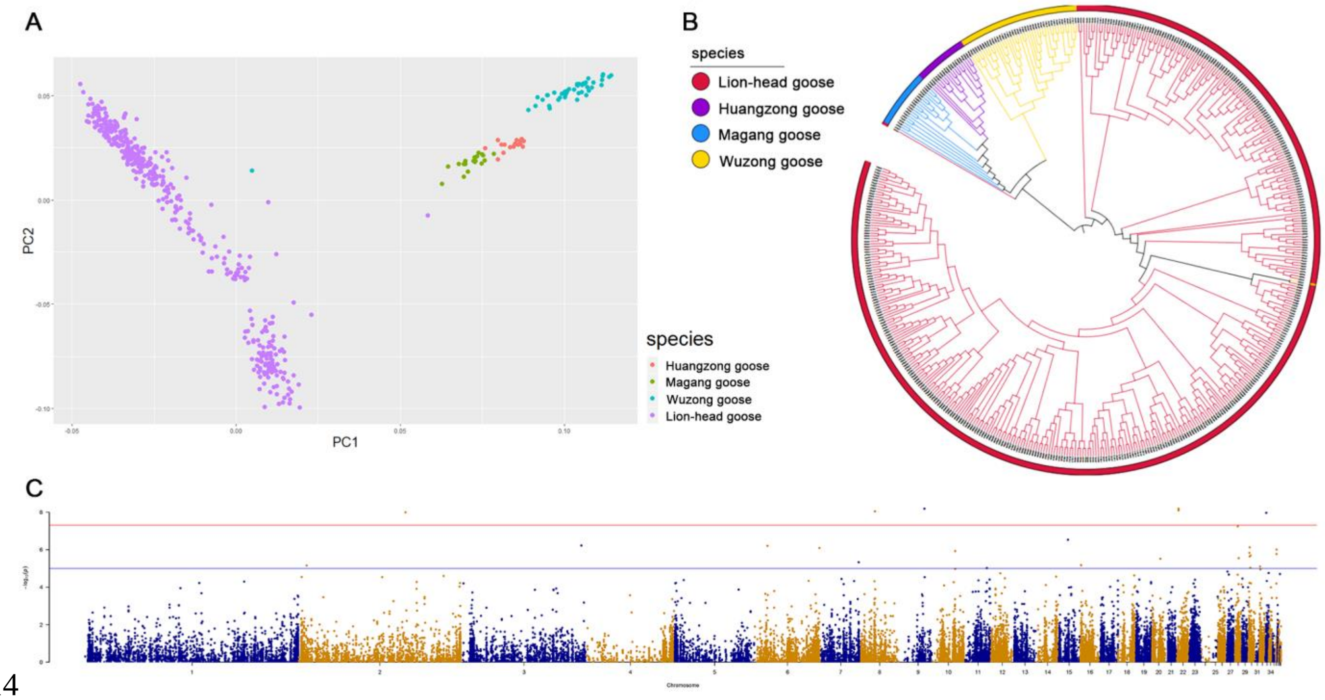

Figure 5. Comparison of different goose species and genome-wide association analysis of body weight. (A) Principal component analysis of sample structures using first two principal components. **(B)** The phylogenetic trees of several goose species. **(C)** Manhattan plot of genome-wide association analysis for body weight. The X-axis indicates chromosomes, and Y-axis indicates the P values of the SNP markers. The red solid line indicates the threshold P value for genome-wide significance. The blue solid line indicates the threshold P value for the significance of potential association.

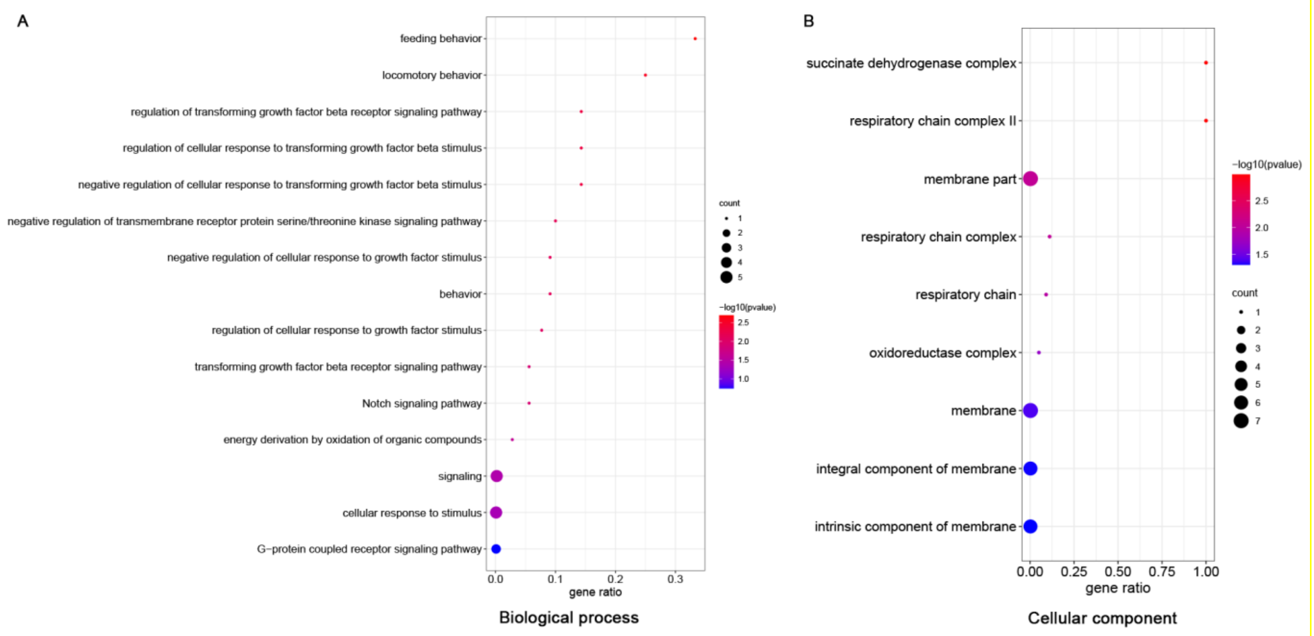

Figure 6. GO analysis of body weight-related genes:(A) Biological processes level, (B) Cellular component level.

Table 1: Summary of repeat classification.

Type	Length	Percent
Long interspersed nuclear element	76,437,757	5.98
Simple sequence repeats	23,026,311	1.80
Low complexity	4,663,288	0.36
Tandem repeats	52,426,380	4.10
Total	156,553,736	12.25

Table 2: Comparison of the present study with previous quality metrics of goose genome assembly.

Genomic features	Lion-head goose	Zhedong white goose	Sichuan white goose	Tianfu goose
Estimate of genome size (bp)	1,278,045,811	1,208,661,181	1,198,802,839	1,277,099,016
Total length of contigs (bp)	1,268,074,106	1,086,838,604	1,100,859,441	1,113,842,245
Total length of scaffolds (bp)	1,277,289,474	1,122,178,121	1,130,663,797	1,113,913,845
Number of contigs	1,318	60,979	53,336	2,771
Number of scaffolds	1,266	1,050	1,837	2,055
Contig N50 (bp)	21,589,146	27,602	35,032	1,849,874
Scaffold N50 (bp)	27,064,542	5,202,740	5,103,766	33,116,532
Longest contig (bp)	91,420,268	201,281	399,111	10,766,871
Longest scaffold (bp)	98,160,899	24,051,356	20,207,557	70,896,740
GC content	42.39%	38.00%	41.68%	42.15%
No. of predicted protein-coding genes	21,010	16,150	16,288	17,568
Percentage of repeat sequences	12.25%	6.33%	6.90%	8.67%

Table 3: Descriptive statistical of body weight traits.

Species	Number	Max (Kg)	Min (Kg)	Mean±SEM
Lion-head goose	416	15.70	9.00	13.55±1.97
Magang goose	20	5.50	4.80	5.32±0.36
Huangzong goose	20	4.30	2.70	3.40±0.83
Wuzong goose	44	2.50	1.80	2.24±0.25

Table 4: Genome-wide association analysis of body weight in geese.

Chr	Allele	Physical position	Regression coefficient	P value	Genes
2	A	108496954	-0.1886	1.01E-08	LDLRAD4
2	G	7706165	0.2612	6.98E-06	LDLRAD4
3	T	123032780	-0.3979	6.03E-07	EGF, KBTBD
6	A	13264157	-0.24	6.28E-07	TSPAN
6	T	66027192	0.2127	8.14E-07	IGFN1
7	T	39117443	-0.3131	4.66E-06	—
8	T	14712470	0.1865	8.97E-09	PPEF1
9	T	26883582	-2.7E+12	0	OR
10	C	23997415	-0.3032	1.19E-06	—
10	C	23997399	-0.2542	1.05E-05	—

10	T	23997401	-0.2542	1.05E-05	—
11	A	22838749	0.1548	9.55E-06	—
15	T	10257386	0.2527	2.96E-07	GPR180, GPCPD1
16	A	1477673	-0.1892	6.53E-06	—
16	G	1477679	-0.1891	6.78E-06	—
20	A	8531879	0.151	3.05E-06	—
22	A	1992485	-0.3972	6.51E-09	GALNT, AUTS2
22	A	1992518	-0.3973	7.69E-09	GALNT, AUTS2
22	G	1992501	-0.3974	7.94E-09	GALNT, AUTS2
22	C	1992505	-0.3974	7.94E-09	GALNT, AUTS2
22	C	1992507	-0.3974	7.94E-09	GALNT, AUTS2
22	G	1992515	-0.3974	7.94E-09	GALNT, AUTS2
28	C	3587271	0.2936	5.81E-08	PPP1R15B, FGD2
28	G	4472051	-0.2359	2.82E-06	PPP1R15B, FGD2
30	C	1652158	-0.3469	7.53E-07	SH2
30	T	1258517	0.2205	1.48E-06	SH2
30	G	2422665	0.1894	2.04E-06	SH2
30	T	2422666	0.1894	2.04E-06	SH2
30	A	1652207	-0.3289	2.3E-06	SH2
30	T	2269897	0.211	9.22E-06	SH2
32	G	655318	0.2599	7.95E-06	—
33	A	975487	0.2567	1.07E-08	SDHA
36	A	1523127	-0.3274	9.86E-07	SPRY
36	G	1523132	-0.3216	1.7E-06	SPRY
36	C	1523105	-0.3291	1.72E-06	SPRY

Manuscript pdf Click here to access/download;Manuscript;manuscript.pdf

Chromosome-level genome assembly of goose provides insight into the adaptation and growth of local goose breeds

Qiqi Zhao[1,3,5], Junpeng Chen[2], Zi Xie[1,3,5], Jun Wang[4], Keyu Feng[1,3,5], Wencheng Lin[1,3,5], Hongxin Li[1,3,5], Zezhong Hu[1], Weiguo Chen[1,3,5], Feng Chen[1,3], Muhammad Junaid[4], Huanmin Zhang[6], Zhenping Lin[2*], Qingmei Xie[1,3,5*], Xinheng Zhang[1,3,5*]

[1]Heyuan Branch, Guangdong Provincial Laboratory of Lingnan Modern Agricultural Science and technology & Guangdong Provincial Key Lab of Agro-Animal Genomics and Molecular Breeding, College of Animal Science, South China Agricultural University, Guangzhou, Guangdong 510642, China; [2]Shantou Baisha Research Institute of Original Species of Poultry and Stock, Shantou, Guangdong 515000, China; [3]Department of Science and Technology of Guangdong Province, Key Laboratory of Animal Health Aquaculture and Environmental Control, Guangzhou, Guangdong 510642, China; [4]College of Marine Sciences, South China Agricultural University, Guangzhou, Guangdong, 510642, China; [5]Guangdong Engineering Research Center for Vector Vaccine of Animal Virus, Guangzhou, 510642, China and [6]Avian Disease and Oncology Laboratory, Agriculture Research Service, United States Department of Agriculture, East Lansing, MI, 48823, USA

* Correspondence address:

Zhenping Lin, Shantou Baisha Research Institute of Original Species of Poultry and Stock, Shantou, China. E-mail: Linzp02@163.com; Qingmei Xie and Xinheng Zhang, College of Animal Science, South China Agricultural University, Guangzhou, China. E-mails: qmx@scau.edu.cn (Q.X.); xhzhang@scau.edu.cn (X.Z.)

running title: Goose chromosome-level Genome Assembly

Abstract

Backgrouond: *Anatidae* contains numerous waterfowl species with great economic value, but the genetic diversity basis remains insufficiently investigated. Here, we report a chromosome-level genome assembly of Lion-head goose (*Anser cygnoides*), a native breed in South China, through the combination of PacBio, Bionano and Hi-C technologies. **Findings:** The assembly had a total genome size of 1.19 Gb, consisting of 1,859 contigs with an N50 length of 20.59 Mb, generating 40 pseudochromosomes, representing 97.27% of the assembled genome, and identifying 21,208 protein-coding genes. Comparative genomic analysis revealed that geese and ducks diverged approximately 28.42 million years ago, and geese have undergone massive gene family expansion and contraction. To identify genetic markers associated with body weight in different geese breeds including Wuzong goose, Huangzong goose, Magang goose and Lion-head goose, a genome-wide association study was performed, yielding an average of 1,520.6 Mb of raw data with detecting 44,858 SNPs. GWAS showed that six SNPs were significantly associated with body weight and 25 were potentially associated. The significantly associated SNPs were annotated as *LDLRAD4*, *GPR180*, *OR*, enriching in growth factor receptors regulation pathways. **Conclusions:** We present the first chromosome-level assembly of the Lion-head goose genome, which will expand the genomic resources of the *Anatidae* family, providing a basis for adaptation and evolution. Candidate genes significantly associated with different goose breeds may serve to understand the underlying mechanisms of weight differences.

Keywords: Lion-head goose, Genome assembly, Comparative genome, Genome-wide association study

Introduction

The *Anatidae* is a family of the ancient *Aves* class with order *Anseriformes*, containing 43 genuses and 174 species, including most birds of *Anseriformes*, such as ducks, geese, swans, and is the most prominent family of swimming birds [1]. Physical characteristics and features vary significantly among species, making the *Anatidae* family rich in diversity and specificity. *Anatidae* adults are usually herbivores, feeding on a variety of aquatic plants, which are well suited to sustainable production practices thereby reducing competition for human food; and some species are even used for crop weeds and pests control [1, 2]. For a long time, duck and goose feathers have been popular in pillows, quilts and coats [3]. Several species in the genus *Anser* are commercially important and domesticated as poultry because of their meat-producing performance and the warmth properties of feather products. According to archaeological evidence, geese were domesticated around 6,000 years ago near the Mediterranean Sea, and later spread around the world due to human activities [4]. It is widely believed that *Anser cygnoides* is the ancestor of the Chinese goose (*Anser cygnoides domesticus*) with a domestication history of more than 3,000 years [1]. After artificial domestication, the domestic goose has increased its cold tolerance and roughage-resistance, but its wings are degraded and weakened in flight, unable to travel long distances [1]. Egg-laying rate and goslings survival rate are also improved compared to wild swans, and the lifespan is longer [5]. Furthermore, overfeeding can cause foie gras to be at least three-fold larger than the normal size while the goose remains healthy, making the goose a good model to study human liver steatosis [6]. Chinese domestic geese is a natural gene pool containing local breeds of diverse phenotypes, and adult domestic geese from similar region vary greatly in weight [7]. For example, the Lion-head goose in Shantou (116°14'-117°19' E, 23°02'-23°38' N), Guangdong Province, can weigh more than 9 kg, while in the Wuzong goose from Qingyuan (111°55'-113°55' E, 23°31'-25°12' N), Guangdong Province, the average weight is only about 3 kg [8, 9]. The Lion-head goose has a large body, a deep and wide head, and large sarcomas (five sarcomas) on the front and side of the face **(Fig. 1)**. The adult male goose weighs 9-10 kg and the female goose 7.5-9 kg, grows rapidly and has rich muscles. Wuzong goose is a small goose species with a distinct band of black plumage from

neck to back. The gander weighs 3-3.5kg and the female weighs 2.5-3kg, with wide and short body, flat back, and thin and short feet. Magang goose is a medium-sized goose species, with a long head, wide beak, rectangular body, a gray-black bristle-like feathers on the back of the neck, gray brown breast feathers and white belly feathers. Adult weight is 4-5 kg for males and 3-4 kg for females. Huangzong goose has a compact body, from the top of the head to the back of the neck has a brownish yellow feather belt, shaped like a horse's mane. The chest feather is gray yellow, the belly feather is white, the beak and sarcoma is black. Adult males weigh 3-3.5 kg, females 2.5-3 kg. However, the mechanisms for such differences have not been clarified, let alone being resolved at the genomic level. Therefore, a complete, continuous and accurate reference genome is essential, for deciphering genomic diversity, evolutionary and adaptive processes, improving production efficiency and even promoting the development of goose industry.

High-quality genome assembly sequences enable us to comprehensively and scientifically decode the genetic diversity of species, explore disease mechanisms, and understand species evolution. Recently, Pacbio has offered technology that can generate reads several thousand bases in size, and these long reads can span repetitive regions [10]. Although these long reads have a high error rate, they can be integrated with Illumina's short reads to improve sequencing accuracy [11]. In addition, new scaffolding techniques, such as high-throughput chromosome conformation capture (Hi-C), allow the genome to be assembled to the level of whole chromosomes [12]. Pacbio single molecule real-time (SMRT) sequencing technology has been extensively used in the study of human diseases such as tuberculosis and influenza virus [13], as well as in the study of species evolution, such as the centromere of the human Y chromosome [14]. Bionano optical mapping technology has advantages in obtaining highly repetitive sequences and detecting genomic structural variants, which is helpful for remote sequencing of sequence overlap clusters[15]. Bionano has become a powerful tool for genome assembly, a 5.1 Mbp inversion was found in the genomes of a patient with Duchenne muscular dystrophy[16].

In this study, we report the genome assembly at the chromosome level in Lion-head geese for the first time using combined data generated by four advanced technologies, Illumina, SMRT, Bionano, and Hi-C. In addition, we investigated the relationship between body weight and genetic variations in Lion-

head goose, Wuzong goose, Huangzong goose and Magang goose by genome-wide association analysis, trying to identify the genes involved in body weight determination from different species. These will offer valuable resources for facilitating genetic research and the improvement of the species and for studying speciation and evolution in geese.

Methods

Animal selection

An adult healthy purebred male Lion-head goose (*Anser cygnoides*) with classical traits was selected for whole-genome sequencing and conducting *de novo* assembly from Shantou Baisha Research Institute of Original Species of Poultry and Stock. Blood and eight tissues (i.e., brain, pharyngeal pouch, head sarcoma, spleen, liver, chest muscle, kidney, and heart) from another four healthy adult individuals were collected for RNA-seq analysis. All applicable institutional and national guidelines for the care and use of animals were followed. All the animal work in this study was approved by the South China Agricultural University Committee for Animal Experiments (approval ID: SYXK 2019-0136). All the research procedures and animal care activities were conducted based on the principles stated in the National and Institutional Guide for the Care and Use of Laboratory Animals.

Genome survey library construction and sequencing

To survey the genome profile, high-quality genomic DNA was extracted from the blood of the reference individual for whole-genome sequencing using the Qiagen Blood and Cell Culture DNA Midi Kit according to the manufacturer's instructions. For the quality control of purity, concentration, and integrity, we used Qubit 2.0 Fluorometry (Life Technologies, USA), NanoDrop 2000 spectrophotometer (Thermo Scientific), and pulse-field gel electrophoresis (Bio-rad CHEF-DR II), respectively. The following steps used for DNA extraction and quality control were similar. The short paired-end Illumina DNA library was constructed using the Illumina HiSeq system (with the paired-end 350 bp sequencing strategy). After performing the sequencing and obtaining the data, the k-mer analysis of reads for the genome survey was calculated by the Jellyfish program with the default parameters. Additionally, the genome size, heterozygosity ratio, and repeat sequence ratio were calculated with the GenomeScope

tool based on the k-mer frequency of 17.

Genome sequencing and assembly strategies

A 40 kb *de novo* library for SMRT genome sequencing was constructed using the PacBio Sequel III platform (Pacific Biosciences, USA). All of these reads were used for contigs assembly. A scalable and accurate long-read assembly tool, Canu (v1.8) [17], was employed to correct and assemble the PacBio reads with the listed parameters (minThreads = 4, genome size = 1200m, minOverlapLength = 700, minReadLength = 1000). The resulting contigs and corrected reads were used as inputs for HERA [18] to fill the gaps and produce longer contigs with default parameters. After that, Illumina paired-end clean data were mapped to the corrected contigs with the Burrows-Wheeler Aligner (BWA) [19], and the results were filtered by Q30 with Samtools (v1.8) [20]. At last, Pilon (v1.22) [21] was used to polish the assembly and enhance the base accuracy of the contigs.

Physical optical genome maps from BioNano were used to improve the assembly quality of the genome, with the ultimate goal of generating a chromosome-scale assembly. Nuclear DNA was extracted from the blood sample of the reference individual and digested with nickase Direct Labeling Enzyme I. After labeling, repairing and staining reactions, DNA was loaded onto the Saphyr Chip for sequencing to generate BioNano molecules. Afterward, the data were assembled with RefAligner and Assembler of BioNano Solve. The scaffold was established using BioNano Solve with HERA's contigs and a BioNano genome map. When encountering a conflict between a contig and the genome map, the contig was split to correct the false connection.

For Hi-C library, fresh blood was vacuum-infiltrated with 2% formaldehyde solution and then used for cross-link action. Later nuclear DNA was isolated from the reference animal and digested with the restriction enzyme Mbo I. The Hi-C library with insertion sizes of 350 bp was constructed and sequenced on the Illumina HiSeq X Ten instrument. The Hi-C reads were assigned to the scaffolds by Juicer [22]. The scaffolds were further clustered, ordered, and oriented to the chromosome-level scaffolds by 3D-DNA [23]. Thus, a heatmap of Hi-C chromosomal interaction was created using the HiC-pro software [24].

RNA-Seq and transcripts assembly

RNA-seq was conducted on blood and eight different tissues (i.e., brain, pharyngeal pouch, head sarcoma, spleen, liver, chest muscle, kidney, and heart) from four healthy adult Lion-head goose. Total RNA was extracted from four individuals using the TRIZOL reagent and purified following the manufacturer's protocols. The concentration and quality of the isolated RNA were assessed using the Nanodrop Spectrophotometer, Qubit 2.0 Fluorometry, and the Agilent 2100 bioanalyzer (Agilent Technologies, USA). Libraries construction and sequencing were performed using the Illumina NovaSeq 6000 platform. Raw RNA-seq data with 150 bp paired-end reads were trimmed for quality using Trimmomatic [25]. Thus, the Illumina sequence adaptors were removed, then low-quality reads based on Phred scores and polluted reads containing N > 5% were trimmed, using the following parameters: LEADING:3 TRAILING:3 SLIDINGWINDOW:4:15 -threads 20 MINLEN:50. Furthermore, Trinity [26] was used to *de novo* assemble the data after quality filtering. To remove redundant sequences, CD-HIT [27] was employed to remove highly identical transcript isoforms, retaining only the longest one. After filtering, the RNA-seq reads were mapped to the assembled genome using the default parameters of STAR [28].

Assembly evaluation

Finishing the genome assembly, quality control for the assembly's quality, accuracy, and integrity was predicted by Benchmarking Universal Single-Copy Orthologs (BUSCO, v 3.0), using aves_odb10 as the query [29].

Genome annotation

The genome assembly was annotated by MAKER, mainly including gene annotation and repeat annotation. The detailed pipeline was based on proteins from the Uniprot, the *de novo* assembly of RNA-seq data, and the total proteins of the relative species *Anser cygnoides* [30]. The transposable elements (TE) associated genes that were filtered out by the TEseeker database, and the results were used to conduct functional annotation using InterProScan. The repeat sequencing library was identified and annotated by a combination of LTR-FINDER and RepeatModeler. RepeatMasker and the query species "Chicken" were used to mask the repeats in the assembly, based on the Repbase database and the previous repeat sequence library. Tandem repeats were discovered by the Tandem Repeats Finder [31].

Gene families and phylogenetic analysis

Interspecific syntenic blocks between the Lion-head goose and duck were explored using MCscan [32] after coding sequence alignment by BLASTn. The same method was used for intraspecific collinearity analysis. To gain insight into the gene family evolution of the goose, we compared the gene families of Lion-head goose with the genomes of the following avian species: Zhedong white goose (*Anser cygnoides*), duck (*Anas platyrhynchos*), turkey (*Meleagris gallopavo*), chicken (*Gallus gallus*), pigeon (*Columba livia*), saker (*Falco cherrug*), titmouse (*Pseudopodoces humilis*), and green lizard (*Anolis carolinensis*). Initially, alternative splicing and genes encoding less than 50 amino acids with a proportion of stop codon greater than 20% were filtered; meanwhile, the longest transcript of genes with multiple isoforms was retained to represent the gene. Similarity relationships among the protein sequences of species were aligned by BLASTP algorithm and clustered using OrthoMCL methodology with an expansion coefficient of 1.5 to obtain single- and multiple-copy gene families, and specific gene families of Lion-head goose. The sequences of the single-copy gene families were employed to perform multiple alignments by MUSCLE. Then RAxML [33] was used to construct a phylogenetic tree of nine species, with the green lizard (*Anolis carolinensis*) being designated an outgroup. Taking the divergence time of the pigeon and turkey (92.9Mya, http://www.timetree.org/) as the calibration, the r8s [34] software was used to estimate the divergence time of the species and construct ultrametric trees. After filtering out gene families with gene counts of more than 100 in some individual species, CAFÉ [35] was employed to detect gene families that had undergone expansion or contraction per million years independently along each branch of the phylogenetic tree. Subsequently, a gene ontology (GO) enrichment analysis of gene families was performed using the clusterProfiler package in R [36].

Experimental sample processing and variant detection for Genome-wide association study

Blood samples of 514 geese (including Lion-head goose, Wuzong goose, Huangzong goose and Magang goose) were collected and stored in 2 mL tubes containing ACD anticoagulant for DNA extraction, and the weight of the geese was recorded. DNA was extracted from blood samples using the HiPure Blood DNA Mini Kit (Magenbio, Guangzhou, China). The samples that passed the quality testing were

subjected to library construction using Easy DNA Library Prep Kit (MGI, Shenzhen, China) and paired-end 100 sequencing using MGIseq 500. Raw data were filtered for adaptors and low quality reads using SOAPnuke software, low quality threshold parameters set to 5, and the filtered sequences were compared with the constructed goose reference genome using BWA software with parameters: mem, -M. Then variant detection was performed using Samtools, GATK4 software with parameters: HaplotypeCaller –ERC GVCF. SNP variants were filtered based on a minimum allele frequency threshold of 0.05, a Hardy Weinberg equilibrium test significance threshold of 10^{-7}, and a max missing rate threshold of 0.7. Principal component analysis (PCA) was performed and plotted with R. To understand the kinship among the samples, the phylogenetic trees were constructed using SNP data with Phylip software.

Genome-wide association study

The genetic variation was analyzed with individualcorresponding weight information using the asymptotic Wald test (assoc) in Plink. The top 20 PCs in PCA analysis were used as covariates, and linear analysis was performed on sample variances with corresponding weight information by Plink.And the common parameters in two types of model analysis is --allow-extra-chr --allow-no-sex -out, where the assoc parameter is -assoc and the linear parameter is --linear --covar plink.eigenvec. The statistical analysis model for genome-wide association analysis was as follows:

$$P = \mu + Z\alpha + SNP + e$$

where P is the phenotypic variable; μ is the intercept; Z is the random multigene effect relationship matrix; α is the random multigene effect; SNP is the SNP effect; e is the residual, distributed as e~(0, I σ_e), and I is the unit matrix.

Genome-wide 5% significance threshold was determined using the Bonferrorni method. To reduce false negative, the threshold was expanded by 20-fold as a second threshold and the SNP in this region was defined as potentially associated. The SNPs with Bonferroni corrected p-values less than 0.05 in the results of the assoc and linear analyses were annotated. The corresponding genes annotated with significantly related SNPs were used to identify the GO pathway.

Selective-sweep analysis

To analyze regions affected by long-term selection and are associated with domestication of geese, we calculated the Fixation indices (F_{ST}) for four goose species using vcftools software with sliding windows length of 20 kb that had a 10-kb overlap between adjacent windows. The top 5% of regions were designated as candidate selective regions and the genes in these regions were considered as candidate genes.

Results

Genome sequencing and assembly

The Lion-head goose is a famous local variety in China and one of the most giant goose breeds worldwide, with a unique appearance and social benefits. Here, we attempt to construct a highly continuous chromosome-scale genome of an adult purebred male Lion-head goose with a high degree of homozygosity to minimize heterozygous alleles. The following sequencing and genome assemble strategies were applied: Illumina sequencing, Pacbio SMRT sequencing, BioNano optical mapping, and Hi-C approach **(Supplementary Table S1)**. We assemble these data step by step and generate progressively improved assembled genome **(Supplementary Figure S1)**. A total of 185.37 Gb of high-quality Pacbio long reads were generated, representing a ~168× depth of the estimated 1.05 Gb genome with heterozygosity of 0.335% based on the k-mer analysis of the Illumina sequences **(Supplementary Figure S1, Supplementary Table S2)**. Combing the *de novo* assembly of the Illumina and Pacbio sequences resulted in a draft genome of 1.20 Gb, yielding 1,859 contigs with a length of 13.7 Mb for contig N50 and 57.6 Mb for the longest **(Table 1)**. Furthermore, with the help of BioNano optical mapping, the scaffold N50 value was increased to 37 Mb. To obtain a chromosome-scale assembly, a set of ~230 Gb Hi-C data was used to orient, order, phase, and anchor the contigs. Approximately 97.27% of the reads assembled were anchored to 40 high-confidence pseudo-chromosomes (39 autosomes and Z chromosome) using the high-density genetic map **(Supplementary Figure S1, Fig. 2)**. After polishing, we finally assembled the ultimate genome into 1.19 Gb with the final contig N50 of 20.59 Mb and scaffold N50 of 25.8 Mb, with a GC content of 42.39% **(Supplementary Table S2 and S3)**. The structure and quality of the assembled genome were determined by mapping a Hi-C chromosomal

contact map.

The completeness of the Lion-head goose genome assembly was assessed using the BUSCO gene set. The result showed that almost 99.02% of the reads were correctly mapped to the genome. We then evaluated the assembled genome with 98.24% single-copy and 1.76% duplicated orthologs from the BUSCO dataset, confirming that 8,081 genes (96.92%) were intact in this genome. These results indicate the high reliability and integrity of the assembled genome **(Supplementary Figure S2 and Table S4)**.

Genome annotation

To support the genome annotation, we conducted RNA-Seq analysis using RNA samples of blood and eight tissues (brain, pharyngeal pouch, head sarcoma, spleen, liver, chest muscle, kidney, and heart) from four healthy adult individuals. The aggregate of 760 Gb raw reads was accumulated by the paired-end sequencing of the 36 constructed libraries. After filtering the adaptor and low-quality sequences, 723 Gb qualified Illumina reads remained, *de novo* assembled into unique transcripts (unigenes). Overall, a total of 216,229 unigenes were assembled and at the level N50, 5,082 nucleotides were obtained. Total 21,208 protein-coding gene annotations were predicted in Lion-head goose by combining *de novo* prediction, homologous protein prediction, and transcription alignment. After filtering TE-related genes, a total of 21,010 protein-coding gene annotations were finally obtained by the TE seeker database **(Fig. 2)**. Furthermore, a total of 8.15% repeat sequence and 4.10% tandem repeats of the genome were detected **(Table 1)**. Comparative statistics of genome quality metrics with the assembled goose genome (including Zhedong white goose, Sichuan white goose and Tianfu goose) are shown in **Table 2**.

Phylogenetic analysis

To investigate the genomic evolution of poultry, we compared the sequences of eight bird species (Lion-head goose, Zhedong white goose, duck, turkey, chicken, pigeon, saker, and titmouse) and green lizard, clustering the genes into 15,162 gene families **(Fig. 3A, Supplementary Table S5)**. Among these, 6,422 single-copy gene families were identified and used to construct a phylogenetic tree **(Fig. 3B)**. This revealed that the geese and ducks were clustered into a subclade that probably evolved from a common ancestor approximately 28.42 million years ago (Mya). As expected, the Lion-head goose displayed a

close relationship with the Zhedong white goose. The divergence time between the Lion-head goose and Zhedong white goose was estimated to be 13.79 Mya, and that between chicken and turkey was nearly 25.07 Mya. The above results confirmed the reliability of the tree.

Of all the gene families in the Lion-head goose, 4,233 gene families were significantly expanded and 324 were contracted. Compared with Zhedong white goose, the Lion-head goose had more gene families and there are also more events of gene family expansion and contraction. Moreover, we mixed the gene family sets of several *Anatidae* varieties (duck, Zhedong white goose, Lion-head goose), and performed expansion and contraction analysis and corresponding GO enrichment analysis. In this task, the GO analysis of expanded gene families suggested the olfactory perception, such as detection of chemical stimulus involved in sensory perception of smell (GO:0050911, $p = 6.97\times10^{-8}$), and odorant-binding (GO:0005549, $p = 1.47\times10^{-5}$), both of which may be related to the adaptation of the species to find food in water **(Fig. 4A, Supplementary Table S6)**. Meanwhile, contracted gene families were concentrated in the areas of glucose synthesis and metabolism, such as hexokinase activity (GO:0004396, $p = 7.64\times10^{-26}$), glucose binding (GO:0005536, $p = 2.30\times10^{-22}$), cellular glucose homeostasis (GO:0001678, $p = 6.84\times10^{-18}$), glycolytic process (GO:0006096, $p = 1.75\times10^{-15}$), hexose metabolic process (GO:0019318, $p = 2.66\times10^{-14}$), carbohydrate phosphorylation (GO:0046835, $p = 1.68\times10^{-9}$), and glucose 6-phosphate metabolic process (GO:0051156, $p = 1.27\times10^{-9}$), which may be closely related to characteristics of glycogen storage and utilization during migration **(Fig. 4B, Supplementary Table S7)**. Besides, 220 unique gene families (other species lack these gene families) of the Lion-head goose were identified and functionally annotated in GO categories, such as protein kinase activity (GO:0004672, $p = 6.85\times10^{-9}$), the regulation of apoptotic process (GO:0042981, $p = 5.78\times10^{-34}$), the adenylate cyclase-modulating G protein-coupled receptor signaling pathway (GO:0007188, $p = 5.92\times10^{-3}$), and fatty-acyl-CoA reductase (alcohol-forming) activity (GO:0080019, $p = 8.94\times10^{-5}$, **Fig. 4C, Supplementary Table S8)**. Interestingly, we annotated a reproduction-related protein in the species-specific gene family, *Sterile* (Pfam ID: PF03015), acting on fatty-acyl-CoA reductase (alcohol-forming) activity, which may be related to the low reproductive rate caused by congenital infertility in geese.

Collinearity analysis allows one to judge molecular evolutionary events between species and explain

the structural differences between the two genomes. We identified synteny blocks among avian genomes and found high collinearity between our assembly and the duck genome (genome size =1.19 Gb). Here, multiple chromosomes (Chr 1-5, 10, 12, 15, 17-20, 23, 26, 27, 29, 30, 32, 34, 36, 37, 39) of Lion-head goose were almost one-to-one collinear with those of the duck, but some chromosomal rearrangements occurred **(Fig. 3C, Supplementary Figure S3)**. For example, on some chromosomes like Chr 1, 2, 3, and 4 of the duck genome, genes break and rearrange on the Lion-head goose genome, resulting in sequential inversion. In addition, some scaffolds such as Chr 9, 24, 25, 31, 35, 38 and 40, were not correlated with any chromosome of the duck genome maybe due to the different sources of genes on the chromosome. These results indicate that chromosome inversion and interchromosomal recombination may have occurred specifically in Lion-head goose during the evolutionary process, but this requires further investigation and verification. Moreover, Chr 4 of Lion-head goose was found to correspond to the sex chromosome Z of duck, except for the inversions of small patches of segments; therefore, we inferred that Chr 4 was the sex chromosome of the Lion-head goose. This information will be fundamental for comparative genomic studies in *Anatidae* animals.

Cluster analysis of different goose species population

Blood samples were collected from 514 geese (including Lion-head goose, Wuzong goose, Huangzong goose and Magang goose), and their weight was recorded, with the Lion-head goose using the minimum weight, the Wuzong goose using the maximum weight, and the Huangzong goose and Magang goose using the average weight. That is, the Lion-head goose weighed at least 9 kg, the Wuzong goose weighed at most 2.5 kg, the Huangzong goose weighed about 3-4 kg, and the Magang goose weighed 4.8-5.5 kg (**Table 6**). Blood from each sample was used for paired-end 100 resequencing. And the average raw data was 1,520.60 Mb, the average sequencing depth was 12.05×, the average coverage was 7.56%, the average matching rate was 91.31%, and 44,858 SNP loci were retained for subsequent analysis after screening SNPs with minimum allele frequency <5%, Hardy-Weinberg equilibrium test significance threshold of 10^{-7}, and maximum deletion rate threshold of 0.7. We reconstructed the goose population structure using SNP data, revealing four distinct subpopulations. The PCA results demonstrated that the Lion-head Goose population was clearly distinguishable from the Magang Goose, Wuzong Goose and

Huangzong Goose, and there was a clear differentiation within the species (**Fig. 5A**). The clustering of Magang Goose and Huangzong Goose was closer together, probably related to their closer geographical location and the existence of some genetic exchange. The phylogenetic tree results were consistent with the PCA results. The clustering of Magang Goose and Huangzong Goose was closer to each other, and they clustered into one branch with Wuzong Goose (**Fig. 5B**).

Candidate genomic regions for body weight based on combined analyses of GWAS and selective-sweep

The Lion-head Goose, Huangzong Goose, Magang Goose, and Wuzong Goose are all local species in Guangdong, but they differ greatly in body weight. In this study, we sought to reveal genomic changes associated with body weight in the four goose species and screen genomic regions and genes. Selective sweep analysis was performed based on the F_{ST} index, considering the top 5% window as candidate regions. And 979 selective regions containing 818 genes were detected.

We then combined the GWAS results with the detected selective features to screen for candidate genomic regions responsible for the differences in goose weight. From the Manhattan plot (**Fig. 5C**), a total of 10 significant signals were found to be associated with body weight trait in geese at the genome-wide level, including one significant SNP detected on Chr 2, 8, 9, and 33 respectively (-log (p) > 7.30), and six significant SNPs annotated by two genes on Chr 22, with the closest Manhattan plot SNP peak on Chr 9 for the gene *OR* (Olfactory receptor). Six significant SNPs on Chr 22 are located between 1,992,485 and 1,992,520 bp, a region that spans only a physical distance of 35 bp but contains six SNP loci, making it necessary to analyze these SNPs in this small region in detail to determine whether multiple QTL are involved. The most significant SNP in this region could explain about 8.19% of the phenotypic variation. Apart from significant SNPs, potentially significant QTLs were detected on many chromosomes (including Chr 2, 3, 6, 7, 10, 11, 15, 16, 20, 28, 30, 32, 36), with a total of 25 implied significant SNPs (4.90< -log (p) <7.30). On Chr 30, the suggestively significant SNPs were located between 1,258,517 and 2,422,666 bp, spanning approximately 1.16 Mb, with the most significant SNPs in this region explaining approximately 6.12% of the phenotypic variation (**Table 4**). In the present study, we identified genes in the region near the significant SNPs, annotating a total of 21 genes. These genes

may be important in mediating growth and development, and we inference that the *LDLRAD4* gene may play a key role in developmental plasticity in geese, while the *GPR180* gene may regulate the locomotor behavior of geese to make them stronger (**Fig. 6**). GWAS peaks overlapped with genomic regions with selective features on some chromosomes (**Supplementary Data**). This suggests that the region carrying QTL are not only associated with body weight in GWAS, but are also under selection during domestication.

Discussion

Despite the importance of the genus *Anser*, an economically important animal, the relative scarcity of genomic resources has largely hindered progress in studying genome evolution and molecular breeding in the major animals. High-quality chromosome-level genomes can provide key resources for studying. This study describes a chromosome-scale assembly of Lion-head goose obtained by a combination of data from the Illumina, SMRT, BioNano, and Hi-C platforms. The genome assembly is 1.19 Gb in length, and more than 97.27% of the assembled genome is anchored on 40 pseudo-chromosomes. The BUSCO assessment revealed 99.02% complete genes in the assembled genome, making it a better-continuity and higher-quality genome assembly than the recently published Tianfu goose genome with a contig N50 of 1.85 Mb and scaffold N50 of 33.12 Mb [37]. Compared with the cultivated breed Tianfu goose, Lion-head goose, a traditional native breed, should occupy a more prominent position in the germplasm resources, and its evolving message can provide a reference for other local breeds which is worthy of in-depth study.

Comparative genomics is the analysis of the structural characteristics of multiple individual genomes of a species or genomes of multiple species to find out the similarities and differences of gene sequences of species with the help of bioinformatics, and then to study the gene family analysis, analyze the differentiation and evolution of species, to provide a basis for elucidating species evolution. In this study, the evolutionary events of the Lion-head goose were analyzed by comparing the genome sequences with those of other birds. The results showed that the Lion-head goose and Zhedong White goose were most closely related, diverging at about 13.8 Mya, while the geese and ducks diverged at 28.4 Mya. The

results were similar to those of Zhedong White goose, Sichuan White goose and Tianfu goose, indicating the accuracy of the assembly result of this study. Comparative genomic analysis revealed the genetic basis of interesting characters, which helped elucidate important biological implications and obtain solutions for genomic evolution between Lion-head geese and other species of *Anatidae* family, facilitating future genetic breeding programs. This is the first chromosomal level reference genome of Lion-head goose, providing important genomic data for the study of the family *Anatidae*.

The genomic information of the species population was obtained by whole-genome resequencing, and a large amount of variation information was obtained by comparison with the reference genome. Based on the correlation between differences in variation information and phenotypic differences of individuals, the adaptation of species to the environment, scanning of variant loci associated with important traits at the genome level, and localization of genetic mutations were discussed. Lion head goose, Magang goose, Huangzong goose and Wuzong goose are the main breeds of geese in Guangdong Province. Although they all belong to Guangdong Province, the body weight of adult geese varies greatly, and the molecular mechanism causing the huge difference is still unclear. In this study, four goose species were resequenced and examined for variation. Principal component analysis and phylogenetic tree analysis revealed significant differences among several goose species, indicating the feasibility of this study. Subsequently, GWAS was used to identify the candidate functional SNPs that might cause the weight difference of the four goose species, and the genes such as LDLRAD4, GPR180, and OR were analyzed and annotated, attributed to play an important role in mediating growth and development. Recently, there have been several studies related to agricultural traits that have achieved success in animal GWAS projects, for example, GWAS for improving reproductive performance and egg quality in geese and *TMEM161A* gene for embryo development [38]. Genome-wide association analysis of the early-lactation milk fat content in 3,513 Fleckvieh bulls and 2327 Holstein bulls detected 6 associated QTL regions, two of which were located near the gene DGAT1 [39]. GWAS was conducted on 225 ducks with different-sized black spots, and the results showed that EDNRB2 was the gene responsible for the variation in duck body surface spot size [40]. In this study, *LDLRAD4* (low-

density lipoprotein receptor class A domain containing 4), *OR* (Olfactory receptor), and *GPR180* (G protein-coupled receptor 180) were mainly found to function in body weight traits. Knockdown of *LDLRAD4* enhances transforming growth factor (TGF)-β-induced cell migration, which in turn regulates cell growth, differentiation, motility, apoptosis and matrix protein production [41]. The olfactory receptor (*OR2AT4*) has been shown to stimulate the proliferation of keratin-forming cells in peripheral human tissues [42]. *GPR180*, a component of the TGF-β signaling pathway, also has metabolic relevance in the body and may play an essential role in regulating adipose tissue and systemic energy metabolism [43]. Here we found some correlation between these genes and the TGF-β signaling, presumably this pathway also acts on body weight. Identifying of molecular genetic markers and the main effect QTL associated with critical agricultural traits is of great interest to breeders. Nevertheless, the candidate genes identified in this study were only detected by sequencing data and not experimentally validated. The functions of these candidate SNPs and gene markers need to be further verified by experimental results or other techniques. Thus, the findings in our GWAS study represent a valuable resource for geese and provide a new opportunity and basis for geneticists and breeders to work together to explore the genetics behind various agricultural traits.

Conclusions

In summary, we have obtained a high-quality chromosome-scale draft assembly of a purebred Lion-head goose, which provides a genetic basis for understanding the acquisition of related traits and facilitates advances in goose genomics and genetic improvement. Moreover, the candidate genes and their variants identified in this study will help clarify our understanding of goose selective breeding and the development of new breeds. The obtained genome sequence of Lion-head goose is a vital addition to the genome of genus *Anser* and is valuable for further understanding goose molecular breeding strategies. This genomic resource is also of high value for evolutionary studies of closely related species.

Data Availability

The final genome assembly data supporting the results of this article is available in the NCBI BioProject repository, [Accession number: PRJNA736831]. The RNA assembly data is available in the NCBI

BioProject repository, [Accession number: PRJNA807796]. The raw re-sequencing genome data supporting of the GWAS study is available in the NCBI BioProject repository [Accession number: PRJNA552198, PRJNA552383, and PRJNA552384].

Additional Files

Supplementary Figure S1. Sequencing process and presentation.

Supplementary Figure S2. BUSCO assessment of the assembly genome of Lion-head goose.

Supplementary Figure S3. Gene synteny between the Lion-head goose and duck genomes.

Supplementary Table S1. Statistics of sequenced clean data.

Supplementary Table S2. Statistics of genome survey.

Supplementary Table S3. Statistics of genome assembly quality.

Supplementary Table S4. Summary of BUSCOs genome evaluation.

Supplementary Table S5: Summary of gene families from several species.

Supplementary Table S6. GO annotation of expanded gene families from Anatidae varieties (Duck, Zhedong white goose, Lion-head goose; Top 20).

Supplementary Table S7. GO annotation of contraction gene families from Anatidae varieties (Duck, Zhedong white goose, Lion-head goose; Top 20).

Supplementary Table S8. GO annotation of unique gene families from the Lion-head goose.

Supplementary Data. Significant information of selective-sweep analysis.

Abbreviations

BLAST: Basic Local Alignment Search Tool; BWA: Burrows-Wheeler Aligner; BUSCO: Benchmarking Universal Single-Copy Orthologs; Chr: chromosome; GATK4: Genome Analysis Toolkit 4; Gb: gigabase pairs; GO: gene ontology; GPR180: G protein-coupled receptor 180; GWAS: genome-wide association study; HERA: Highly Efficient Repeat Assembly; Hi-C: high-throughput chromosome conformation capture; Kb: kilobase pairs; kg: kilogram; LDLRAD4: low-density lipoprotein receptor class A domain containing 4; LTR: long terminal repeat; Mb: megabase pairs; Mya: million years ago; NCBI: National Center for Biotechnology Information; OR: Olfactory receptor; OR2AT4: olfactory

receptor family 2 subfamily AT member 4; PacBio: Pacific Biosciences; PCA: Principal component analysis; QTL: quantitative trait locus; RAxML: Randomized Axelerated Maximum Likelihood; RNA-seq: RNA sequencing; SMRT: single molecule real-time; SNP: single-nucleotide polymorphism; STAR: Spliced Transcripts Alignment to a Reference; TE: transposable element; TGF: transforming growth factor; TMEM161A: Transmembrane protein 161A.

Competing Interests

The authors declare that they have no conflict of interest.

Funding

This work was supported by the Key Research and Development Program of Guangdong Province (2020B020222001), the Construction of Modern Agricultural Science and Technology Innovation Alliance in Guangdong Province (2021KJ128, 2020KJ128), the National Modern Agricultural Industry Science and Technology Innovation Center in Guangzhou (2018kczx01), the Guangdong Provincial Promotion Project on Preservation and Utilization of Local Breed of Livestock and Poultry (4000-F18260), the Guangdong Basic and Applied Basic Research Foundation (2019A1515012006). The authors would like to thank the BGI in Shenzhen for their work on genome sequencing. We also thank the staff of Minglead Gene for providing the technical and computing support during the research.

Author's Contributions

Q.X., Z.L., and X.Z. conceived and designed the research. X.Z., J.C., and Q.Z. coordinated the project. J.C. and Z.L. provided animal samples. Q.Z. and Z. X. collected and prepared the samples. Q.Z. performed sequencing, assembly and bioinformatics analysis. W.L., and F.C. led work identifying genes, and H.L., W.C. aided with many aspects of gene identification and did the GO analyses. Q.Z., X.Z. wrote and revised the manuscript and the supplementary information. J.W., M.J., Z.H., H.Z., Z.L., and Q.X. participated in discussions and provided valuable advice. All authors read and approved the manuscript.

References

1. Hoyo JD, Elliott A, Sargatal J, et al. Handbook of the birds of the world. Barcelona: Lynx Edicions; 1992.
2. Madsen J, Marcussen LK, Knudsen N, et al. Does intensive goose grazing affect breeding waders? Ecol Evol 2019;**9**(24):14512-14522. doi:10.1002/ece3.5923.
3. Wang Y, Li SM, Huang J, et al. Mutations of TYR and MITF Genes are Associated with Plumage Colour Phenotypes in Geese. Asian-Australas J Anim Sci 2014;**27**(6):778-83. doi:10.5713/ajas.2013.13350.
4. Gao G, Zhao X, Li Q, et al. Genome and metagenome analyses reveal adaptive evolution of the host and interaction with the gut microbiota in the goose. Sci Rep 2016;**6**:32961. doi:10.1038/srep32961.
5. Yao Y, Yang YZ, Gu TT, et al. Comparison of the broody behavior characteristics of different breeds of geese. Poult Sci 2019;**98**(11):5226-5233. doi:10.3382/ps/pez366.

6. Lu L, Chen Y, Wang Z, et al. The goose genome sequence leads to insights into the evolution of waterfowl and susceptibility to fatty liver. Genome Biol 2015;**16**:89. doi:10.1186/s13059-015-0652-y.
7. Li HF, Zhu WQ, Chen KW, et al. Two maternal origins of Chinese domestic goose. Poult Sci 2011;**90**(12):2705-10. doi:10.3382/ps.2011-01425.
8. Tang J, Shen X, Ouyang H, et al. Transcriptome analysis of pituitary gland revealed candidate genes and gene networks regulating the growth and development in goose. Anim Biotechnol 2020:1-11. doi:10.1080/10495398.2020.1801457.
9. Zhang X, Wang J, Li X, et al. Transcriptomic investigation of embryonic pectoral muscle reveals increased myogenic processes in Shitou geese compared to Wuzong geese. Br Poult Sci 2021;**62**(5):650-657. doi:10.1080/00071668.2021.1912292.
10. Ardui S, Ameur A, Vermeesch JR, et al. Single molecule real-time (SMRT) sequencing comes of age: applications and utilities for medical diagnostics. Nucleic Acids Res 2018;**46**(5):2159-2168. doi:10.1093/nar/gky066.
11. Yoshinaga Y, Daum C, He G, et al. Genome Sequencing. Methods Mol Biol 2018;**1775**:37-52. doi:10.1007/978-1-4939-7804-5_4.
12. Kong S, Zhang Y. Deciphering Hi-C: from 3D genome to function. Cell Biol Toxicol 2019;**35**(1):15-32. doi:10.1007/s10565-018-09456-2.
13. Nakano K, Shiroma A, Shimoji M, et al. Advantages of genome sequencing by long-read sequencer using SMRT technology in medical area. Hum Cell 2017;**30**(3):149-161. doi:10.1007/s13577-017-0168-8.
14. Jain M, Olsen HE, Turner DJ, et al. Linear assembly of a human centromere on the Y chromosome. Nat Biotechnol 2018;**36**(4):321-323. doi:10.1038/nbt.4109.
15. Sun L, Gao T, Wang F, et al. Chromosome-level genome assembly of a cyprinid fish Onychostoma macrolepis by integration of nanopore sequencing, Bionano and Hi-C technology. Mol Ecol Resour 2020;**20**(5):1361-1371. doi:10.1111/1755-0998.13190.
16. Bocklandt S, Hastie A, Cao H. Bionano Genome Mapping: High-Throughput, Ultra-Long Molecule Genome Analysis System for Precision Genome Assembly and Haploid-Resolved Structural Variation Discovery. Adv Exp Med Biol 2019;**1129**:97-118. doi:10.1007/978-981-13-6037-4_7.
17. Koren S, Walenz BP, Berlin K, et al. Canu: scalable and accurate long-read assembly via adaptive k-mer weighting and repeat separation. Genome Res 2017;**27**(5):722-736. doi:10.1101/gr.215087.116.
18. Du H, Liang C. Assembly of chromosome-scale contigs by efficiently resolving repetitive sequences with long reads. Nat Commun 2019;**10**(1):5360. doi:10.1038/s41467-019-13355-3.
19. Li H, Durbin R. Fast and accurate short read alignment with Burrows-Wheeler transform. Bioinformatics 2009;**25**(14):1754-60. doi:10.1093/bioinformatics/btp324.
20. Li H, Handsaker B, Wysoker A, et al. The Sequence Alignment/Map format and SAMtools. Bioinformatics 2009;**25**(16):2078-9. doi:10.1093/bioinformatics/btp352.
21. Walker BJ, Abeel T, Shea T, et al. Pilon: an integrated tool for comprehensive microbial variant detection and genome assembly improvement. Plos One 2014;**9**(11):e112963. doi:10.1371/journal.pone.0112963.
22. Durand NC, Shamim MS, Machol I, et al. Juicer Provides a One-Click System for Analyzing Loop-Resolution Hi-C Experiments. Cell Syst 2016;**3**(1):95-8. doi:10.1016/j.cels.2016.07.002.
23. Dudchenko O, Batra SS, Omer AD, et al. De novo assembly of the Aedes aegypti genome using Hi-C yields chromosome-length scaffolds. Science 2017;**356**(6333):92-95. doi:10.1126/science.aal3327.
24. Servant N, Varoquaux N, Lajoie BR, et al. HiC-Pro: an optimized and flexible pipeline for Hi-C data processing. Genome Biol 2015;**16**(1). doi:10.1186/s13059-015-0831-x.
25. Bolger AM, Lohse M, Usadel B. Trimmomatic: a flexible trimmer for Illumina sequence data. Bioinformatics 2014;**30**(15):2114-20. doi:10.1093/bioinformatics/btu170.
26. Grabherr MG, Haas BJ, Yassour M, et al. Full-length transcriptome assembly from RNA-Seq data without a reference genome. Nat Biotechnol 2011;**29**(7):644-52. doi:10.1038/nbt.1883.
27. Huang Y, Niu B, Gao Y, et al. CD-HIT Suite: a web server for clustering and comparing biological sequences. Bioinformatics 2010;**26**(5):680-2. doi:10.1093/bioinformatics/btq003.
28. Dobin A, Davis CA, Schlesinger F, et al. STAR: ultrafast universal RNA-seq aligner. Bioinformatics 2013;**29**(1):15-21. doi:10.1093/bioinformatics/bts635.
29. Seppey M, Manni M, Zdobnov EM. BUSCO: Assessing Genome Assembly and Annotation Completeness. Methods Mol Biol 2019;**1962**:227-245. doi:10.1007/978-1-4939-9173-0_14.
30. Lu L, Chen Y, Wang Z, et al. The goose genome sequence leads to insights into the evolution of waterfowl and susceptibility to fatty liver. Genome Biol 2015;**16**:89. doi:10.1186/s13059-015-0652-y.
31. Benson G. Tandem repeats finder: a program to analyze DNA sequences. Nucleic Acids Res 1999;**27**(2):573-80. doi:10.1093/nar/27.2.573.
32. Wang Y, Tang H, Debarry JD, et al. MCScanX: a toolkit for detection and evolutionary analysis of gene

synteny and collinearity. Nucleic Acids Res 2012;**40**(7):e49. doi:10.1093/nar/gkr1293.

33. Stamatakis A. RAxML version 8: a tool for phylogenetic analysis and post-analysis of large phylogenies. Bioinformatics 2014;**30**(9):1312-3. doi:10.1093/bioinformatics/btu033.
34. Sanderson MJ. r8s: inferring absolute rates of molecular evolution and divergence times in the absence of a molecular clock. Bioinformatics 2003;**19**(2):301-2. doi:10.1093/bioinformatics/19.2.301.
35. Han MV, Thomas GW, Lugo-Martinez J, et al. Estimating gene gain and loss rates in the presence of error in genome assembly and annotation using CAFE 3. Mol Biol Evol 2013;**30**(8):1987-97. doi:10.1093/molbev/mst100.
36. Yu G, Wang LG, Han Y, et al. clusterProfiler: an R package for comparing biological themes among gene clusters. Omics 2012;**16**(5):284-7. doi:10.1089/omi.2011.0118.
37. Li Y, Gao G, Lin Y, et al. Pacific Biosciences assembly with Hi-C mapping generates an improved, chromosome-level goose genome. Gigascience 2020;**9**(10). doi:10.1093/gigascience/giaa114.
38. Gao G, Gao D, Zhao X, et al. Genome-Wide Association Study-Based Identification of SNPs and Haplotypes Associated With Goose Reproductive Performance and Egg Quality. Front Genet 2021;**12**:602583. doi:10.3389/fgene.2021.602583.
39. Daetwyler HD, Capitan A, Pausch H, et al. Whole-genome sequencing of 234 bulls facilitates mapping of monogenic and complex traits in cattle. Nat Genet 2014;**46**(8):858-65. doi:10.1038/ng.3034.
40. Xi Y, Xu Q, Huang Q, et al. Genome-wide association analysis reveals that EDNRB2 causes a dose-dependent loss of pigmentation in ducks. Bmc Genomics 2021;**22**(1):381. doi:10.1186/s12864-021-07719-7.
41. Nakano N, Maeyama K, Sakata N, et al. C18 ORF1, a novel negative regulator of transforming growth factor-beta signaling. J Biol Chem 2014;**289**(18):12680-92. doi:10.1074/jbc.M114.558981.
42. Cheret J, Bertolini M, Ponce L, et al. Olfactory receptor OR2AT4 regulates human hair growth. Nat Commun 2018;**9**(1):3624. doi:10.1038/s41467-018-05973-0.
43. Balazova L, Balaz M, Horvath C, et al. GPR180 is a component of TGFbeta signalling that promotes thermogenic adipocyte function and mediates the metabolic effects of the adipocyte-secreted factor CTHRC1. Nat Commun 2021;**12**(1):7144. doi:10.1038/s41467-021-27442-x.

Figure legends

Figure 1. A picture of a male adult Lion-head goose.

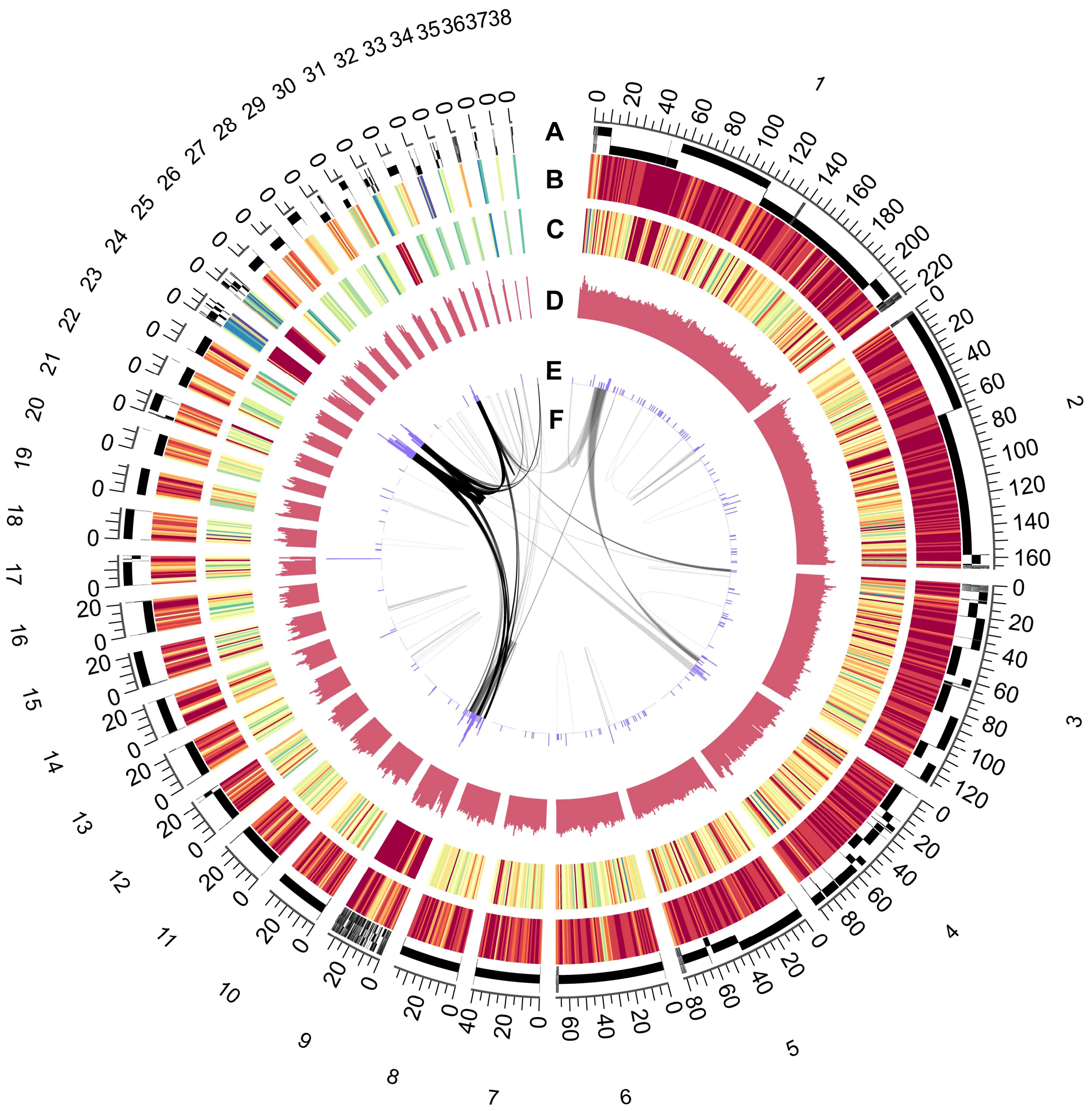

Figure 2. Distribution of genomic features. Concentric circle diagram presents the distribution of genomic features of Lion-head goose using nonoverlapping sliding windows with sizes of 1 Mb (from outmost to innermost). **(A)** the assembled pseudo-chromosome and the corresponding position; **(B)** gene density calculated on the basis of the number of genes; **(C)** average expression level of overall 36 samples. eight tissues (i.e., brain, pharyngeal pouch, head sarcoma, spleen, liver, chest muscle, kidney and heart) and blood collected from four healthy adult animals; **(D)** GC content; **(E)** density of TE; **(F)** gene synteny and collinearity analysis.

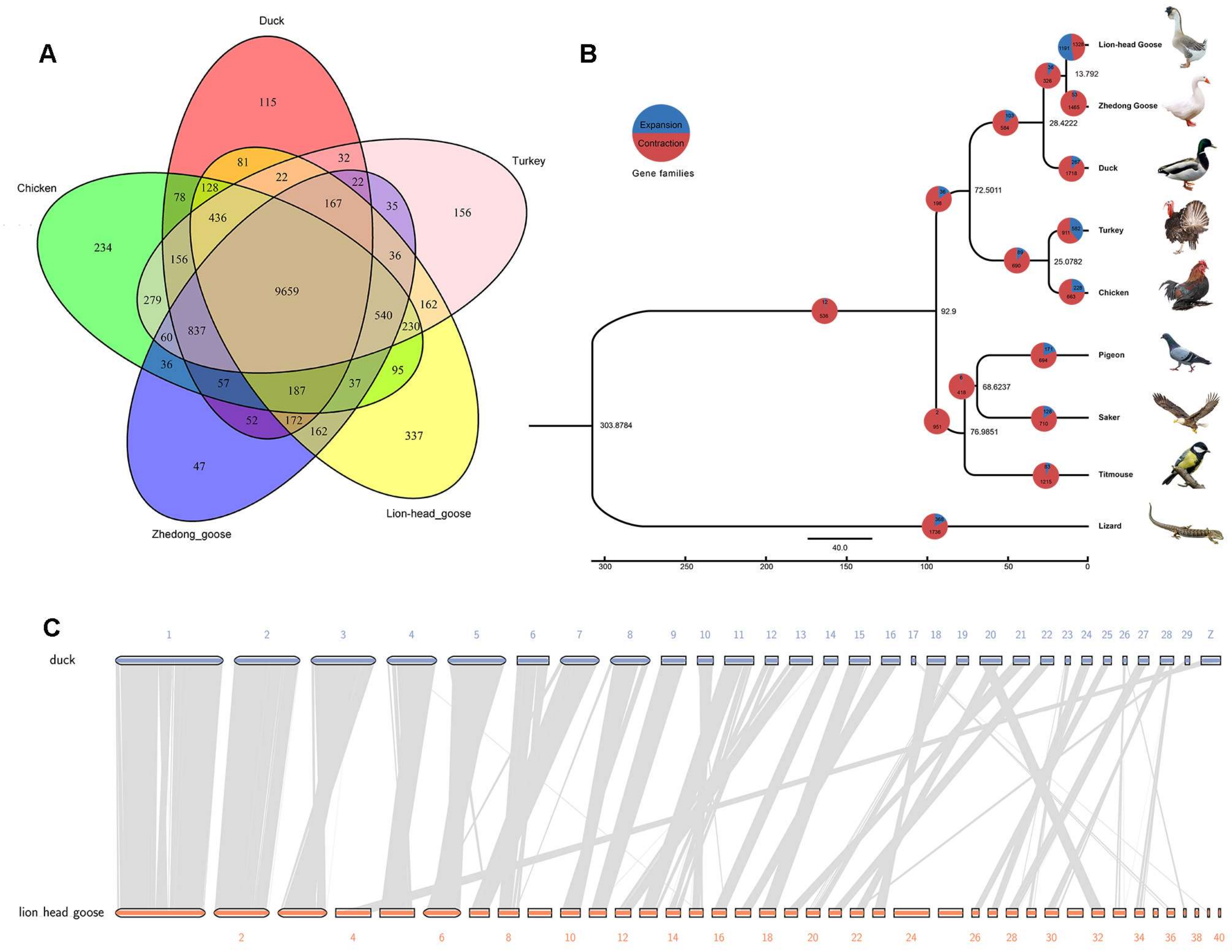

Figure 3. Phylogenetic relationship and comparative genomics analyses. (A) Venn diagram showing the orthologous gene families shared among the genomes of Lion-head goose, Zhedong white goose, chicken, duck, and turkey. **(B)** Phylogenetic tree with the divergence times and history of orthologous gene families. Numbers on the nodes represent divergence times. The numbers of gene families that expanded (green) or contracted (red) in each lineage after speciation are shown on the circles of the corresponding branch. **(C)** Gene comparison of homologous chromosomes between Lion-head goose and duck. Gray lines indicate collinearity between the genomes.

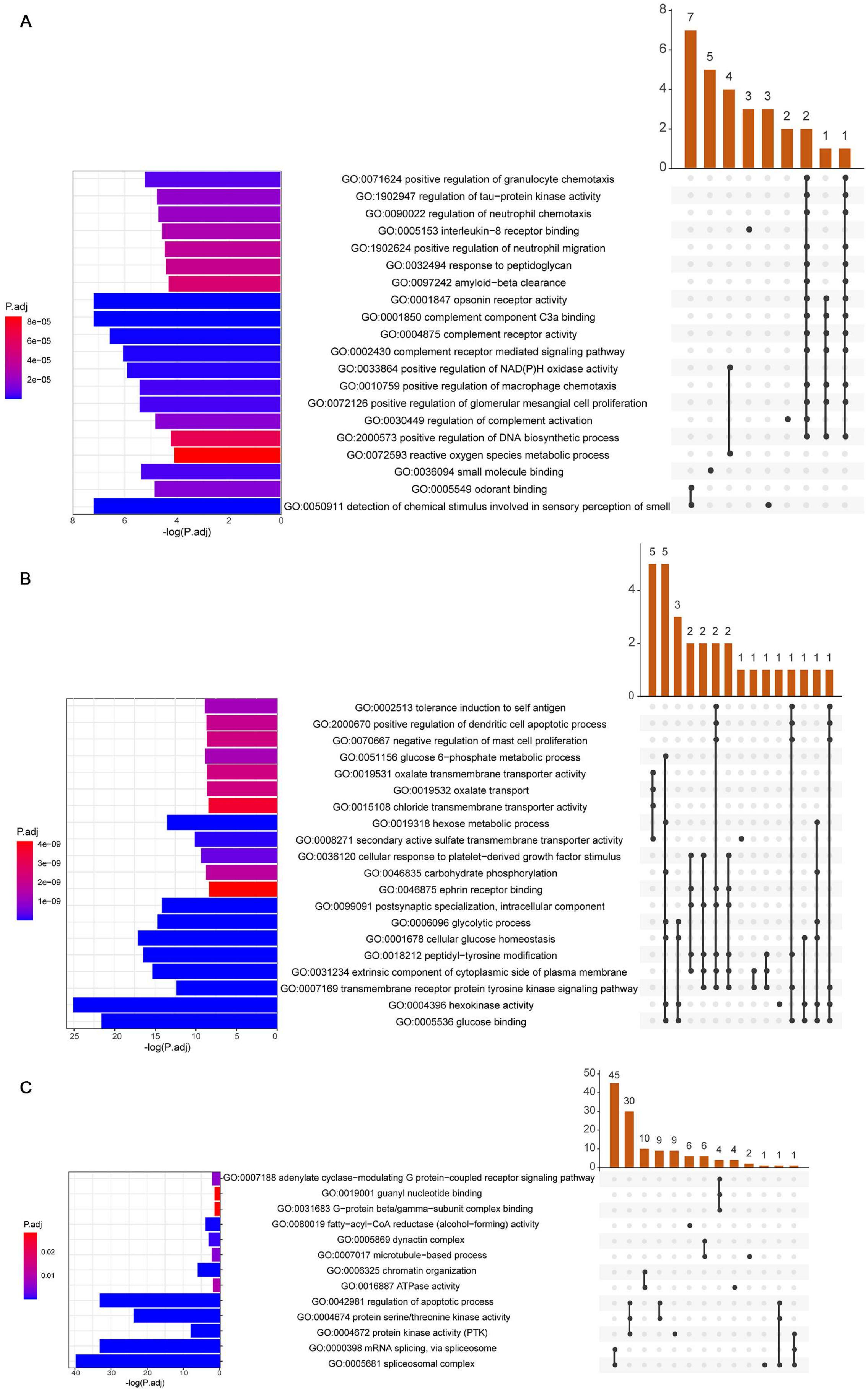
A
P.adj
8e−05
6e−05
4e−05
2e−05
-log(P.adj)
GO:0071624 positive regulation of granulocyte chemotaxis
GO:1902947 regulation of tau−protein kinase activity
GO:0090022 regulation of neutrophil chemotaxis
GO:0005153 interleukin−8 receptor binding
GO:1902624 positive regulation of neutrophil migration
GO:0032494 response to peptidoglycan
GO:0097242 amyloid−beta clearance
GO:0001847 opsonin receptor activity
GO:0001850 complement component C3a binding
GO:0004875 complement receptor activity
GO:0002430 complement receptor mediated signaling pathway
GO:0033864 positive regulation of NAD(P)H oxidase activity
GO:0010759 positive regulation of macrophage chemotaxis
GO:0072126 positive regulation of glomerular mesangial cell proliferation
GO:0030449 regulation of complement activation
GO:2000573 positive regulation of DNA biosynthetic process
GO:0072593 reactive oxygen species metabolic process
GO:0036094 small molecule binding
GO:0005549 odorant binding
GO:0050911 detection of chemical stimulus involved in sensory perception of smell
B
P.adj
4e−09
3e−09
2e−09
1e−09
-log(P.adj)
GO:0002513 tolerance induction to self antigen
GO:2000670 positive regulation of dendritic cell apoptotic process
GO:0070667 negative regulation of mast cell proliferation
GO:0051156 glucose 6−phosphate metabolic process
GO:0019531 oxalate transmembrane transporter activity
GO:0019532 oxalate transport
GO:0015108 chloride transmembrane transporter activity
GO:0019318 hexose metabolic process
GO:0008271 secondary active sulfate transmembrane transporter activity
GO:0036120 cellular response to platelet−derived growth factor stimulus
GO:0046835 carbohydrate phosphorylation
GO:0046875 ephrin receptor binding
GO:0099091 postsynaptic specialization, intracellular component
GO:0006096 glycolytic process
GO:0001678 cellular glucose homeostasis
GO:0018212 peptidyl−tyrosine modification
GO:0031234 extrinsic component of cytoplasmic side of plasma membrane
GO:0007169 transmembrane receptor protein tyrosine kinase signaling pathway
GO:0004396 hexokinase activity
GO:0005536 glucose binding
C
P.adj
0.02
0.01
-log(P.adj)
GO:0007188 adenylate cyclase−modulating G protein−coupled receptor signaling pathway
GO:0019001 guanyl nucleotide binding
GO:0031683 G−protein beta/gamma−subunit complex binding
GO:0080019 fatty−acyl−CoA reductase (alcohol−forming) activity
GO:0005869 dynactin complex
GO:0007017 microtubule−based process
GO:0006325 chromatin organization
GO:0016887 ATPase activity
GO:0042981 regulation of apoptotic process
GO:0004674 protein serine/threonine kinase activity
GO:0004672 protein kinase activity (PTK)
GO:0000398 mRNA splicing, via spliceosome
GO:0005681 spliceosomal complex

Figure 4. GO enrichment analysis of gene families. (A) Expanded and **(B)** contracted gene families from Anatidae varieties (duck, Zhedong white goose, Lion-head goose). **(C)** Unique gene families from the Lion-head goose. The bar graph on the left represents the P-adjust gradient of GO terms, and the color corresponds to the number on the x-axis (i.e. -log (P.adj)). The bluer the color is, the smaller the P-adjust is, and the more significant it is. The redder the color is, the larger the P-adjust is, and the less significant it is. The upper right bar chart exhibits that several genes act together on the terms below. The lower right chart displays the intersection of the genes of each term; the dots connected by lines represent the intersection of multiple terms; the black dots represent "yes", and the gray dots represent "no".

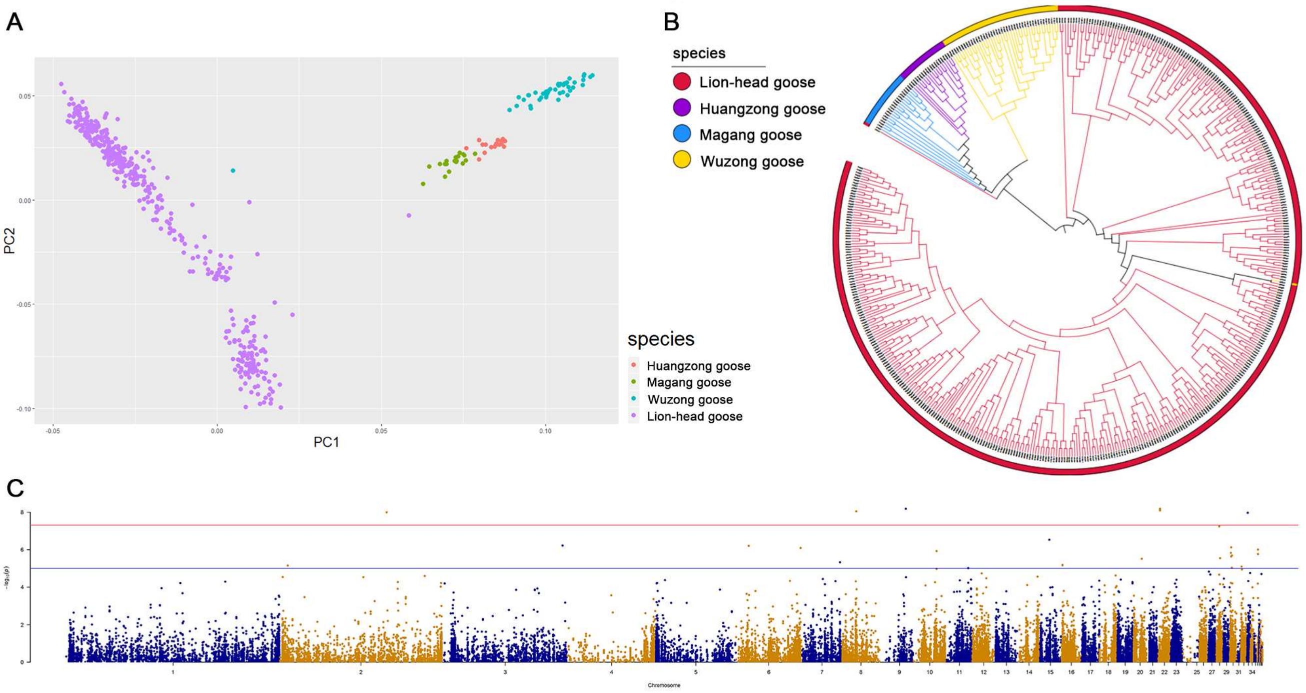

Figure 5. Comparison of different goose species and genome-wide association analysis of body weight. (A) Principal component analysis of sample structures using first two principal components. **(B)** The phylogenetic trees of several goose species. **(C)** Manhattan plot of genome-wide association analysis for body weight. The X-axis indicates chromosomes, and Y-axis indicates the P values of the SNP markers. The red solid line indicates the threshold P value for genome-wide significance. The blue solid line indicates the threshold P value for the significance of potential association.

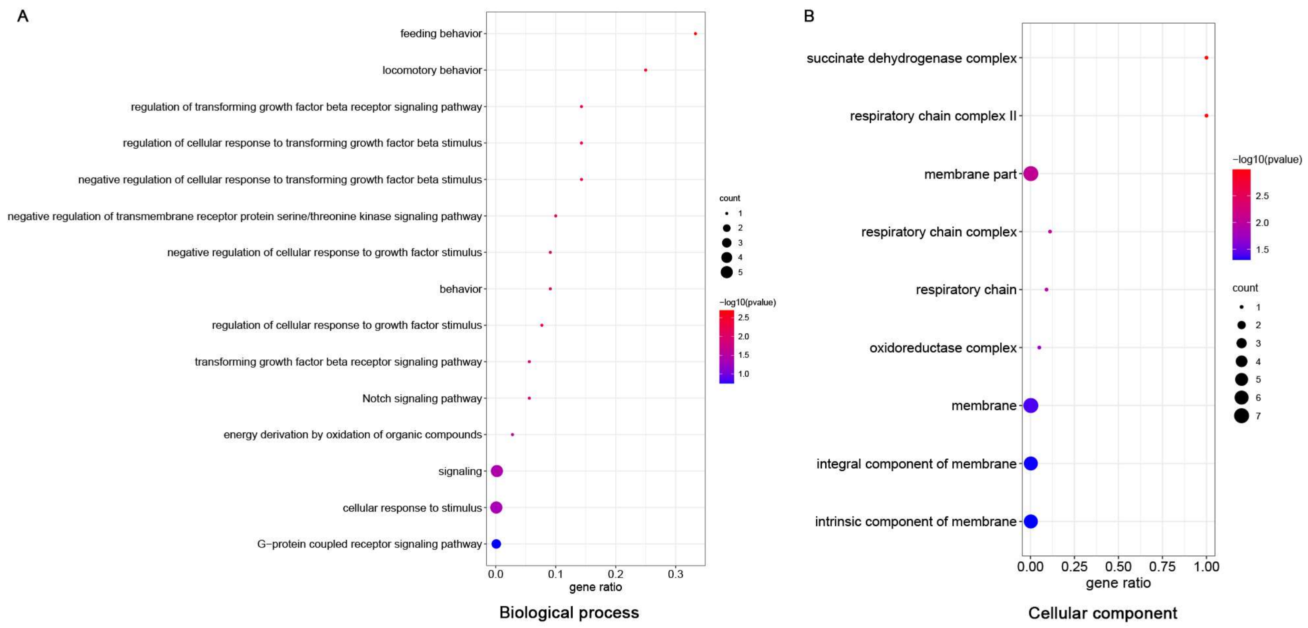

Figure 6. GO analysis of body weight-related genes:(A) Biological processes level, (B) Cellular component level.

Table 1: Summary of repeat classification.

Type	Length	Percent
Long interspersed nuclear element	76,437,757	5.98
Simple sequence repeats	23,026,311	1.80
Low complexity	4,663,288	0.36
Tandem repeats	52,426,380	4.10
Total	156,553,736	12.25

Table 2: Comparison of the present study with previous quality metrics of goose genome assembly.

Genomic features	Lion-head goose	Zhedong white goose	Sichuan white goose	Tianfu goose
Estimate of genome size (bp)	1,278,045,811	1,208,661,181	1,198,802,839	1,277,099,016
Total length of contigs (bp)	1,268,074,106	1,086,838,604	1,100,859,441	1,113,842,245
Total length of scaffolds (bp)	1,277,289,474	1,122,178,121	1,130,663,797	1,113,913,845
Number of contigs	1,318	60,979	53,336	2,771
Number of scaffolds	1,266	1,050	1,837	2,055
Contig N50 (bp)	21,589,146	27,602	35,032	1,849,874
Scaffold N50 (bp)	27,064,542	5,202,740	5,103,766	33,116,532
Longest contig (bp)	91,420,268	201,281	399,111	10,766,871
Longest scaffold (bp)	98,160,899	24,051,356	20,207,557	70,896,740
GC content	42.39%	38.00%	41.68%	42.15%
No. of predicted protein-coding genes	21,010	16,150	16,288	17,568
Percentage of repeat sequences	12.25%	6.33%	6.90%	8.67%

Table 3: Descriptive statistical of body weight traits.

Species	Number	Max (Kg)	Min (Kg)	Mean±SEM
Lion-head goose	416	15.70	9.00	13.55±1.97
Magang goose	20	5.50	4.80	5.32±0.36
Huangzong goose	20	4.30	2.70	3.40±0.83
Wuzong goose	44	2.50	1.80	2.24±0.25

Table 4: Genome-wide association analysis of body weight in geese.

Chr	Allele	Physical position	Regression coefficient	P value	Genes
2	A	108496954	-0.1886	1.01E-08	LDLRAD4
2	G	7706165	0.2612	6.98E-06	LDLRAD4
3	T	123032780	-0.3979	6.03E-07	EGF, KBTBD
6	A	13264157	-0.24	6.28E-07	TSPAN
6	T	66027192	0.2127	8.14E-07	IGFN1
7	T	39117443	-0.3131	4.66E-06	—
8	T	14712470	0.1865	8.97E-09	PPEF1
9	T	26883582	-2.7E+12	0	OR
10	C	23997415	-0.3032	1.19E-06	—
10	C	23997399	-0.2542	1.05E-05	—

10	T	23997401	-0.2542	1.05E-05	—
11	A	22838749	0.1548	9.55E-06	—
15	T	10257386	0.2527	2.96E-07	GPR180, GPCPD1
16	A	1477673	-0.1892	6.53E-06	—
16	G	1477679	-0.1891	6.78E-06	—
20	A	8531879	0.151	3.05E-06	—
22	A	1992485	-0.3972	6.51E-09	GALNT, AUTS2
22	A	1992518	-0.3973	7.69E-09	GALNT, AUTS2
22	G	1992501	-0.3974	7.94E-09	GALNT, AUTS2
22	C	1992505	-0.3974	7.94E-09	GALNT, AUTS2
22	C	1992507	-0.3974	7.94E-09	GALNT, AUTS2
22	G	1992515	-0.3974	7.94E-09	GALNT, AUTS2
28	C	3587271	0.2936	5.81E-08	PPP1R15B, FGD2
28	G	4472051	-0.2359	2.82E-06	PPP1R15B, FGD2
30	C	1652158	-0.3469	7.53E-07	SH2
30	T	1258517	0.2205	1.48E-06	SH2
30	G	2422665	0.1894	2.04E-06	SH2
30	T	2422666	0.1894	2.04E-06	SH2
30	A	1652207	-0.3289	2.3E-06	SH2
30	T	2269897	0.211	9.22E-06	SH2
32	G	655318	0.2599	7.95E-06	—
33	A	975487	0.2567	1.07E-08	SDHA
36	A	1523127	-0.3274	9.86E-07	SPRY
36	G	1523132	-0.3216	1.7E-06	SPRY
36	C	1523105	-0.3291	1.72E-06	SPRY

Figure 1

Click here to access/download;Figure;1.jpg

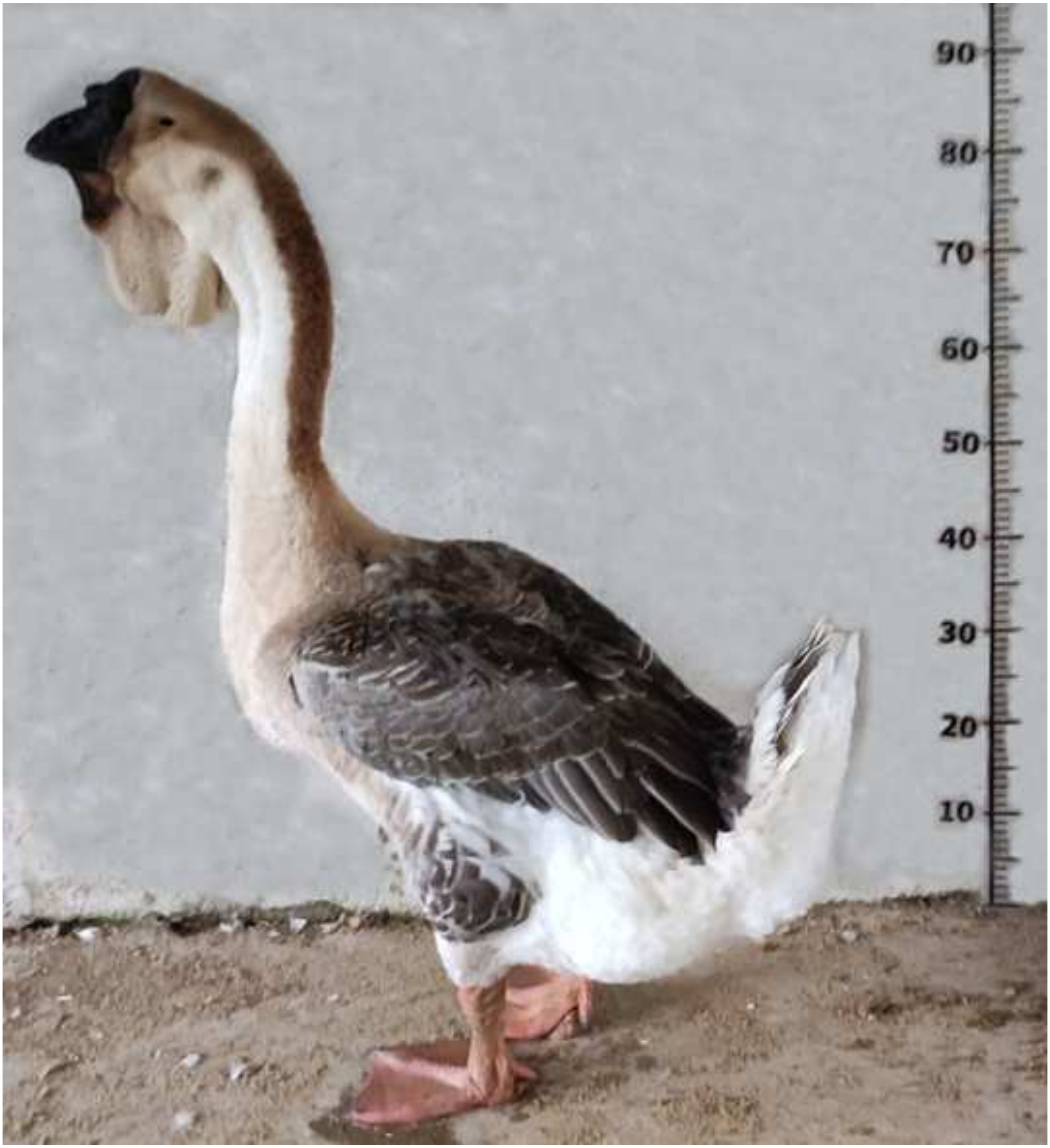

Figure 2

Click here to access/download;Figure;2.tif

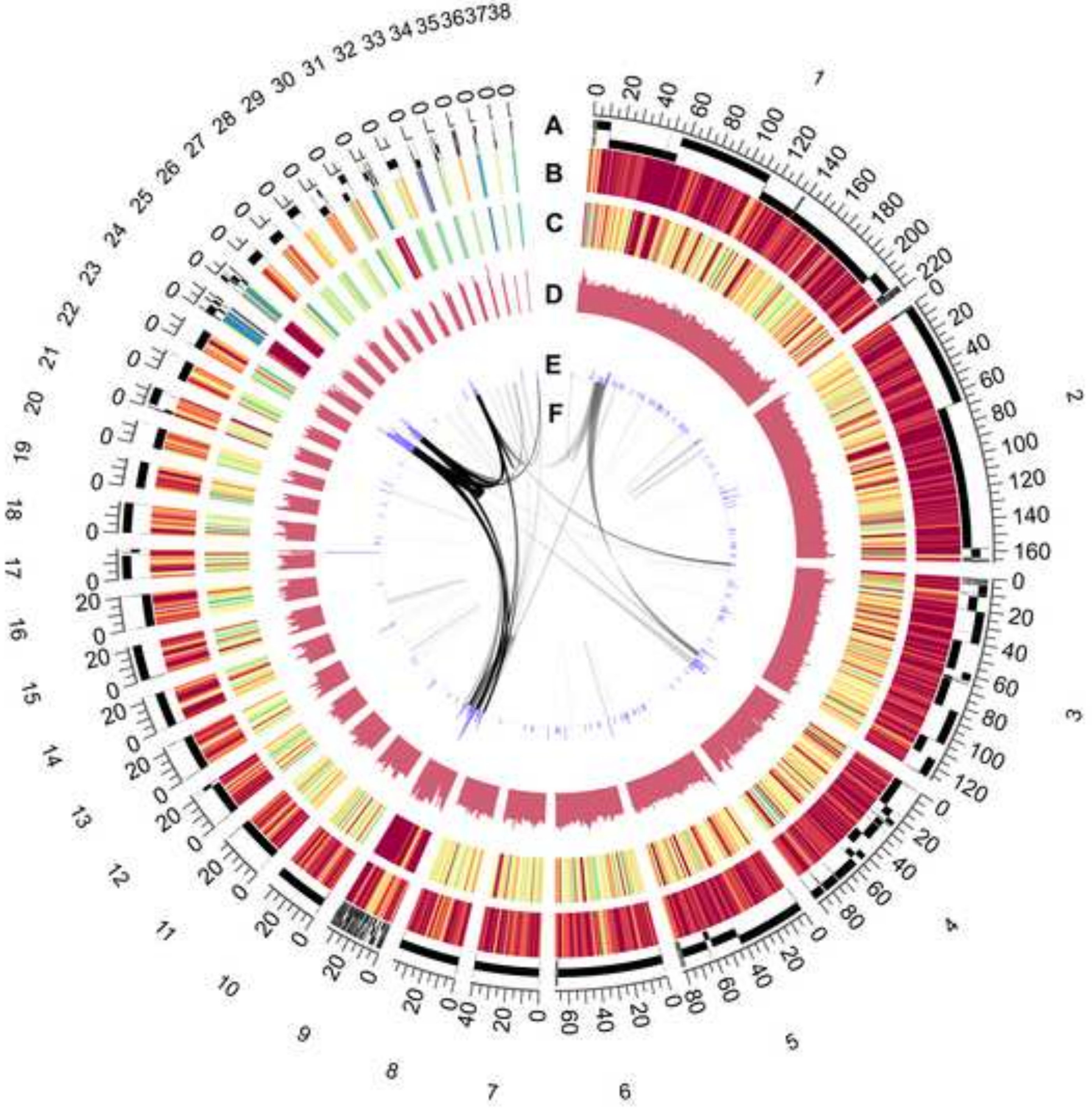

Figure 3

Click here to access/download;Figure;3.tif

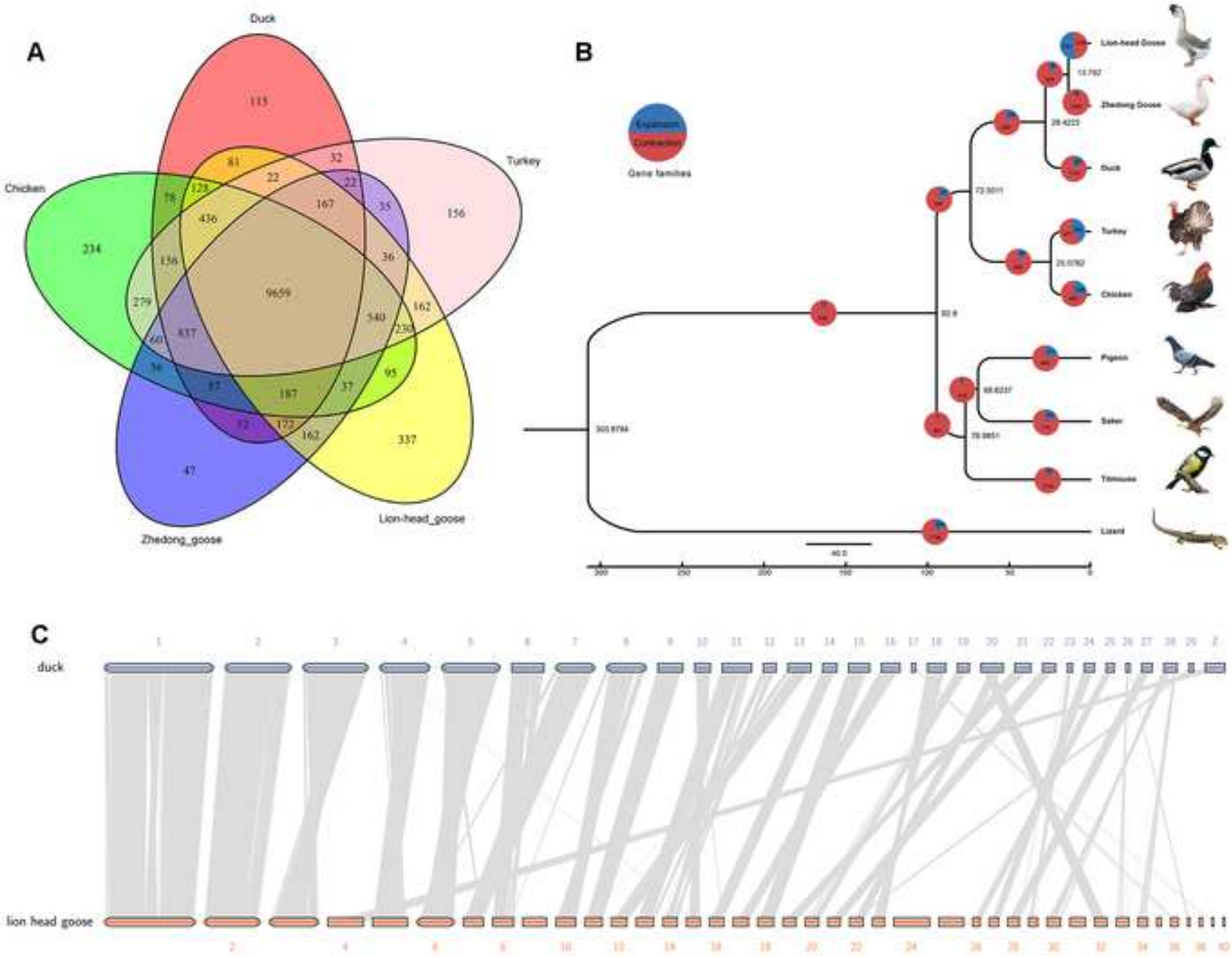

Figure 4

Click here to access/download;Figure;4.tif

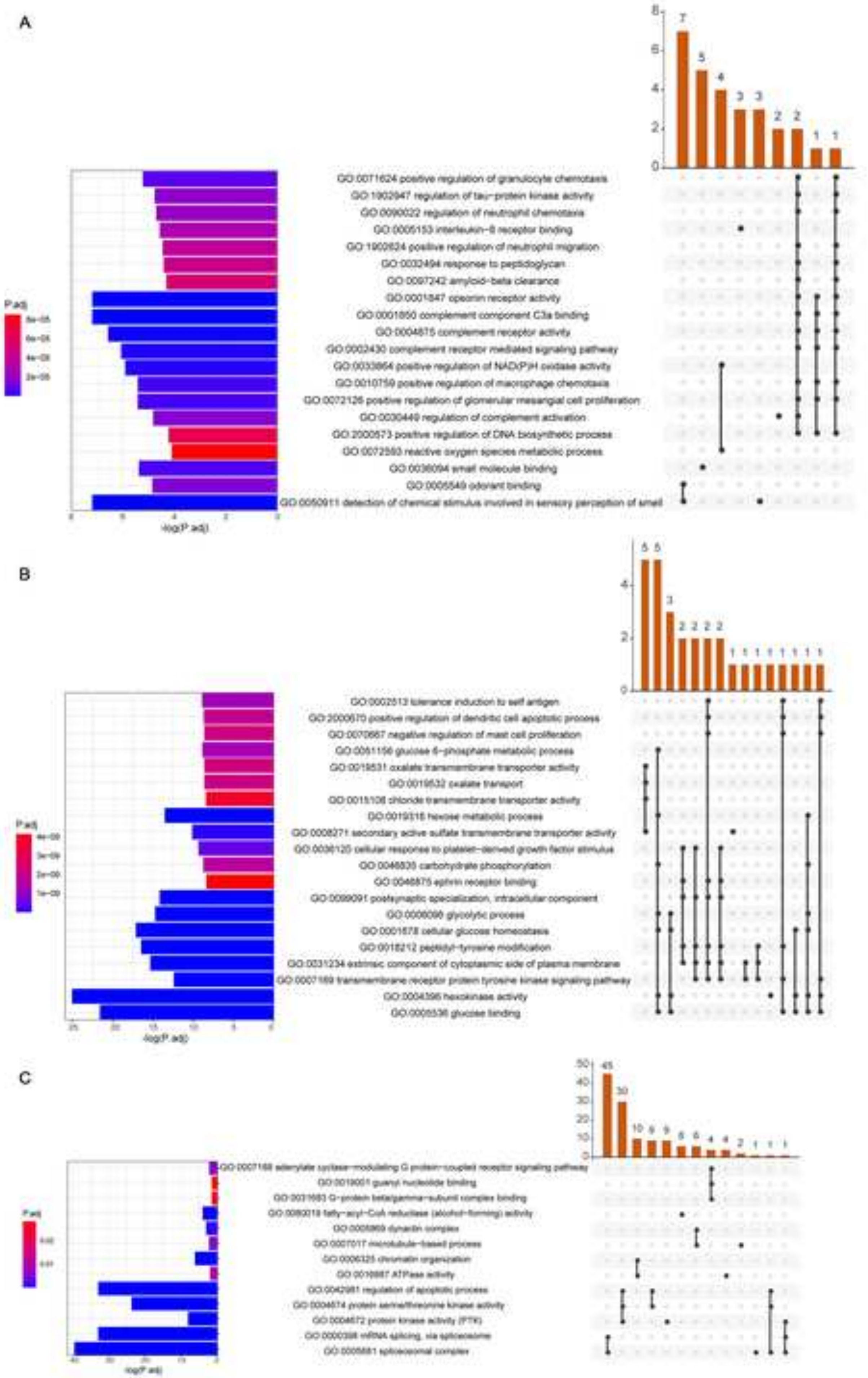

Figure 5

Click here to access/download;Figure;5.tif

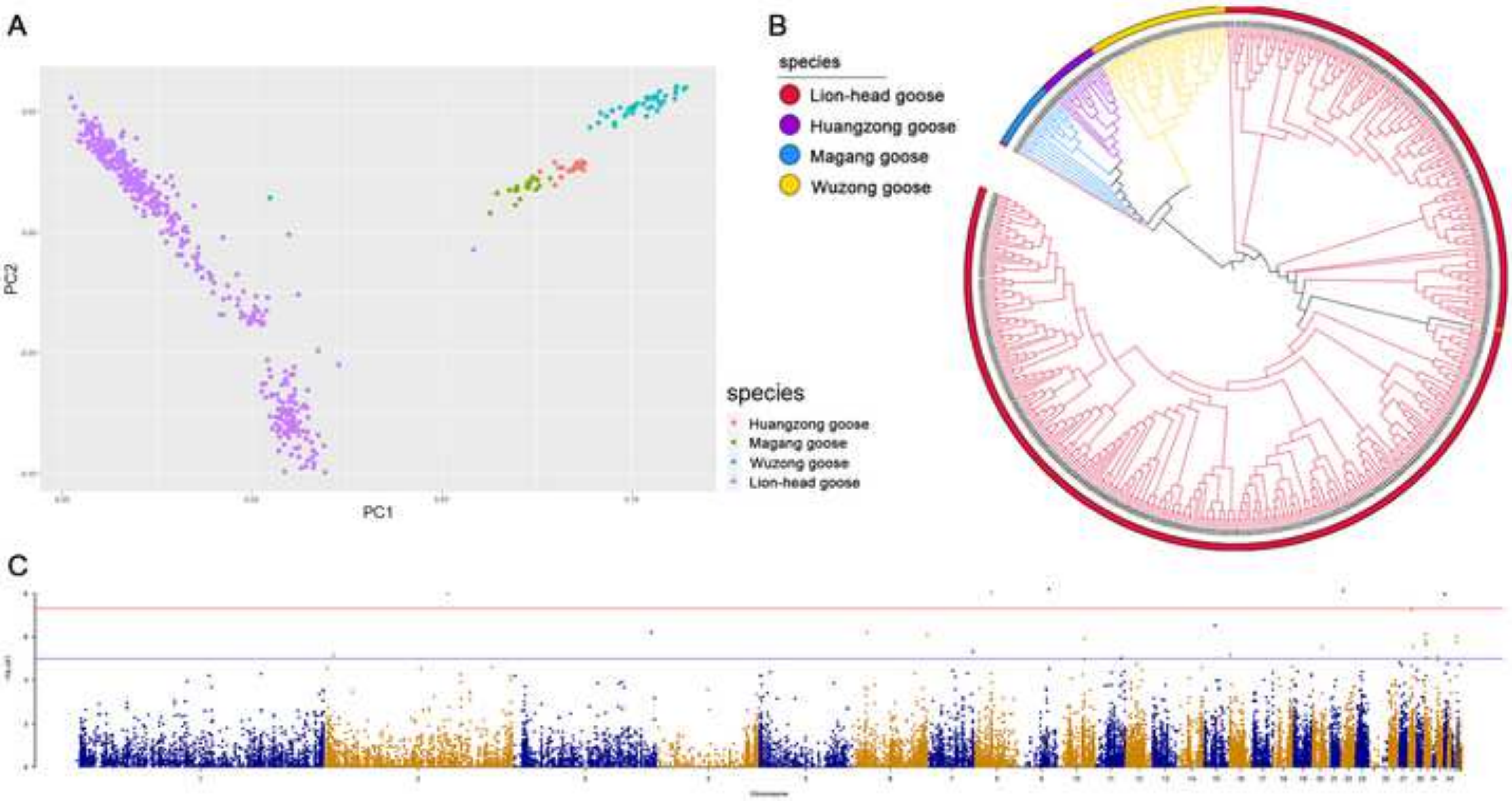

Figure 6

Click here to access/download;Figure;6.tif

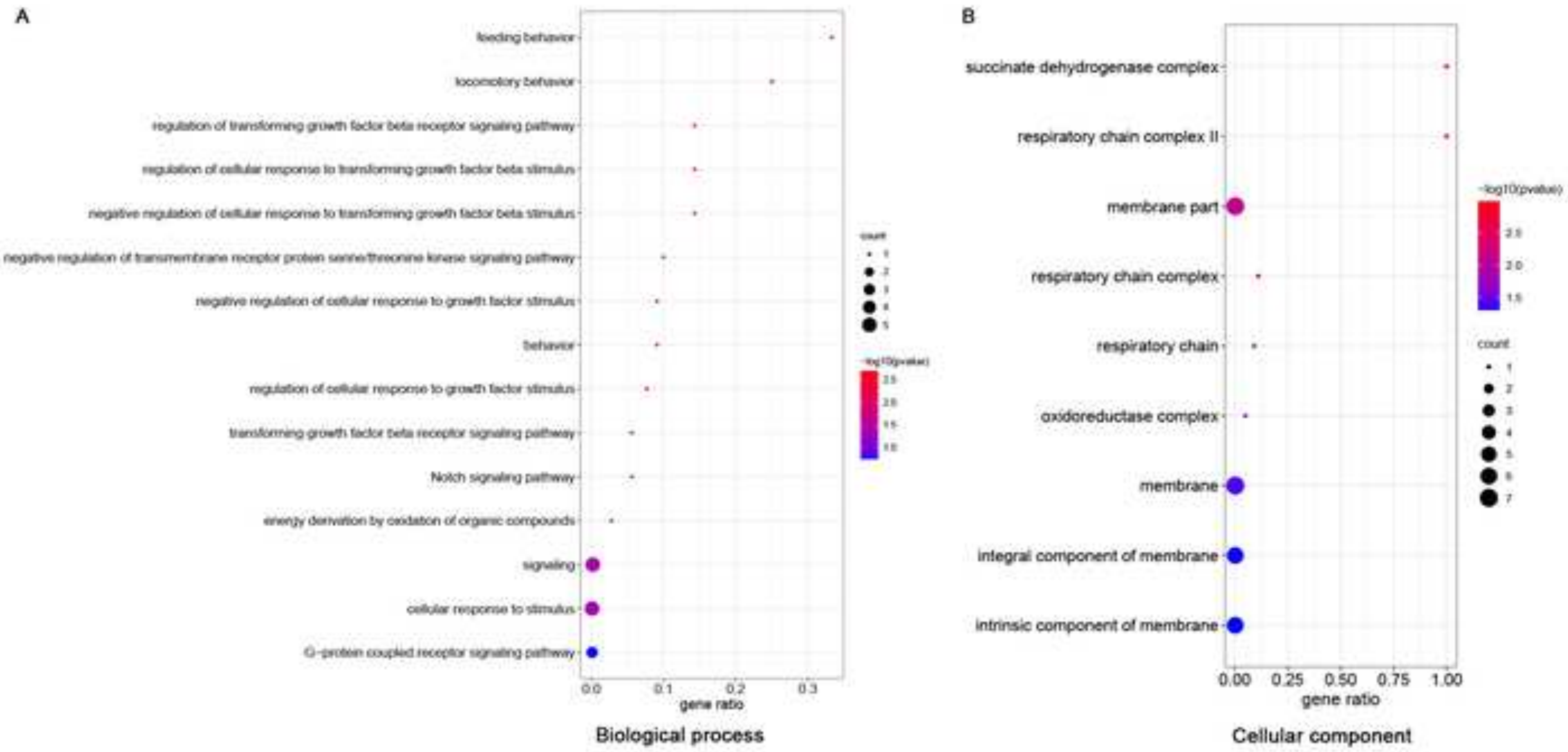

Supplementary data

Click here to access/download
Supplementary Material
10 circos_plot_data.rar

Click here to access/download
Supplementary Material
renamed_1f2a4.docx
