# Supplementary material for: Chromosome-level genome assembly of goose provides insight into the adaptation and growth of local goose breeds
Source: Gigascience. 2023 Feb 3;12:giad003. doi: 10.1093/gigascience/giad003 (PMC9896136; doi:10.1093/gigascience/giad003)
Supplement: giad003_GIGA-D-22-00016_Revision_3 [file giad003_giga-d-22-00016_revision_3.pdf]

GigaScience

Chromosome-level genome assembly of goose provides insight into the adaptation and growth of local goose breeds

--Manuscript Draft--

**Manuscript Number:**	GIGA-D-22-00016R3
**Full Title:**	Chromosome-level genome assembly of goose provides insight into the adaptation and growth of local goose breeds
**Article Type:**	Research
**Funding Information:**	Key Research and Development Program of Guangdong Province (2020B020222001) — Not applicable<br>Construction of Modern Agricultural Science and Technology Innovation Alliance in Guangdong Province (2021KJ128, 2020KJ128) — Not applicable<br>National Modern Agricultural Industry Science and Technology Innovation Center in Guangzhou (2018kczx01) — Not applicable<br>Guangdong Provincial Promotion Project on Preservation and Utilization of Local Breed of Livestock and Poultry (4000-F18260) — Not applicable<br>Guangdong Basic and Applied Basic Research Foundation (2019A1515012006) — Not applicable
**Abstract:**	Backgrouond: Anatidae contains numerous waterfowl species with great economic value, but the genetic diversity basis remains insufficiently investigated. Here, we report a chromosome-level genome assembly of Lion-head goose (Anser cygnoides), a native breed in South China, through the combination of PacBio, Bionano and Hi-C technologies. Findings: The assembly had a total genome size of 1.19 Gb, consisting of 1,859 contigs with an N50 length of 20.59 Mb, generating 40 pseudochromosomes, representing 97.27% of the assembled genome, and identifying 21,208 protein-coding genes. Comparative genomic analysis revealed that geese and ducks diverged approximately 28.42 million years ago, and geese have undergone massive gene family expansion and contraction. To identify genetic markers associated with body weight in different geese breeds including Wuzong goose, Huangzong goose, Magang goose and Lion-head goose, a genome-wide association study was performed, yielding an average of 1,520.6 Mb of raw data with detecting 44,858 SNPs. GWAS showed that six SNPs were significantly associated with body weight and 25 were potentially associated. The significantly associated SNPs were annotated as LDLRAD4 , GPR180 , OR , enriching in growth factor receptors regulation pathways. Conclusions: We present the first chromosome-level assembly of the Lion-head goose genome, which will expand the genomic resources of the Anatidae family, providing a basis for adaptation and evolution. Candidate genes significantly associated with different goose breeds may serve to understand the underlying mechanisms of weight differences.
**Corresponding Author:**	Xinheng Zhang<br>South China Agricultural University<br>Guangzhou, Guangdong CHINA
**Corresponding Author Secondary Information:**	
**Corresponding Author's Institution:**	South China Agricultural University
**Corresponding Author's Secondary Institution:**	
**First Author:**	Qiqi Zhao
**First Author Secondary Information:**	

Powered by Editorial Manager® and ProduXion Manager® from Aries Systems Corporation

**Order of Authors:**	Qiqi Zhao
	Junpeng Chen
	Zi Xie
	Jun Wang
	Keyu Feng
	Wencheng Lin
	Hongxin Li
	Zezhong Hu
	Weiguo Chen
	Feng Chen
	Muhammad Junaid
	Huanmin Zhang
	Zhenping Lin
	Qingmei Xie
	Xinheng Zhang
**Order of Authors Secondary Information:**	
**Response to Reviewers:**	Dear editor,<br><br>Thank you very much for your letter dated 19 Sep 2022, and the reviewer's comments concerning our manuscript "Chromosome-level genome assembly of goose provides insight into the adaptation and growth of local goose breeds" (ID: GIGA-D-22-00016).<br><br>These comments are of great value and very helpful for revising and improving our paper, as well as the importance guiding significant to our research. According to your opinion and request, we have made some revisions to the original manuscript. The responses to the questions are shown below, the black font part is the questions raised by the reviewers, and the dark blue font part is our reply.<br><br>We have resubmitted the revised version in both PDF and MS word format, on the system for your review. The revised parts are marked in yellow in the MS word file of the revised manuscript for your review.<br><br>Should you have any questions, please contact us without hesitate.<br><br>Best wishes,<br>Xinheng Zhang<br><br><br>Questions and Responses:<br><br>Reviewer reports:<br>Reviewer #3: There are still important aspects of your approach which are not clear, and raise doubts as to how the results where produced and are to be interpreted, and also as to your full appreciation of the in's and out's of GWAS models. Please take in serious consideration the remarks below, if needed you can also consult somebody with experience in the theory and practice of GWAS.<br><br>- The specification of the model for GWAS is still wrong, and contains several incorrect statements. Extensive corrections are needed<br>- Also the software implementation for GWAS gives rise to doubts: Plink does not allow for mixed models, so what about your model? There could not be the Z*alpha term, with the associated variance component. Please check carefully, because either the model equation you report in the text (L217) is not the one you used, or you used a different software.

Powered by Editorial Manager® and ProduXion Manager® from Aries Systems Corporation

L138-139: please add more details on how the Perl script hybridScaffold.pl solved the conflict by performing the split
Response: Thank you for your comments, which we have refined as described below: The nature of the conflict is that there are excessive numbers of unmatched markers on the optical and physical maps. To address this problem, hybrid assemblies were processed using Hybrid Scaffold. Firstly, a pattern search of the genomic sequence was performed to find possible cleavage labels, and the number of labels on matched and unmatched pairs in each linkage was counted and their positions was recorded. Supervised processing is then performed to resolve the positions with conflicting match, then RefAligner is called for iterative sequences merge by pairwise alignment. Finally, the sequence map and genome map are re-matched to the hybrid scaffold and checked again.

L156: please choose between "adapter" and "adaptor"
Response: Thank you for your careful guidance, we have checked and corrected the “adaptor” to “adapter” in full manuscript.

L163-165: please report what you mentioned in the response to my comment also in text: explain that a higher ratio of the mapped intact genes in the assembled genome means a higher completeness/quality/integrity of the assembled genome
Response: Thank you for your comment, we have checked carefully and found that this was a calculation error on our part and have made a correction: We then evaluated the assembled genome with 95.21% single-copy and 1.70% duplicated orthologs from the BUSCO dataset, confirming that 8,081 genes (96.92%) were intact in this genome.

L213: you need to add a reference to Plink. Moreover, which version of Plink did you use?
Response: Thanks to your suggestion, we have added the version number (v1.90b6.21) and the reference (Purcell S et al., 2007) to the text.

L214: " ... covariates in the linear model model for the genotype-phenotype association analysis: BW = mu + Zalpha + SNP + e "
L215: delete "The statistical analysis model for genome-wide association analysis was as follows"
L217: in the model equation, I think that you are using matrix notation: this means that mu must be preceded by a vector of 1's, and SNP must be preceded by the corresponding incidence matrix X (indicating for instance the n. of copies of the minor allele in each individual - this is one possible parameterization)
L218: BW is the vector of goose body weights;
L218: Z is not the relationship matrix! Z is the incidence matrix, relating each polygenic effect alpha_i to each individual goose i (probably in your case a diagonal identity matrix). The relationship matrix comes into play in the variance of y, specifically of the polygenic (random multigene) effect alpha --> Var(alpha) = G*sigma_a^2.
L219: the SNP effect should be the SNP effect, i.e. the effect for which you are trying to estimate the magnitude and the significance (is the SNP associated with BW?). The SNP effect is specified with an associated design matrix that relates individuals and genotypes/alleles. If you used principal components as covariates in the GWAS model, you need to add an extra term for this
L220: I is not the unit matrix (a matrix of 1's), but it is the idetity matrix (a diagonal matrix with 1's only in the diagonal)
L220-221: if you used Plink to perform the association analysis, I think that you could not fit the polygenic/mutligene effect, since Plink does not allow to use mixed models with random effects and associated variance components. Please check!!

Response: With regard to the questions relating to GWAS, thank you very much for your professional guidance. In response, we have consulted with the relevant professional technicians and have concluded that there is nothing wrong with our workflow, but only a significant deviation in the textual description in the article, so we have made a complete revision of this section with a view to resolving your confusion. We have sorted out that part of the description, as described below: Based on the SNP set obtained above, the genetic variation was analyzed with individual corresponding body weight information using the two separate and independent models to assess the

	significance of SNP effects in Plink (v 1.90b6.21) [38]. In the first model, top 20 PCs in the PCA analysis were used as covariates, and a linear analysis was performed on sample variances with the following parameters: --linear --allow-extra-chr --allow-no-sex --covar. In the second model, an asymptotic Wald test analysis was carried out with the following parameters: --assoc --allow-extra-chr --allow-no-sex. Finally, SNPs with Bonferroni corrected p-values less than 0.05 were taken as significant loci in the SNPs obtained from the two models above, and these loci were annotated. The annotated genes were subjected to GO enrichment analysis using the genomic genes of Lion-head goose as background.<br>For the statistical model problem, we rechecked the relevant information and found that the model should be<br>Y = b0 + b1. ADD + b2. COV1 + b3. COV2 + ... + b21. COV20 + b22. ADDxCOV1 + b23. ADDxCOV2 + ... b41. ADDxCOV20 + e;<br>where b0~b41 are coefficients, COV x are the top 20 PCs of the PCA as the covariates; e is the residual.<br><br><br>L223: when you use Bonferroni correction, you can either divide the threshold (e.g. alpha = 0.05) by the number of independent tests (e.g. the n. of SNP markers), or you can keep alpha an multiply the p-values obtained from the GWAS model (p-value <= alpha/m). Which one did you do? Did you start with alpha = 10^-6 and then applied Bonferroni correction to this initial threshold? What was the number of test (markers) "m" that you used for correction?<br>Response: Thank you for your comments. We keep alpha a multiply the p-values obtained from the GWAS model (p-value ≤ alpha/m). Number of test (markers) "m" is the number of population SNPs used in GWAS (89716). Bonferroni correction was done by the function p.adjust() in R.
**Additional Information:**	
**Question**	**Response**
Are you submitting this manuscript to a special series or article collection?	No
**Experimental design and statistics**<br><br><br>Full details of the experimental design and statistical methods used should be given in the Methods section, as detailed in our Minimum Standards Reporting Checklist. Information essential to interpreting the data presented should be made available in the figure legends.<br><br><br>Have you included all the information requested in your manuscript?	Yes
**Resources**<br><br><br>A description of all resources used, including antibodies, cell lines, animals and software tools, with enough information to allow them to be uniquely identified, should be included in the Methods section. Authors are strongly	Yes

Powered by Editorial Manager® and ProduXion Manager® from Aries Systems Corporation

encouraged to cite Research Resource Identifiers (RRIDs) for antibodies, model organisms and tools, where possible.<br><br>Have you included the information requested as detailed in our Minimum Standards Reporting Checklist?	
**Availability of data and materials**<br><br>All datasets and code on which the conclusions of the paper rely must be either included in your submission or deposited in publicly available repositories (where available and ethically appropriate), referencing such data using a unique identifier in the references and in the "Availability of Data and Materials" section of your manuscript.<br><br>Have you have met the above requirement as detailed in our Minimum Standards Reporting Checklist?	Yes

Manuscript Click here to access/download;Manuscript;Goose genome-Jan 9.docx

Chromosome-level genome assembly of goose provides insight into the adaptation and growth of local goose breeds

Qiqi Zhao[1,3,5,7+], Zhenping Lin[2+], Junpeng Chen[2], Zi Xie[1,3,5], Jun Wang[4], Keyu Feng[1,3,5], Wencheng Lin[1,3,5,7], Hongxin Li[1,3,5,7], Zezhong Hu[1], Weiguo Chen[1,3,5,7], Feng Chen[1,3,7], Muhammad Junaid[4], Huanmin Zhang[6], Qingmei Xie[1,3,5,7*], Xinheng Zhang[1,3,5,7*]

[1]Heyuan Branch, Guangdong Provincial Laboratory of Lingnan Modern Agricultural Science and technology, College of Animal Science, South China Agricultural University, Guangzhou, Guangdong 510642, China; [2]Shantou Baisha Research Institute of Original Species of Poultry and Stock, Shantou, Guangdong 515000, China; [3]Department of Science and Technology of Guangdong Province, Key Laboratory of Animal Health Aquaculture and Environmental Control, Guangzhou, Guangdong 510642, China; [4]College of Marine Sciences, South China Agricultural University, Guangzhou, Guangdong, 510642, China; [5]Guangdong Engineering Research Center for Vector Vaccine of Animal Virus, Guangzhou, 510642, China; [6]Avian Disease and Oncology Laboratory, Agriculture Research Service, United States Department of Agriculture, East Lansing, MI, 48823, USA and [7]Guangdong Provincial Key Lab of AgroAnimal Genomics and Molecular Breeding, College of Animal Science, South China Agricultural University, Guangzhou, Guangdong 510642, China

* Correspondence address:

Qingmei Xie and Xinheng Zhang, College of Animal Science, South China Agricultural University, Guangzhou, China. E-mails: qmx@scau.edu.cn (Q.X.); xhzhang@scau.edu.cn (X.Z.)

+These authors contributed equally to this work.

running title: Goose chromosome-level Genome Assembly

Qiqi Zhao [0000-0002-7661-9171];

Xinheng Zhang [0000-0001-6409-3160]

Abstract

Backgrouond: *Anatidae* contains numerous waterfowl species with great economic value, but the genetic diversity basis remains insufficiently investigated. Here, we report a chromosome-level genome assembly of Lion-head goose (*Anser cygnoides*), a native breed in South China, through the combination of PacBio, Bionano and Hi-C technologies. **Findings:** The assembly had a total genome size of 1.19 Gb, consisting of 1,859 contigs with an N50 length of 20.59 Mb, generating 40 pseudochromosomes, representing 97.27% of the assembled genome, and identifying 21,208 protein-coding genes. Comparative genomic analysis revealed that geese and ducks diverged approximately 28.42 million years ago, and geese have undergone massive gene family expansion and contraction. To identify genetic markers associated with body weight in different geese breeds including Wuzong goose, Huangzong goose, Magang goose and Lion-head goose, a genome-wide association study was performed, yielding an average of 1,520.6 Mb of raw data with detecting 44,858 SNPs. GWAS showed that six SNPs were significantly associated with body weight and 25 were potentially associated. The significantly associated SNPs were annotated as *LDLRAD4*, *GPR180*, *OR*, enriching in growth factor receptors regulation pathways. **Conclusions:** We present the first chromosome-level assembly of the Lion-head goose genome, which will expand the genomic resources of the *Anatidae* family, providing a basis for adaptation and evolution. Candidate genes significantly associated with different goose breeds may serve to understand the underlying mechanisms of weight differences.

Keywords: Lion-head goose, Genome assembly, Comparative genome, Genome-wide association study

Introduction

The *Anatidae* is a family of the ancient *Aves* class with order *Anseriformes*, containing 43 genuses and 174 species, including most birds of *Anseriformes* order, such as ducks, geese, swans, and is the most prominent family of swimming birds [1]. Physical characteristics and features vary significantly among species, making the *Anatidae* family rich in diversity and specificity. *Anatidae* adults are usually herbivores, feeding on a variety of aquatic plants, which are well suited to sustainable production practices thereby reducing competition for human food; and some species are even used for crop weeds and pests control [1, 2]. For a long time, duck and goose feathers have been popular in pillows, quilts and coats [3]. Several species in the genus *Anser* are commercially important and domesticated as poultry because of their meat-producing performance and natural stuffing for warm clothing and bedding. According to archaeological evidence, geese were domesticated around 6,000 years ago near the Mediterranean Sea, and later spread around the world due to human activities [4]. It is widely believed that *Anser cygnoides* (NCBI:txid8845) is the ancestor of the Chinese goose (*Anser cygnoides domesticus*) with a domestication history of more than 3,000 years [1]. After artificial domestication, the domestic goose has increased its cold tolerance and roughage-resistance, but its wings are degraded and weakened in flight, unable to travel long distances [1]. Egg-laying rate and goslings survival rate are also improved compared to wild swans, and the lifespan is longer [5]. Furthermore, overfeeding can cause foie gras to be at least three-fold larger than the normal size while the goose remains healthy, making the goose a good model to study human liver steatosis [6]. Chinese domestic geese is a natural gene pool containing local breeds of diverse phenotypes, and adult domestic geese from similar region vary greatly in weight [7]. For example, the Lion-head goose in Shantou (116°14'-117°19' E, 23°02'-23°38' N), Guangdong Province, can weigh more than 9 kg, while in the Wuzong goose from Qingyuan (111°55'-113°55' E, 23°31'-25°12' N), Guangdong Province, the average weight is only about 3 kg [8, 9]. The Lion-head goose has a large body, a deep and wide head, and large sarcomas (five sarcomas) on the front and side of the face **(Fig. 1)**. The adult male goose weighs 9-10 kg and the female goose 7.5-9 kg, grows rapidly and has rich muscles. Wuzong goose is a small goose species with a distinct band of

black plumage from neck to back. The gander weighs 3-3.5kg and the female weighs 2.5-3kg, with wide and short body, flat back, and thin and short feet. Magang goose is a medium-sized goose species, with a long head, wide beak, rectangular body, a gray-black bristle-like feathers on the back of the neck, gray brown breast feathers and white belly feathers. Adult weight is 4-5 kg for males and 3-4 kg for females. Huangzong goose has a compact body, from the top of the head to the back of the neck has a brownish yellow feather belt, shaped like a horse's mane. The chest feather is gray yellow, the belly feather is white, the beak and sarcoma is black. Adult males weigh 3-3.5 kg, females 2.5-3 kg. However, the mechanisms for such differences have not been clarified, let alone being resolved at the genomic level. Therefore, a complete, continuous and accurate reference genome is essential, for deciphering genomic diversity, evolutionary and adaptive processes, improving production efficiency and even develop better tools for breeding to promote the development of goose industry.

High-quality genome assembly sequences enable us to comprehensively and scientifically decode the genetic diversity of species, explore disease mechanisms, and understand species evolution. Recently, Pacbio has offered technology that can generate reads several thousand bases in size, and these long reads can span repetitive regions [10]. Although these long reads have a high error rate, they can be integrated with Illumina's short reads to improve sequencing accuracy [11]. In addition, new scaffolding techniques, such as high-throughput chromosome conformation capture (Hi-C), allow the genome to be assembled to the level of whole chromosomes [12]. Pacbio single molecule real-time (SMRT) sequencing technology has been extensively used in the study of human diseases such as tuberculosis and influenza virus [13], as well as in the study of species evolution, such as the centromere of the human Y chromosome [14]. Bionano optical mapping technology has advantages in obtaining highly repetitive sequences and detecting genomic structural variants, which is helpful for remote sequencing of sequence overlap clusters[15]. Bionano has become a powerful tool for genome assembly, a 5.1 Mbp inversion was found in the genomes of a patient with Duchenne muscular dystrophy[16].

In this study, we report the genome assembly at the chromosome level in Lion-head geese for the first time using combined data generated by four advanced technologies, Illumina, SMRT, Bionano, and Hi-C. In addition, we investigated the relationship between body weight and genetic variations in Lion-

head goose, Wuzong goose, Huangzong goose and Magang goose by genome-wide association analysis, trying to identify the genes involved in body weight determination from different species. These will offer valuable resources for facilitating genetic research and the improvement of the species and for studying speciation and evolution in geese.

Methods

Animal selection

An adult healthy purebred male Lion-head goose (*Anser cygnoides*) with classical traits was selected for whole-genome sequencing and conducting *de novo* assembly from Shantou Baisha Research Institute of Original Species of Poultry and Stock. Blood and eight tissues (i.e., brain, pharyngeal pouch, head sarcoma, spleen, liver, chest muscle, kidney, and heart) from another four healthy adult individuals were collected for RNA-seq analysis. All applicable institutional and national guidelines for the care and use of animals were followed. All the animal work in this study was approved by the South China Agricultural University Committee for Animal Experiments (approval ID: SYXK 2019-0136). All the research procedures and animal care activities were conducted based on the principles stated in the National and Institutional Guide for the Care and Use of Laboratory Animals.

Genome survey library construction and sequencing

To survey the genome profile, high-quality genomic DNA was extracted from the blood of the reference individual for whole-genome sequencing using the Qiagen Blood and Cell Culture DNA Midi Kit according to the manufacturer's instructions. For the quality control of purity, concentration, and integrity, we used Qubit 2.0 Fluorometry (Life Technologies, USA), NanoDrop 2000 spectrophotometer (Thermo Scientific), and pulse-field gel electrophoresis (Bio-rad CHEF-DR II), respectively. The following steps used for DNA extraction and quality control were similar. The short paired-end Illumina DNA library was constructed using the Illumina HiSeq X ten system (Illumina HiSeq X Ten, RRID:SCR_016385) with the paired-end 350 bp sequencing strategy. After performing the sequencing and obtaining the data, the k-mer analysis of reads for the genome survey was calculated by the Jellyfish (Jellyfish, RRID:SCR_005491) program with the default parameters. Additionally, the genome size,

heterozygosity ratio, and repeat sequence ratio were calculated with the GenomeScope (GenomeScope, RRID:SCR_017014) tool based on the k-mer frequency of 17.

Genome sequencing and assembly strategies

A 40 kb *de novo* library for SMRT genome sequencing was constructed using the PacBio Sequel II platform (PacBio Sequel II System, RRID:SCR_017990) (Pacific Biosciences, USA). All of these reads were used for contigs assembly. A scalable and accurate long-read assembly tool, Canu (Canu, RRID:SCR_015880) v1.8 [17], was employed to correct and assemble the PacBio reads with the listed parameters (minThreads = 4, genome size = 1200m, minOverlapLength = 700, minReadLength = 1000). The resulting contigs and corrected reads were used as inputs for HERA [18] to fill the gaps and produce longer contigs with default parameters. After that, Illumina paired-end clean data were mapped to the corrected contigs with the Burrows-Wheeler Aligner (BWA, RRID:SCR_010910) [19], and the results were filtered by Q30 with Samtools (SAMTOOLS, RRID:SCR_002105) v1.8 [20]. Finally, Pilon (Pilon, RRID:SCR_014731) v1.22 [21] was used to polish the assembly and enhance the base accuracy of the contigs.

Physical optical genome maps from BioNano were used to improve the assembly quality of the genome, with the ultimate goal of generating a chromosome-scale assembly. Nuclear DNA was extracted from the blood sample of the reference individual and digested with nickase Direct Labeling Enzyme Ⅰ. After labeling, repairing and staining reactions, DNA was loaded onto the Saphyr Chip for sequencing to generate BioNano molecules. Afterward, the data were assembled with RefAligner and Assembler of BioNano Solve. The scaffold was established using BioNano Solve with HERA's contigs and a BioNano genome map. When encountering a conflict between a contig and the BioNano genome map, the contig was split by the program "hybridScaffold.pl" to correct the false connection. In brief, a pattern search of the genomic sequence was first performed to find possible cleavage labels, and the number of labels on matched and unmatched pairs in each linkage was counted and their positions was recorded. Supervised processing is then performed to resolve the positions with conflicting match, then RefAligner is called for iterative sequences merge by pairwise alignment. Finally, the sequence map and genome map are re-matched to the hybrid scaffold and checked again.

For Hi-C library, fresh blood was vacuum-infiltrated with 2% formaldehyde solution and then used for cross-link action. Later nuclear DNA was isolated from the reference animal and digested with the restriction enzyme Mbo I. The Hi-C library with insertion sizes of 350 bp was constructed and sequenced on the Illumina HiSeq X Ten instrument. The Hi-C reads were assigned to the scaffolds by Juicer (Juicer, RRID:SCR_017226) [22]. The scaffolds were further clustered, ordered, and oriented to the chromosome-level scaffolds by 3D-DNA [23]. Thus, a heatmap of Hi-C chromosomal interaction was created using the HiC-pro software (HiC-Pro, RRID:SCR_017643) [24].

RNA-Seq and transcripts assembly

RNA-seq was conducted on blood and eight different tissues (i.e., brain, pharyngeal pouch, head sarcoma, spleen, liver, chest muscle, kidney, and heart) from four healthy adult Lion-head goose. Total RNA was extracted from four individuals using the TRIZOL reagent and purified following the manufacturer's protocols. The concentration and quality of the isolated RNA were assessed using the Nanodrop Spectrophotometer, Qubit 2.0 Fluorometry, and the Agilent 2100 bioanalyzer (Agilent Technologies, USA). Libraries construction and sequencing were performed using the Illumina NovaSeq 6000 platform (Illumina NovaSeq 6000 Sequencing System, RRID:SCR_016387). Raw RNA-seq data with 150 bp paired-end reads were trimmed for quality using Trimmomatic (Trimmomatic, RRID:SCR_011848) [25]. Thus, the Illumina sequence adapters were removed, then low-quality reads based on Phred scores, adapter-polluted reads containing >5 adapter-polluted bases, and those containing N > 5% were trimmed, using the following parameters: LEADING:3 TRAILING:3 SLIDINGWINDOW:4:15 -threads 20 MINLEN:50. Furthermore, Trinity [26] was used to *de novo* assemble the data after quality filtering. To remove redundant sequences, CD-HIT (CD-HIT, RRID:SCR_007105) [27] was employed to remove highly identical transcript isoforms, retaining only the longest one. After filtering, the RNA-seq reads were mapped to the assembled genome using the default parameters of STAR [28].

Assembly evaluation

Finishing the genome assembly, quality control for the assembly's quality, accuracy, and integrity was assessed by Benchmarking Universal Single-Copy Orthologs (BUSCO, RRID:SCR_015008), v 5.3.0,

using aves_odb10 as the query with parameters: -l aves_odb10 -m genome -c 5 [29, 30].

Genome annotation

The genome assembly was annotated by MAKER (MAKER, RRID:SCR_005309), mainly including gene annotation and repeat annotation. The detailed pipeline was based on proteins from the Uniprot, the *de novo* assembly of RNA-seq data, and the total proteins of the relative species *Anser cygnoides* [31]. The transposable elements (TE) associated genes that were filtered out by the TEseeker database, and the results were used to conduct functional annotation using InterProScan. The repeat sequencing library was identified and annotated by a combination of LTR-FINDER and RepeatModeler (RepeatModeler, RRID:SCR_015027). RepeatMasker and the query species "Chicken" were used to mask the repeats in the assembly, based on the Repbase database and the previous repeat sequence library. Tandem repeats were discovered by the Tandem Repeats Finder [32].

Gene families and phylogenetic analysis

Interspecific syntenic blocks between the Lion-head goose and duck were explored using MCscan (MCScan, RRID:SCR_017650) [33] after coding sequence alignment by BLASTn (BLASTN, RRID:SCR_001598). The same method was used for intraspecific collinearity analysis. To gain insight into the gene family evolution of the goose, we compared the gene families of Lion-head goose with the genomes of the following avian species: Zhedong white goose (*Anser cygnoides*), duck (*Anas platyrhynchos*), turkey (*Meleagris gallopavo*), chicken (*Gallus gallus*), pigeon (*Columba livia*), saker (*Falco cherrug*), titmouse (*Pseudopodoces humilis*), and green lizard (*Anolis carolinensis*). Initially, alternative splicing and genes encoding less than 50 amino acids with a proportion of stop codon greater than 20% were filtered; meanwhile, the longest transcript of genes with multiple isoforms was retained to represent the gene. Similarity relationships among the protein sequences of species were aligned by BLASTP (BLASTP, RRID:SCR_001010) algorithm and clustered using OrthoMCL methodology with an expansion coefficient of 1.5 to obtain single- and multiple-copy gene families, and specific gene families of Lion-head goose. The sequences of the single-copy gene families were employed to perform multiple alignments by MUSCLE (MUSCLE,

RRID:SCR_011812). Then RAxML (RAxML, RRID:SCR_006086) [34] was used to construct a phylogenetic tree of nine species, with the green lizard (*Anolis carolinensis*) being designated an outgroup. Taking the divergence time of the pigeon and turkey (92.9Mya) as the calibration, the r8s (r8s, RRID:SCR_021161) [35] software was used to estimate the divergence time of the species and construct ultrametric trees. After filtering out gene families with gene counts of more than 100 in some individual species, CAFÉ (CAFE, RRID:SCR_005983) [36] was employed to detect gene families that had undergone expansion or contraction per million years independently along each branch of the phylogenetic tree. Subsequently, a gene ontology (GO) enrichment analysis of gene families was performed using the clusterProfiler package in R [37].

Experimental sample processing and variant detection for Genome-wide association study

Blood samples of 514 geese (including Lion-head goose, Wuzong goose, Huangzong goose and Magang goose) were collected and stored in 2 mL tubes containing ACD anticoagulant for DNA extraction, and the weight of the geese was recorded. DNA was extracted from blood samples using the HiPure Blood DNA Mini Kit (Magenbio, Guangzhou, China). The samples that passed the quality testing were subjected to library construction using Easy DNA Library Prep Kit (MGI, Shenzhen, China) and paired-end 100 sequencing using BGIseq 500 (BGISEQ-500, RRID:SCR_017979). Raw data were filtered for adapters and low quality reads using SOAPnuke software, low quality threshold parameters set to 20, and the filtered sequences were compared with the constructed goose reference genome using BWA software with parameters: mem, -M. Then variant detection was performed using Samtools, GATK4 software with parameters: HaplotypeCaller –ERC GVCF. SNP variants were filtered based on a minimum allele frequency threshold of 0.05, a Hardy Weinberg equilibrium test significance threshold of 10^{-7}, and a max missing rate threshold of 0.7. Principal component analysis (PCA) was performed and plotted with R. To understand relationships among groups of the samples, the phylogenetic trees were constructed using SNP data with Phylip software.

Genome-wide association study

Based on the SNP set obtained above, the genetic variation was analyzed with individual corresponding body weight information using the two separate and independent models to assess the significance of

SNP effects in Plink (PLINK, RRID:SCR_001757) v1.90b6.21 [38]. In the first model, top 20 PCs from the PCA analysis were used as covariates, and a linear analysis was performed on sample variances with the following parameters: --linear --allow-extra-chr --allow-no-sex --covar. In the second model, an asymptotic Wald test analysis was carried out with the following parameters: --assoc --allow-extra-chr --allow-no-sex. Finally, SNPs with Bonferroni corrected p-values less than 0.05 were taken as significant loci in the SNPs obtained from the two models above, and these loci were annotated. The annotated genes were subjected to GO enrichment analysis using the genomic genes of Lion-head goose as background.

Selective-sweep analysis

To analyze regions affected by long-term selection and are associated with domestication of geese, we calculated the Fixation indices (F_{ST}) for four goose species using vcftools software with sliding windows length of 20 kb that had a 10-kb overlap between adjacent windows. The top 5% of regions were designated as candidate selective regions and the genes in these regions were considered as candidate genes.

Results

Genome sequencing and assembly

The Lion-head goose is a famous local variety in China and one of the most giant goose breeds worldwide, with a unique appearance and social benefits. Here, we attempt to construct a highly continuous chromosome-scale genome of an adult purebred male Lion-head goose with a high degree of homozygosity to minimize heterozygous alleles. The following sequencing and genome assemble strategies were applied: Illumina sequencing, Pacbio SMRT sequencing, BioNano optical mapping, and Hi-C approach **(Supplementary Table S1)**. We assemble these data step by step and generate progressively improved assembled genome **(Supplementary Figure S1)**. A total of 185.37 Gb of high-quality Pacbio long reads were generated, representing a ~168× depth of the estimated 1.05 Gb genome with heterozygosity of 0.335% based on the k-mer analysis of the Illumina sequences **(Supplementary Figure S1, Supplementary Table S2)**. Combing the *de novo* assembly of the Illumina and Pacbio

sequences resulted in a draft genome of 1.20 Gb, yielding 1,859 contigs with a length of 13.7 Mb for contig N50 and 57.6 Mb for the longest **(Table 1)**. Furthermore, with the help of BioNano optical mapping, the scaffold N50 value was increased to 37 Mb. To obtain a chromosome-scale assembly, a set of ~230 Gb Hi-C data was used to orient, order, phase, and anchor the contigs. Approximately 97.27% of the reads assembled were anchored to 40 high-confidence pseudo-chromosomes (39 autosomes and Z chromosome) using the high-density genetic map **(Supplementary Figure S1, Fig. 2)**. After polishing, we finally assembled the ultimate genome into 1.19 Gb with the final contig N50 of 20.59 Mb and scaffold N50 of 25.8 Mb, with a GC content of 42.39% **(Supplementary Table S2 and S3)**. The structure and quality of the assembled genome were determined by mapping a Hi-C chromosomal contact map.

The completeness of the Lion-head goose genome assembly was assessed using the BUSCO gene set. The result showed that almost 99.02% of the reads were correctly mapped to the genome. We then evaluated the assembled genome with 95.21% single-copy and 1.70% duplicated orthologs from the BUSCO dataset, confirming that 8,081 genes (96.92%) were intact in this genome. These results indicate the high reliability and integrity of the assembled genome **(Supplementary Figure S2 and Table S4)**.

Genome annotation

To support the genome annotation, we conducted RNA-Seq analysis using RNA samples of blood and eight tissues (brain, pharyngeal pouch, head sarcoma, spleen, liver, chest muscle, kidney, and heart) from four healthy adult individuals. The aggregate of 760 Gb raw reads was accumulated by the paired-end sequencing of the 36 constructed libraries. After filtering the adapter and low-quality sequences, 723 Gb qualified Illumina reads remained, *de novo* assembled into unique transcripts (unigenes). Overall, a total of 216,229 unigenes were assembled and at the level N50, 5,082 nucleotides were obtained. Total 21,208 protein-coding gene annotations were predicted in Lion-head goose by combining *de novo* prediction, homologous protein prediction, and transcription alignment. After filtering TE-related genes, a total of 21,010 protein-coding gene annotations were finally obtained by the TE seeker database **(Fig. 2)**. Furthermore, a total of 8.15% repeat sequence and 4.10% tandem repeats of the genome were

detected **(Table 1)**. Comparative statistics of genome quality metrics with the assembled goose genome (including Zhedong white goose, Sichuan white goose and Tianfu goose) are shown in **Table 2**.

Phylogenetic analysis

To investigate the genomic evolution of poultry, we compared the sequences of eight bird species (Lion-head goose, Zhedong white goose, duck, turkey, chicken, pigeon, saker, and titmouse) and green lizard, clustering the genes into 15,162 gene families **(Fig. 3A, Supplementary Table S5)**. Among these, 6,422 single-copy gene families were identified and used to construct a phylogenetic tree **(Fig. 3B)**. This revealed that the geese and ducks were clustered into a subclade that probably evolved from a common ancestor approximately 28.42 million years ago (Mya). As expected, the Lion-head goose displayed a close relationship with the Zhedong white goose. The divergence time between the Lion-head goose and Zhedong white goose was estimated to be 13.79 Mya, and that between chicken and turkey was nearly 25.07 Mya. The above results confirmed the reliability of the tree.

Of all the gene families in the Lion-head goose, 4,233 gene families were significantly expanded and 324 were contracted. Compared with Zhedong white goose, the Lion-head goose had more gene families and there are also more events of gene family expansion and contraction. Moreover, we mixed the gene family sets of several *Anatidae* varieties (duck, Zhedong white goose, Lion-head goose), and performed expansion and contraction analysis and corresponding GO enrichment analysis. In this task, the GO analysis of expanded gene families suggested the olfactory perception, such as detection of chemical stimulus involved in sensory perception of smell (GO:0050911, $p = 6.97\times10^{-8}$), and odorant-binding (GO:0005549, $p = 1.47\times10^{-5}$), both of which may be related to the adaptation of the species to find food in water **(Fig. 4A, Supplementary Table S6)**. Meanwhile, contracted gene families were concentrated in the areas of glucose synthesis and metabolism, such as hexokinase activity (GO:0004396, $p = 7.64\times10^{-26}$), glucose binding (GO:0005536, $p = 2.30\times10^{-22}$), cellular glucose homeostasis (GO:0001678, $p = 6.84\times10^{-18}$), glycolytic process (GO:0006096, $p = 1.75\times10^{-15}$), hexose metabolic process (GO:0019318, $p = 2.66\times10^{-14}$), carbohydrate phosphorylation (GO:0046835, $p = 1.68\times10^{-9}$), and glucose 6-phosphate metabolic process (GO:0051156, $p = 1.27\times10^{-9}$), which may be closely related to characteristics of glycogen storage and utilization during migration **(Fig. 4B, Supplementary Table**

S7). Besides, 220 unique gene families (other species lack these gene families) of the Lion-head goose were identified and functionally annotated in GO categories, such as protein kinase activity (GO:0004672, $p = 6.85\times10^{-9}$), the regulation of apoptotic process (GO:0042981, $p = 5.78\times10^{-34}$), the adenylate cyclase-modulating G protein-coupled receptor signaling pathway (GO:0007188, $p = 5.92\times10^{-3}$), and fatty-acyl-CoA reductase (alcohol-forming) activity (GO:0080019, $p = 8.94\times10^{-5}$, **Fig. 4C, Supplementary Table S8**). Interestingly, we annotated a reproduction-related protein in the species-specific gene family, *Sterile* (Pfam ID: PF03015), acting on fatty-acyl-CoA reductase (alcohol-forming) activity, which may be related to the low reproductive rate caused by congenital infertility in geese.

Collinearity analysis allows one to judge molecular evolutionary events between species and explain the structural differences between the two genomes. We identified synteny blocks among avian genomes and found high collinearity between our assembly and the duck genome (genome size =1.19 Gb). Here, multiple chromosomes (Chr 1-5, 10, 12, 15, 17-20, 23, 26, 27, 29, 30, 32, 34, 36, 37, 39) of Lion-head goose were almost one-to-one collinear with those of the duck, but some chromosomal rearrangements occurred **(Fig. 3C, Supplementary Figure S3)**. For example, on some chromosomes like Chr 1, 2, 3, and 4 of the duck genome, genes break and rearrange on the Lion-head goose genome, resulting in sequential inversion. In addition, some scaffolds such as Chr 9, 24, 25, 31, 35, 38 and 40, were not correlated with any chromosome of the duck genome maybe due to the different sources of genes on the chromosome. These results indicate that chromosome inversion and interchromosomal recombination may have occurred specifically in Lion-head goose during the evolutionary process, but this requires further investigation and verification. Moreover, Chr 4 of Lion-head goose was found to correspond to the sex chromosome Z of duck, except for the inversions of small patches of segments; therefore, we inferred that Chr 4 was the sex chromosome of the Lion-head goose. This information will be fundamental for comparative genomic studies in *Anatidae* animals.

Cluster analysis of different goose species population

Blood samples were collected from 514 geese (including Lion-head goose, Wuzong goose, Huangzong goose and Magang goose), and their weight was recorded, with the Lion-head goose using the minimum weight, the Wuzong goose using the maximum weight, and the Huangzong goose and Magang goose

using the average weight. That is, the Lion-head goose weighed at least 9 kg, the Wuzong goose weighed at most 2.5 kg, the Huangzong goose weighed about 3-4 kg, and the Magang goose weighed 4.8-5.5 kg (**Table 6**). Blood from each sample was used for paired-end 100 resequencing. And the average raw data was 1,520.60 Mb, the average sequencing depth was 12.05×, the average coverage was 7.56%, the average matching rate was 91.31%, and 44,858 SNP loci were retained for subsequent analysis after screening SNPs with minimum allele frequency <5%, Hardy-Weinberg equilibrium test significance threshold of 10^{-7}, and maximum deletion rate threshold of 0.7. We reconstructed the goose population structure using SNP data, revealing four distinct subpopulations. The PCA results demonstrated that the Lion-head Goose population was clearly distinguishable from the Magang Goose, Wuzong Goose and Huangzong Goose, and there was a clear differentiation within the species (**Fig. 5A**). The clustering of Magang Goose and Huangzong Goose was closer together, probably related to their closer geographical location and the existence of some genetic exchange. The phylogenetic tree results were consistent with the PCA results. The clustering of Magang Goose and Huangzong Goose was closer to each other, and they clustered into one branch with Wuzong Goose (**Fig. 5B**).

Candidate genomic regions for body weight based on combined analyses of GWAS and selective-sweep

The Lion-head Goose, Huangzong Goose, Magang Goose, and Wuzong Goose are all local species in Guangdong, but they differ greatly in body weight. In this study, we sought to reveal genomic changes associated with body weight in the four goose species and screen genomic regions and genes. Selective sweep analysis was performed based on the F_{ST} index, considering the top 5% window as candidate regions. And 979 selective regions containing 818 genes were detected.

We then combined the GWAS results with the detected selective features to screen for candidate genomic regions responsible for the differences in goose weight. From the Manhattan plot (**Fig. 5C**), a total of 10 significant signals were found to be associated with body weight trait in geese at the genome-wide level, including one significant SNP detected on Chr 2, 8, 9, and 33 respectively ($-\log(p) > 7.30$), and six significant SNPs annotated by two genes on Chr 22, with the closest Manhattan plot SNP peak on Chr 9 for the gene *OR* (Olfactory receptor). Six significant SNPs on Chr 22 are located between

1,992,485 and 1,992,520 bp, a region that spans only a physical distance of 35 bp but contains six SNP loci, making it necessary to analyze these SNPs in this small region in detail to determine whether multiple QTL are involved. The most significant SNP in this region could explain about 8.19% of the phenotypic variation. Apart from significant SNPs, potentially significant QTLs were detected on many chromosomes (including Chr 2, 3, 6, 7, 10, 11, 15, 16, 20, 28, 30, 32, 36), with a total of 25 implied significant SNPs (4.90< -log (*p*) <7.30). On Chr 30, the suggestively significant SNPs were located between 1,258,517 and 2,422,666 bp, spanning approximately 1.16 Mb, with the most significant SNPs in this region explaining approximately 6.12% of the phenotypic variation (**Table 4**). In the present study, we identified genes in the region near the significant SNPs, annotating a total of 21 genes. These genes may be important in mediating growth and development, and we inference that the *LDLRAD4* gene may play a key role in developmental plasticity in geese, while the *GPR180* gene may regulate the locomotor behavior of geese to make them stronger (**Fig. 6**). GWAS peaks overlapped with genomic regions with selective features on some chromosomes (**Supplementary Data**). This suggests that the region carrying QTL are not only associated with body weight in GWAS, but are also under selection during domestication.

Discussion

Despite the importance of the genus *Anser*, an economically important animal, the relative scarcity of genomic resources has largely hindered progress in studying genome evolution and molecular breeding in the major animals. High-quality chromosome-level genomes can provide key resources for studying. This study describes a chromosome-scale assembly of Lion-head goose obtained by a combination of data from the Illumina, SMRT, BioNano, and Hi-C platforms. The genome assembly is 1.19 Gb in length, and more than 97.27% of the assembled genome is anchored on 40 pseudo-chromosomes. The BUSCO assessment revealed 99.02% complete genes in the assembled genome, making it a better-continuity and higher-quality genome assembly than the recently published Tianfu goose genome with a contig N50 of 1.85 Mb and scaffold N50 of 33.12 Mb [39]. Compared with the cultivated breed Tianfu goose, Lion-head goose, a traditional native breed, should occupy a more prominent position in the germplasm

resources, and its evolving message can provide a reference for other local breeds which is worthy of in-depth study.

Comparative genomics is the analysis of the structural characteristics of multiple individual genomes of a species or genomes of multiple species to find out the similarities and differences of gene sequences of species with the help of bioinformatics, and then to study the gene family analysis, analyze the differentiation and evolution of species, to provide a basis for elucidating species evolution. In this study, the evolutionary events of the Lion-head goose were analyzed by comparing the genome sequences with those of other birds. The results showed that the Lion-head goose and Zhedong White goose were most closely related, diverging at about 13.8 Mya, while the geese and ducks diverged at 28.4 Mya. The results were similar to those of Zhedong White goose, Sichuan White goose and Tianfu goose, indicating the accuracy of the assembly result of this study. Comparative genomic analysis revealed the genetic basis of interesting characters, which helped elucidate important biological implications and obtain solutions for genomic evolution between Lion-head geese and other species of *Anatidae* family, facilitating future genetic breeding programs. This is the first chromosomal level reference genome of Lion-head goose, providing important genomic data for the study of the family *Anatidae*.

The genomic information of the species population was obtained by whole-genome resequencing, and a large amount of variation information was obtained by comparison with the reference genome. Based on the correlation between differences in variation information and phenotypic differences of individuals, the adaptation of species to the environment, scanning of variant loci associated with important traits at the genome level, and localization of genetic mutations were discussed. Lion head goose, Magang goose, Huangzong goose and Wuzong goose are the main breeds of geese in Guangdong Province. Although they all belong to Guangdong Province, the body weight of adult geese varies greatly, and the molecular mechanism causing the huge difference is still unclear. In this study, four goose species were resequenced and examined for variation. Principal component analysis and phylogenetic tree analysis revealed significant differences among several goose species, indicating the feasibility of this study. Subsequently, GWAS was used to identify the candidate functional SNPs that might cause the weight difference of the four goose species, and the genes such as LDLRAD4, GPR180, and OR

were analyzed and annotated, attributed to play an important role in mediating growth and development. Recently, there have been several studies related to agricultural traits that have achieved success in animal GWAS projects, for example, GWAS for improving reproductive performance and egg quality in geese and *TMEM161A* gene for embryo development [40]. Genome-wide association analysis of the early-lactation milk fat content in 3,513 Fleckvieh bulls and 2327 Holstein bulls detected 6 associated QTL regions, two of which were located near the gene DGAT1 [41]. GWAS was conducted on 225 ducks with different-sized black spots, and the results showed that EDNRB2 was the gene responsible for the variation in duck body surface spot size [42]. In this study, *LDLRAD4* (low-density lipoprotein receptor class A domain containing 4), *OR* (Olfactory receptor), and *GPR180* (G protein-coupled receptor 180) were mainly found to function in body weight traits. Knockdown of *LDLRAD4* enhances transforming growth factor (TGF)-β-induced cell migration, which in turn regulates cell growth, differentiation, motility, apoptosis and matrix protein production [43]. The olfactory receptor (*OR2AT4*) has been shown to stimulate the proliferation of keratin-forming cells in peripheral human tissues [44]. *GPR180*, a component of the TGF-β signaling pathway, also has metabolic relevance in the body and may play an essential role in regulating adipose tissue and systemic energy metabolism [45]. Here we found some correlation between these genes and the TGF-β signaling, presumably this pathway also acts on body weight. Identifying of molecular genetic markers and the main effect QTL associated with critical agricultural traits is of great interest to breeders. Nevertheless, the candidate genes identified in this study were only detected by sequencing data and not experimentally validated. The functions of these candidate SNPs and gene markers need to be further verified by experimental results or other techniques. Thus, the findings in our GWAS study represent a valuable resource for geese and provide a new opportunity and basis for geneticists and breeders to work together to explore the genetics behind various agricultural traits.

Conclusions

In summary, we have obtained a high-quality chromosome-scale draft assembly of a purebred Lion-head goose, which provides a genetic basis for understanding the acquisition of related traits and

facilitates advances in goose genomics and genetic improvement. Moreover, the candidate genes and their variants identified in this study will help clarify our understanding of goose selective breeding and the development of new breeds. The obtained genome sequence of Lion-head goose is a vital addition to the genome of genus *Anser* and is valuable for further understanding goose molecular breeding strategies. This genomic resource is also of high value for evolutionary studies of closely related species.

Data Availability

The final genome assembly data supporting the results of this article is available in the NCBI BioProject repository, [Accession number: PRJNA736831]. The RNA assembly data is available in the NCBI BioProject repository, [Accession number: PRJNA807796]. The raw re-sequencing genome data supporting of the GWAS study is available in the NCBI BioProject repository [Accession number: PRJNA552198, PRJNA552383, and PRJNA552384]. All supporting data are available in the *GigaScience* GigaDB database [46].

Additional Files

Supplementary Figure S1. Sequencing process and presentation.

Supplementary Figure S2. BUSCO assessment of the assembly genome of Lion-head goose.

Supplementary Figure S3. Gene synteny between the Lion-head goose and duck genomes.

Supplementary Table S1. Statistics of sequenced clean data.

Supplementary Table S2. Statistics of genome survey.

Supplementary Table S3. Statistics of genome assembly quality.

Supplementary Table S4. Summary of BUSCOs genome evaluation.

Supplementary Table S5: Summary of gene families from several species.

Supplementary Table S6. GO annotation of expanded gene families from Anatidae varieties (Duck, Zhedong white goose, Lion-head goose; Top 20).

Supplementary Table S7. GO annotation of contraction gene families from Anatidae varieties (Duck, Zhedong white goose, Lion-head goose; Top 20).

Supplementary Table S8. GO annotation of unique gene families from the Lion-head goose.

Supplementary Data. Significant information of selective-sweep analysis.

Abbreviations

BLAST: Basic Local Alignment Search Tool; BWA: Burrows-Wheeler Aligner; BUSCO: Benchmarking Universal Single-Copy Orthologs; Chr: chromosome; GATK4: Genome Analysis Toolkit 4; Gb: gigabase pairs; GO: gene ontology; GPR180: G protein-coupled receptor 180; GWAS: genome-wide association study; HERA: Highly Efficient Repeat Assembly; Hi-C: high-throughput chromosome conformation capture; Kb: kilobase pairs; kg: kilogram; LDLRAD4: low-density lipoprotein receptor class A domain containing 4; LTR: long terminal repeat; Mb: megabase pairs; Mya: million years ago; NCBI: National Center for Biotechnology Information; OR: Olfactory receptor; OR2AT4: olfactory receptor family 2 subfamily AT member 4; PacBio: Pacific Biosciences; PCA: Principal component analysis; QTL: quantitative trait locus; RAxML: Randomized Axelerated Maximum Likelihood; RNA-seq: RNA sequencing; SMRT: single molecule real-time; SNP: single-nucleotide polymorphism; STAR: Spliced Transcripts Alignment to a Reference; TE: transposable element; TGF: transforming growth factor; TMEM161A: Transmembrane protein 161A.

Competing Interests

The authors declare that they have no conflict of interest.

Funding

This work was supported by the Key Research and Development Program of Guangdong Province (2020B020222001), the Construction of Modern Agricultural Science and Technology Innovation Alliance in Guangdong Province (2021KJ128, 2020KJ128), the National Modern Agricultural Industry Science and Technology Innovation Center in Guangzhou (2018kczx01), the Guangdong Provincial Promotion Project on Preservation and Utilization of Local Breed of Livestock and Poultry (4000-F18260), the Guangdong Basic and Applied Basic Research Foundation (2019A1515012006), the Science and Technology Program of Guangdong province, China (2020B1212060060), the China Agriculture Research System of MOF and MARA (CARS-42-13), the Special Project of National Modern Agricultural Industrial Technology System (CARS-41) and Provincial Science and Technology

Special Fund Project for Zhongshan City (major special project + Task list management mode) (2021sdr003). The authors would like to thank the BGI in Shenzhen for their work on genome sequencing. We also thank the staff of Minglead Gene for providing the technical and computing support during the research.

Author's Contributions

Q.X., Z.L., and X.Z. conceived and designed the research. X.Z., J.C., and Q.Z. coordinated the project. J.C. and Z.L. provided animal samples. Q.Z. and Z. X. collected and prepared the samples. Q.Z. performed sequencing, assembly and bioinformatics analysis. W.L., and F.C. led work identifying genes, and H.L., W.C. aided with many aspects of gene identification and did the GO analyses. Q.Z., X.Z. wrote and revised the manuscript and the supplementary information. J.W., M.J., Z.H., H.Z., Z.L., and Q.X. participated in discussions and provided valuable advice. All authors read and approved the manuscript.

References

1. Hoyo JD, Elliott A, Sargatal J, et al. Handbook of the birds of the world. Barcelona: Lynx Edicions; 1992.
2. Madsen J, Marcussen LK, Knudsen N, et al. Does intensive goose grazing affect breeding waders? Ecol Evol 2019;**9**(24):14512-14522. doi:10.1002/ece3.5923.
3. Wang Y, Li SM, Huang J, et al. Mutations of TYR and MITF Genes are Associated with Plumage Colour Phenotypes in Geese. Asian-Australas J Anim Sci 2014;**27**(6):778-83. doi:10.5713/ajas.2013.13350.
4. Gao G, Zhao X, Li Q, et al. Genome and metagenome analyses reveal adaptive evolution of the host and interaction with the gut microbiota in the goose. Sci Rep 2016;**6**:32961. doi:10.1038/srep32961.
5. Yao Y, Yang YZ, Gu TT, et al. Comparison of the broody behavior characteristics of different breeds of geese. Poult Sci 2019;**98**(11):5226-5233. doi:10.3382/ps/pez366.
6. Lu L, Chen Y, Wang Z, et al. The goose genome sequence leads to insights into the evolution of waterfowl and susceptibility to fatty liver. Genome Biol 2015;**16**:89. doi:10.1186/s13059-015-0652-y.
7. Li HF, Zhu WQ, Chen KW, et al. Two maternal origins of Chinese domestic goose. Poult Sci 2011;**90**(12):2705-10. doi:10.3382/ps.2011-01425.
8. Tang J, Shen X, Ouyang H, et al. Transcriptome analysis of pituitary gland revealed candidate genes and gene networks regulating the growth and development in goose. Anim Biotechnol 2020:1-11. doi:10.1080/10495398.2020.1801457.
9. Zhang X, Wang J, Li X, et al. Transcriptomic investigation of embryonic pectoral muscle reveals increased myogenic processes in Shitou geese compared to Wuzong geese. Br Poult Sci 2021;**62**(5):650-657. doi:10.1080/00071668.2021.1912292.
10. Ardui S, Ameur A, Vermeesch JR, et al. Single molecule real-time (SMRT) sequencing comes of age: applications and utilities for medical diagnostics. Nucleic Acids Res 2018;**46**(5):2159-2168. doi:10.1093/nar/gky066.
11. Yoshinaga Y, Daum C, He G, et al. Genome Sequencing. Methods Mol Biol 2018;**1775**:37-52. doi:10.1007/978-1-4939-7804-5_4.
12. Kong S, Zhang Y. Deciphering Hi-C: from 3D genome to function. Cell Biol Toxicol 2019;**35**(1):15-32. doi:10.1007/s10565-018-09456-2.
13. Nakano K, Shiroma A, Shimoji M, et al. Advantages of genome sequencing by long-read sequencer using SMRT technology in medical area. Hum Cell 2017;**30**(3):149-161. doi:10.1007/s13577-017-0168-8.
14. Jain M, Olsen HE, Turner DJ, et al. Linear assembly of a human centromere on the Y chromosome. Nat Biotechnol 2018;**36**(4):321-323. doi:10.1038/nbt.4109.
15. Sun L, Gao T, Wang F, et al. Chromosome-level genome assembly of a cyprinid fish Onychostoma macrolepis by integration of nanopore sequencing, Bionano and Hi-C technology. Mol Ecol Resour 2020;**20**(5):1361-1371. doi:10.1111/1755-0998.13190.
16. Bocklandt S, Hastie A, Cao H. Bionano Genome Mapping: High-Throughput, Ultra-Long Molecule Genome Analysis System for Precision Genome Assembly and Haploid-Resolved Structural Variation Discovery. Adv Exp Med Biol 2019;**1129**:97-118. doi:10.1007/978-981-13-6037-4_7.

17. Koren S, Walenz BP, Berlin K, et al. Canu: scalable and accurate long-read assembly via adaptive k-mer weighting and repeat separation. Genome Res 2017;**27**(5):722-736. doi:10.1101/gr.215087.116.
18. Du H, Liang C. Assembly of chromosome-scale contigs by efficiently resolving repetitive sequences with long reads. Nat Commun 2019;**10**(1):5360. doi:10.1038/s41467-019-13355-3.
19. Li H, Durbin R. Fast and accurate short read alignment with Burrows-Wheeler transform. Bioinformatics 2009;**25**(14):1754-60. doi:10.1093/bioinformatics/btp324.
20. Danecek P, Bonfield JK, Liddle J. et al. Twelve years of SAMtools and BCFtools. Gigascience. 2021;10(2):giab008. doi: 10.1093/gigascience/giab008.
21. Walker BJ, Abeel T, Shea T, et al. Pilon: an integrated tool for comprehensive microbial variant detection and genome assembly improvement. Plos One 2014;**9**(11):e112963. doi:10.1371/journal.pone.0112963.
22. Durand NC, Shamim MS, Machol I, et al. Juicer Provides a One-Click System for Analyzing Loop-Resolution Hi-C Experiments. Cell Syst 2016;**3**(1):95-8. doi:10.1016/j.cels.2016.07.002.
23. Dudchenko O, Batra SS, Omer AD, et al. De novo assembly of the Aedes aegypti genome using Hi-C yields chromosome-length scaffolds. Science 2017;**356**(6333):92-95. doi:10.1126/science.aal3327.
24. Servant N, Varoquaux N, Lajoie BR, et al. HiC-Pro: an optimized and flexible pipeline for Hi-C data processing. Genome Biol 2015;**16**(1). doi:10.1186/s13059-015-0831-x.
25. Bolger AM, Lohse M, Usadel B. Trimmomatic: a flexible trimmer for Illumina sequence data. Bioinformatics 2014;**30**(15):2114-20. doi:10.1093/bioinformatics/btu170.
26. Grabherr MG, Haas BJ, Yassour M, et al. Full-length transcriptome assembly from RNA-Seq data without a reference genome. Nat Biotechnol 2011;**29**(7):644-52. doi:10.1038/nbt.1883.
27. Huang Y, Niu B, Gao Y, et al. CD-HIT Suite: a web server for clustering and comparing biological sequences. Bioinformatics 2010;**26**(5):680-2. doi:10.1093/bioinformatics/btq003.
28. Dobin A, Davis CA, Schlesinger F, et al. STAR: ultrafast universal RNA-seq aligner. Bioinformatics 2013;**29**(1):15-21. doi:10.1093/bioinformatics/bts635.
29. Seppey M, Manni M, Zdobnov EM. BUSCO: Assessing Genome Assembly and Annotation Completeness. Methods Mol Biol 2019;**1962**:227-245. doi:10.1007/978-1-4939-9173-0_14.
30. Manni M, Berkeley MR, Seppey M, et al. BUSCO: Assessing Genomic Data Quality and Beyond. Curr Protoc 2021;**1**(12):e323. doi:10.1002/cpz1.323.
31. Lu L, Chen Y, Wang Z, et al. The goose genome sequence leads to insights into the evolution of waterfowl and susceptibility to fatty liver. Genome Biol 2015;**16**:89. doi:10.1186/s13059-015-0652-y.
32. Benson G. Tandem repeats finder: a program to analyze DNA sequences. Nucleic Acids Res 1999;**27**(2):573-80. doi:10.1093/nar/27.2.573.
33. Wang Y, Tang H, Debarry JD, et al. MCScanX: a toolkit for detection and evolutionary analysis of gene synteny and collinearity. Nucleic Acids Res 2012;**40**(7):e49. doi:10.1093/nar/gkr1293.
34. Stamatakis A. RAxML version 8: a tool for phylogenetic analysis and post-analysis of large phylogenies. Bioinformatics 2014;**30**(9):1312-3. doi:10.1093/bioinformatics/btu033.
35. Sanderson MJ. r8s: inferring absolute rates of molecular evolution and divergence times in the absence of a molecular clock. Bioinformatics 2003;**19**(2):301-2. doi:10.1093/bioinformatics/19.2.301.
36. Han MV, Thomas GW, Lugo-Martinez J, et al. Estimating gene gain and loss rates in the presence of error in genome assembly and annotation using CAFE 3. Mol Biol Evol 2013;**30**(8):1987-97. doi:10.1093/molbev/mst100.
37. Yu G, Wang LG, Han Y, et al. clusterProfiler: an R package for comparing biological themes among gene clusters. Omics 2012;**16**(5):284-7. doi:10.1089/omi.2011.0118.
38. Purcell S, Neale B, Todd-Brown K, et al. PLINK: a tool set for whole-genome association and population-based linkage analyses. Am J Hum Genet 2007;**81**(3):559-75. doi:10.1086/519795.
39. Li Y, Gao G, Lin Y, et al. Pacific Biosciences assembly with Hi-C mapping generates an improved, chromosome-level goose genome. Gigascience 2020;**9**(10). doi:10.1093/gigascience/giaa114.
40. Gao G, Gao D, Zhao X, et al. Genome-Wide Association Study-Based Identification of SNPs and Haplotypes Associated With Goose Reproductive Performance and Egg Quality. Front Genet 2021;**12**:602583. doi:10.3389/fgene.2021.602583.
41. Daetwyler HD, Capitan A, Pausch H, et al. Whole-genome sequencing of 234 bulls facilitates mapping of monogenic and complex traits in cattle. Nat Genet 2014;**46**(8):858-65. doi:10.1038/ng.3034.
42. Xi Y, Xu Q, Huang Q, et al. Genome-wide association analysis reveals that EDNRB2 causes a dose-dependent loss of pigmentation in ducks. Bmc Genomics 2021;**22**(1):381. doi:10.1186/s12864-021-07719-7.
43. Nakano N, Maeyama K, Sakata N, et al. C18 ORF1, a novel negative regulator of transforming growth factor-beta signaling. J Biol Chem 2014;**289**(18):12680-92. doi:10.1074/jbc.M114.558981.
44. Cheret J, Bertolini M, Ponce L, et al. Olfactory receptor OR2AT4 regulates human hair growth. Nat Commun 2018;**9**(1):3624. doi:10.1038/s41467-018-05973-0.

45. Balazova L, Balaz M, Horvath C, et al. GPR180 is a component of TGFbeta signalling that promotes thermogenic adipocyte function and mediates the metabolic effects of the adipocyte-secreted factor CTHRC1. Nat Commun 2021;**12**(1):7144. doi:10.1038/s41467-021-27442-x.
46. Zhao Q, Chen J, Xie Z, et al. Supporting data for "Chromosome-level genome assembly of goose provides insight into the adaptation and growth of local goose breeds" GigaScience Database. 2023. http://dx.doi.org/10.5524/102339.

Figure legends

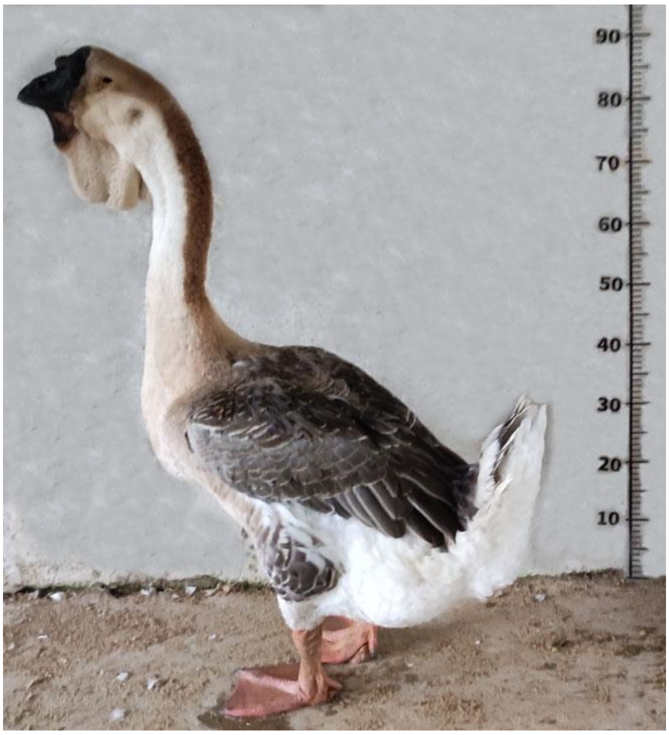

Figure 1. A picture of a male adult Lion-head goose.

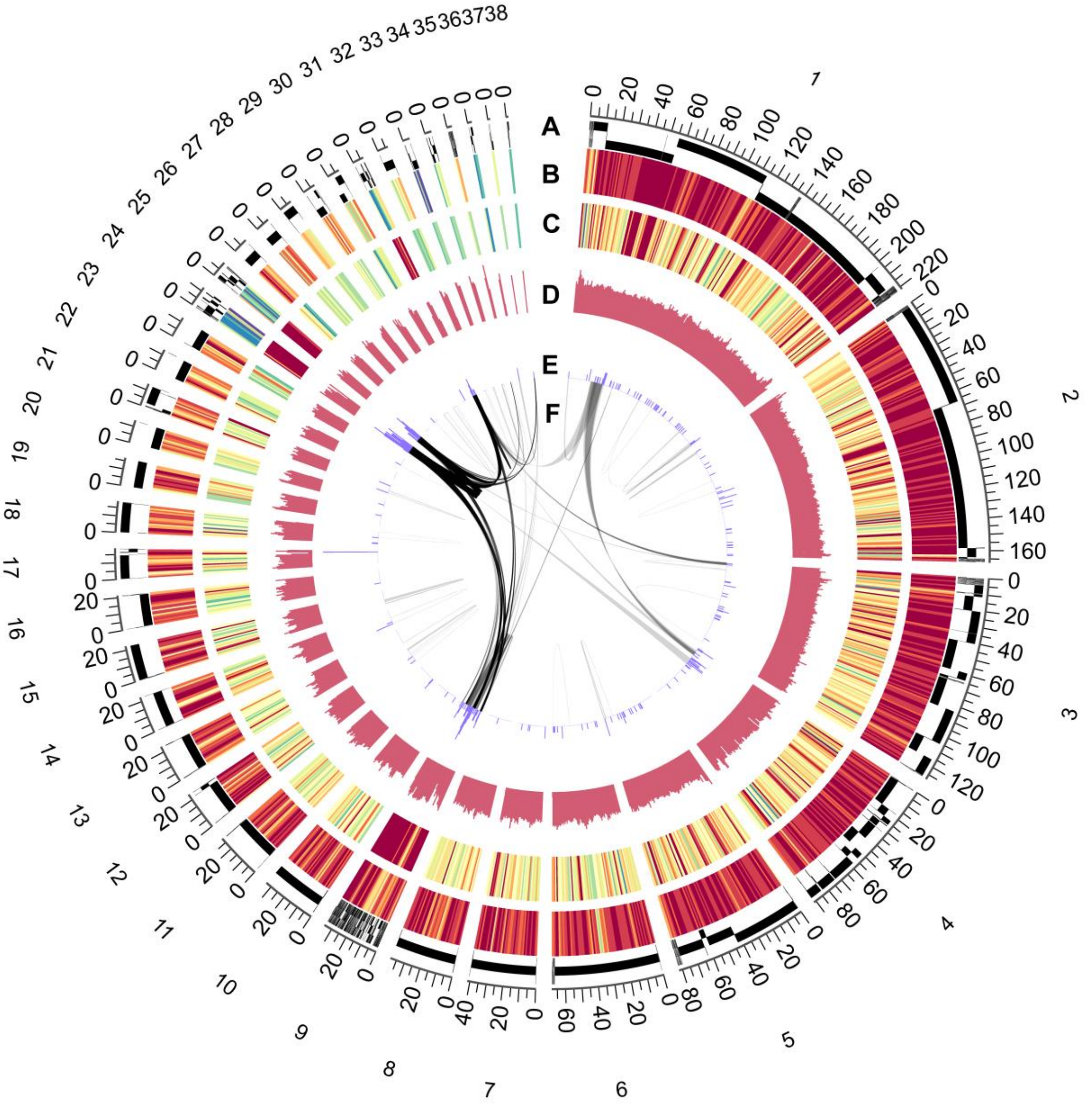

Figure 2. Distribution of genomic features. Concentric circle diagram presents the distribution of genomic features of Lion-head goose using nonoverlapping sliding windows with sizes of 1 Mb (from outmost to innermost). **(A)** the assembled pseudo-chromosome and the corresponding position; **(B)** gene density calculated on the basis of the number of genes; **(C)** average expression level of overall 36 samples. eight tissues (i.e., brain, pharyngeal pouch, head sarcoma, spleen, liver, chest muscle, kidney and heart) and blood collected from four healthy adult animals; **(D)** GC content; **(E)** density of TE; **(F)** gene synteny and collinearity analysis.

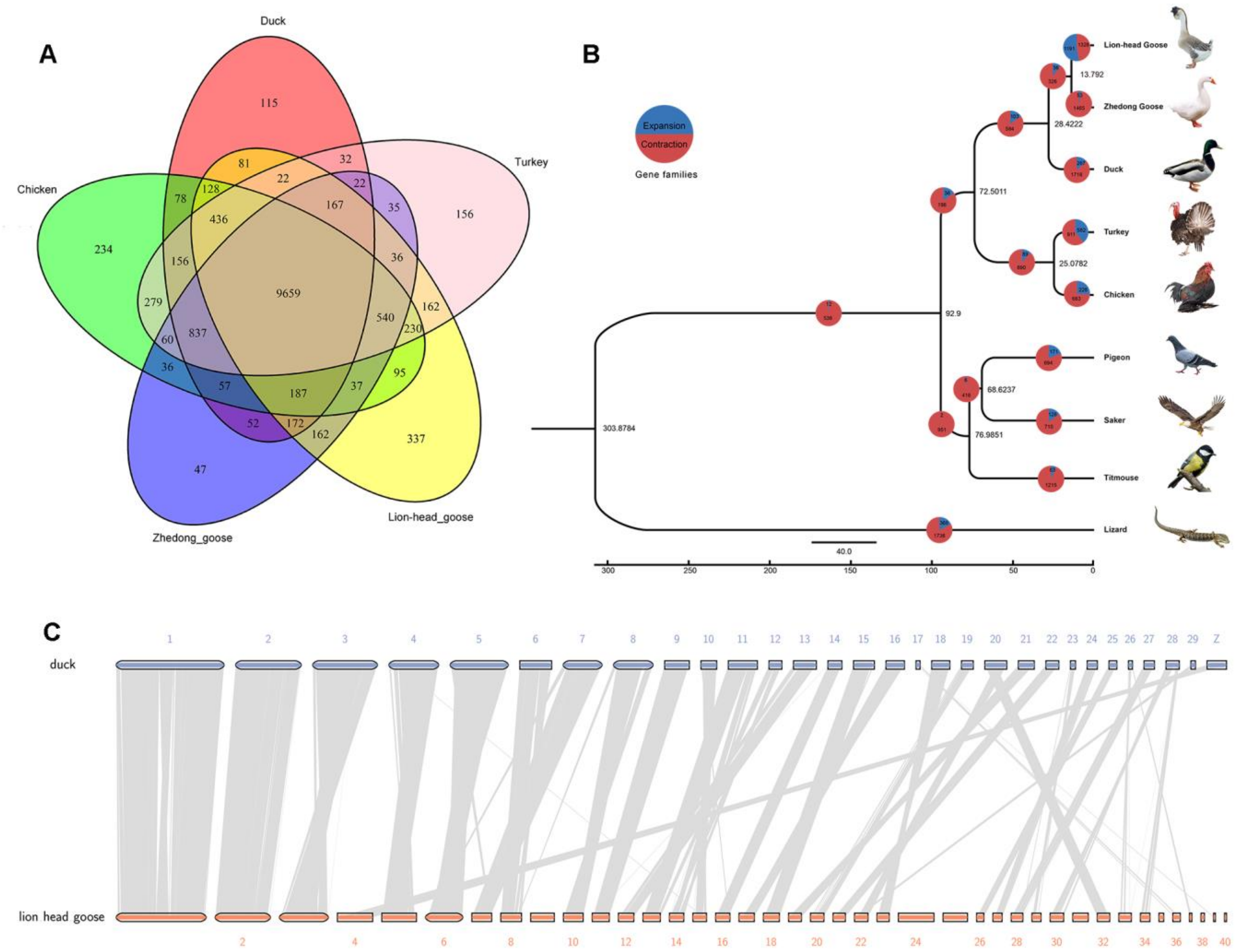

Figure 3. Phylogenetic relationship and comparative genomics analyses. (A) Venn diagram showing the orthologous gene families shared among the genomes of Lion-head goose, Zhedong white goose, chicken, duck, and turkey. **(B)** Phylogenetic tree with the divergence times and history of orthologous gene families. Numbers on the nodes represent divergence times. The numbers of gene families that expanded (green) or contracted (red) in each lineage after speciation are shown on the circles of the corresponding branch. **(C)** Gene comparison of homologous chromosomes between Lion-head goose and duck. Gray lines indicate collinearity between the genomes.

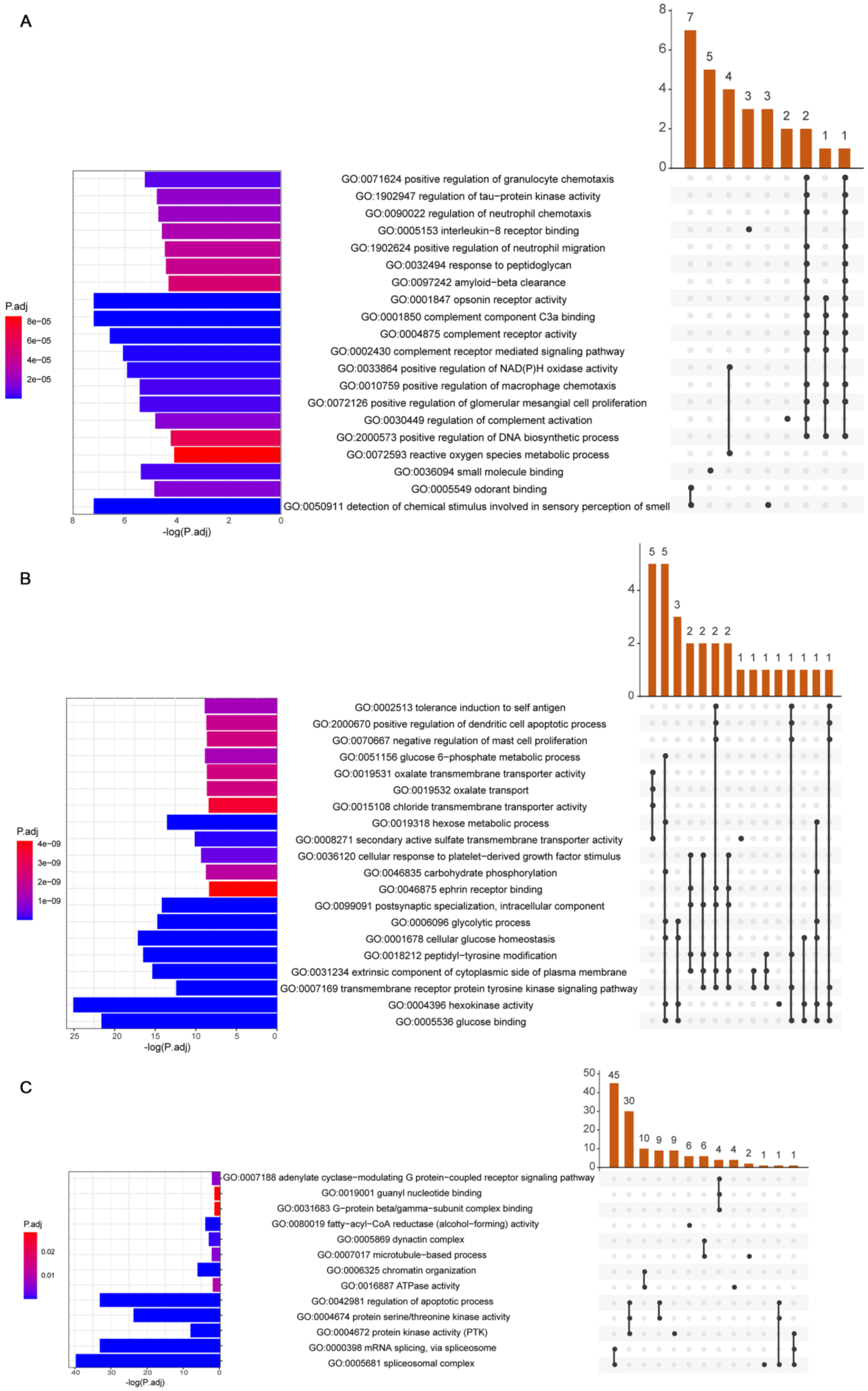
A
P.adj
-log(P.adj)
GO:0071624 positive regulation of granulocyte chemotaxis
GO:1902947 regulation of tau−protein kinase activity
GO:0090022 regulation of neutrophil chemotaxis
GO:0005153 interleukin−8 receptor binding
GO:1902624 positive regulation of neutrophil migration
GO:0032494 response to peptidoglycan
GO:0097242 amyloid−beta clearance
GO:0001847 opsonin receptor activity
GO:0001850 complement component C3a binding
GO:0004875 complement receptor activity
GO:0002430 complement receptor mediated signaling pathway
GO:0033864 positive regulation of NAD(P)H oxidase activity
GO:0010759 positive regulation of macrophage chemotaxis
GO:0072126 positive regulation of glomerular mesangial cell proliferation
GO:0030449 regulation of complement activation
GO:2000573 positive regulation of DNA biosynthetic process
GO:0072593 reactive oxygen species metabolic process
GO:0036094 small molecule binding
GO:0005549 odorant binding
GO:0050911 detection of chemical stimulus involved in sensory perception of smell
B
P.adj
-log(P.adj)
GO:0002513 tolerance induction to self antigen
GO:2000670 positive regulation of dendritic cell apoptotic process
GO:0070667 negative regulation of mast cell proliferation
GO:0051156 glucose 6−phosphate metabolic process
GO:0019531 oxalate transmembrane transporter activity
GO:0019532 oxalate transport
GO:0015108 chloride transmembrane transporter activity
GO:0019318 hexose metabolic process
GO:0008271 secondary active sulfate transmembrane transporter activity
GO:0036120 cellular response to platelet−derived growth factor stimulus
GO:0046835 carbohydrate phosphorylation
GO:0046875 ephrin receptor binding
GO:0099091 postsynaptic specialization, intracellular component
GO:0006096 glycolytic process
GO:0001678 cellular glucose homeostasis
GO:0018212 peptidyl−tyrosine modification
GO:0031234 extrinsic component of cytoplasmic side of plasma membrane
GO:0007169 transmembrane receptor protein tyrosine kinase signaling pathway
GO:0004396 hexokinase activity
GO:0005536 glucose binding
C
P.adj
-log(P.adj)
GO:0007188 adenylate cyclase−modulating G protein−coupled receptor signaling pathway
GO:0019001 guanyl nucleotide binding
GO:0031683 G−protein beta/gamma−subunit complex binding
GO:0080019 fatty−acyl−CoA reductase (alcohol−forming) activity
GO:0005869 dynactin complex
GO:0007017 microtubule−based process
GO:0006325 chromatin organization
GO:0016887 ATPase activity
GO:0042981 regulation of apoptotic process
GO:0004674 protein serine/threonine kinase activity
GO:0004672 protein kinase activity (PTK)
GO:0000398 mRNA splicing, via spliceosome
GO:0005681 spliceosomal complex

Figure 4. GO enrichment analysis of gene families. (A) Expanded and **(B)** contracted gene families from Anatidae varieties (duck, Zhedong white goose, Lion-head goose). **(C)** Unique gene families from the Lion-head goose. The bar graph on the left represents the P-adjust gradient of GO terms, and the color corresponds to the number on the x-axis (i.e. -log (P.adj)). The bluer the color is, the smaller the P-adjust is, and the more significant it is. The redder the color is, the larger the P-adjust is, and the less significant it is. The upper right bar chart exhibits that several genes act together on the terms below. The lower right chart displays the intersection of the genes of each term; the dots connected by lines represent the intersection of multiple terms; the black dots represent “yes”, and the gray dots represent “no”.

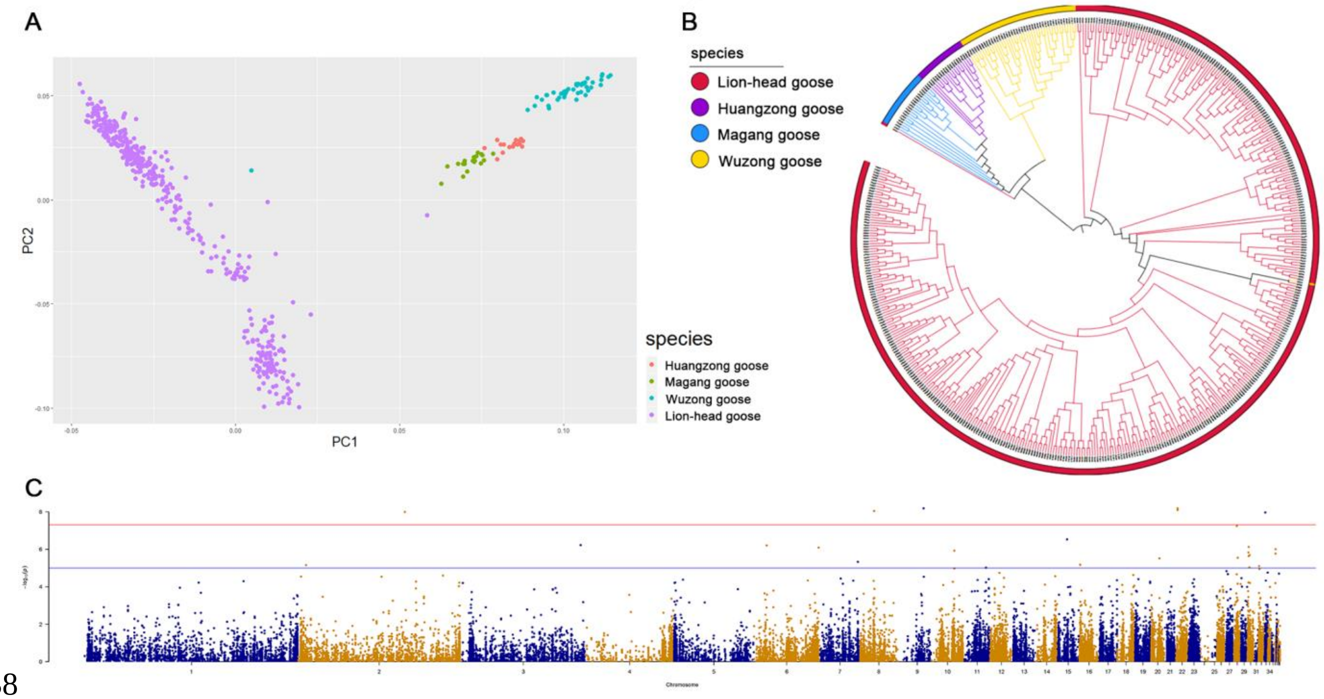

Figure 5. Comparison of different goose species and genome-wide association analysis of body weight. (A) Principal component analysis of sample structures using first two principal components. **(B)** The phylogenetic trees of several goose species. **(C)** Manhattan plot of genome-wide association analysis for body weight. The X-axis indicates chromosomes, and Y-axis indicates the P values of the SNP markers. The red solid line indicates the threshold P value for genome-wide significance. The blue solid line indicates the threshold P value for the significance of potential association.

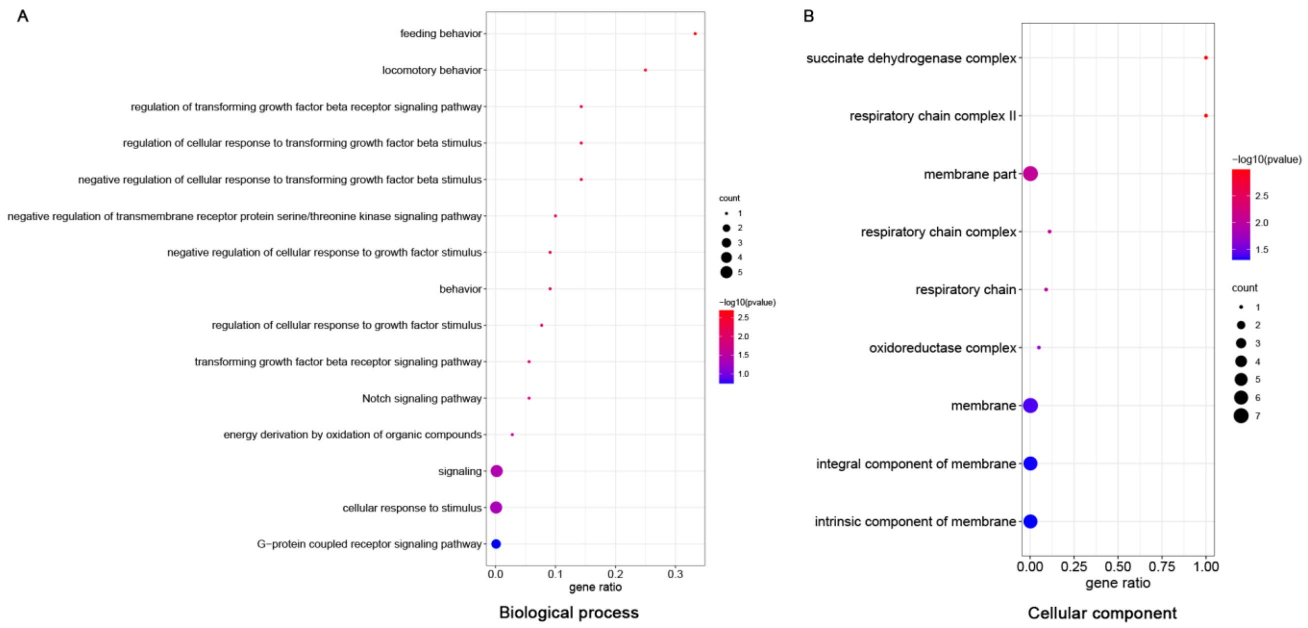

Figure 6. GO analysis of body weight-related genes:(A) Biological processes level, (B) Cellular component level.

Table 1: Summary of repeat classification.

Type	Length	Percent
Long interspersed nuclear element	76,437,757	5.98
Simple sequence repeats	23,026,311	1.80
Low complexity	4,663,288	0.36
Tandem repeats	52,426,380	4.10
Total	156,553,736	12.25

Table 2: Comparison of the present study with previous quality metrics of goose genome assembly.

Genomic features	Lion-head goose	Zhedong white goose	Sichuan white goose	Tianfu goose
Estimate of genome size (bp)	1,278,045,811	1,208,661,181	1,198,802,839	1,277,099,016
Total length of contigs (bp)	1,268,074,106	1,086,838,604	1,100,859,441	1,113,842,245
Total length of scaffolds (bp)	1,277,289,474	1,122,178,121	1,130,663,797	1,113,913,845
Number of contigs	1,318	60,979	53,336	2,771
Number of scaffolds	1,266	1,050	1,837	2,055
Contig N50 (bp)	21,589,146	27,602	35,032	1,849,874
Scaffold N50 (bp)	27,064,542	5,202,740	5,103,766	33,116,532
Longest contig (bp)	91,420,268	201,281	399,111	10,766,871
Longest scaffold (bp)	98,160,899	24,051,356	20,207,557	70,896,740
GC content	42.39%	38.00%	41.68%	42.15%
No. of predicted protein-coding genes	21,010	16,150	16,288	17,568
Percentage of repeat sequences	12.25%	6.33%	6.90%	8.67%

Table 3: Descriptive statistical of body weight traits.

Species	Number	Max (Kg)	Min (Kg)	Mean±SEM
Lion-head goose	416	15.70	9.00	13.55±1.97
Magang goose	20	5.50	4.80	5.32±0.36
Huangzong goose	20	4.30	2.70	3.40±0.83
Wuzong goose	44	2.50	1.80	2.24±0.25

Table 4: Genome-wide association analysis of body weight in geese.

Chr	Allele	Physical position	Regression coefficient	P value	Genes
2	A	108496954	-0.1886	1.01E-08	LDLRAD4
2	G	7706165	0.2612	6.98E-06	LDLRAD4
3	T	123032780	-0.3979	6.03E-07	EGF, KBTBD
6	A	13264157	-0.24	6.28E-07	TSPAN
6	T	66027192	0.2127	8.14E-07	IGFN1
7	T	39117443	-0.3131	4.66E-06	—
8	T	14712470	0.1865	8.97E-09	PPEF1
9	T	26883582	-2.7E+12	0	OR
10	C	23997415	-0.3032	1.19E-06	—
10	C	23997399	-0.2542	1.05E-05	—

10	T	23997401	-0.2542	1.05E-05	—
11	A	22838749	0.1548	9.55E-06	—
15	T	10257386	0.2527	2.96E-07	GPR180, GPCPD1
16	A	1477673	-0.1892	6.53E-06	—
16	G	1477679	-0.1891	6.78E-06	—
20	A	8531879	0.151	3.05E-06	—
22	A	1992485	-0.3972	6.51E-09	GALNT, AUTS2
22	A	1992518	-0.3973	7.69E-09	GALNT, AUTS2
22	G	1992501	-0.3974	7.94E-09	GALNT, AUTS2
22	C	1992505	-0.3974	7.94E-09	GALNT, AUTS2
22	C	1992507	-0.3974	7.94E-09	GALNT, AUTS2
22	G	1992515	-0.3974	7.94E-09	GALNT, AUTS2
28	C	3587271	0.2936	5.81E-08	PPP1R15B, FGD2
28	G	4472051	-0.2359	2.82E-06	PPP1R15B, FGD2
30	C	1652158	-0.3469	7.53E-07	SH2
30	T	1258517	0.2205	1.48E-06	SH2
30	G	2422665	0.1894	2.04E-06	SH2
30	T	2422666	0.1894	2.04E-06	SH2
30	A	1652207	-0.3289	2.3E-06	SH2
30	T	2269897	0.211	9.22E-06	SH2
32	G	655318	0.2599	7.95E-06	—
33	A	975487	0.2567	1.07E-08	SDHA
36	A	1523127	-0.3274	9.86E-07	SPRY
36	G	1523132	-0.3216	1.7E-06	SPRY
36	C	1523105	-0.3291	1.72E-06	SPRY

Manuscript

Click here to access/download
Supplementary Material
Supplemental_Information 2022.docx
