# Supplementary material for: Chromosome-level genome assembly of goose provides insight into the adaptation and growth of local goose breeds
Source: Gigascience. 2023 Feb 3;12:giad003. doi: 10.1093/gigascience/giad003 (PMC9896136; doi:10.1093/gigascience/giad003)
Supplement: giad003_Supplemental_File [file giad003_supplemental_file.docx]

**Chromosome-Level Genome Assembly of Goose Provides Insight into the Adaptation and Growth of Local Goose Breeds**

**Table of Contents:**

| Supplementary Figure S1 | Page 1 |
| --- | --- |
| Supplementary Figure S2 | Page 2 |
| Supplementary Figure S3 | Page 3 |
| Supplementary Table S1 | Page 4 |
| Supplementary Table S2 | Page 5 |
| Supplementary Table S3 | Page 6 |
| Supplementary Table S4 | Page 7 |
| Supplementary Table S5 | Page 8 |
| Supplementary Table S6 | Page 9 |
| Supplementary Table S7 | Page 10 |
| Supplementary Table S8 | Page 11 |

**
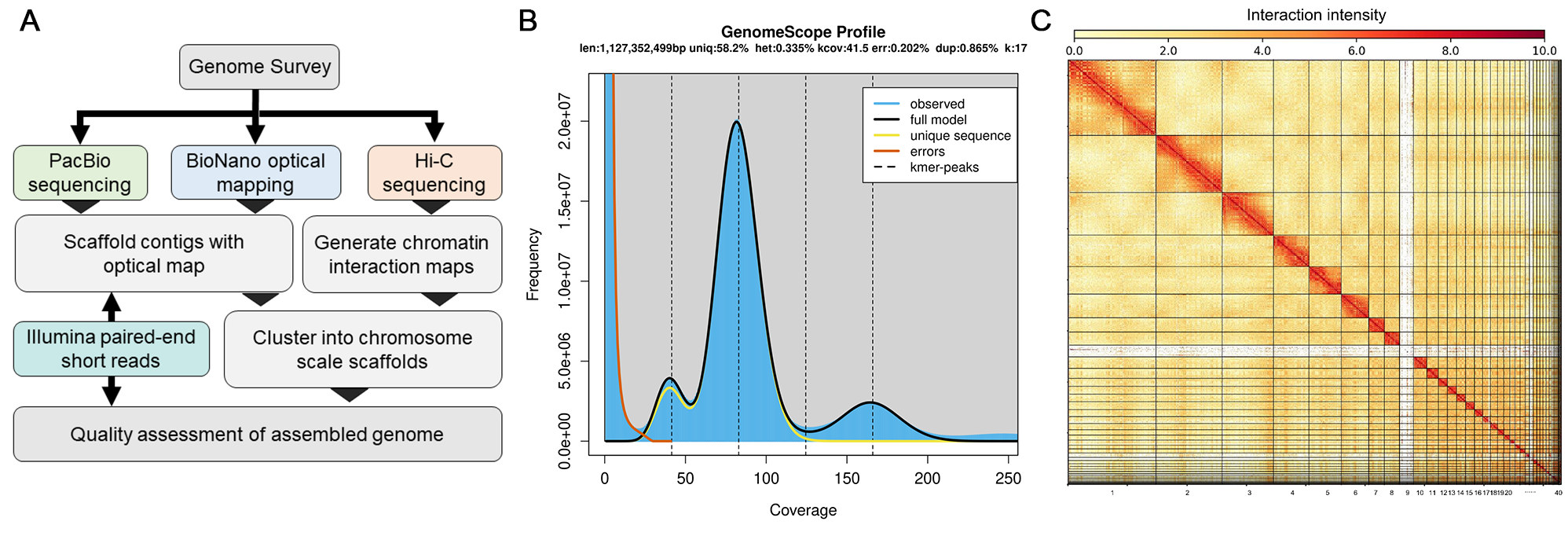
**

**Figure S1. Sequencing process and presentation. (A)**The pipeline for generating chromosome scale scaffolds. Four sets of sequencing data (PacBio, BioNano optical mapping, Hi-C, and Illumina paired-end reads) were produced to generate the Lion-head goose reference genome. A tiered assembled technique using optical mapping data, followed by Hi-C assembly, was used to produce a high-quality assembled genome. **(B)** K-mer (17-mer) analysis for estimating the genome size of Lion-head goose. **(C)** Heatmap of Hi-C chromosomal interaction density. Hi-C interactions among 40 pseudo-chromosomes ordered by length. Dark red indicates strong interactions and yellow indicates weak interactions.


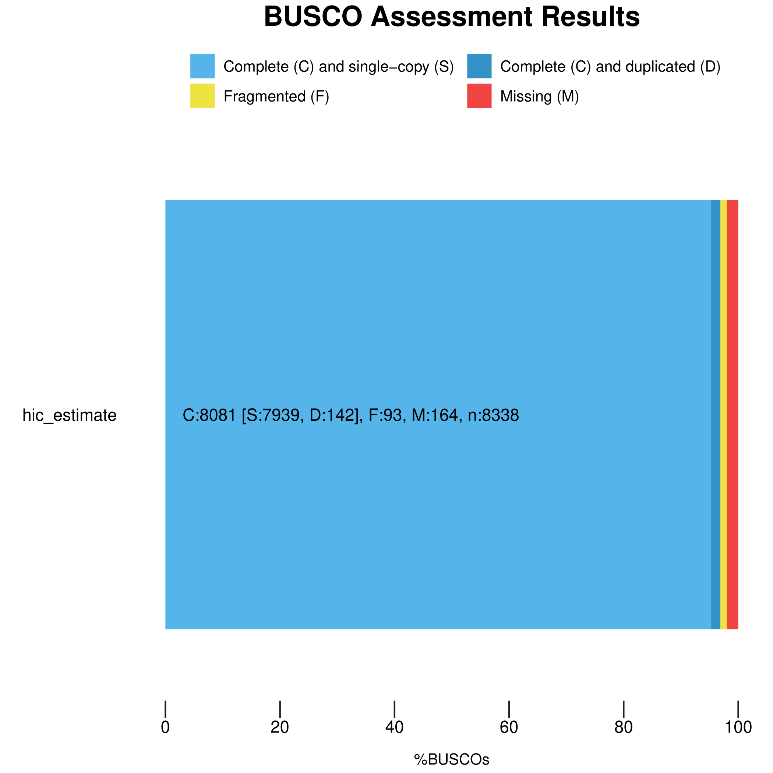


**Figure S2.** BUSCO assessment of the assembly genome of Lion-head goose.


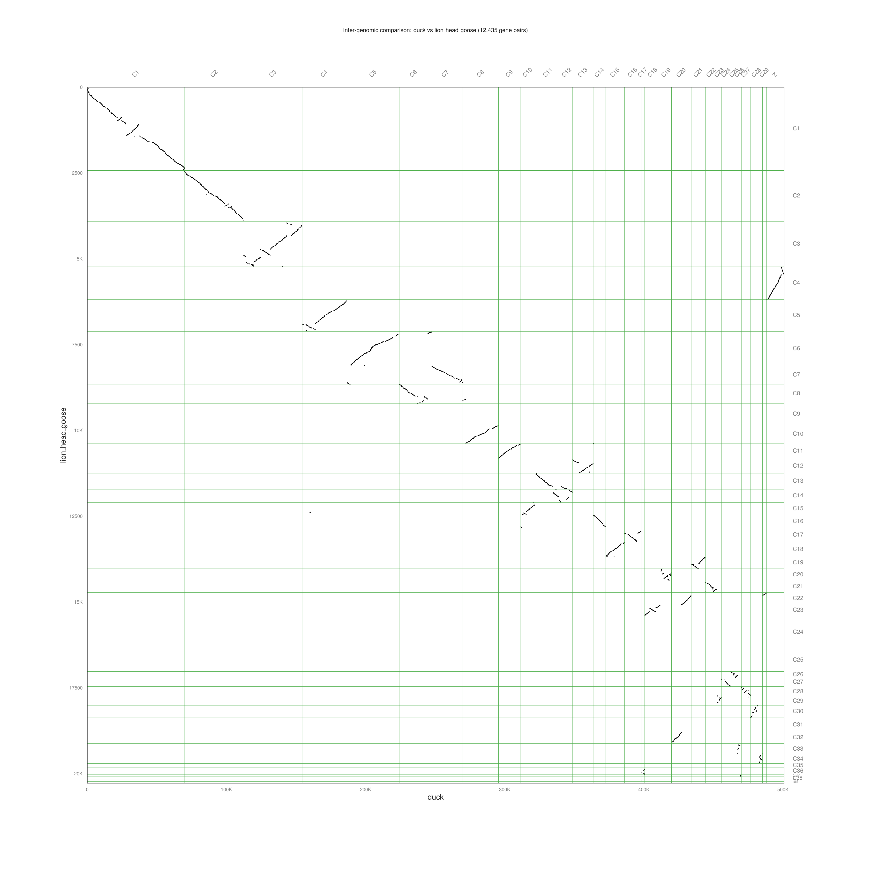


**Figure S3.** Gene synteny between the Lion-head goose and duck genomes.

**Table S1.** Statistics of sequenced clean data.

| Type | Base (Gbp) | Depth (×) | Sequenced platform |
| --- | --- | --- | --- |
| SMRT | 185.37 | 168 | PacBio Sequel Ⅲ |
| BioNano | 468.4 | 277.59 | Bionano Saphyr |
| Hi-C | 230 | 219 | Illumina HiSeq X Ten |
| Illumina | 111.25 | 108.6 | Illumina HiSeq |

**Table S2.** Statistics of genome survey.

| Type | Info |
| --- | --- |
| Genome size | 1,127,352,499 bp |
| Heterozygosity | 0.335% |
| Repetitive rate | 0.865% |
| GC content | 42.39% |
| Error rate | 0.202% |

| **Table S3:** Statistics of genome assembly quality. | | | | |
| --- | --- | --- | --- | --- |
| Method | Type | N50 | Number | Max length |
| PacBio | Contig | 13,732,492 | 1,859 | 57,632,554 |
| BioNano | Scaffold | 37,123,516 | 110 | 98,698,500 |
| Hi-C | Contig | 21,589,146 | 1,318 | 91,420,268 |
|  | Scaffold | 27,064,542 | 1,266 | 98,160,899 |

| **Table S4:** Summary of BUSCOs genome evaluation. | | |
| --- | --- | --- |
| Item | Number | Percent (%) |
| Complete BUSCOs (C) | 8081 | 96.9 |
| Complete and single-copy BUSCOs (S) | 7939 | 95.2 |
| Complete and duplicated BUSCOs (D) | 142 | 1.7 |
| Fragmented BUSCOs (F) | 93 | 1.1 |
| Missing BUSCOs (M) | 164 | 2.0 |
| Total BUSCO groups searched | 8338 | 100 |

| **Table S5:** Summary of gene families from several species. | | | | |
| --- | --- | --- | --- | --- |
| Animals | Expansion | Contraction | Unique | Total |
| Lion-head goose | 1,191 | 1,328 | 220 | 12,451 |
| Zhedong white goose | 53 | 1,465 | 2 | 12,106 |
| Chicken | 228 | 663 | 94 | 13,049 |
| Duck | 267 | 1,718 | 80 | 12,201 |
| Turkey | 582 | 911 | 46 | 12,829 |
| Pigeon | 171 | 694 | 21 | 12,454 |
| Saker | 128 | 710 | 7 | 12,427 |
| Titmouse | 83 | 1,215 | 15 | 12,407 |
| Lizard | 368 | 1,736 | 282 | 13,034 |

**Table S6.** GO annotation of expanded gene families from Anatidae varieties (Duck, Zhedong white goose, Lion-head goose; Top 20).

| GO ID | Class | Description | P.adjust | Count | Distribution |
| --- | --- | --- | --- | --- | --- |
| GO:0050911 | BP | detection of chemical stimulus involved in sensory perception of smell | 6.97E-08 | 10 | Duck (6); Zdg (4) |
| GO:0001847 | MF | opsonin receptor activity | 6.97E-08 | 4 | Duck (2); Zdg (2) |
| GO:0001850 | MF | complement component C3a binding | 6.97E-08 | 4 | Duck (2); Zdg (2) |
| GO:0004875 | MF | complement receptor activity | 2.87E-07 | 4 | Duck (2); Zdg (2) |
| GO:0002430 | BP | complement receptor mediated signaling pathway | 9.17E-07 | 4 | Duck (2); Zdg (2) |
| GO:0033864 | BP | positive regulation of NAD(P)H oxidase activity | 1.32E-06 | 4 | Duck (2); Zdg (2) |
| GO:0010759 | BP | positive regulation of macrophage chemotaxis | 4.05E-06 | 4 | Duck (2); Zdg (2) |
| GO:0072126 | BP | positive regulation of glomerular mesangial cell proliferation | 4.05E-06 | 4 | Duck (2); Zdg (2) |
| GO:0036094 | MF | small molecule binding | 4.45E-06 | 5 | Duck (2); Zdg (2); LHG (1) |
| GO:0071624 | BP | positive regulation of granulocyte chemotaxis | 6.36E-06 | 3 | Duck (2); Zdg (1) |
| GO:0005549 | MF | odorant binding | 1.47E-05 | 7 | Duck (4); Zdg (3) |
| GO:0030449 | BP | regulation of complement activation | 1.60E-05 | 4 | Duck (3); Zdg (1) |
| GO:1902947 | BP | regulation of tau-protein kinase activity | 1.85E-05 | 3 | Duck (2); Zdg (1) |
| GO:0090022 | BP | regulation of neutrophil chemotaxis | 2.11E-05 | 3 | Duck (2); Zdg (1) |
| GO:0005153 | MF | interleukin-8 receptor binding | 2.86E-05 | 3 | Duck (1); Zdg (1); LHG (1) |
| GO:1902624 | BP | positive regulation of neutrophil migration | 3.74E-05 | 3 | Duck (2); Zdg (1) |
| GO:0032494 | BP | response to peptidoglycan | 4.11E-05 | 3 | Duck (2); Zdg (1) |
| GO:0097242 | BP | amyloid-beta clearance | 5.16E-05 | 3 | Duck (2); Zdg (1) |
| GO:2000573 | BP | positive regulation of DNA biosynthetic process | 6.30E-05 | 4 | Duck (2); Zdg (2) |
| GO:0072593 | BP | reactive oxygen species metabolic process | 8.51E-05 | 4 | Duck (2); Zdg (2) |
| Note: BP - Biological process; CC - Cellular component; MF - Molecular function; Zdg - Zhedong white goose; LHG - Lion-head goose. The number in brackets in the DISTRIBUTION column is the number of gene families in the species that participate in the GO enrichment. | | | | | |

**Table S7.** GO annotation of contraction gene families from Anatidae varieties (Duck, Zhedong white goose, Lion-head goose; Top 20).

| GO ID | Class | Description | P.adjust | Count | Distribution |
| --- | --- | --- | --- | --- | --- |
| GO:0004396 | MF | hexokinase activity | 7.64E-26 | 10 | Duck (2); Zdg (4); LHG (4) |
| GO:0005536 | MF | glucose binding | 2.30E-22 | 10 | Duck (2); Zdg (4); LHG (4) |
| GO:0001678 | BP | cellular glucose homeostasis | 6.84E-18 | 10 | Duck (2); Zdg (4); LHG (4) |
| GO:0018212 | BP | peptidyl-tyrosine modification | 3.13E-17 | 12 | Duck (6); Zdg (6) |
| GO:0031234 | CC | extrinsic component of cytoplasmic side of plasma membrane | 4.26E-16 | 12 | Duck (6); Zdg (6) |
| GO:0006096 | BP | glycolytic process | 1.75E-15 | 9 | Duck (2); Zdg (4); LHG (3) |
| GO:0099091 | CC | postsynaptic specialization, intracellular component | 6.20E-15 | 8 | Duck (4); Zdg (4) |
| GO:0019318 | BP | hexose metabolic process | 2.66E-14 | 6 | Duck (2); Zdg (4) |
| GO:0007169 | BP | transmembrane receptor protein tyrosine kinase signaling pathway | 3.65E-13 | 10 | Duck (5); Zdg (5) |
| GO:0008271 | MF | secondary active sulfate transmembrane transporter activity | 6.98E-11 | 6 | Duck (2); Zdg (2); LHG (2) |
| GO:0036120 | BP | cellular response to platelet-derived growth factor stimulus | 4.30E-10 | 6 | Duck (3); Zdg (3) |
| GO:0002513 | BP | tolerance induction to self-antigen | 1.21E-09 | 4 | Duck (2); Zdg (2) |
| GO:0051156 | BP | glucose 6-phosphate metabolic process | 1.27E-09 | 5 | Duck (2); Zdg (3) |
| GO:0046835 | BP | carbohydrate phosphorylation | 1.68E-09 | 6 | Duck (2); Zdg (4) |
| GO:2000670 | BP | positive regulation of dendritic cell apoptotic process | 1.96E-09 | 4 | Duck (2); Zdg (2) |
| GO:0019531 | MF | oxalate transmembrane transporter activity | 2.23E-09 | 5 | Duck (1); Zdg (2); LHG (1) |
| GO:0019532 | BP | oxalate transport | 2.23E-09 | 5 | Duck (1); Zdg (2); LHG (1) |
| GO:0070667 | BP | negative regulation of mast cell proliferation | 2.23E-09 | 4 | Duck (2); Zdg (2) |
| GO:0015108 | MF | chloride transmembrane transporter activity | 3.64E-09 | 5 | Duck (2); Zdg (2); LHG (1) |
| GO:0046875 | MF | ephrin receptor binding | 4.14E-09 | 6 | Duck (3); Zdg (3) |
| Note: BP - Biological process; CC - Cellular component; MF - Molecular function; Zdg - Zhedong white goose; LHG - Lion-head goose. The number in brackets in the DISTRIBUTION column is the number of gene families in the species that participate in the GO enrichment. | | | | | |

**Table S8.** GO annotation of unique gene families from the Lion-head goose.

| GO | Class | Description | P.adjust | Count |
| --- | --- | --- | --- | --- |
| GO:0005681 | CC | spliceosomal complex | 1.34E-40 | 40 |
| GO:0000398 | BP | mRNA splicing, via spliceosome | 5.78E-34 | 40 |
| GO:0042981 | BP | regulation of apoptotic process | 5.78E-34 | 46 |
| GO:0004674 | MF | protein serine/threonine kinase activity | 1.24E-24 | 46 |
| GO:0004672 | MF | protein kinase activity | 6.85E-09 | 53 |
| GO:0006325 | BP | chromatin organization | 6.30E-07 | 10 |
| GO:0080019 | MF | fatty-acyl-CoA reductase (alcohol-forming) activity | 8.94E-05 | 6 |
| GO:0005869 | CC | dynactin complex | 8.11E-04 | 6 |
| GO:0007017 | BP | microtubule-based process | 5.28E-03 | 8 |
| GO:0007188 | BP | adenylate cyclase-modulating G protein-coupled receptor signaling pathway | 5.92E-03 | 4 |
| GO:0016887 | MF | ATPase activity | 9.76E-03 | 14 |
| GO:0019001 | MF | guanyl nucleotide binding | 2.82E-02 | 4 |
| GO:0031683 | MF | G-protein beta/gamma-subunit complex binding | 2.82E-02 | 4 |
