# Supplementary material for: Chromosome-level genome assembly of goose provides insight into the adaptation and growth of local goose breeds
Source: Gigascience. 2023 Feb 3;12:giad003. doi: 10.1093/gigascience/giad003 (PMC9896136; doi:10.1093/gigascience/giad003)
Supplement: giad003_Response_to_Reviewer_Comments_Original_Submission [file giad003_response_to_reviewer_comments_original_submission.pdf]

Author's Response To Reviewer Comments

Close

Dear editor,

Thank you very much for your letter dated 07 Apr 2022, and the reviewer's comments concerning our manuscript "Chromosome-level genome assembly of goose provides insight into the adaptation and growth of local goose breeds" (ID: GIGA-D-22-00016).

These comments are of great value and very helpful for revising and improving our paper, as well as the importance guiding significant to our research. According to your opinion and request, we have made some revisions to the original manuscript. The responses to the questions are shown below, the black bold font part is the questions raised by the reviewers, and the dark blue font part is our reply.

We have resubmitted the revised version in both PDF and MS word format, on the system for your review. The revised parts are marked in yellow in the MS word file of the revised manuscript for your review.

Should you have any questions, please contact us without hesitate.

Best wishes,
Xinheng Zhang

Questions and Responses:

Reviewer #1: Annotation and assembly of the Lion-headed goose genome were performed using a combination of four technologies, including Illumina, SMRT, Bionano, and Hi-C. Based on the chromosome-level genome sequence, a genome-wide association study (GWAS) was performed on 514 geese including Wuzong goose, Huangzong goose, Magang goose, and Lion-head goose, yielding an average of 1.52 Gb raw data, identifying 44,858 SNPs The GWAS results showed that six SNPs were significantly associated with body weight and 25 were potentially associated.
The authors should explore the spatial organization of chromatin and gene expression in the goose blood tissue (inter-pseudo-chromosomal interaction patterns, compartments, topologically associating domains, and promoter-enhancer interactions), to check if the goose genome shows similar basic principles to other animal genomes in terms of its inter-chromosomal interaction pattern, compartments, topologically associating domains, and promoter-enhancer interactions. Providing basic characterization of the three-dimensional organization of the goose genome, and supports the conclusion the goose genome assembly is chromosomal-level. For the four goose population, the authors should perform selective sweep analysis (Fst、XP-CLR、π or Tajima's D), and combine with the GWAS results to illustrate the topic of the this article.
Response: Thank you very much for your questions and suggestions. The purpose of this article is to construct a complete genome map of the Lion-head Goose and to analyze the evolutionary relationships with other avian species, and to determine the genomic level variations in the selection of Lion-head, Magang, Huangzong and Wuzong geese. In response to your questions, we made the following replies.
1. The question of spatial organization of chromatin and gene expression in the goose blood you raise is a good point, and we have reviewed the relevant literature and are concerned that this issue has been investigated in the chromatin in goose liver tissue of Tianfu goose [1], chicken embryonic fibroblasts (CEF) and adult erythrocytes [2], suggesting the additive effects of enhancers on the transcriptional levels of target genes, and the important role of topologically associated domains (TADs) as genomic regulatory units, respectively. The aim in this article is to construct the Lion-head goose genome and perform evolutionary and population analysis. The analysis of the samples collected in this study did not perform blood whole transcriptome analysis, so we could not perform the analysis of spatial organization of

chromatin and gene expression. But your comments are constructive and we will focus on your suggestions for further study in the next article.
2. The diploid cotton genome researchers used Hi-C technology to anchored and oriented 1,573 Mb of assembly to 13 pseudochromosomes [3]. The chromosome-level genome assembly of scimitar-horned oryx, generated 29 chromosomes using 10X Chromium sequencing and Hi-C technology [4]. The genome of autopolyploid sugarcane Saccharum spontaneum L. was assembled using a Hi-C-based physical map to obtain 32 pseudochromosomes [5]. The Musa Balbisiana Genome constructed a high-throughput chromosomal conformational capture (Hi-C) library with 430 Mb (87.27%) of assembly and 94.0% of genes placed on 11 chromosome groups [6]. The above examples fully demonstrate that the construction of chromosomal level genome by Hi-C is feasible and credible, and it is a widely used assembly method. Most researchers use Hi-C technology to illustrate genomic chromosome number. In this study, the Hi-C method we used to construct chromosomal level genomes is also an assisted assembly method based on Hi-C sequencing results, showing the three-dimensional structural characteristics of goose genomes.
3. For the four goose populations, we performed selective sweep analysis and combine with the GWAS results. Related description was added in L227-232, L341-365.

Reference:
[1] Li Y, Gao G, Lin Y, Hu S, Luo Y,Wang G, et al. Pacific Biosciences assembly with Hi-C mapping generates an improved, chromosome-level goose genome. Gigascience 2020;9:
[2] Fishman V, Battulin N, Nuriddinov M, Maslova A, Zlotina A,Strunov A, et al. 3D organization of chicken genome demonstrates evolutionary conservation of topologically associated domains and highlights unique architecture of erythrocytes' chromatin. Nucleic Acids Res 2019;47:648-65
[3] Du X, Huang G, He S, Yang Z, Sun G,Ma X, et al. Resequencing of 243 diploid cotton accessions based on an updated A genome identifies the genetic basis of key agronomic traits. Nat Genet 2018;50:796-802
[4] Humble E, Dobrynin P, Senn H, Chuven J, Scott A F,Mohr D W, et al. Chromosomal-level genome assembly of the scimitar-horned oryx: Insights into diversity and demography of a species extinct in the wild. Mol Ecol Resour 2020;20:1668-81
[5] Zhang J, Zhang X, Tang H, Zhang Q, Hua X,Ma X, et al. Publisher Correction: Allele-defined genome of the autopolyploid sugarcane Saccharum spontaneum L. Nat Genet 2018;50:1754
[6] Wang Z, Miao H, Liu J, Xu B, Yao X,Xu C, et al. Musa balbisiana genome reveals subgenome evolution and functional divergence. Nat Plants 2019;5:810-21

Line 27, "and identifying 21,208 protein-coding genes". Previous studies have shown that there are 16,150, 16288 and 17568 genes in Zhedong White goose, Sichuan white goose and Tianfu goose genome, respectively, please illustrate reason why the gene number were different the results from previous studies.
Response: Thank you for your comments. This is because of the differences of each species itself. By the previous studies, we can see that the number of genes is different among Zhedong White Goose, Sichuan White Goose and Tianfu Goose. The genome size of the Lion-head Goose in our present study is even larger than the former three, so it is normal to have a higher number of genes. During the process, we have performed TE filtering on the gene annotation.

Line 27, "…generating 40 pseudochromosomes", the assignment of 40 chromosomes to Hi-C scaffolds is very tentative and needs to be validated, the 40 pseudo-chromosomes do not equate to the 40 physical chromosomes. Moreover, the result is conflict with the 39 pseudochromosomes in Tianfu goose genome, how did the authors confirm the number of chromosomes?
Response: Thank you for your comments. According to the analysis results of Hi-C sequencing of spatial genome, there are indeed 40 chromosomes. Others are described in the second part of the first question: The diploid cotton genome researchers used Hi-C technology to anchored and oriented 1,573 Mb of assembly to 13 pseudochromosomes. The chromosome-level genome assembly of scimitar-horned oryx, generated 29 chromosomes using 10X Chromium sequencing and Hi-C technology. The genome of autopolyploid sugarcane Saccharum spontaneum L. was assembled using a Hi-C-based physical map to obtain 32 pseudochromosomes. The Musa Balbisiana Genome constructed a high-throughput chromosomal conformational capture (Hi-C) library with 430 Mb (87.27%) of assembly and 94.0% of genes placed on 11 chromosome groups. The above examples fully demonstrate that the construction of chromosomal level genome by Hi-C is feasible and credible, and it is a widely used assembly method. Most researchers use Hi-C technology to illustrate genomic chromosome number. In this study, the Hi-C method we used to

construct chromosomal level genomes is also an assisted assembly method based on Hi-C sequencing results, showing the three-dimensional structural characteristics of goose genomes.

Line 33, "…an average of 1,520.6 Mb of raw data with detecting 44,858 SNPs". Based on whole-genome resequencing data, researchers have identified 9,279,339 SNPs in the goose genome using an average depth of 12.44× whole genome resequencing data. Referring to SNP number, it is uncertain whether the results in this study (44,858 SNPs generated from 1,5Gb data) is correct. Therefore, the authors should apply the BWA-GATK pipeline to Tianfu meat goose and Lion-head goose for GWAS analysis to determine whether the results are correct.
Response: Thank you for your comments. The sequencing data of the Lion-head goose population is from enzyme-based RAD-Seq, and the average sequencing depth is 12.44×. Due to the depth of sequencing coverage, it is normal to detect differences in the number of SNPs. In addition, the genome assembly of Tianfu goose has been completed by Chongqing Academy of Animal Husbandry, and the assembly data can be found on NCBI, but no re-sequencing data of Tianfu goose could be found on NCBI. Therefore, there is currently no way to conduct genome-wide association analysis between Tianfu meat goose and Lion-head goose. Furthermore, the Tianfu goose is a cultivated breed, while the Lion-head goose is a native breed, and a comparative analysis of the two does not seem to yield a relatively accurate result.

Line 61 to 65, It is recommended to rewrite or replace the descriptions for the goose breeds with methods sections.
Response: Thank you for your comments. We refined the phenotypic descriptions of the four goose species.

Methods section
Line 88, Provide a detailed description of the picture(s) for the Lion-head goose to display the "classical trails". Please supply the pictures for the four goose breed (Wuzong goose, Huangzong goose, Magang goose and Lion-head goose) to help the more clear the understanding of design.
Response: Thank you for your comments. Due to the limitation of conditions, it is difficult for us to take photos of four goose breed in the same background. Therefore, we use text to describe the characteristics of four goose species in the INTRODUCTION, and show pictures of individual Lion-head goose. The description is as follows: The Lion-head goose has a large body, a deep and wide head, and large sarcomas (five sarcomas) on the front and side of the face. The adult male goose weighs 9-10 kg and the female goose 7.5-9 kg, grows rapidly and has rich muscles. Wuzong goose is a small goose species with a distinct band of black plumage from neck to back. The gander weighs 3-3.5kg and the female weighs 2.5-3kg, with wide and short body, flat back, and thin and short feet. Magang goose is a medium-sized goose species, with a long head, wide beak, rectangular body, a gray-black bristle-like feathers on the back of the neck, gray brown breast feathers and white belly feathers. Adult weight is 4-5 kg for males and 3-4 kg for females. Huangzong goose has a compact body, from the top of the head to the back of the neck has a brownish yellow feather belt, shaped like a horse's mane. The chest feather is gray yellow, the belly feather is white, the beak and sarcoma are black. Adult males weigh 3-3.5 kg, females 2.5-3 kg. Displayed in L64-74.

Line 91 to 92, "from another four healthy adult accessions were collected for RNA-seq analysis", please rewrite the sentence since it is unclear.
Response: Thank you for your comments. We have modified it as “another four healthy adult individuals”.

Supply the detail information for GWAS analysis, including the software, models What parameters were used to run GATK, plink, BWA? did the authors performed GWAS analysis using plink software, rather than GEMMA, TASSEL or other software ?
Response: Thank you for your suggestions. We have supplemented the relevant software parameters and correlation analysis model, showing in L212-232.

line 200 "the results of the assoc and linear analyses were…", supply the detail of GWAS analysis, including the software, analysis model. please provide more detailed information about the models and assumptions.
Response: Thank you for your suggestions. We have supplemented the relevant data.

What the top 20 PCs? Did the PCs paly an important role in GWAS analysis?
Response: Thank you for your comments. The top 20 PCs are the top 20 principal components after PCA

analysis based on genetic variant information, and top 20 PCs were used as covariables for GWAS to reduce the interference of population structure on results.

Detailed information is not given in several parts of this paper, especially the methodology. How many individuals from the four-goose population? The GWAS analysis were performed in one goose population or the four-goose population? How did the authors do the GWAS analysis and annotation the SNPs? please supply detail analysis steps and analysis models, software. For GWAS analysis model, were there any family or environmental effects? how did you test the significance of the random variables? Many sentences are not clear all over the entire manuscript and need to be re-writen. For instance, line 201, "The corresponding genes of significantly related SNPs were used to identify the GO pathway", define the corresponding genes, and how did the GO pathway analysis?
Response: Thank you for your suggestion, we have polished the points you mentioned, please see the manuscript marked yellow.

Line 203, please rewrite the statistical analysis section to provide more detail. For example, authors should define "potential associated" in this section.
Response: Thank you for your comments. Based on the opinions of other reviewers, we deleted this section and integrated relevant information into other analysis section.

Line 283 to 284, "…correlated with any chromosome of the duck genome due to the presence of a large number of tandem repeats". Provide the detail data or the figure(s) to support your claim.
Response: Thank you for your suggestions. We are very sorry that we have described it wrong here. The collinearity analyses use blocks of several genes as basic units to determine whether regions of two genomes are homologous. Therefore, we change to "…were not correlated with any chromosome of the duck genome maybe due to the heterogenous of genes on the chromosome".

Results section
Compare with the quality metrics of this study with the previous four goose genome, including contig N50, scaffold N50, gene number, Repetitive regions proportion of genome, etc.
Response: Thank you for your comments, we have added Table 2 to compare the assembled genomes of the four goose species (i.e., Zhedong white goose, Sichuan white goose, Tianfu goose and Lion-head goose).

For gene annotation, the authors did not perform the none coding RNA in the goose genome, please supply the analysis.
Response: Thank you for your comments. In this study, mRNA was used for transcriptome sequencing. Sequencing of the none coding RNA was not performed, so annotation analysis of none coding RNA could not be performed. Your suggestions are very constructive, and we will continue to conduct in-depth studies from the whole transcriptome in the future.

The author(s) should perform the positive selection genes analysis with the avian chromosome genomes, such as chicken, duck, zebra finch, etc.
Response: Thank you for your comments. We have conducted gene family expansion and contraction analysis of some avian species that already reflect the selection of functions in various avian species during evolution. We have done the correlation analysis, and the relevant description is in the Line 285-303 of the article. Described as follows: Moreover, we mixed the gene family sets of several Anatidae varieties (duck, Zhedong white goose, Lion-head goose), and performed expansion and contraction analysis and corresponding GO enrichment analysis. In this task, the GO analysis of expanded gene families suggested the olfactory perception, such as detection of chemical stimulus involved in sensory perception of smell (GO:0050911, $p = 6.97\times10\text{-}8$), and odorant-binding (GO:0005549, $p = 1.47\times10\text{-}5$), both of which may be related to the adaptation of the species to find food in water. Meanwhile, contracted gene families were concentrated in the areas of glucose synthesis and metabolism, such as hexokinase activity (GO:0004396, $p = 7.64\times10\text{-}26$), glucose binding (GO:0005536, $p = 2.30\times10\text{-}22$), cellular glucose homeostasis (GO:0001678, $p = 6.84\times10\text{-}18$), glycolytic process (GO:0006096, $p = 1.75\times10\text{-}15$), hexose metabolic process (GO:0019318, $p = 2.66\times10\text{-}14$), carbohydrate phosphorylation (GO:0046835, $p = 1.68\times10\text{-}9$), and glucose 6-phosphate metabolic process (GO:0051156, $p = 1.27\times10\text{-}9$), which may be closely related to characteristics of glycogen storage and utilization during migration. Besides, 220 unique gene families

(other species lack these gene families) of the Lion-head goose were identified and functionally annotated in GO categories, such as protein kinase activity (GO:0004672, p = 6.85×10-9), the regulation of apoptotic process (GO:0042981, p = 5.78×10-34), the adenylate cyclase-modulating G protein-coupled receptor signaling pathway (GO:0007188, p = 5.92×10-3), and fatty-acyl-CoA reductase (alcohol-forming) activity (GO:0080019, p = 8.94×10-5).

Please supply the detail information of the 40 pseudo-chromosomes for the goose genome assembly.
Response: Thank you for your comments. In fact, we have already uploaded the raw data to the GigaSciences as requested, and provided the circos plot with the corresponding data including the GC content of 40 chromosomes, gene abundance and other information. Meanwhile, we have uploaded the data to the submission system, please check.

Please show the summary of the economic traits used in this study, including the mean, stand error, numbers of individuals, breed, male or female.
Response: Thank you for your comments. We have added tables of descriptive statistics for different goose weights, as shown in Table 3.

line 233-234, "The aggregate of 760 Gb raw reads was accumulated by the paired-end sequencing of the 36 constructed libraries", Why did the authors conduct 760 Gb RNAseq? It is obvious too much larger than previous goose genome annotation, did they perform more analysis?
Response: Thank you for your suggestions. We performed RNA-seq to assist genome assembly and increase the credibility of genome annotations. The 760 Gb of data is composed of 4 individuals, 8 tissues and blood for a total of 36 samples combined, and we have uploaded the transcriptome data to NCBI.

Line 286 to 287, "Chr 4 of Lion-head goose was found to correspond to the sex chromosome Z of duck, except for the inversions of small patches of segments; therefore, we inferred that Chr 4 was the sex chromosome of the Lion-head goose", To better understand the unique biological characteristics and breeding of geese, it is essential to distinguish the sex chromosomes from the autosomes. For updating the sequence of Z and W chromosomes, it is recommended to filter the sequence of autosomes using experimental methods. How did the authors filter autosomal sequences in the Chr4? Moreover, the W chromosome sequence should be identified similarly to the Z chromosome. Authors should identify the Z and W chromosome sequence from public databases based on the Z and W chromosome sequence from the chromosome-level avian genome.
Response: Thank you for your suggestions. The aim of this study was to construct a complete and accurate genome map of Lion-head goose, and to analyze evolutionary relationship with avian species, and to determine the changes of genome level of different goose species during the selection and breeding process. Refinement analysis and confirmation of sex chromosomes are not the focus, but your suggestion is interesting, and we are curious about it, and we will focus on it in the future. Additionally, this study was used for genome assembly in male Lion-head goose, which have been described at Methods - Animal selection, and males are without W chromosomes.

Line 292-294, "and their weight was recorded, with the Lion-head goose using the minimum weight, the Wuzong goose using the maximum weight, and the Huangzong goose and Magang goose using the average weight." Why did the authors select the body weight trait? The artificial selection would lead to the inaccurate GWAS results.
Response: Thank you for your comments. In this study, we did not make artificial selection. We only selected four domestic geese from Guangdong province of China for the association analysis of body weight and SNP. The difference in body weight was due to the goose species themselves, e.g., Lion-head geese weighing more than 9 kg, the Wuzong geese 1.8-2.5kg, Huangzong geese 2.7-4.3kg, and Magang geese 4.8-5.5kg. These weight ranges were obtained from the samples collected, without any artificial selection involved.

From figure 5A, there are significant population stratification in Lion goose population (obvious clustering 2 clusters), how did the authors sure to provide accurate GWAS results? Did the author detect the SNPs associated with body weight in the goose population to test the accurate of GWAS results? The discussion tends to be mere story telling.
Response: Thank you for your suggestions. The stratification of the Lion-head geese mainly due to gender

differences. Using PCA analysis to obtain the top 20 principal components as covariables for GWAS, which can reduce the interference of group structure on the results and eliminate the influence of group stratification on the statistical results of GWAS as much as possible. Based on your suggestions, we have revised the discussion to make it more specific and in-depth.

Tables and Figures
In table 1, the "Hi-C" results is repeat with the "Assembly", please modify it.
Response: Thank you for your suggestions. We have modified.

The table 2-4, Figure 1-2, are not very informative and I suggest moving these to the supplementary information.
Response: Thank you for your suggestions, we have adjusted all the figures and tables.

Reviewer#2: In this manuscript titled "Chromosome-level genome assembly of goose provides insight into the adaptation and growth of local goose breeds", Zhao et al. Based on PacBio, Bionano and Hi-C technologies, they report a chromosome-level genome assembly of Lion-head goose (Anser cygnoides).The assembly had a total genome size of 1.19 Gb, consisting of 1,859 contigs with an N50 length of 20.59 Mb, generating 40 pseudochromosomes, representing 97.27% of the assembled genome, and identifying 21,208 protein-coding genes. To identify genetic markers associated with body weight in different geese breeds including Wuzong goose, Huangzong goose, Magang goose and Lion-head goose, a genome-wide association study was performed, yielding an average of 1,520.6 Mb of raw data with detecting 44,858 SNPs. GWAS showed that six SNPs were significantly associated with body weight and 25 were potentially associated. The writing of the paper is mostly clear, except a few things I would like to have them clarified (or re-written):
(1) Line 209, "sequencing strategies" should be replaced with "sequencing and genome assemble strategies".
Response: Thank you for your suggestions. We have revised it according to your suggestion.

(2) Line 210, "Hi-C" should be replaced with "Hi-C approach".
Response: Thank you for your suggestions. We have modified it according to your suggestion.

(3) Line 233, "four healthy adult animals" should be replaced with "four healthy adult individuals".
Response: Thank you for your comments. We have modified it according to your suggestion.

(4) Line 243-244, "eight bird species (Lion-head goose, Zhedong white goose, duck, turkey, chicken, pigeon, saker, and titmouse) and green lizard". Please give the very exact Latin name and the article published after the sequencing of the genome of the eight bird species.
Response: Thank you for your comments. We have added the very exact Latin name.

(5) Line 273, gene name (Sterile) should be italic.
Response: Thank you very much for your comments and suggestions. We have revised the above problems and marked them in yellow in the text.

(6) Line 296, "The average raw data was 1,520.60 Mb". What kind of data is this raw data?
Response: Thank you for your comments. We may not have elaborated well in the original manuscript; this kind of data is about the resequencing data of 514 goose blood samples. And we have revised it as follows: Blood from each sample was used for paired-end 100 resequencing. And the average raw data was 1,520.60 Mb. Marked yellow on L327.

(7) Lines 290-306, The logic of paragraph Cluster analysis of different goose species was confused. What is the purpose of this analysis?
Response: Thank you for your comments. This part of the study was conducted by whole-genome resequencing of blood samples from four goose populations to identify genetic variants and demonstrate the great phenotypic differences among the four goose populations by PCA and phylogenetic trees, followed by genome-wide association analysis of SNPs with body weight of different goose species to mine the available functional SNPs.

(8) Line 299, please use uniform scientific notation, 10e-7 should be ×10-7.
Response: Thank you for your comments. We have refined this.

(9) The PCA results in Figure 5A need a statistic test.
Response: Thank you for your comments. The main purpose of PCA analysis is to visualize between- and within-group differences in subgroups, and top PC20 of PCA is also used for statistical analysis of GWAS to reduce the interference of group structure on the results.

(10) Lines 258-271, 310, 317, Pvalue or P? The form in the whole manuscript should remain uniform.
Response: Thank you for your comments. We have performed a full-text check of the Pvalue and unified them as “p”.

Reviewer #3: Zhao et al.
Title: "Chromosome-level genome assembly of goose provides insight into the adaptation and growth of local goose breeds"
- please place figures and tables within the text, not at the end: this makes it difficult to read and review the article (From editors: you can disagree this comment)
- English: needs to be improved
Response: Thank you for your comments. The English of this manuscript has been improved according to your suggestion.

- Figures: low quality, low resolution --> hard to read. Additionally, Figure legends/captions are separate from Figures --> difficult reading
Response: Thank you for your comments. We have uploaded the high-definition images and put them together with the legends.

Introduction

L44: what do you mean with "the majority of birds"?
Response: Thank you for your comments. It means most birds of Anseriformes, and we have polished it.

L51: warmth retention of the birds?
Response: Thank you for your comments. We changed it to “the warmth properties of feather products”.

L63: " ... while IN the Wuzong goose ... the average weight is ..."
Response: Thank you for your comments. We have modified it.

L67: how is an accurate reference genome essential to decipher the industry's development? Which industry?
Response: Thank you for your comments. We revised it to “improving production efficiency and even promoting the development of goose industry.”

L73: maybe it's new scaffolding techniques
Response: Thank you for your comments. We have modified it.

L80-81: here you mention two sequencing technologies (SMRT, Illumina NGS), one scaffolding method (Hi-C) and one unspecified technology/methods by Bionano (which was not mentioned earlier). Please rephrase and be more specific and clearer
Response: Thank you for your comments. Thank you for your comments. We have described the Bionano optical mapping technology as described below: Bionano optical mapping technology has advantages in obtaining highly repetitive sequences and detecting genomic structural variants, which is helpful for remote sequencing of sequence overlap clusters. Bionano has become a powerful tool for genome assembly, a 5.1 Mbp inversion was found in the genomes of a patient with Duchenne muscular dystrophy. Displayed in L88-91.

L81: correlation of body weight with what?
Response: Thank you for your comments. Correlation of body weight with genetic variations.

Methods

L119-181: how were Bionano maps used to improve the quality of your genome assembly?
Response: Thank you for your comments. This sentence is a general introduction to the whole text, and we describe the relevant methods and parameters after the sentence: “Afterward, the data were assembled with RefAligner and Assembler of BioNano Solve. The scaffold was established using BioNano Solve with HERA’s contigs and a BioNano genome map. When encountering a conflict between a contig and the genome map, the contig was split to correct the false connection”.

L136: adult accessions?
Response: Thank you for your comments. We have changed “adult accessions” to “adult Lion-head goose”.

L142: how were low-quality reads defined? Based on average Phred scores?
Response: Thank you for your comments. Adaptors and low-quality reads of raw data were removed using Trimmomatic and the sequence will be trimmed according to the base quality value (i.e. Phred scores) with the following parameters: LEADING:3 TRAILING:3 SLIDINGWINDOW:4:15 -threads 20 MINLEN:50, and other default.

L143: what do you mean by polluted reads?
Response: Thank you for your comments. Polluted reads are erroneous sequences such as those containing more than 5% of N base due to systematic errors.

L143: what do you mean with "Trinity was arranged"?
Response: Thank you for your comments. We have changed “Trinity was arranged” to “Trinity was used”.

L148-150: poor English, please rephrase. Additionally, more details are needed on the quality metrics used to evaluate the assembled genome
Response: Thank you for your comments. The reference library used for BUSCO evaluation is aves_odb10, and the rest of the parameters are BUSCO default, so no detailed parameters are listed.

L164-165: could you add the scientific names (genus species) of the mentioned avian species? (green lizard is not an avian species)
Response: Thank you for your comments. We have made changes and additions, and the changes are as follows "we compared the gene families of Lion-head goose with the genomes of the following avian species: Zhedong white goose (Anser cygnoides), duck (Anas platyrhynchos), turkey (Meleagris gallopavo), chicken (Gallus gallus), pigeon (Columba livia), saker (Falco cherrug), titmouse (Pseudopodoces humilis), and green lizard (Anolis carolinensis).”

L173: from where did you get the divergence time between turkeys and pigeons? (~100 million years? Really?) And why did you choose this specific divergence value for calibration?
Response: Thank you for your comments. Divergence time data were obtained from the website http://www.timetree.org/. Pigeon and turkey divergence times were chosen as controls because these two species are genetically distant in avian species and have lower correction errors.

L173: the r8s software was served to estimate: bad English
Response: Thank you for your comments. We have changed “served” to “used”.

L179: "Experimental sample processing and genotyping" this is a bit unclear: you already took biological samples, maybe you need to highlight that this is genotyping (your title should be more about genotyping and phenotyping for GWAS, since you spend the first few lines of the paragraph to describe the phenotypes)
Response: Thank you for your comments. We have changed “Experimental sample processing and genotyping” to “Experimental sample processing and variant detection for Genome-wide association study”.

L181-185: body weight is naturally a continuous trait, it would be rather arbitrary to split it into categories: therefore I don't understand this whole bit on categorical vs continuous body weight

Response: Thank you for your comments. This may be due to our choice of species, each species has a wide range of body weight, but basically within a relatively fixed range. The detailed values are as follows: 9-14 kg for Lion-head goose, 2.7-4.3 kg for Huangzong goose, 1.8-2.5 kg for Wuzong goose, and 4.8-5.5 kg for Magang goose. Although there seems to be some stratification, we still consider their weight as a continuous variable.

L186-190: what you describe is RAD-sequencing/GBS/resequencing, not "genotyping". By genotyping usually an array-based approach is meant
Response: Thank you very much for your professional opinion. We have improved it by removing the description of “genotyping” and modifying it as follows: “Then variant detection as well as genotyping was performed using Samtools, GATK4 software”.

L188: how did you define low quality reads here? (Phred scores?) No filters on average reads coverage per site?
Response: Thank you for your comments. Low quality threshold parameters set to 5.

L191: it is not clear which variants were called? SNP? MNP? Indels? All? etc.
Response: Thank you for your comments. It means SNP variants, and we have modified in the manuscript.

L191: why did you set the MAF threshold at 5%? You have 514 samples, with a filter at MAF 1% you'd still have more than 10 copies of the minor allele in the worst case scenario
Response: Thank you for your comments. Because MAF threshold at 5% is a conventional threshold, this is to ensure that this SNP analysis remains consistent with previous and subsequent analyses, and does not introduce additional systematic errors.

L192: maximum deletion threshold? Is this max missing rate?
Response: Thank you for your suggestion, we have modified it.

L192-193: what was the objective of PCA? PCA on which data? (I guess the genotype data? Which?)
Response: Thank you for your comments. In this study, PCA was performed based on SNP variation information to extract the main principal components, and the top principal components were used as dependent variables for genome-wide association analysis.

L193-194: "To understand the kinship among the samples, and phylogenetic trees were constructed." This sentence seems wrong/incomplete
Response: Thank you for your comments. We made the following changes: To understand the kinship among the samples, and the phylogenetic trees were constructed using SNP data with Phylip software.

L196: maybe you mean genetic variation?
Response: Thank you for your suggestion, we have modified it.

L197: did you use the --linear option in Plink?
Response: Thank you for your suggestion, GWAS analysis was done for both the assoc model and the linear model in Plink.

L197-199: this sentence is poorly written, please rephrase
Response: Thank you for your comments. We modified it to “The top 20 PCs in the PCA analysis were used as covariates, and regression analysis was performed on sample variances with corresponding weight information by Plink.”

L199: I guess its the variants, not the variances (if it is SNPs, please say SNPs)
Response: Thank you for your comments. We modified it to “SNPs”.

L196-200: I think it would be better if you wrote the GWAS model explicitly (the model equation)
Response: Thanks to your suggestion, we have added the model to line 212-232.

L200: why did you choose Bonferroni correction over other methods to control for spurious results (e.g.

FDR, Bayesian odds, permutation test, q-values etc.)
Response: Thank you for your comments. Because the Bonferroni correction is more rigorous, it is more effective in removing false positive results.

L202: this part is useless, as it is: which statistical analysis? Why did you choose the 0.05 threshold for significance? (you just said above that you used Bonferroni corrected p-values for GWAS)
Response: Thank you for your suggestion, we have removed this section.

Results

L211: "Assemble these data step by step and produce progressively improved assemblies (Fig. 1A)." This sentence seems incomplete or wrong
Response: Thank you for your comments. We modified it to “We assemble these data step by step and produce generate progressively improved assemblies assembled genome”.

Close
