# Supplementary material for: Chromosome-level genome assembly of goose provides insight into the adaptation and growth of local goose breeds
Source: Gigascience. 2023 Feb 3;12:giad003. doi: 10.1093/gigascience/giad003 (PMC9896136; doi:10.1093/gigascience/giad003)
Supplement: giad003_Response_to_Reviewer_Comments_Revision_1 [file giad003_response_to_reviewer_comments_revision_1.pdf]

Author's Response To Reviewer Comments

Close

Dear editor,

Thank you very much for your letter dated 08 Aug 2022, and the reviewer's comments concerning our manuscript "Chromosome-level genome assembly of goose provides insight into the adaptation and growth of local goose breeds" (ID: GIGA-D-22-00016).

These comments are of great value and very helpful for revising and improving our paper, as well as the importance guiding significant to our research. According to your opinion and request, we have made some revisions to the original manuscript. The responses to the questions are shown below, the black font part is the questions raised by the reviewers, and the dark blue font part is our reply.

We have resubmitted the revised version in both PDF and MS word format, on the system for your review. The revised parts are marked in yellow in the MS word file of the revised manuscript for your review.

Should you have any questions, please contact us without hesitate.

Best wishes,
Xinheng Zhang

Questions and Responses:
Reviewer #3:
L44: most birds of the Anseriformes order
Response: Thank you for your suggestion, we have made the changes.

>L51: warmth properties still doesn't sound right: maybe thermal (or thermic?) properties?
Response: Thank you for your suggestion. We have changed it to "natural stuffing for warm clothing and bedding".

>L76: what do you mean with "continuous reference genome"?
Response: Thank you for your comments. The "continuous reference genome" means that the genomic contigs obtained in this study are fewer in number and longer in sequence length than the reported assembled goose genome.

>L77-78: the link between the reference genome and the development of the goos industry is still loose: maybe you want to say that a complete and more accurate genome would make it possible to develop better tools for good breeding (e.g. genetic markers for marker-assisted selection, genomic breeding values, precise estimates of inbreeding, relatedness matrices between individuals etc.) Is this what you have in mind?
Response: Thank you for your comments. Yes, you said what we needed to say, and we changed the original text as follows: "… and even develop better tools for breeding to promote the development of goose industry."

>L130: replace "At last" with Finally
Response: Thank you, we have made the changes.

>L139: how was the contig split? Based on which criteria? (e.g. one half aligned in one region of the genome, the other half aligned somewhere else on the genome?)
Response: Thank you for your comments. The program "hybridScaffold.pl" in the BioNano Solve package

was used to merge the HERA’s contig with BioNano CMAP. When there is a conflict, the program split the HERA’s contig as the setting parameter of -B 1 -N2. “-B 1” means that it does not split the CMAP, and “-N 2” means that it split the contig at the conflict site. We have provided more detailed notes in the manuscript.

>L156: why do you say "polluted reads"? Do you mean contaminated samples? Do you have evidence that some of your samples were contaminated (i.e. external non-goos DNA)? uncalled nucelotides (the N's) can arise also from reading errors when generating the reads.
Response: Thank you for your comments. The “polluted reads” mean adaptor-polluted reads, but not contaminated samples. We have revised the sentence “Low-quality reads based on Phred scores, adaptor-polluted reads containing >5 adapter-polluted bases, and those containing N > 5% were trimmed, using the following parameters: LEADING:3 TRAILING:3 SLIDINGWINDOW:4:15 -threads 20 MINLEN:50” in L155-157.

>L163-164: "quality control for the assembly's quality, accuracy, and integrity was predicted": it is not clear what you predicted, please clarify (and write in better English please)
Response: Thank you for your suggestion. The gene set from the BUSCO v5.3.0 database was used to assess the confidence and completeness of the final genome assembly. A higher ratio of the mapped intact genes in the assembled genome means a higher completeness of the assembling. In addition, we have changed the word "predicted" to the more accurate word "assessed". See L163-165 for details.

>L165: at least say that you used default parameters (and add a reference to these, e.g. the online manual)
Response: Based on your suggestion, we have updated the parameters and references, with the following modifications in L166: using aves_odb10 as the query with parameters: -l aves_odb10 -m genome -c 5 [29, 30]. See L163-165 for details.

>L203: what is this low quality parameter? Some sort of modified Phred? (A Phred threshold of 5 would be a bit low, allowing many errors -wrong bases- in the analysis)
Response: Thank you for your comments. After checking the script, we found that the threshold parameter was set to 20, but not the default value (5). We have corrected the mistake.

>L209: maybe it is better to write "To understand relationships among groups of samples, the phylogenetic ..."
Response: Thank you for your comments. We have revised this.

>L212: corresponding BODY weight
Response: Thank you. We have corrected it.

>L213: Wald test is one of many possible statistical tests to assess the significance of SNP effects from the results of the linear regression model used for the association study
Response: Thank you. We have revised this.

>L213: The top 20 principal components PCs) from the principal components analysis (PCA) of SNP variant data were used as covariates in the model used for the association study.
Response: Thank you for your comments. The top 20 principal components (PCs) based on the principal components analysis (PCA) of SNP variant data were used as covariates, and subjected for the association study.

>L214: you can delete this (you already mentioned Plink, or can mention Plink at the end of the GWAS section)
Response: Thank you for your suggestion. We have deleted this sentence.

>L215-216: this is written in a confused way: I suggest you reorganise the text on Plink and the command lines that you used all together in a final couple of sentences on software implementation
Response: Thank you for your suggestion. We have rearranged the order of descriptions, see L214-225.

>L219: P is the body weight (you could directly write BW instead of P)
Response: Thank you for your suggestion. We have replaced the “P” to “BW” in the analysis model and related information.

>L219-220: it is not clear what Z*alpha is: this seems to be the specification of a random polygenic (multigene) effect, with Z being the incidence matrix and alpha the multigene effect. This would then need an associated variance component, e.g. sigma^2_g (genetic variance) with a kinship matrix (genetic relationships between individuals). However, you first mention PCs, which are used to account for population structure in GWAS, but then PCs do not appear in the specification of the GWAS model. Additionally, I don't think that you can fit a polygenic effect with a covariance matrix (mixed model) in Plink: if you did, please report the command line that you used, and which was the kinship matrix that you used as covariance (e.g. VanRaden, Astle & Balding etc.)
Response: Thank you for your comments. There are two types of plink correlation analysis. The analysis method with the parameter "--assoc" has no covariates and run fast, with the following parameters: --assoc --allow-extra-chr --allow-no- sex. And the other analysis sets the parameter '--linear'. First assoc analysis in plink with sample variants and corresponding weight information, i.e. asymptotic Wald test analysis. Linear analysis allows for covariates and runs slowly, using the top 20 pc's in the PCA analysis as covariates, PCA analysis with the following parameters: --pca --allow-extra-chr --allow-no-sex. And the GWAS parameters are as follows: --linear --allow-extra-chr --allow-no-sex --covar plink.eigenvec

>L222-224: Bonferroni corrects the threshold (or, equivalently, the SNP p-values) by the number of tests performed (i.e. the number of SNPs tested in GWAS). I don't understand the reference to a "further 20-fold expansion": can you please report the final threshold for significance that you obtain after all these corrections? This is needed to assees your results
Response: Thank you very much for your suggestion, we have changed the unclear expression to the following: Genome-wide -log10(10-6) significance threshold was determined using the Bonferrorni method. To reduce false negative, the threshold was expanded to -log10(5-8) as a second threshold and the SNP in this region was defined as potentially associated.

Close
