# Supplementary material for: Chromosome-level genome assembly of goose provides insight into the adaptation and growth of local goose breeds
Source: Gigascience. 2023 Feb 3;12:giad003. doi: 10.1093/gigascience/giad003 (PMC9896136; doi:10.1093/gigascience/giad003)
Supplement: giad003_Response_to_Reviewer_Comments_Revision_2 [file giad003_response_to_reviewer_comments_revision_2.pdf]

Author's Response To Reviewer Comments

Close

Dear editor,

Thank you very much for your letter dated 19 Sep 2022, and the reviewer’s comments concerning our manuscript “Chromosome-level genome assembly of goose provides insight into the adaptation and growth of local goose breeds” (ID: GIGA-D-22-00016).

These comments are of great value and very helpful for revising and improving our paper, as well as the importance guiding significant to our research. According to your opinion and request, we have made some revisions to the original manuscript. The responses to the questions are shown below, the black font part is the questions raised by the reviewers, and the dark blue font part is our reply.

We have resubmitted the revised version in both PDF and MS word format, on the system for your review. The revised parts are marked in yellow in the MS word file of the revised manuscript for your review.

Should you have any questions, please contact us without hesitate.

Best wishes,
Xinheng Zhang

Questions and Responses:

Reviewer reports:
Reviewer #3: There are still important aspects of your approach which are not clear, and raise doubts as to how the results where produced and are to be interpreted, and also as to your full appreciation of the in's and out's of GWAS models. Please take in serious consideration the remarks below, if needed you can also consult somebody with experience in the theory and practice of GWAS.

- The specification of the model for GWAS is still wrong, and contains several incorrect statements. Extensive corrections are needed
- Also the software implementation for GWAS gives rise to doubts: Plink does not allow for mixed models, so what about your model? There could not be the Z*alpha term, with the associated variance component. Please check carefully, because either the model equation you report in the text (L217) is not the one you used, or you used a different software.

L138-139: please add more details on how the Perl script hybridScaffold.pl solved the conflict by performing the split
Response: Thank you for your comments, which we have refined as described below: The nature of the conflict is that there are excessive numbers of unmatched markers on the optical and physical maps. To address this problem, hybrid assemblies were processed using Hybrid Scaffold. Firstly, a pattern search of the genomic sequence was performed to find possible cleavage labels, and the number of labels on matched and unmatched pairs in each linkage was counted and their positions was recorded. Supervised processing is then performed to resolve the positions with conflicting match, then RefAligner is called for iterative sequences merge by pairwise alignment. Finally, the sequence map and genome map are re-matched to the hybrid scaffold and checked again.

L156: please choose between "adapter" and "adaptor"
Response: Thank you for your careful guidance, we have checked and corrected the “adaptor” to “adapter”

in full manuscript.

L163-165: please report what you mentioned in the response to my comment also in text: explain that a higher ratio of the mapped intact genes in the assembled genome means a higher completeness/quality/integrity of the assembled genome
Response: Thank you for your comment, we have checked carefully and found that this was a calculation error on our part and have made a correction: We then evaluated the assembled genome with 95.21% single-copy and 1.70% duplicated orthologs from the BUSCO dataset, confirming that 8,081 genes (96.92%) were intact in this genome.

L213: you need to add a reference to Plink. Moreover, which version of Plink did you use?
Response: Thanks to your suggestion, we have added the version number (v1.90b6.21) and the reference (Purcell S et al., 2007) to the text.

L214: " ... covariates in the linear model model for the genotype-phenotype association analysis: BW = mu + Zalpha + SNP + e "
L215: delete "The statistical analysis model for genome-wide association analysis was as follows"
L217: in the model equation, I think that you are using matrix notation: this means that mu must be preceded by a vector of 1's, and SNP must be preceded by the corresponding incidence matrix X (indicating for instance the n. of copies of the minor allele in each individual - this is one possible parameterization)
L218: BW is the vector of goose body weights;
L218: Z is not the relationship matrix! Z is the incidence matrix, relating each polygenic effect alpha_i to each individual goose i (probably in your case a diagonal identity matrix). The relationship matrix comes into play in the variance of y, specifically of the polygenic (random multigene) effect alpha --> Var(alpha) = G*sigma_a^2.
L219: the SNP effect should be the SNP effect, i.e. the effect for which you are trying to estimate the magnitude and the significance (is the SNP associated with BW?). The SNP effect is specified with an associated design matrix that relates individuals and genotypes/alleles. If you used principal components as covariates in the GWAS model, you need to add an extra term for this
L220: I is not the unit matrix (a matrix of 1's), but it is the idetity matrix (a diagonal matrix with 1's only in the diagonal)
L220-221: if you used Plink to perform the association analysis, I think that you could not fit the polygenic/mutligene effect, since Plink does not allow to use mixed models with random effects and associated variance components. Please check!!

Response: With regard to the questions relating to GWAS, thank you very much for your professional guidance. In response, we have consulted with the relevant professional technicians and have concluded that there is nothing wrong with our workflow, but only a significant deviation in the textual description in the article, so we have made a complete revision of this section with a view to resolving your confusion. We have sorted out that part of the description, as described below: Based on the SNP set obtained above, the genetic variation was analyzed with individual corresponding body weight information using the two separate and independent models to assess the significance of SNP effects in Plink (v 1.90b6.21) [38]. In the first model, top 20 PCs in the PCA analysis were used as covariates, and a linear analysis was performed on sample variances with the following parameters: --linear --allow-extra-chr --allow-no-sex --covar. In the second model, an asymptotic Wald test analysis was carried out with the following parameters: --assoc --allow-extra-chr --allow-no-sex. Finally, SNPs with Bonferroni corrected p-values less than 0.05 were taken as significant loci in the SNPs obtained from the two models above, and these loci were annotated. The annotated genes were subjected to GO enrichment analysis using the genomic genes of Lion-head goose as background.
For the statistical model problem, we rechecked the relevant information and found that the model should be
Y = b0 + b1. ADD + b2. COV1 + b3. COV2 + ... + b21. COV20 + b22. ADDxCOV1 + b23. ADDxCOV2 + ... b41. ADDxCOV20 + e;
where b0~b41 are coefficients, COV x are the top 20 PCs of the PCA as the covariates; e is the residual.

L223: when you use Bonferroni correction, you can either divide the threshold (e.g. alpha = 0.05) by the

number of independent tests (e.g. the n. of SNP markers), or you can keep alpha an multiply the p-values obtained from the GWAS model (p-value <= alpha/m). Which one did you do? Did you start with alpha = 10^-6 and then applied Bonferroni correction to this initial threshold? What was the number of test (markers) "m" that you used for correction?
Response: Thank you for your comments. We keep alpha a multiply the p-values obtained from the GWAS model (p-value ≤ alpha/m). Number of test (markers) "m" is the number of population SNPs used in GWAS (89716). Bonferroni correction was done by the function p.adjust() in R.

Close
