# Supplementary material for: Chromosome-level genome assembly of goose provides insight into the adaptation and growth of local goose breeds
Source: Gigascience. 2023 Feb 3;12:giad003. doi: 10.1093/gigascience/giad003 (PMC9896136; doi:10.1093/gigascience/giad003)
Supplement: giad003_Reviewer_1_Report_Original_Submission — Guangliang Gao bang Luo -- 4/1/2022 Reviewed [file giad003_reviewer_1_report_original_submission.pdf]

Reviewer Report

Title: Chromosome-level genome assembly of goose provides insight into the adaptation and growth of local goose breeds

Version: Original Submission **Date:** 4/1/2022

Reviewer name: Guangliang Gao

Reviewer Comments to Author:

Annotation and assembly of the Lion-headed goose genome were performed using a combination of four technologies, including Illumina, SMRT, Bionano, and Hi-C. Based on the chromosome-level genome sequence, a genome-wide association study (GWAS) was performed on 514 geese including Wuzong goose, Huangzong goose, Magang goose, and Lion-head goose, yielding an average of 1.52 Gb raw data, identifying 44,858 SNPs The GWAS results showed that six SNPs were significantly associated with body weight and 25 were potentially associated.
The authors should explore the spatial organization of chromatin and gene expression in the goose blood tissue (inter-pseudo-chromosomal interaction patterns, compartments, topologically associating domains, and promoter-enhancer interactions), to check if the goose genome shows similar basic principles to other animal genomes in terms of its inter-chromosomal interaction pattern, compartments, topologically associating domains, and promoter-enhancer interactions. Providing basic characterization of the three-dimensional organization of the goose genome, and supports the conclusion the goose genome assembly is chromosomal-level. For the four goose population, the authors should perform selective sweep analysis (Fstã€�XP-CLRã€�Ï€ or Tajima's D), and combine with the GWAS results to illustrate the topic of the this article.
Line 27, "and identifying 21,208 protein-coding genes". Previous studies have shown that there are 16,150, 16288 and 17568 genes in Zhedong White goose, Sichuan white goose and Tianfu goose genome, respectively, please illustrate reason why the gene number were different the results from previous studies.
Line 27, "…generating 40 pseudochromosomes", the assignment of 40 chromosomes to Hi-C scaffolds is very tentative and needs to be validated, the 40 pseudo-chromosomes do not equate to the 40 physical chromosomes. Moreover, the result is conflict with the 39 pseudochromosomes in Tianfu goose genome, how did the authors confirm the number of chromosomes?
Line 33, "…an average of 1,520.6 Mb of raw data with detecting 44,858 SNPs". Based on whole-genome resequencing data, researchers have identified 9,279,339 SNPs in the goose genome using an average depth of 12.44Ã— whole genome resequencing data. Referring to SNP number, it is uncertain whether the results in this study (44,858 SNPs generated from 1,5Gb data) is correct. Therefore, the authors should apply the BWA-GATK pipeline to Tianfu meat goose and Lion-head goose for GWAS analysis to determine whether the results are correct.
Line 61 to 65, It is recommended to rewrite or replace the descriptions for the goose breeds with methods sections.
Methods section

Line 88, Provide a detailed description of the picture(s) for the Lion-head goose to display the "classical trails".
Please supply the pictures for the four goose breed (Wuzong goose, Huangzong goose, Magang goose and Lion-head goose) to help the more clear the understanding of design.
Line 91 to 92, "from another four healthy adult accessions were collected for RNA-seq analysis", please rewrite the sentence since it is unclear.
Supply the detail information for GWAS analysis, including the software, models
What parameters were used to run GATK, plink, BWA? did the authors performed GWAS analysis using plink software, rather than GEMMA, TASSEL or other software ?
line 200 "the results of the assoc and linear analyses were…", supply the detail of GWAS analysis, including the software, analysis model. please provide more detailed information about the models and assumptions.
What the top 20 PCs? Did the PCs paly an important role in GWAS analysis?
Detailed information is not given in several parts of this paper, especially the methodology. How many individuals from the four-goose population? The GWAS analysis were performed in one goose population or the four-goose population? How did the authors do the GWAS analysis and annotation the SNPs? please supply detail analysis steps and analysis models, software. For GWAS analysis model, were there any family or environmental effects? how did you test the significance of the random variables?
Many sentences are not clear all over the entire manuscript and need to be re-writen. For instance, line 201, "The corresponding genes of significantly related SNPs were used to identify the GO pathway", define the corresponding genes, and how did the GO pathway analysis?
Line 203, please rewrite the statistical analysis section to provide more detail. For example, authors should define "potential associated" in this section.
Line 283 to 284, "…correlated with any chromosome of the duck genome due to the presence of a large number of tandem repeats". Provide the detail data or the figure(s) to support your claim.
Results section
Compare with the quality metrics of this study with the previous four goose genome, including contig N50, scaffold N50, gene number, Repetitive regions proportion of genome, etc.
For gene annotation, the authors did not perform the none coding RNA in the goose genome, please supply the analysis.
The author(s) should perform the positive selection genes analysis with the avian chromosome genomes, such as chicken, duck, zebra finch, etc.
Please supply the detail information of the 40 pseudo-chromosomes for the goose genome assembly.
Please show the summary of the economic traits used in this study, including the mean, stand error, numbers of individuals, breed, male or female.
line 233-234, "The aggregate of 760 Gb raw reads was accumulated by the paired-end sequencing of the 36 constructed libraries", Why did the authors conduct 760 Gb RNAseq? It is obvious too much larger than previous goose genome annotation, did they perform more analysis?
Line 286 to 287, "Chr 4 of Lion-head goose was found to correspond to the sex chromosome Z of duck, except for the inversions of small patches of segments; therefore, we inferred that Chr 4 was the sex chromosome of the Lion-head goose", To better understand the unique biological characteristics and breeding of geese, it is essential to distinguish the sex chromosomes from the autosomes. For updating

the sequence of Z and W chromosomes, it is recommended to filter the sequence of autosomes using experimental methods. How did the authors filter autosomal sequences in the Chr4? Moreover, the W chromosome sequence should be identified similarly to the Z chromosome. Authors should identify the Z and W chromosome sequence from public databases based on the Z and W chromosome sequence from the chromosome-level avian genome.
Line 292-294, "and their weight was recorded, with the Lion-head goose using the minimum weight, the Wuzong goose using the maximum weight, and the Huangzong goose and Magang goose using the average weight." Why did the authors select the body weight trait? The artificial selection would lead to the inaccurate GWAS results.
From figure 5A, there are significant population stratification in Lion goose population (obvious clustering 2 clusters), how did the authors sure to provide accurate GWAS results? Did the author detect the SNPs associated with body weight in the goose population to test the accurate of GWAS results?
The discussion tends to be mere story telling.
Tables and Figures
In table 1, the "Hi-C" results is repeat with the "Assembly", please modify it.
The table 2-4, Figure 1-2, are not very informative and I suggest moving these to the supplementary information.

Methods

Are the methods appropriate to the aims of the study, are they well described, and are necessary controls included? Choose an item.

Conclusions

Are the conclusions adequately supported by the data shown? Choose an item.

Reporting Standards

Does the manuscript adhere to the journal's guidelines on minimum standards of reporting? Choose an item.

Choose an item.

Statistics

Are you able to assess all statistics in the manuscript, including the appropriateness of statistical tests used? Choose an item.

Quality of Written English

Please indicate the quality of language in the manuscript: Choose an item.

Declaration of Competing Interests

Please complete a declaration of competing interests, considering the following questions:

- Have you in the past five years received reimbursements, fees, funding, or salary from an organisation that may in any way gain or lose financially from the publication of this manuscript, either now or in the future?
- Do you hold any stocks or shares in an organisation that may in any way gain or lose financially from the publication of this manuscript, either now or in the future?
- Do you hold or are you currently applying for any patents relating to the content of the manuscript?
- Have you received reimbursements, fees, funding, or salary from an organization that holds or has applied for patents relating to the content of the manuscript?
- Do you have any other financial competing interests?
- Do you have any non-financial competing interests in relation to this paper?

If you can answer no to all of the above, write 'I declare that I have no competing interests' below. If your reply is yes to any, please give details below.

I declare that I have no competing interests.

I agree to the open peer review policy of the journal. I understand that my name will be included on my report to the authors and, if the manuscript is accepted for publication, my named report including any attachments I upload will be posted on the website along with the authors' responses. I agree for my report to be made available under an Open Access Creative Commons CC-BY license (http://creativecommons.org/licenses/by/4.0/). I understand that any comments which I do not wish to be included in my named report can be included as confidential comments to the editors, which will not be published.

Choose an item.

To further support our reviewers, we have joined with Publons, where you can gain additional credit to further highlight your hard work (see: https://publons.com/journal/530/gigascience). On publication of this paper, your review will be automatically added to Publons, you can then choose whether or not to claim your Publons credit. I understand this statement.

Yes Choose an item.
