# Supplementary material for: Chromosome-level genome assembly of goose provides insight into the adaptation and growth of local goose breeds
Source: Gigascience. 2023 Feb 3;12:giad003. doi: 10.1093/gigascience/giad003 (PMC9896136; doi:10.1093/gigascience/giad003)
Supplement: giad003_Reviewer_3_Report_Original_Submission — Filippo Biscarini -- 4/7/2022 Reviewed [file giad003_reviewer_3_report_original_submission.pdf]

Reviewer Report

Title: Chromosome-level genome assembly of goose provides insight into the adaptation and growth of local goose breeds

Version: Original Submission **Date:** 4/7/2022

Reviewer name: Filippo Biscarini

Reviewer Comments to Author:

Zhao et al.
Title: "Chromosome-level genome assembly of goose provides insight into the adaptation and growth of local goose breeds"
- please place figures and tables within the text, not at the end: this makes it difficult to read and review the article
- English: needs to be improved
- Figures: low quality, low resolution --> hard to read. Additionally, Figure legends/captions are separate from Figures --> difficult reading
Introduction

L44: what do you mean with "the majority of birds"?
L51: warmth retention of the birds?
L63: " ... while IN the Wuzong goose ... the average weight is ..."
L67: how is an accurate reference genome essential to decipher the industry's development? Which industry?
L73: maybe it's new scaffolding techniques
L80-81: here you mention two sequencing technologies (SMRT, Illumina NGS), one scaffolding method (Hi-C) and one unspecified technology/methods by Bionano (which was not mentioned earlier). Please rephrase and be more specific and clearer
L81: correlation of body weight with what?
Methods

L119-181: how were Bionano maps used to improve the quality of your genome assembly?
L136: adult accessions?
L142: how were low-quality reads defined? Based on average Phred scores?
L143: what do you mean by polluted reads?
L143: what do you mean with "Trinity was arranged"?
L148-150: poor English, please rephrase. Additionally, more details are needed on the quality metrics used to evaluate the assembled genome
L164-165: could you add the scientific names (genus species) of the mentioned avian species? (green lizard is not an avian species)
L173: from where did you get the divergence time between turkeys and pigeons? (~100 million years?

Really?) And why did you choose this specific divergence value for calibration?
L173: the r8s software was served to estimate: bad English
L179: "Experimental sample processing and genotyping" this is a bit unclear: you already took biological samples, maybe you need to highlight that this is genotyping (your title should be more about genotyping and phenotyping for GWAS, since you spend the first few lines of the paragraph to describe the phenotypes)
L181-185: body weight is naturally a continuous trait, it would be rather arbitrary to split it into categories: therefore I don't understand this whole bit on categorical vs continuous body weight
L186-190: what you describe is RAD-sequencing/GBS/resequencing, not "genotyping". By genotyping usually an array-based approach is meant
L188: how did you define low quality reads here? (Phred scores?) No filters on average reads coverage per site?
L191: it is not clear which variants were called? SNP? MNP? Indels? All? etc.
L191: why did you set the MAF threshold at 5%? You have 514 samples, with a filter at MAF 1% you'd still have more than 10 copies of the minor allele in the worst case scenario
L192: maximum deletion threshold? Is this max missing rate?
L192-193: what was the objective of PCA? PCA on which data? (I guess the genotype data? Which?)
L193-194: "To understand the kinship among the samples, and phylogenetic trees were constructed." This sentence seems wrong/incomplete
L196: maybe you mean genetic variation?
L197: did you use the --linear option in Plink?
L197-199: this sentence is poorly written, please rephrase
L199: I guess its the variants, not the variances (if it is SNPs, please say SNPs)
L196-200: I think it would be better if you wrote the GWAS model explicitly (the model equation)
L200: why did you choose Bonferroni correction over other methods to control for spurious results (e.g. FDR, Bayesian odds, permutation test, q-values etc.)
L202: this part is useless, as it is: which statistical analysis? Why did you choose the 0.05 threshold for significance? (you just said above that you used Bonferroni corrected p-values for GWAS)
Results

L211: "Assemble these data step by step and produce progressively improved assemblies (Fig. 1A)." This sentence seems incomplete or wrong

Methods

Are the methods appropriate to the aims of the study, are they well described, and are necessary controls included? Choose an item.

Conclusions

Are the conclusions adequately supported by the data shown? Choose an item.

Reporting Standards

Does the manuscript adhere to the journal's guidelines on minimum standards of reporting? Choose an item.

Choose an item.

Statistics

Are you able to assess all statistics in the manuscript, including the appropriateness of statistical tests used? Choose an item.

Quality of Written English

Please indicate the quality of language in the manuscript: Choose an item.

Declaration of Competing Interests

Please complete a declaration of competing interests, considering the following questions:

- Have you in the past five years received reimbursements, fees, funding, or salary from an organisation that may in any way gain or lose financially from the publication of this manuscript, either now or in the future?
- Do you hold any stocks or shares in an organisation that may in any way gain or lose financially from the publication of this manuscript, either now or in the future?
- Do you hold or are you currently applying for any patents relating to the content of the manuscript?
- Have you received reimbursements, fees, funding, or salary from an organization that holds or has applied for patents relating to the content of the manuscript?
- Do you have any other financial competing interests?
- Do you have any non-financial competing interests in relation to this paper?

If you can answer no to all of the above, write 'I declare that I have no competing interests' below. If your reply is yes to any, please give details below.

I declare that I have no competing interests

I agree to the open peer review policy of the journal. I understand that my name will be included on my report to the authors and, if the manuscript is accepted for publication, my named report including any attachments I upload will be posted on the website along with the authors' responses. I agree for my report to be made available under an Open Access Creative Commons CC-BY license (http://creativecommons.org/licenses/by/4.0/). I understand that any comments which I do not wish to be included in my named report can be included as confidential comments to the editors, which will not be published.

Choose an item.

To further support our reviewers, we have joined with Publons, where you can gain additional credit to further highlight your hard work (see: https://publons.com/journal/530/gigascience). On publication of this paper, your review will be automatically added to Publons, you can then choose whether or not to claim your Publons credit. I understand this statement.

Yes Choose an item.
