# Supplementary material for: Chromosome-level genome assembly of goose provides insight into the adaptation and growth of local goose breeds
Source: Gigascience. 2023 Feb 3;12:giad003. doi: 10.1093/gigascience/giad003 (PMC9896136; doi:10.1093/gigascience/giad003)
Supplement: giad003_Reviewer_3_Report_Revision_1 — Filippo Biscarini -- 8/8/2022 Reviewed [file giad003_reviewer_3_report_revision_1.pdf]

Reviewer Report

Title: Chromosome-level genome assembly of goose provides insight into the adaptation and growth of local goose breeds

Version: Revision 1 **Date:** 8/8/2022

Reviewer name: Filippo Biscarini

Reviewer Comments to Author:

L44: most birds of the Anseriformes order
L51: warmth properties still doesn't sound right: maybe thermal (or thermic?) properties?
L76: what do you mean with "continuous reference genome"?
L77-78: the link between the reference genome and the development of the goos industry is still loose: maybe you want to say that a complete and more accurate genome would make it possible to develop better tools for good breeding (e.g. genetic markers for marker-assisted selection, genomic breeding values, precise estimates of inbreeding, relatedness matrices between individuals etc.) Is this what you have in mind?
L130: replace "At last" with Finally
L139: how was the contig split? Based on which criteria? (e.g. one half aligned in one region of the genome, the other half aligned somewhere else on the genome?)
L156: why do you say "polluted reads"? Do you mean contaminated samples? Do you have evidence that some of your samples were contaminated (i.e. external non-goos DNA)? uncalled nucelotides (the N's) can arise also from reading errors when generating the reads.
L163-164: "quality control for the assembly's quality, accuracy, and integrity was predicted": it is not clear what you predicted, please clarify (and write in better English please)
L165: at least say that you used default parameters (and add a reference to these, e.g. the online manual)
L203: what is this low quality parameter? Some sort of modified Phred? (A Phred threshold of 5 would be a bit low, allowing many errors -wrong bases- in the analysis)
L209: maybe it is better to write "To understand relationships among groups of samples, the phylogenetic ..."
L212: corresponding BODY weight
L213: Wald test is one of many possible statistical tests to assess the significance of SNP effects from the results of the linear regression model used for the association study
L213: The top 20 principal components PCs) from the principal components analysis (PCA) of SNP variant data were used as covariates in the model used for the association study.
L214: you can delete this (you already mentioned Plink, or can mention Plink at the end of the GWAS section)
L215-216: this is written in a confused way: I suggest you reorganise the text on Plink and the command lines that you used all together in a final couple of sentences on software implementation
L219: P is the body weight (you could directly write BW instead of P)

L219-220: it is not clear what Z*alpha is: this seems to be the specification of a random polygenic (multigene) effect, with Z being the incidence matrix and alpha the multigene effect. This would then need an associated variance component, e.g. sigma^2_g (genetic variance) with a kinship matrix (genetic relationships between individuals). However, you first mention PCs, which are used to account for population structure in GWAS, but then PCs do not appear in the specification of the GWAS model. Additionally, I don't think that you can fit a polygenic effect with a covariance matrix (mixed model) in Plink: if you did, please report the command line that you used, and which was the kinship matrix that you used as covariance (e.g. VanRaden, Astle & Balding etc.)
L222-224: Bonferroni corrects the threshold (or, equivalently, the SNP p-values) by the number of tests performed (i.e. the number of SNPs tested in GWAS). I don't understand the reference to a "further 20-fold expansion": can you please report the final threshold for significance that you obtain after all these corrections? This is needed to assees your results

Methods

Are the methods appropriate to the aims of the study, are they well described, and are necessary controls included? Choose an item.

Conclusions

Are the conclusions adequately supported by the data shown? Choose an item.

Reporting Standards

Does the manuscript adhere to the journal's guidelines on [minimum standards of reporting?](minimum standards of reporting) Choose an item.

Choose an item.

Statistics

Are you able to assess all statistics in the manuscript, including the appropriateness of statistical tests used? Choose an item.

Quality of Written English

Please indicate the quality of language in the manuscript: Choose an item.

Declaration of Competing Interests

Please complete a declaration of competing interests, considering the following questions:

- Have you in the past five years received reimbursements, fees, funding, or salary from an organisation that may in any way gain or lose financially from the publication of this manuscript, either now or in the future?

- Do you hold any stocks or shares in an organisation that may in any way gain or lose financially from the publication of this manuscript, either now or in the future?
- Do you hold or are you currently applying for any patents relating to the content of the manuscript?
- Have you received reimbursements, fees, funding, or salary from an organization that holds or has applied for patents relating to the content of the manuscript?
- Do you have any other financial competing interests?
- Do you have any non-financial competing interests in relation to this paper?

If you can answer no to all of the above, write 'I declare that I have no competing interests' below. If your reply is yes to any, please give details below.

I declare that I have no competing interests

I agree to the open peer review policy of the journal. I understand that my name will be included on my report to the authors and, if the manuscript is accepted for publication, my named report including any attachments I upload will be posted on the website along with the authors' responses. I agree for my report to be made available under an Open Access Creative Commons CC-BY license (http://creativecommons.org/licenses/by/4.0/). I understand that any comments which I do not wish to be included in my named report can be included as confidential comments to the editors, which will not be published.

Choose an item.

To further support our reviewers, we have joined with Publons, where you can gain additional credit to further highlight your hard work (see: https://publons.com/journal/530/gigascience). On publication of this paper, your review will be automatically added to Publons, you can then choose whether or not to claim your Publons credit. I understand this statement.

Yes Choose an item.
