# Supplementary material for: Chromosome-level genome assembly of goose provides insight into the adaptation and growth of local goose breeds
Source: Gigascience. 2023 Feb 3;12:giad003. doi: 10.1093/gigascience/giad003 (PMC9896136; doi:10.1093/gigascience/giad003)
Supplement: giad003_Reviewer_3_Report_Revision_2 — Filippo Biscarini -- 9/16/2022 Reviewed [file giad003_reviewer_3_report_revision_2.pdf]

Reviewer Report

Title: Chromosome-level genome assembly of goose provides insight into the adaptation and growth of local goose breeds

Version: Revision 2 **Date:** 9/16/2022

Reviewer name: Filippo Biscarini

Reviewer Comments to Author:

There are still important aspects of your approach which are not clear, and raise doubts as to how the results where produced and are to be interpreted, and also as to your full appreciation of the in's and out's of GWAS models. Please take in serious consideration the remarks below, if needed you can also consult somebody with experience in the theory and practice of GWAS.
- The specification of the model for GWAS is still wrong, and contains several incorrect statements. Extensive corrections are needed
- Also the software implementation for GWAS gives rise to doubts: Plink does not allow for mixed models, so what about your model? There could not be the Z*alpha term, with the associated variance component. Please check carefully, because either the model equation you report in the text (L217) is not the one you used, or you used a different software.
L138-139: please add more details on how the Perl script hybridScaffold.pl solved the conflict by performing the split
L156: please choose between "adapter" and "adaptor"
L163-165: please report what you mentioned in the response to my comment also in text: explain that a higher ratio of the mapped intact genes in the assembled genome means a higher completeness/quality/integrity of the assembled genome
L213: you need to add a reference to Plink. Moreover, which version of Plink did you use?
L214: " ... covariates in the linear model model for the genotype-phenotype association analysis: BW = mu + Zalpha + SNP + e "
L215: delete "The statistical analysis model for genome-wide association analysis was as follows"
L217: in the model equation, I think that you are using matrix notation: this means that mu must be preceded by a vector of 1's, and SNP must be preceded by the corresponding incidence matrix X (indicating for instance the n. of copies of the minor allele in each individual - this is one possible parameterization)
L218: BW is the vector of goose body weights;
L218: Z is not the relationship matrix! Z is the incidence matrix, relating each polygenic effect alpha_i to each individual goose i (probably in your case a diagonal identity matrix). The relationship matrix comes into play in the variance of y, specifically of the polygenic (random multigene) effect alpha --> Var(alpha) = G*sigma_a^2.
L219: the SNP effect should be the SNP effect, i.e. the effect for which you are trying to estimate the magnitude and the significance (is the SNP associated with BW?). The SNP effect is specified with an associated design matrix that relates individuals and genotypes/alleles. If you used principal

components as covariates in the GWAS model, you need to add an extra term for this
L220: I is not the unit matrix (a matrix of 1's), but it is the idetity matrix (a diagonal matrix with 1's only in the diagonal)
L220-221: if you used Plink to perform the association analysis, I think that you could not fit the polygenic/mutligene effect, since Plink does not allow to use mixed models with random effects and associated variance components. Please check!!
L223: when you use Bonferroni correction, you can either divide the threshold (e.g. alpha = 0.05) by the number of independent tests (e.g. the n. of SNP markers), or you can keep alpha an multiply the p-values obtained from the GWAS model (p-value <= alpha/m). Which one did you do? Did you start with alpha = 10^-6 and then applied Bonferroni correction to this initial threshold? What was the number of test (markers) "m" that you used for correction?

Methods

Are the methods appropriate to the aims of the study, are they well described, and are necessary controls included? Choose an item.

Conclusions

Are the conclusions adequately supported by the data shown? Choose an item.

Reporting Standards

Does the manuscript adhere to the journal's guidelines on minimum standards of reporting? Choose an item.

Choose an item.

Statistics

Are you able to assess all statistics in the manuscript, including the appropriateness of statistical tests used? Choose an item.

Quality of Written English

Please indicate the quality of language in the manuscript: Choose an item.

Declaration of Competing Interests

Please complete a declaration of competing interests, considering the following questions:

- Have you in the past five years received reimbursements, fees, funding, or salary from an organisation that may in any way gain or lose financially from the publication of this manuscript, either now or in the future?

- Do you hold any stocks or shares in an organisation that may in any way gain or lose financially from the publication of this manuscript, either now or in the future?
- Do you hold or are you currently applying for any patents relating to the content of the manuscript?
- Have you received reimbursements, fees, funding, or salary from an organization that holds or has applied for patents relating to the content of the manuscript?
- Do you have any other financial competing interests?
- Do you have any non-financial competing interests in relation to this paper?

If you can answer no to all of the above, write 'I declare that I have no competing interests' below. If your reply is yes to any, please give details below.

'I declare that I have no competing interests'

I agree to the open peer review policy of the journal. I understand that my name will be included on my report to the authors and, if the manuscript is accepted for publication, my named report including any attachments I upload will be posted on the website along with the authors' responses. I agree for my report to be made available under an Open Access Creative Commons CC-BY license (http://creativecommons.org/licenses/by/4.0/). I understand that any comments which I do not wish to be included in my named report can be included as confidential comments to the editors, which will not be published.

Choose an item.

To further support our reviewers, we have joined with Publons, where you can gain additional credit to further highlight your hard work (see: https://publons.com/journal/530/gigascience). On publication of this paper, your review will be automatically added to Publons, you can then choose whether or not to claim your Publons credit. I understand this statement.

Yes Choose an item.
